# Genotyping and population characteristics of the China Kadoorie Biobank

**DOI:** 10.1016/j.xgen.2023.100361

**Published:** 2023-07-20

**Authors:** Robin G. Walters, Iona Y. Millwood, Kuang Lin, Dan Schmidt Valle, Pandora McDonnell, Alex Hacker, Daniel Avery, Ahmed Edris, Hannah Fry, Na Cai, Warren W. Kretzschmar, M. Azim Ansari, Paul A. Lyons, Rory Collins, Peter Donnelly, Michael Hill, Richard Peto, Hongbing Shen, Xin Jin, Chao Nie, Xun Xu, Yu Guo, Canqing Yu, Jun Lv, Robert J. Clarke, Liming Li, Zhengming Chen, China Kadoorie Biobank Collaborative Group

**Affiliations:** 1 Nuffield Department of Population Health, University of Oxford, Oxford OX3 7LF, UK; 2 MRC Population Health Research Unit, University of Oxford, Oxford OX3 7LF, UK; 3 Wellcome Centre for Human Genetics, University of Oxford, Oxford OX3 7BN, UK; 4 Nuffield Department of Medicine, Oxford University, Oxford OX1 3SY, UK; 5 NIHR Oxford Biomedical Research Centre, Oxford University Hospitals NHS Foundation Trust, Oxford OX3 9DU, UK; 6 Cambridge Institute for Therapeutic Immunology and Infectious Disease, University of Cambridge, Cambridge CB2 0AW, UK; 7 Department of Medicine, University of Cambridge, Cambridge CB2 0QQ, UK; 8 Department of Epidemiology, Collaborative Innovation Center for Cancer Medicine, Nanjing Medical University, Nanjing 211116, China; 9 BGI-Shenzhen, Shenzhen 518083, China; 10 Fuwai Hospital, Chinese Academy of Medical Sciences, Beijing 100037, China; 11 Department of Epidemiology and Biostatistics, School of Public Health, Peking University, Beijing 100191, China; 12 Center for Public Health and Epidemic Preparedness and Response, Peking University, Beijing 100191, China

**Keywords:** biobank, prospective, complex disease, genotyping, genetics, cardiovascular health, genetic epidemiology, genetic association studies, GWAS, omics

## Abstract

The China Kadoorie Biobank (CKB) is a population-based prospective cohort of >512,000 adults recruited from 2004 to 2008 from 10 geographically diverse regions across China. Detailed data from questionnaires and physical measurements were collected at baseline, with additional measurements at three resurveys involving ∼5% of surviving participants. Analyses of genome-wide genotyping, for >100,000 participants using custom-designed Axiom arrays, reveal extensive relatedness, recent consanguinity, and signatures reflecting large-scale population movements from recent Chinese history. Systematic genome-wide association studies of incident disease, captured through electronic linkage to death and disease registries and to the national health insurance system, replicate established disease loci and identify 14 novel disease associations. Together with studies of candidate drug targets and disease risk factors and contributions to international genetics consortia, these demonstrate the breadth, depth, and quality of the CKB data. Ongoing high-throughput omics assays of collected biosamples and planned whole-genome sequencing will further enhance the scientific value of this biobank.

## Highlights

• The China Kadoorie Biobank is a very large, population-based, prospective cohort
• Comprehensive data and biosamples collected at baseline and during periodic resurveys
• Electronic linkage to death/disease registries and national health insurance system
• Genotyping of >100,000 participants enables GWASs of many diseases and risk factors

## Graphical abstract

**Figure:**
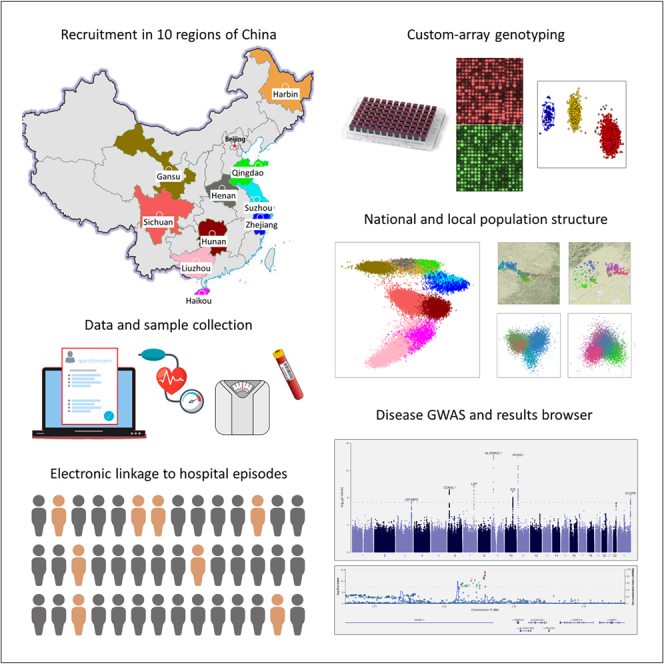

## Teaser

Walters et al. present genetic analyses of >100,000 participants of the China Kadoorie Biobank (CKB), a population-based, prospective cohort of >512,000 from 10 diverse regions of China. They describe how the CKB has contributed to understanding of the genetic basis for many diseases and risk factors and report GWASs of 124 diverse disease outcomes.

## Introduction

Major non-communicable chronic diseases, such as heart attack, stroke, cancer, and chronic obstructive pulmonary disease (COPD), account for much of the adult disease burden in China and globally. Several such diseases display large unexplained variations in incidence between different regions in China, indicating that important genetic and non-genetic causes remain to be discovered. The China Kadoorie Biobank (CKB) was initiated in 2002, with the goal of investigating the causal relevance of established and novel disease risk factors in the adult Chinese population.^1^ From 2004 to 2008, CKB recruited >512,000 adults aged 30–79 years from 10 geographically diverse (five urban, five rural) regions across China, making it one of the largest blood-based prospective biobanks in the world.^2^

Many aspects of the CKB study design support a wide range of hypothesis-driven and hypothesis-free research into many different diseases: population-based recruitment; prospective sample collection; a relatively medication-naive population; rich and diverse exposure and lifestyle data; and comprehensive capture of incident disease events through electronic linkage to death and disease registries and to health insurance records. The CKB also contributes to the growing demand for ancestrally diverse biobanks, which not only expand opportunities for novel discoveries of potential value to all human populations but also address potential inequalities in healthcare that may arise from the historical focus of research on individuals of European ancestry, findings from which are not necessarily transferable to other populations.^3^^,^^4^ In common with many other large biobanks, the value of the CKB has been greatly enhanced by large-scale genotyping of study participants. Such genotype information enables investigation of the contribution of genetic variation to phenotype and disease risk, Mendelian randomization (MR) assessment of the causal contribution of risk factors and behaviors to disease, and phenome-wide analyses of the impact of variation at specific loci.

We describe the design and performance of a custom Affymetrix Axiom array optimized for individuals of Chinese Han ancestry, which provides both genome-wide coverage to enable high-quality imputation of both common and low-frequency variation, and direct genotyping of ∼68,000 putative loss-of-function, missense, and expression quantitative trait loci (eQTL) variants of potential use for MR or phenome-wide association studies. On the basis of genotyping of >100,000 CKB participants, we demonstrate extensive population diversity across China, identify substantial relatedness within the CKB study population, and observe principal component (PC) signatures consistent with population movements from recent Chinese history. Through linkage to deep phenotyping and >1.2 million recorded disease outcomes in the CKB, this genotyping has already facilitated a wide range of studies, from investigation of ancestry-specific loss of function variants to inform drug target identification, validation, and repurposing^5^^,^^6^^,^^7^^,^^8^; to participation in international trans-ancestry genome-wide association study (GWAS) consortia including the Global Biobank Meta-Analysis Initiative (GBMI).^9^^,^^10^^,^^11^^,^^12^^,^^13^^,^^14^ We report the results of GWASs of 224 disease outcomes, which can be accessed through a CKB PheWeb browser.

## Study population and data collection

Recruitment of CKB participants was community-based, taking place at a large number of local assessment centers within each of 10 diverse regions of China (five rural counties and five urban districts), respectively referred to by the name of the province or city in which recruitment took place. At baseline assessment participants completed an extensive interviewer-administered questionnaire on factors including demographics and socio-economic status, diet and lifestyle (e.g., smoking, alcohol), physical activity, reproductive history (for women), and medical history and current medication. In addition, all participants underwent physical examination including measurements of anthropometrics, blood pressure, spirometry, exhaled carbon monoxide, and body composition (using bioimpedance). Furthermore, there were onsite blood tests of (non-fasting) glucose and hepatitis B virus (HBV) surface antigen, and blood samples were processed within a few hours to separate plasma and buffy coat for long-term storage.^2^

**Figure 1:**
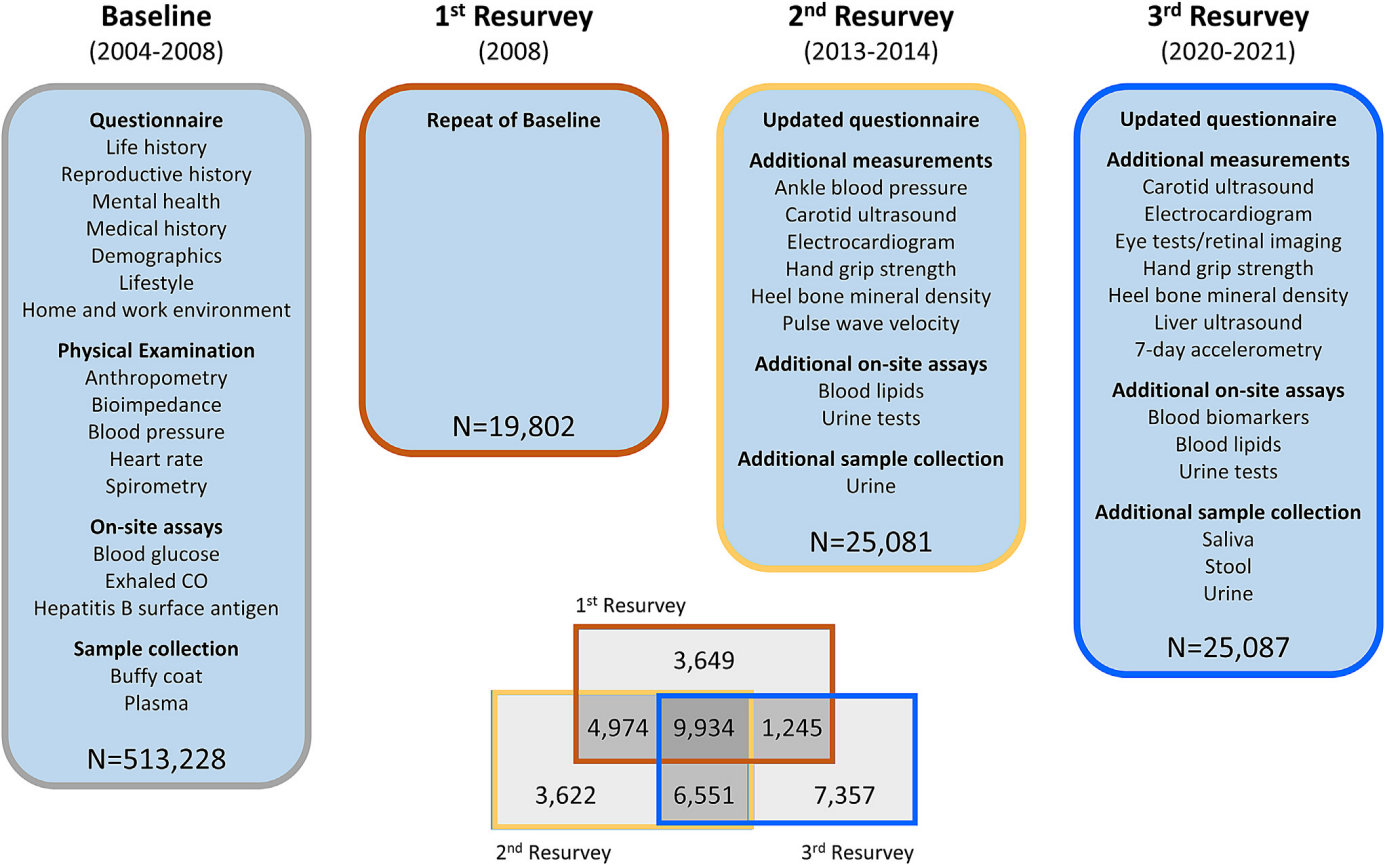
China Kadoorie Biobank (CKB) survey details Baseline questionnaire content, physical measurements, on-site assays, and biosample collection were repeated at three subsequent resurveys of approximately 5% of surviving participants. Second and third resurveys used updated questionnaires, included additional physical measurements and on-site assays, and collected additional biosamples, as shown. Participants attending more than one resurvey are as indicated in the Venn diagram. See also Table S1.

Subsequent to initial recruitment, three periodic resurveys have been undertaken of approximately 5% of surviving participants, selected on the basis of representative random samples of assessment centers, to provide repeat measurements for correction of regression dilution bias, gather additional questionnaire information, conduct additional physical measurements and blood tests, and collect repeat blood samples and other additional biosamples for long-term storage (Figure 1). The first resurvey of 19,802 participants, conducted immediately after completion of study recruitment in 2008, was largely a repeat of the baseline survey.^2^ This was extended in the second resurvey (25,091 participants), conducted in 2013 and 2014, with additional questionnaire data, additional physical measurements, on-site assays of blood lipids, and collection, testing, and storage of urine samples. Additional enhancements in the third resurvey (25,087 participants) in 2020 and 2021 included additional measurements of abdominal ultrasound and retinal imaging, and collection of saliva and stool samples. Baseline characteristics of resurvey participants were similar to those of the overall CKB cohort, with only minor differences attributable to survivor bias (Table S1). More than 22,000 individuals attended at least two resurveys; these multiple measurements at different time points will enable future longitudinal analyses of trajectories of risk factors for major diseases.

In addition to data collected at baseline and the resurveys, an increasing range of data are being generated from assays of stored biosamples. As part of a nested case-control study of stroke and ischemic heart disease (IHD), plasma samples from up to 18,728 participants (all with genotyping) were assayed for 17 clinical biochemistry measurements, with ^1^H nuclear magnetic resonance (NMR) metabolomics for 4,657; further ^1^H-NMR metabolomics measurements were conducted for other nested case-control studies of pancreatic cancer and diabetes (2,500 samples to date). Olink proteomics (3,072 proteins) and SomaLogic proteomics (up to 7,000 proteins) have recently been assayed for a further nested case-subcohort study of myocardial infarction (MI; 3,977 participants, all with genotyping), with additional larger scale measurements planned in the near future. Further assays include multiplex serology of antibodies to antigens from 19 pathogens (4,500 samples to date, with measurement in 40,000 cancer cases and controls under way) and ^1^H-NMR metabolomics of urine samples from 25,251 resurvey participants.

## Array design

CKB genotyping used custom-designed arrays on the Affymetrix (now Thermo Fisher Scientific) Axiom platform, with content selection based on similar overall principles to those used for the UK Biobank array design,^15^^,^^16^ but adapted to optimize performance for individuals of East Asian ancestry. This addressed three high-level criteria: (1) maximization of genome-wide coverage of common and low-frequency variation in individuals across the whole of China, (2) detection of important variants and of rare loss-of-function and other protein-coding variants that are present in Chinese populations, and (3) consistent performance of photolithographic manufacturing processes across array designs and batches of arrays manufactured over an extended time period.^17^

Using the UK Biobank probe list as the starting point for the CKB array design, this was then modified and extended, informed by allele frequency and sequence data for more than 12,000 East Asians that were available to us in 2013 (Data S1; Figure S1). In brief, we (1) removed variants identified as absent or at low frequency in East Asians; (2) added specific variants confirmed as being present in East Asians, including loss-of-function, missense, and eQTL variants; (3) constructed an East Asian-specific genome-wide grid that maximized imputation of both common (>5%) and low-frequency (1%–5%) variants; and (4) included multiple copies of a series of degenerate probes for detection and classification of circulating HBV viral DNA. The resulting array design comprised 781,937 probe sets assaying 700,701 variants, of which 354,399 were also present in the UK Biobank array (Figure S2; Table S2). The design included duplicate probe sets, one for each strand, for 81,236 variants that did not have a validated assay on the Axiom platform.

**Figure 2:**
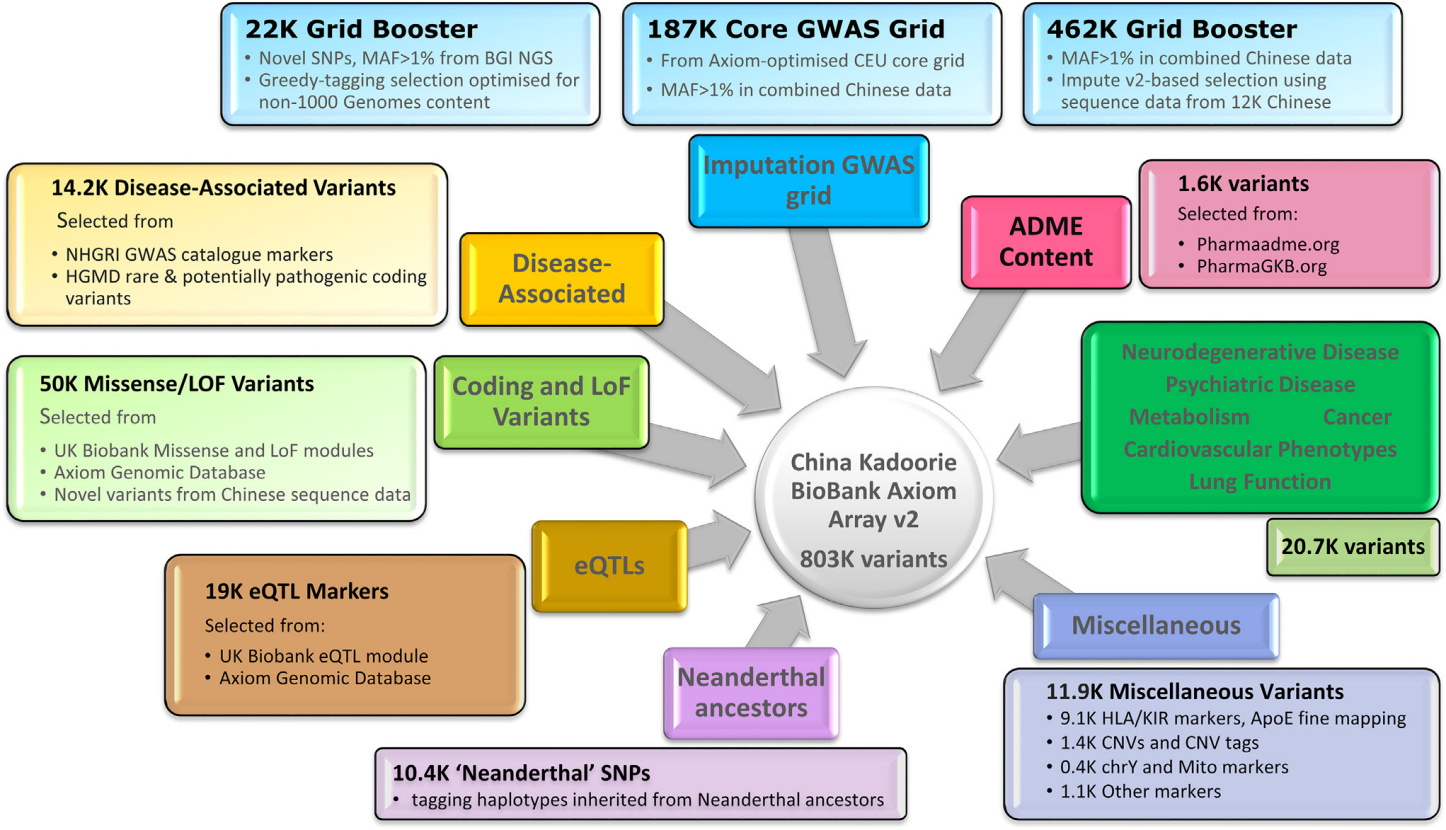
Design of the CKB Axiom genotyping array The figure illustrates the different categories of content on the revised CKB array. Numbers indicate the approximate counts of variants in each category. Some variants fall into more than one category. See also Figures S1–S3, Tables S2 and S3, and Data S1 and S2.

The initial array design was revised on the basis of genotyping data from the first 100 plates (8,995 samples after quality control [QC]) (Data S2; Figure S3). Failed, poor-quality, or otherwise uninformative monomorphic probe sets were removed, along with the poorer-performing probes of each pair of duplicate probe sets. Further specific content of interest (including further HBV probes and tags for variants that failed QC) were added to the design. Finally, variants were added to improve or restore genome-wide imputation coverage. Figure 2 summarizes the content of the final updated array, comprising 804,496 probe sets assaying 803,030 variants, of which 340,562 are present in the UK Biobank array (Table S3).

## Genotyping and QC

Genotyping and QC of a total of 105,408 CKB DNA samples are summarized in Table 1. The initial CKB array design was used to genotype 33,408 samples that had been selected for nested case-control studies of cardiovascular disease and COPD. On the basis of disease follow-up to January 1, 2014 (see below), this initial genotyping included all incident cases of intracerebral hemorrhage (ICH 5,020), subarachnoid hemorrhage (SAH; 455), and fatal IHD (753); randomly selected incident cases of ischemic stroke (IS; 5,662), MI (1,008), and COPD (5,376); participants with no cardiovascular events (n = 10,038) at time of selection, matched to ICH cases for sex, age, and region; and 4,766 randomly selected individuals who had attended the second resurvey.

**Table 1:** Genotyping sample selection and quality control

	Array version 1	Array version 2	Total
Total samples genotyped^a^	33,408	72,000	105,408

Sample ascertainment			

ICH	5,020	602	5,622
SAH	455	46	501
IS	5,662	–	5,662
MI	1,008	1,028	2,036
Fatal IHD	753	163	936
COPD hospitalization	5,358	–	5,358
ICH-matched controls	10,038	–	10,038
Random selection	4,766^b^	69,378^c^	74,144
Intentional duplicates	347	766	1,113
Unintentional duplicates	1	36	37
Total unique samples	33,060	71,198	104,277

QC exclusions			

Failed initial QC	524	2,184	2,708
Call rate <95%	2	0	2
Excess heterozygosity^d^	89	253	342
Excess homozygosity^e^	3	0	3
Sex mismatch	47	121	168
Other linkage error	2	33	35
XY aneuploidy^f^	91	173	264
Ancestry outlier	3	1	4
Consent missing/withdrawn	–	31	31
Samples in current data release	32,300	68,406	100,706

Number of samples genotyped on each genotyping array, showing reasons for selection for genotyping and for quality control exclusion.

a Excluding repeats of plate failures.

b Random selection of samples from participants attending the second resurvey.

c Selected as complete boxes of DNA samples, prioritizing boxes with large numbers of samples from participants eligible for the second resurvey.

d More than 3 SDs greater than mean heterozygosity for participants recruited in the same region.

e More than 3 SDs less than mean heterozygosity for participants recruited in the same region and with total runs of homozygosity <2 SDs greater than the mean.

f Identified as XXY, XY with non-negligible chrX heterozygosity, XXX, X0, or mosaic X0. Some samples failed QC on the basis of >1 criterion.

The updated array design was then used to genotype a further 72,000 samples, including all available additional cases of ICH (602), SAH (46), MI (1,028), and fatal IHD (163) that had not previously been genotyped. The remaining genotyped samples came from boxes of DNA samples that were either randomly selected or selected as containing samples derived from the assessment centers used for the second resurvey, in either case being largely representative of the overall CKB cohort. Duplicate samples were present on each pair of consecutive plates to support sample and plate tracking and for other QC purposes.

Genotyping and QC followed the Affymetrix best practice workflow^18^ with additional checks for probe sets that displayed substantial between-plate or between-batch differences in allele frequency or call rate. After QC and removal of duplicate probe sets, 76.1% and 85.5% of probe sets remained for the initial and revised arrays, respectively (Table S4); 3.4% of samples failed QC (summarized in Table 1), which were mostly initial QC failures, likely reflecting low quality or low concentration DNA samples; only 0.16% of samples were excluded because of sex mismatch, reflecting the CKB’s stringent sample tracking procedures.^19^ All pairs of duplicate samples showed high concordance of non-missing genotypes, overall concordance being 99.88% and 99.87% for the first and second array designs respectively (with 0.67% and 0.64% calls missing in one or both of a pair). Where probe sets on the revised CKB array were present in the UK Biobank array, 192 samples genotyped on both arrays yielded concordance of 99.80% (0.46% missing).

**Figure 3:**
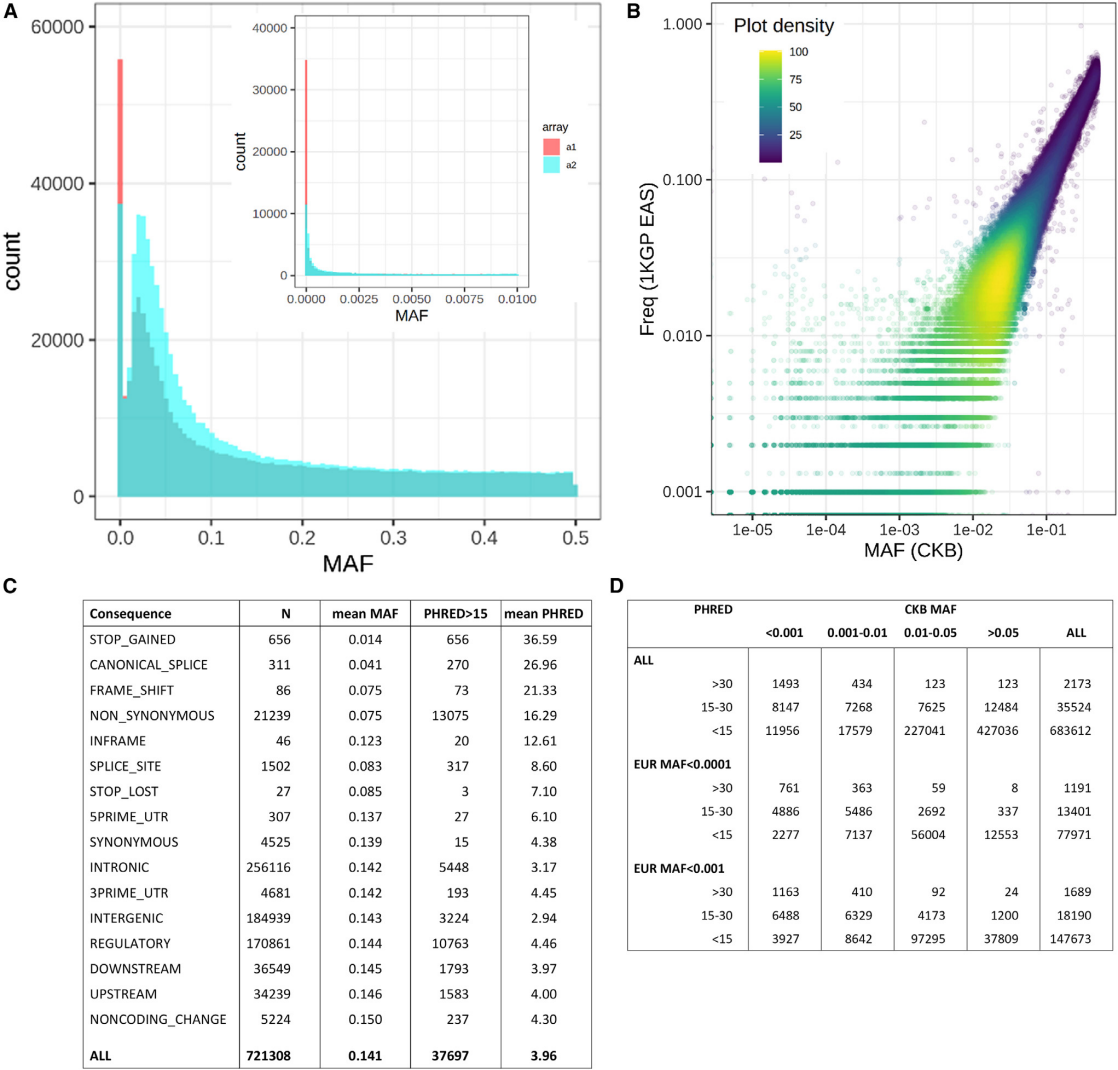
Allele frequency and functional annotation of genotyped variants (A) Allele frequency distribution in unrelated CKB participants of variants passing QC on the two versions of the CKB genotyping array. (B) Comparison of CKB allele frequency of quality-controlled variants on array version 2 with the corresponding allele in the East Asian subset of the 1000 Genomes Phase 3 reference. (C) Frequency and characteristics of different classes of quality-controlled variants on array version 2, according to Combined Annotation Dependent Depletion (CADD version 1.6).^21^^,^^22^ (D) Allele frequency distribution in CKB and European populations of quality-controlled variants on array version 2, for variants with different levels of predicted functional impact according to CADD. See also Figure S4.

The allele frequency distribution for the two datasets reflected the design characteristics of the arrays (Figure 3A). There were many more monomorphic or very low frequency (minor allele frequency [MAF] < 0.0001) variants on the original array design (5.7%) than on the revised array (1.6%) on which many such variants had been removed. Conversely, revision of the array design included selection of additional variants to improve imputation of variants with MAF of 0.01–0.05, and correspondingly more variants passed QC in this MAF range. The allele frequencies of variants that passed QC showed strong agreement with the East Asian populations in the 1000 Genomes reference dataset^20^ (Figure 3B), even at lower MAF where estimates in the 1000 Genomes reference were affected by small sample size, and this agreement was consistent across all recruitment regions, although with somewhat greater variation at lower MAF (Figure S4).

These allele frequency data provide some insight into the potential utility of variants included on the CKB array for purposes of investigating the impact of protein loss of function. Variants on the revised array that passed QC were categorized according to their potential functional significance as predicted by Combined Annotation Dependent Depletion (CADD version 1.6)^21^^,^^22^; this identified 7 classes of variant annotation representing 23,867 variants that had both substantially lower mean MAFs and higher mean Phred values than the other classes, indicating strong enrichment for deleterious variants (Figure 3C). Overall, 37,697 variants had Phred values > 15, corresponding to the top 3% most damaging variants genome-wide, with a high likelihood of pathogenicity.^23^ Of these variants, more than half (20,355 [54%]) had MAFs >0.01 in the CKB (28,057 [74%] for CKB MAF > 0.001), of which 5,489 (27%) are virtually absent from European populations (Figure 3D). These will provide opportunities not available in European cohorts for genetic investigations of the importance of the affected genes for disease and disease risk, such as those already conducted for *PLA2G7* and *CETP*.^5^^,^^6^

Initially, imputation was performed separately for each array dataset, but the revised array provided only a modest improvement in imputation quality, despite the substantially larger number of informative variants passing QC (Table S4). Therefore, to minimize batch and array effects, we derived a single imputation dataset, using only those variants passing QC in all batches on both array versions (although variants excluded for imputation remain available for analysis in the final dataset). We achieved high-confidence imputation for the large majority of common and low-frequency variants present in the EAS populations of the 1000 Genomes Phase 3 reference (Tables S4 and S5; Figure S5): mean info scores were 0.950 for variants with MAFs > 0.05, 0.849 for MAFs of 0.01–0.05, and 0.695 for MAFs of 0.005–0.01. Imputation was typically poorer for rare variants with MAF <0.005, which are excluded from many analyses.

## Relatedness

The community-based recruitment of CKB participants resulted in family groups attending together, so that many individuals had close relatives also recruited into the study (Tables S6 and S7). Among genotyped participants, 31.9% had an also genotyped second-degree or closer relative (23.6% having at least one first-degree relative), with more relatedness in rural than in urban regions (39.0% vs. 22.8% with first-/second-degree relatives), with the exception of participants in Suzhou (54.7%); Suzhou recruitment took place in a previously rural district that has only recently become urbanized. Suzhou also had a particularly high proportion of individuals with multiple close relatives (1,561 [20%] with ≥3 genotyped relatives). Further analysis of relatedness in the CKB also identified 32 pairs of twins, 13,875 individuals with at least 1 sibling (comprising 6,325 family groupings of up to 7 siblings) and 6,571 parent-child relationships, including 1,189 trios.

Several regions displayed patterns of relatedness suggestive of historical consanguinity (Figure S6). Histograms of pairwise identity by descent (IBD) displayed not only the expected peaks corresponding to integer numbers of meioses separating relatives (at IBDs of 0.5, 0.25, 0.125, etc.), but also other peaks centered on values corresponding to relationships that arise as a result of consanguineous unions between individuals with recent common ancestors, for instance triple second cousins (expected IBD of 0.09375). Consistent with this, 1,050 participants had heterozygosity >3 SDs below the mean values across all genotyped samples, all but 3 of whom had correspondingly extensive runs of homozygosity (Figure S7).

## CKB population structure

**Figure 4:**
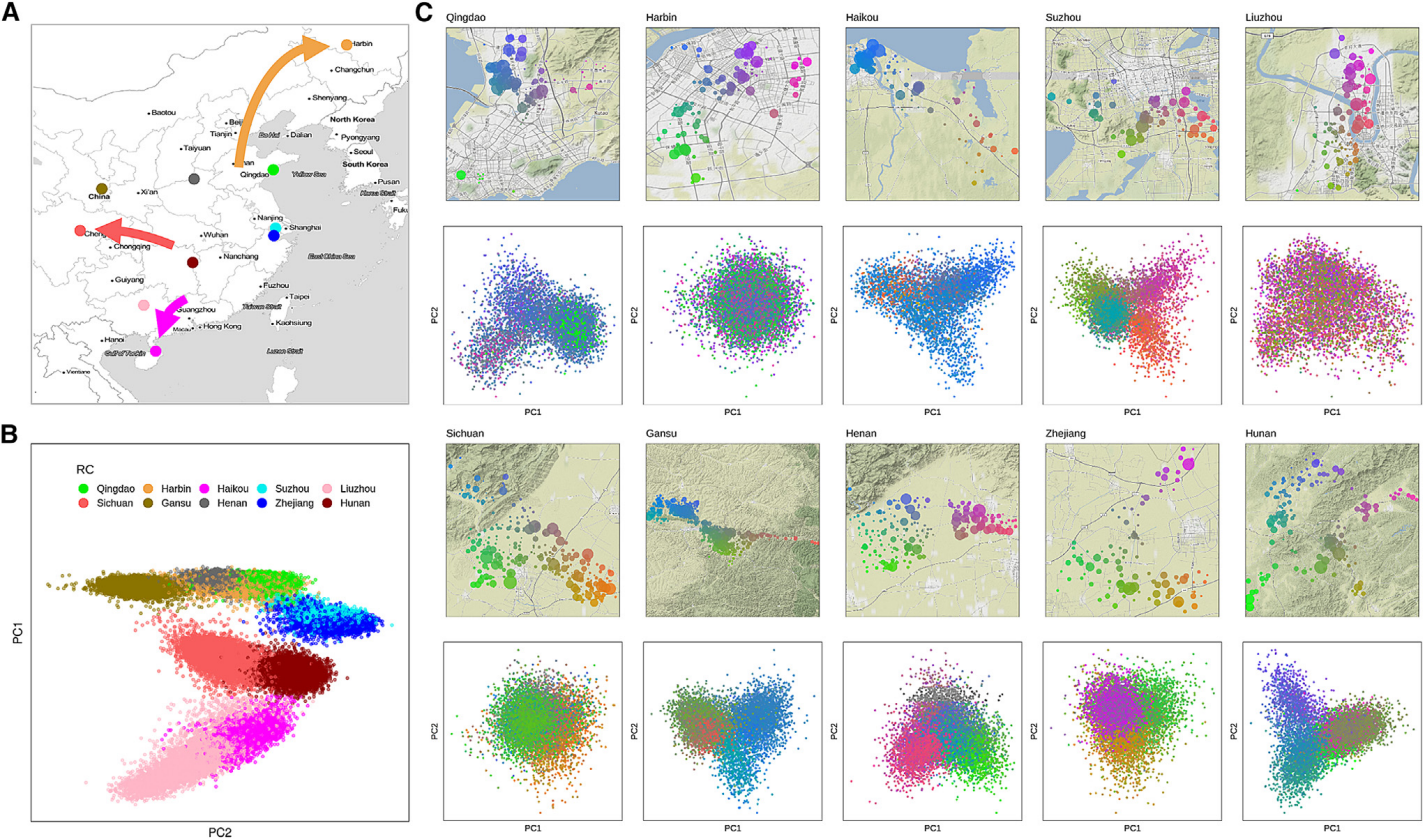
National and local population structure in the CKB (A) Map of China and adjacent countries showing the locations of the ten CKB regional centers (RCs). Arrows denote known major population movements in recent history that can account for mismatches in the correlation between PCA and geography. (B) Plot of the two leading principal components from PCA of CKB genotypes, with each participant color-coded according to the RC where they were recruited. (C) Local maps are shown for each recruitment region, showing the geolocation of individual assessment centers color-coded according to latitude and longitude; the size of the symbol is proportional to the number of genotyped individuals from that center. Corresponding PCA plots show the first two principal components from PCA of individuals from that region, color-coded according to their recruitment center. Top 2 rows, urban regions; bottom 2 rows, rural regions. See also Figures S9, S10, S12, and S13.

We performed PC analysis (PCA) of 76,719 unrelated CKB participants and identified that the first 11 PCs were informative for CKB population structure, according to the Bayesian information criterion (BIC) for models predicting individuals’ recruitment region (Figure S8). Consistent with findings from many previous studies, individuals formed discrete clusters, whose locations on a plot of the first 2 PCs closely resembled the pattern of longitude and latitude for the regions in which they were recruited (Figures 4A, 4B, and S9). For three regions, however, the positions of their PCA clusters were clearly offset compared with their geographic location. In each case, the apparent discrepancy corresponds to a known major population movement from recent Chinese history: large-scale migration in the 16th and 17th centuries ad from Guangdong to Hainan island; repopulation of the Chengdu Basin in Sichuan in the late 17th and early 18th centuries, a large proportion of migrants coming from Huguang (now Hubei/Hunan); and settlement of largely unpopulated Manchuria in the late 19th and early 20th centuries, with the majority of settlers originating from Shandong province. Thus, the lead PCs reflect the known historical geographic origins of the population in a region, rather than its current physical location.

Although the leading PCs tightly clustered most individuals for each recruitment region, a small proportion (5.7% overall) lay outside the main cluster (>3 SDs from the region mean for PCs 1–11) and thus appeared to have non-local ancestry. Of these, for those with data available from the second resurvey, a high proportion (47.8%) reported that they or at least one parent were born in a different province of China; by comparison, only 12.5% of the remainder reported origins from a province other than that in which they were recruited. The large majority of participants with non-local ancestry was recruited in Liuzhou, among whom a substantial fraction of individuals (25.4%) lay outside the main PCA cluster, mostly reflecting outlier values for PC 1 (corresponding to the major north-south axis); 87.2% of these individuals reported that either they or a parent was born outside Guangxi province.

Local population structure within each region was not readily apparent from the above pan-China PCA but was clearly observed for individuals within each region-specific cluster (excluding those identified as having non-local ancestry). Between 2 and 9 PCs were informative for the latitude and longitude of the assessment center at which an individual was recruited (Figures 4C, S10, and S11). Rural regions (plus previously rural Suzhou) typically displayed substantial structure, reflecting established communities with little population movement; by contrast, there was only limited population structure for most urban regions, with the exception of Liuzhou, for which an appreciable proportion of second resurvey participants reported having non-Han ancestry (17.6% compared with 1.0% across the other 9 regions). Although uninformative for geographical location within Liuzhou, the first 4 PCs from whole-cohort PCA or the first 2 local PCs were informative for Han status (Figure S12); however, there was no clear discrimination between Han and non-Han that might suggest a need to analyze these individuals separately.

The geographical and/or historical relationships between the different CKB populations are reflected in F_st_ analyses of the genetic distance between regions (Figure S13): the four northern regions cluster together, and also with the northern Han 1000 Genomes population (CHB); the four regions situated on or near the Yangtze River cluster together, in two pairs, and also with the southern Han 1000 Genomes population (CHS); and the southern two regions cluster together, with no appreciable distinction in Liuzhou between Han and non-Han identity. The positions of the 1000 Genomes Project East Asian populations in this analysis indicate that the 10 regions in the CKB population are components of a continuum running from north to south with no clear separation from neighboring countries (KHV, from Vietnam) or ethnic populations (Dai Chinese, from near to the borders with Laos and Myanmar).

## Disease outcomes

In common with the other biobanks contributing to the GBMI,^14^ one of the chief strengths of the CKB is the ability to follow up study participants for a wide range of fatal and non-fatal disease outcomes.^2^ In the CKB, disease follow-up is obtained by electronic linkage using participants’ unique national identity numbers to registries for death (with cause of death recorded) and for 4 major diseases (stroke, IHD, cancers, diabetes) and to the national health insurance system, which records all inpatient hospital events. These procedures are complemented by active follow-up through annual checks of local residential records and, if necessary, in-person visits by local staff to check key data including vital status and to identify hospitalized episodes in a small proportion of the CKB (currently approximately 2%) who have not joined the health insurance scheme.^24^ Data from these multiple sources, including parsing of free-text Chinese language disease descriptions and matching to a clinician-curated disease description library, are integrated and standardized into International Classification of Diseases, 10th Revision (ICD-10), coded incident disease events. By January 1, 2019, >1.2 million incident events and 49,428 deaths (including causes of death) had been recorded for the whole of the CKB, covering >5,000 separate disease types (defined according to three-character ICD-10 code), with only 5,302 (1.0%) of participants being lost to follow-up.

Table 2 shows the number of individuals with selected incident events as recorded through disease follow-up, in all CKB participants and in the genotyped subset. Additional prevalent cases are available through medical questionnaire data, and on-site measurements at baseline (e.g., type 2 diabetes and COPD, through blood glucose assays and spirometry). Reflecting the strategy for selecting the genotyped samples, there was enrichment for cardiovascular diseases, including IS (ICD-10 code I63), intracerebral hemorrhage (ICD-10 code I61), and MI (I21), for COPD (ICD-10 codes J41–J44 and J47), and for all-cause mortality. By contrast, other diseases unrelated to the ascertained case types were present in proportion with the number of genotyped samples.

**Table 2:** Death and disease events in the CKB

Disease	Definition, ICD-10 codes	All participants	Genotyped
Tuberculosis	A15–A19, J65, K23.0, K67.3, M01.1, M49.0, M90.0, N33.0, N74.0, N74.1	3,053	768
Lung cancer	C33–C34	6,574	1,552
Liver cancer	C22	3,256	665
Stomach cancer	C16	3,771	756
Diabetes	E10–E14	32,748	7,310
MI	I21	7,984	3,386
ICH	I61	11,638	6,663
IS	I63	50,675	14,302
Heart failure	I50	5,160	1,467
Pneumonia	J12–J18	30,339	7,160
Asthma	J45–J46	2,846	880
COPD	J41–J44	20,391	7,971
Liver cirrhosis	K70, K74	2,785	552
Chronic kidney disease	N02–N03, N07, N11, N18	2,879	678
Self-harm	T39.0, T39.1, T39.3, T40, T42, T43, T51, T52, T60, X60–X84, Y87.0, Z91.5	654	114
All hospitalized events	A00–Z99	286,167	60,558
All deaths	A00–Z99, underlying cause	49,428	16,101

Numbers of CKB participants among the whole cohort and the genotyped subset who underwent selected death and disease events during follow-up prior to January 1, 2018. See also Table S8.

The wide range of disease outcomes recorded during follow-up is illustrated by the 224 different 3-character ICD-10 codes that have at least 100 incident events recorded in genotyped participants (Table S8). Although limited, this number of cases is sufficient to permit analysis using software packages such as SAIGE,^25^ and subsequent contributions to multi-cohort meta-analyses. Work is ongoing to convert the ICD-10 data into Phecodes,^26^ to aid harmonization of disease outcomes between CKB and other biobanks and to facilitate phenome-wide association analyses.

## Analytical approach and GWAS

The historical consanguinity and extensive relatedness in the CKB have been exploited in analysis of the impact of inbreeding on reproductive success^27^ and for within-sibship GWASs to derive estimates of direct genetic effects unaffected by genetic nurture.^28^ However, for the majority of studies, the population structure of the CKB cohort together with the strategy for selection of samples for genotyping require thoughtful analytical approaches. The substantial relatedness within the CKB, as in many other population-based cohorts, means that exclusion of individuals to avoid inclusion of pairs of close relatives (typically kinship > 0.05, corresponding to third-degree relatives, e.g., first cousins) would result in a substantial reduction in sample size, with some recruitment regions being disproportionately affected (Tables S6, S7, and S9). Therefore, we typically use well-established software packages such as BOLT-LMM^29^ and SAIGE^25^ that implement linear mixed models to account for both relatedness and population stratification, thereby permitting inclusion of related individuals.

It is unclear, however, that current software packages fully account for all aspects of population structure in the CKB, with its recruitment in 10 discrete regions each with their own distinct genetic characteristics, varying environments, cultures, demographics, and incidence rates of major disease outcomes. For some diseases, this may be not only because they vary in prevalence but also because of varying access to healthcare (e.g., in rural compared with urban regions), so that patterns of severity in reported cases may also vary between regions. Therefore, although it is frequently appropriate to conduct analyses across the full genotyped dataset with adjustment for recruitment center (as a 10-level categorical covariate), wherever possible we supplement this with region-stratified analyses (excluding the 5.7% of individuals with non-local ancestry) and meta-analysis, to ensure that association signals are not due to unresolved population stratification or subject to other biases (e.g., arising from heterogeneity between regions).

A second consideration is that the selection for genotyping of nested case-control samples has resulted in substantial over-representation of participants with hospitalization for cardiovascular disease or COPD; although the additional cases were selected on the basis of incident events (i.e., after recruitment), the baseline characteristics of these individuals nevertheless differ from the overall population (e.g., cardiovascular disease events are positively associated with blood pressure, adiposity, blood lipids, smoking, and alcohol consumption). Their inclusion potentially introduces biases or confounding into analyses using the complete genotyping dataset, and we have therefore developed approaches that seek to minimize or eliminate these biases. Where traits are available only in non-random subsets of individuals (e.g., clinical biochemistry measures), we either exclude ascertained cases entirely, include case ascertainment as a covariate, or conduct analyses stratified by ascertainment; note that this is not required for measurements taken at the second resurvey, which was representative of surviving CKB participants. For quantitative traits available for all participants, such as blood pressure or reproductive traits, we perform all adjustments for covariates and data transformations in the full CKB cohort, prior to genetic analyses, so that these adjustments are not distorted by the non-random nature of those genotyped; this is typically performed as a single regression including region as covariates, but we also check the impact of instead performing such adjustments in each region separately. This is the approach used both for contributions to large multi-ancestry meta-analyses^13^^,^^30^ and for several CKB-specific GWASs.^31^^,^^32^

For the analysis of dichotomous variables, to overcome the potential biases due to case enrichment, we have constructed a subset of 77,176 individuals representative of the full CKB cohort in which over-representation of ascertained disease cases was eliminated (STAR Methods; Tables S1 and S9). Analyses of disease outcomes and other binary phenotypes, including contributions to the first round of GBMI studies,^14^^,^^33^^,^^34^^,^^35^ have typically used this population-representative subset supplemented with additional cases from the remainder of the dataset. For these analyses we use SAIGE software,^25^ which is designed to account for imbalances in numbers of cases and controls. This approach is combined with region stratification and meta-analysis for diseases with large numbers of cases, so that separate analyses in each region are possible and do not lead to exclusion from analysis of large numbers of variants due to low minor allele count (MAC; see STAR Methods). It should be noted that use of the population subset does not result in a noticeable loss of power, despite the exclusion of ∼25% of samples, as there is invariably a large excess of controls even for more common diseases. It also provides a reduced dataset, unaffected by disease ascertainment biases, which can be used for sensitivity analyses of studies of other traits that use the full genotyped dataset.

**Figure 5:**
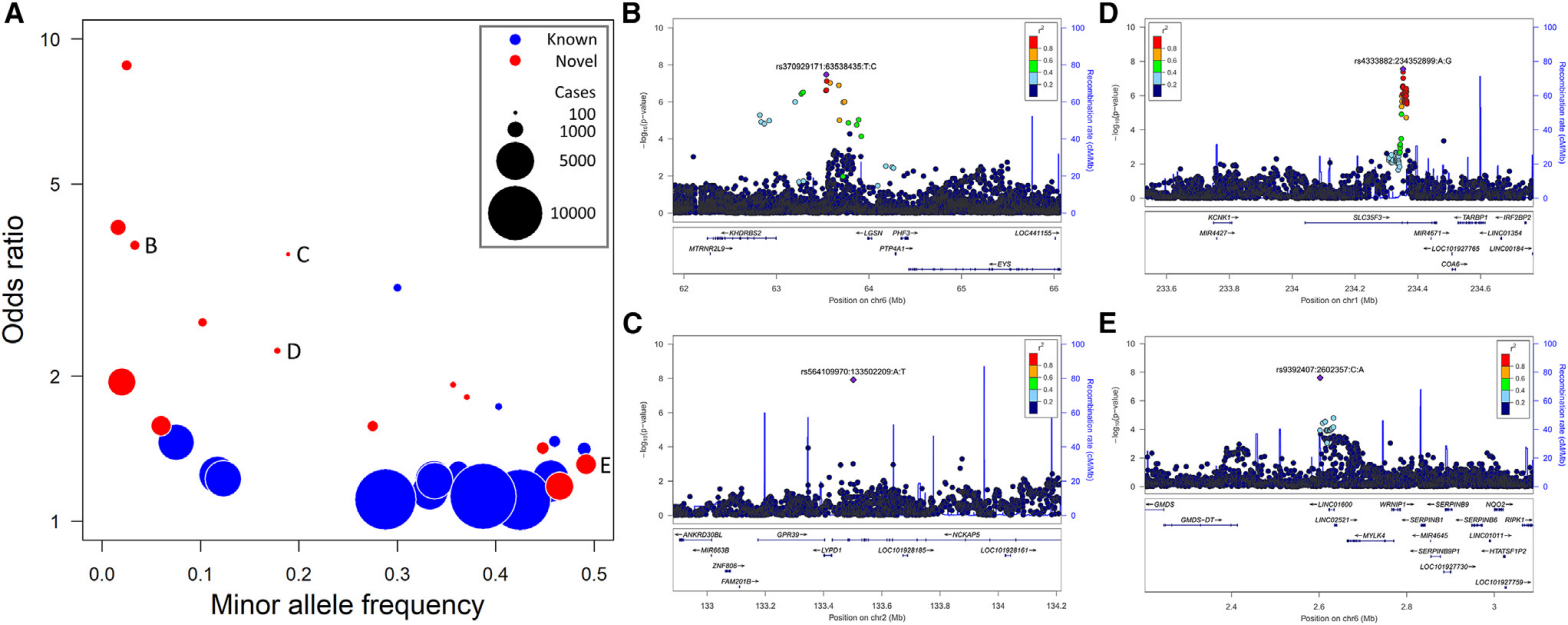
Genome-wide significant associations from GWASs of ICD-10-coded disease events (A) Summary of the minor allele frequency and the effect size for the risk allele, for all associations with ICD-10-coded outcomes reaching genome-wide significance (5 × 10^−8^). Symbols are colored according to whether the association has previously been reported, and are sized in proportion to the number of cases in the corresponding GWAS. (B–E) Labels denote newly identified associations with (B) H40 (glaucoma), (C) H43 (disorders of vitreous body), (D) K60 (fissure and fistula of anal and rectal regions), and (E) K81 (cholecystitis), illustrated in the corresponding regional association plots, for which there are previously reported associations with related phenotypes or diseases at the same locus. Further plots for these and all other ICD-10 GWASs are available on the CKB PheWeb browser at pheweb.ckbiobank.org. See also Table S10.

Using this approach, we have conducted GWASs of the ICD-10 codes with at least 100 genotyped cases, yielding 35 associations at genome-wide significance (5 × 10^−8^) (Figure 5A; Table S10). The majority of these replicated known association signals for a range of diseases (type 2 diabetes, hypertension, atrial fibrillation, cerebral infarction, gout, liver cirrhosis, liver cancer, lung cancer), but we also identified 14 potentially novel disease-associated loci. Although none of the latter associations survived a strict Bonferroni adjustment to take account of the multiple GWASs, and some were based on a small number of cases, several were nevertheless at loci associated with closely related diseases or phenotypes, suggesting that these reflect robust associations: an association with ICD-10 H40 (glaucoma) near *EYS*, at which there is also an association with retinitis pigmentosa,^36^^,^^37^ a known risk factor for primary angle-closure glaucoma^38^ (Figure 5B); an association with H43 (disorders of vitreous body) at *NCKAP5*, close to reported associations with optic disc size^39^ and primary open-angle glaucoma^40^ (Figure 5C); an association with K81 (cholecystitis) at *MYLK4*, at which there is an association with serum alkaline phosphatase,^37^ an established marker of bile duct stones and acute cholecystitis^41^ (Figure 5D); and a variant in *SLC35F3* (rs4333882) associated with K60 (fissure and fistula of anal and rectal regions) and also with one of the causes of fistulas, diverticular disease^42^ (Figure 5E). We have made the results and associated plots of all these GWASs available through a PheWeb browser.^43^

## Research contributions

In combination with genotyping and imputation, and the wide range of phenotypes and disease outcomes available for CKB participants, the above analytical approaches have been applied in diverse studies. Initial studies, using directly genotyped variants, were MR-based investigations that emphasized the value of ancestry diversity for genetic analyses. In early examples of “drug target MR,” we found no association of East Asian-specific variants in *PLA2G7* and *CETP* with major cardiovascular disease outcomes or other major diseases, in each case complementing the results of clinical trials that found no major benefit of drug treatments targeting their respective protein products.^5^^,^^6^^,^^44^ We also exploited the high frequency in East Asians of variants influencing alcohol metabolism, and thereby drinking behavior, to investigate the causal relationship between alcohol consumption and deleterious effects on health: we showed a clear link between alcohol and risk for stroke and, for the first time, we robustly refuted previous reports from observational studies of apparent protective effects of moderate drinking.^45^ Other early genetic studies in the CKB investigated the causal relevance of other disease risk factors, providing evidence that vitamin D deficiency increases risk for diabetes and cardiovascular disease,^46^^,^^47^ that diabetes is itself causally associated with increased risk for cardiovascular disease,^48^ and that, although lowering of low-density lipoprotein (LDL) cholesterol decreases risk for IS, it also increases risk for hemorrhagic stroke.^49^^,^^50^

Since becoming available, the genome-wide imputed data have enabled a wider range of studies, including MR investigation of further drug targets^7^^,^^8^ and of diverse disease risk factors such as bone mineral density,^51^ gallstone disease,^52^ height,^53^ blood pressure,^32^^,^^54^ and resting heart rate.^55^^,^^56^ We have contributed to replication of novel association signals from large GWASs of blood pressure,^57^ menopause,^12^ and early-onset stroke,^58^ and have evaluated the performance of polygenic scores in predicting disease risk for lung function,^9^^,^^59^ lung cancer,^60^ fracture,^61^ and breast cancer.^62^ A comprehensive set of GWASs for major diseases and disease risk factors are in progress, including multiple adiposity and blood pressure traits.^31^^,^^32^ We also recently published the first large GWAS of lung function in an East Asian population,^63^ identifying 48 independent associations of which 18 were novel, once again emphasizing the value of expanding ancestry diversity in genetic studies.

In addition to these analyses conducted primarily within the CKB, we have contributed to many genome-wide association studies in collaboration with major consortia, including trans-ancestry studies of intracranial aneurysm,^10^ recurrent miscarriage,^11^ blood lipids,^64^ fingerprint patterns,^65^ diabetes,^66^ and height.^13^ In particular, CKB has made important contributions to the growing number of studies focused specifically on populations of East Asian ancestry, including the largest single contribution to a GWAS of depression in East Asians^67^; and a major contribution to a GWAS of type 2 diabetes, the largest East Asian GWAS to date.^68^ Summary statistics from CKB GWASs have also contributed to the development of methods for genetic association analyses using very low coverage whole-genome sequencing from non-invasive prenatal testing^69^; trans-ancestry colocalization to assess whether two populations share causal variants^70^; and improved genetic discovery in multi-ancestry meta-analyses.^71^

## Limitations of the study

Although also one of its strengths, the population-based nature of CKB recruitment leads to certain limitations. First, voluntary participation in the study, although mitigated by very low loss to follow-up, might lead to selection bias, with those recruited potentially being healthier and with fewer health conditions that would reduce likelihood of study participation. In addition, because of recruitment in specific, not necessarily representative locations, care may sometimes be required in extrapolating results from CKB to the Chinese population overall.

Second, although there is near-complete linkage to any episode of hospitalization subsequent to recruitment, data on medical (and family) history is restricted to the limited details recorded during the baseline questionnaire, with no outpatient or primary care data recorded (not covered by the health insurance system). This, together with the middle- to old-age profile of the cohort, means that some categories of disease (e.g., relating to female reproduction) are under-reported or not captured. Although addressed in part by updates to the resurvey questionnaires, this only applies to 5% of participants. Nevertheless, the prospective nature of the cohort and comprehensive linkage ensures that analyses in the CKB can provide reliable assessment of the contribution of genetic and non-genetic factors to major diseases in China.

## Future prospects

Together, these many analyses have established the high-quality linkage between genotyping, data collected at baseline recruitment and resurveys, and disease follow-up. With its breadth of phenotypes and disease outcomes, prospective study design, and growing range of diverse omics assays, CKB will continue to make significant contributions to genetic discovery and elucidation of disease etiology and causality. Ongoing work will further enhance the available genetic resources, including DNA methylation arrays for 982 samples^72^; imputation using the Trans-Omics for Precision Medicine (TopMED)^73^ and Westlake Biobank for Chinese (WBBC)^74^ reference panels; and whole-genome sequencing of 10,000 participants with incident IS.^75^ Whole-genome sequencing of the entire 512,000 cohort is planned in the near future through private-public partnership. Together with other notable biobanks across the world, CKB is addressing the recognized need for ancestrally diverse biobanks, and will continue to make strong contributions to the East Asian and trans-ancestry genetic analyses that are beginning to correct the strong Euro-centric bias of the genetic literature.^4^

## STAR★Methods

### Key resources table

**Table:** 

REAGENT or RESOURCE	SOURCE	IDENTIFIER
**Critical commercial assays**		

Golden Gate genotyping	Illumina	https://www.illumina.com/documents/products/technotes/technote_veracode_goldengate_genotyping.pdf
CKB_1/CKB_2 Axiom® arrays	ThermoFisher	Array manifests not publicly available, provided on application

**Deposited data**		

Association results	This paper	https://pheweb.ckbiobank.org
Association results	This paper	GWAS Catalog accessions GCST90246012-GCST90246229, http://ftp.ebi.ac.uk/pub/databases/gwas/summary_statistics/GCST90246001-GCST90247000/
1000 Genomes	1000 Genomes Project Consortium^20^	http://ftp.1000genomes.ebi.ac.uk/vol1/ftp/release/20130502/
GWAS Catalog	Sollis et al.^76^	https://www.ebi.ac.uk/gwas/
OpenGWAS	Elsworth et al.^77^	https://gwas.mrcieu.ac.uk/
ClinVar	Landrum et al.^78^	https://www.ncbi.nlm.nih.gov/clinvar/
Map data	OpenStreetMap	https://www.openstreetmap.org
Map tiles	Stamen	maps.stamen.com
EAS recombination rates	Arnold et al.^79^	http://snipa.helmholtz-muenchen.de/snipa/

**Software and algorithms**		

PLINK	Chang et al.^80^	https://www.cog-genomics.org/plink/
SHAPEIT3	O'Connell et al.^81^	https://jmarchini.org/software/
IMPUTE4	Bycroft et al.^16^	https://jmarchini.org/software/
CADD	Rentzsch et al.^22^	https://cadd.gs.washington.edu/
ALFA	Phan et al.^82^	https://www.ncbi.nlm.nih.gov/snp/docs/gsr/alfa/
FlashPCA	Abraham et al.^83^	https://github.com/gabraham/flashpca
Ggmap	Kahle et al.^84^	https://github.com/dkahle/ggmap
SAIGE	Zhou et al.^25^	https://github.com/weizhouUMICH/SAIGE
LocusZoom	Pruim et al.^85^	https://github.com/statgen/locuszoom-standalone

### Resource availability

#### Lead contact

Further information and requests for resources and reagents should be directed to and will be fulfilled by the lead contact, Robin Walters (robin.walters@ndph.ox.ac.uk).

#### Materials availability

There are restrictions on the availability of extracted DNA due to the Administrative Regulations on Human Genetic Resources of the People’s Republic of China.

### Experimental model and subject details

#### Study permissions

All participants provided written informed consent at each survey visit, allowing access to their medical records and long-term storage of biosamples for future unspecified medical research purposes, without any feedback of results to the individuals concerned. Ethical approval was obtained from the Oxford Tropical Research Ethics Committee, the Ethical Review Committees of the Chinese Center for Disease Control and Prevention, Chinese Academy of Medical Sciences, and the Institutional Review Board (IRB) at Peking University. The Chinese Ministry of Health approved the study at the start in 2004 (including export of plasma samples to Oxford), and also approved electronic linkage to health insurance records in 2011. Raw genotyping data were exported from China to the Oxford CKB International Coordinating Center under Data Export Approvals 2014-13 and 2015-39 from the Office of Chinese Human Genetic Resource Administration.

#### CKB study data

Full details of the CKB study design and methods have been previously reported.^2^ Briefly, 512,726 adults aged 30–79 years were enrolled during 2004–2008 from ten urban and rural areas across China. At local study assessment centers, trained health workers administered a laptop-based questionnaire; undertook physical measurements; and collected a blood sample for long-term storage and onsite blood tests. Three subsequent resurveys of ∼5% randomly selected surviving participants were conducted using similar procedures in 2008, 2013–2014, and 2020–2021. With the exception of genomics data, all CKB survey data were collected and stored using bespoke IT systems and databases tailored to CKB requirements.^86^^,^^87^ Disease follow-up data from death and disease registries and from health insurance records were processed and matched to study identifiers by local staff in each recruitment region, centrally processed and converted into ICD-10-coded events, and integrated into the main study database. The database is regularly processed into research-ready snapshots from which datasets are served to researchers. All results are based on CKB data release version 17.02, incorporating disease follow-up up to 1 January 2019.

### Method details

#### DNA extraction and SNP genotyping

DNA extraction and genotyping was performed at BGI, Shenzhen, China, using KingFisher™ Blood DNA Kit and KingFisher™ Flex 24 Magnetic Particle Processors (Thermo Scientific), yielding 400μL DNA at 220 ng/L mean concentration. Buffy coat sample tubes were barcode scanned, and up to 800μL was manually pipetted into the extraction tube at the positions specified by a bespoke sample-tracking IT system. Extracted DNA was transferred using a Freedom EVO® (Tecan) fluid handling system, up to 200μL to each of two sets of 96-tube racks of 2D-barcoded cryovials (Fluidx, Azenta Life Sciences), and 12μL to a 96-well microtitre plate. DNA concentration and quality was recorded using a NanoDrop Microvolume Spectrophotometer (Thermo Scientific). Tubes were frozen and shipped on dry ice to the CKB sample storage facility in Beijing, for long-term storage at −70C. All sample movements were recorded by the sample-tracking IT system.

The first 95,680 DNA samples (from randomly-selected participants), extracted during 2012–2013, were genotyped using the multiplex Golden Gate® platform (Illumina), for panels of 384 variants which included 3 variants informative for sex within the chromosome XY pseudoautosomal regions. SNP genotyping was performed in 96-well microtitre plates, and used up to 5μL DNA from the microtitre plate produced during DNA extraction. Each genotype plate included positive and negative controls at fixed positions, and 2 pairs of duplicate samples at unique combinations of plate positions. Genotyping was performed for a total of 1,040 plates according to GoldenGate Genotyping Assay Manual Protocols,^88^ with beadchip imaging using an iScan System (Illumina). The 384 SNP panel was revised after genotyping of the first 100 plates (9200 unique samples), and again after the second 100 plates.

Data processing, genotype calling, and quality control was conducted at CTSU, University of Oxford, UK. Genotyping calling was performed in 4 batches using GenomeStudio software, with initial QC based on automated clustering. All negative controls had an SNP call rate of 80% or less (mean = 34%). 15 plates were flagged for inspection due to an initial positive control call rate <95%, but no failures of genotyping were identified; the remaining positive controls had mean call rate of 98.6%. A further 12 plates were flagged for inspection due to 1 or both of 12 pairs of duplicates being among 709 samples excluded with call rate <90%, but again no failures of genotyping were identified.

Following this initial QC, and again after final sample QC, SNPs were reclustered, and within each batch SNPs with GenTrain score <0.7 were inspected manually, and manually reclustered or excluded as appropriate. Across the 3 SNP panels, 30 SNP assays failed genotyping within that panel (either due to gross genotyping failure or call rate <95%), and a further 42 SNP assays failed for a subset of the 4 ‘plexes’. Two SNPs displayed Hardy-Weinberg disequilibrium due to presumed assay interference by nearby SNVs or indels. 15 SNPs displayed potential batch effects, identifying genotype clustering errors that were adjusted manually.

Following SNP QC, an additional 1,518 unique samples (2,217 in total) were excluded on the basis of an SNP call rate <98%. One sample with excess heterozygosity (F-statistic >5 SDs above the mean) was excluded. For 2,063 remaining pairs of duplicate samples, genotyping concordance was between 98.66% and 100% (mean 99.98%), and only 118 samples (0.1%) were identified with mismatches of reported gender and inferred sex based on 3 sex-informative SNPs from chrXY pseudoautosomal regions, confirming good DNA quality and robust linkage to originating study participants. A further 136 samples (0.1%) from blocks with multiple sex-mismatches or with other potential sample linkage errors were also excluded.

#### Genome-wide genotyping

For genotyping using the first version of the CKB array, samples were selected for genotyping as part of nested case-control or case-cohort study designs. Incident cardiovascular disease cases were selected according to available disease follow-up at time of sample selection (August 2014) from amongst those with extracted DNA and no self-reported prior cardiovascular disease history, as follows: (a) all cases of intracerebral haemorrhage (ICH – CD-10: I61, I69.1) where this was the first stroke event, including additional samples selected for prioritised DNA extraction and one case originally incorrectly recorded as an ischaemic stroke (IS); (b) all available cases of subarachnoid haemorrhage (SAH – ICD-10: I60, I69.0) where this was the first stroke event; (c) 5,662 cases of IS (ICD-10: I63, I69.3) occurring prior to 1 January 2014 at age ≤71 years where this was the first stroke event; (d) 1,008 incident cases of myocardial infarction (MI – ICD-10: I21-I23); and (e) all available cases of death with ischaemic heart disease as underlying cause (fatal IHD – ICD-10: I21-I25). Pairs of controls with no cardiovascular disease events or self-report were identified for each ICH case, matched to sex, recruitment region, and year of birth. For respiratory disease, 5,358 participants were selected with at least one event of hospitalisation with chronic obstructive pulmonary disease (COPD – ICD-10: J41-J44); as controls, 4,766 participants were randomly selected from amongst those who attended the second resurvey. For genotyping using the second version of the CKB array, selection was on the basis of complete boxes of DNA samples, prioritising those boxes that contained samples from participants originally recruited in clinics at which the second resurvey was conducted. These samples were supplemented with additional cases of ICH, SAH, MI, and fatal IHD that occurred subsequent to initial sample selection.

Genotyping was performed at BGI, Shenzhen, China. DNA samples selected for genome-wide genotyping were retrieved from storage at −70C, either as complete boxes of 96 samples or (for nested case-control samples) individually selected and transferred to new boxes, and were shipped on dry ice to BGI, Shenzhen. DNA concentration was checked using a NanoDrop Microvolume Spectrophotometer (Thermo Scientific), and a Microlab STAR liquid handling system (Hamilton) was used for transfer of sub-aliquots to new racks of 96 Fluidx cryovials and dilution with TE buffer to 80 ng/μL; the equivalent measured concentration of a subset of samples measured using Qubit DNA quantification (ThermoFisher) was 50 ng/μL. Diluted DNA was plated onto 96-well microtitre plates, with samples from a minimum of 3 boxes distributed across a single plate (a 1:1 mix of cases and controls for nested case-control samples). Samples with low DNA concentration were plated separately for genotyping with a modified first stage of the protocol, using a larger volume of DNA in place of TE buffer. Samples at position H12 were replaced with a duplicate sample from position D1 on the previous plate, thereby providing checks of genotyping quality and sample tracking. Genotyping was performed with manual target preparation according to Affymetrix protocols with automated plate processing and imaging using CKB_1 and CKB_2 Axiom® arrays and GeneTitan® Instruments.^89^ Raw genotyping data were exported from China to the Oxford CKB International Coordinating Center under Data Export Approvals 2014-13 and 2015-39 from the Office of Chinese Human Genetic Resource Administration.

Genotyping quality control and calling (summarized in Table S4) was performed at CTSU, University of Oxford, UK, according to Affymetrix Best Practice workflow^18^ using the Axiom Analysis Suite (Affymetrix) with default settings. Initial QC was performed on samples genotyped on batches of 50 plates. Genotyping was carried out for a preselected set of ∼20K “high performance” SNPs, using the ‘Sample QC’ option, to give initial quality metrics. These were used to identify samples and plates to be excluded from subsequent steps, on the basis of sample DQC<0.82; sample QC call rate <97%; or plates with mean call rate for remaining samples <98.5%. Plates with sample pass rate <95% were flagged for inspection, and were excluded if there was evidence of a general failure of genotyping (e.g. large sections of the plate have failed), or if sample call rate was systematically low relative to sample DQC (rather than having a large number of failing samples due to e.g. a group of samples with poor-quality DNA). Some plate failures identified array manufacturing defects; genotyping of these plates was repeated using a new array.

#### Variant QC

Samples passing initial QC were processed and co-clustered, again in batches of 50 plates, to derive genotypes and further quality metrics. Within each batch, probesets were “failed” if they were classified as “OTV” (off target variation), “CallRateBelowThreshold” (using the default threshold 95%), or “Other”, and genotypes for non-failed probesets; all further QC was performed using PLINK v1.9 and/or v2.0.^80^ Within each batch, probesets were assessed for the presence of plate effects: logistic regressions were conducted to test each individual plate within a batch for significant deviations in genotype calling: each plate in turn was treated as “case” status with all other plates in the batch as controls, with recruitment center as covariate; probesets were failed according to criteria determined empirically through manual review of cluster plots to identify clustering failures – any plate effect with p < 10^−10^, >3 instances of plate effect p < 10^−4^, or any plate effect p < 10^−8^ and clustering metrics FLD<8, HetSO<0.68, and HomRO<3.7; in addition, for probesets with any plate effect P < 2 × 10^−5^, cluster plots were manually reviewed, and appreciable clustering failures (e.g. poor cluster separation) were “failed”.

Probesets passing this initial QC were combined into a single dataset, and a preliminary round of sample QC was performed (see below). A set of autosomal probesets pruned for linkage disequilibrium (LD; PLINK option --indep-pairwise 50 5 0.1) was then used to identify an unrelated subset of samples (PLINK --rel-cutoff 0.025). These were used to test for significant deviations in genotype calling between batches: logistic regressions were performed treating each batch in turn as “case” status with all other batches as controls, again with recruitment center as covariate; probesets were failed entirely, across *all* batches, again according to criteria determined empirically through manual review of cluster plots to identify clustering failures: probesets with any batch effect with p < 10^−10^, >2 or >7 with p < 10^−4^, for array versions 1 and 2 respectively. Clustering was manually checked for remaining probesets with a batch effect with p < 10^−3^ and were scored as either “Pass”, “Batch Fail” (fail in one batch only), or “Fail”. Probesets failing in >10% of batches (i.e. any batch for array version 1, >1 batch for version 2), or with call rates <98% (in passed batches) were excluded entirely from the dataset for that array version.

Probesets were then tested for deviation from Hardy-Weinberg equilibrium (HWE): tests were performed in each recruitment region separately (PLINK --hardy midp) using unrelated individuals (women only for chrX variants), and probesets with an HWE p < 10^−6^ (10 degree of freedom sum-of-Chi-squared test) were excluded. In addition, variants with a minor allele frequency (MAF) > 0.2 different from that in the 3 Chinese populations from the 1000 Genome Project Phase 3 ref. ^20^ were excluded, and one pair of duplicate probesets assaying the same variant (that with the lower call rate) was removed.

Overall performance of the revised array was tested using 192 samples (152 Chinese, 40 European) from the European Vasculitis Genetics Consortium^90^ genotyped using both CKB_2 and UK Biobank Axiom arrays. For 331,838 probesets passing QC on both arrays, concordance between the two arrays was assessed (PLINK --merge-mode 7): 99.5% of genotypes were non-missing for the data from both arrays, with a concordance of 99.80%. Combined Annotation Dependent Depletion (CADD v1.6)^21^^,^^22^ was used to look up the predicted functional consequences of 721,308 variants passing QC on the CKB_2 array; the corresponding allele frequency in Europeans was according to the dbGAP Allele Frequency Aggregator (ALFA)^82^ v2020-11-14, population SAMN10492695.

#### Sample QC

Primary sample QC was conducted for each array version separately, on the basis of criteria as summarized in Table 1. Based on genotyped variants passing QC as above, samples were excluded which had genotyping call rate <0.95, or high/low heterozygosity determined as follows: sample heterozygosity was assessed for autosomal variants with MAF>0.01 (PLINK –het followed by calculation of heterozygosity as 1–HOM/NMISS), mean and SD was determined for samples from each recruitment region (Note: there was a clear North-South gradient in heterozygosity, with a range of values > 1 SD), and samples with a region-specific *Z* score >+3 were excluded; total runs of homozygosity were determined for each sample (PLINK --homozyg-kb 1000), and 3 samples with a region-specific *Z* score <–3 *and* a *Z* score <2 for total runs of homozygosity were excluded (Figure S7).

Samples from individuals with appreciable non-Chinese ancestry were identified by projecting onto principal components derived from 2,504 individuals from 26 populations (5 ancestries) from 1000 Genomes Project Phase 3^20^ using an LD-pruned set of 104,866 variants with MAF >0.01, passing QC for both CKB array versions, and excluding major regions of long-range LD^91^ (PLINK --pca --within --pca-clusters) (Figure S14). A total of 4 individuals were excluded who had a value > 10 SDs from the CKB-wide mean for at least one of the first 10 PCs.

Initial checks of computed sex with that reported in participant data (PLINK --check-sex) identified multiple clusters of sex mismatches, indicating systematic linkage errors. All such clusters of mismatches were tracked back through all steps of sample handling, and the majority could be unambiguously traced to specific sample-handling errors (e.g. 180° rotation of boxes of DNA samples), such that correcting such sample-linkage errors removed all instances of sex mismatch in a cluster without leading to new ones. For clusters that remained uncorrected, all samples in the affected block of samples, irrespective of sex mismatch, were marked for exclusion from the dataset. Other individual sex-mismatched samples were also excluded.

For more detailed checks for sex mismatch, the chrY/chrX probe intensity ratio (parameter cn-probe-chrXY-ratio_gender_ratio output to file AxiomGT1.report.txt during genotyping) was plotted against the chromosome X heterozygosity F-statistics (from PLINK --check-sex), grouping genetically male and female samples into distinct clusters and clearly identifying sex mismatches (Figure S15A). In addition, groups of samples were observed representing potential chromosome XY aneuploidies, including Klinefelter Syndrome and non-Klinefelter XXY, and XO (Turner Syndrome) or XXX, and phenotypic males with appreciable chrX heterozygosity and lower than average chrY/chrX probe ratio. These latter individuals may include individuals with partial chrX translocations, but for most of them the heterozygous markers were distributed along the length of chrX. The samples corresponding to phenotypically male participants were clearly identifiable and were excluded without further investigation. To more robustly identify aneuploid female samples, probe intensity data was extracted using Affymetrix Axiom® CNV Tools software,^92^ and 363 samples were identified whose mean probe intensity (LRR) on chrX was >3 SDs from the mean; for these, probe heterozygosity (BAF) was visualised across chromosome X, enabling identification of 6 Turner, 10 Turner mosaic, and 36 XXX individuals, either with no chrX heterozygosity (Turner) or with BAF values for heterozygous states consistently different from 0.5 (Figure S15B); other aneuploidies were also identified including a partial deletion of the p-arm of chrX and a complex rearrangement with both a partial q-deletion and partial p-duplication. All these individuals were marked for exclusion.

After merging the datasets for the two array versions into a single dataset, genetically identical samples with PI_HAT of ∼1.0 were identified using an LD-pruned and thinned set of 10k autosomal SNPs with MAF>0.05 (PLINK --thin-count 10000 –make-rel). All expected duplicate pairs (including a small number of samples genotyped twice in error) were identified, confirming correct genotyping plate layout and order. All unexpected duplicate pairs were resolved as due either to repeat samples from the ∼2,000 individuals known to have attended the baseline survey twice, or to pairs of individuals whose personal data at recruitment (e.g. recruitment location, date of birth) supported their assignment as putative monozygotic twins. For each pair of duplicate samples, the dataset with the lower call rate was excluded.

#### Imputation

Prior to imputation, additional QC excluded variants at multiallelic sites or with mismatched alleles compared with the 1000 Genomes Project Phase 3 refernece^20^ (October 2014 release); where indicated, strand-flips were performed to match the reference. Imputation was conducted for each array version separately, in each case excluding variants that failed QC in any genotyping batch, and for a combined dataset limited to variants passing QC in all batches on both array versions. For imputation, samples were included which had been excluded from the main dataset on the basis of sex mismatch, linkage errors, or chromosome XY aneuploidy (autosomal imputation only). Phasing was performed for entire chromosomes using biobank-scale SHAPEIT3 r882^81^ with default parameters, except for chromosome X (SHAPEIT2 v2.17^93^ with the -X option, and with pseudoautosomal regions excluded). Imputation used the 1000 Genomes Project Phase 3 reference panel^20^ filtered to exclude variants with MAF = 0 in the 5 East Asian populations, leaving 24,759,908 variants, and was conducted in 20 batches of samples split into 713 chunks (length ranges from 330Kbp to 5264Kbp, mean 3948Kbp) with buffer regions of 500Kbp, using IMPUTE4 v4.r265^16^ for autosomes and IMPUTE2 v2.3.2^94^ for chromosome X. Subsequent to imputation, checks for batch and array-version effects were conducted by testing for association using BOLT-LMM v2.3.1^29^ with individual batches or array version as binary variables; 3867 variants displaying significant batch effects (P < 5 × 10^−8^) were excluded from the imputed dataset. After exclusion of variants with imputation info<0.3, imputed genotypes were available for 21,024,481 variants, of which 8,976,892 had MAF≥0.01 (Tables S4 and S5; Figure S5).

#### Genetic analyses

Unless otherwise specified, genetic analyses were conducted using PLINK v1.9 and PLINK v2.0.^80^ Sets of unrelated samples for variant QC and F_st_ analyses were derived using --rel-cutoff, but for PCA and exclusions for GWAS instead used --king-cutoff 0.05, in each case determined using LD-pruned sets of 122,675 autosomal variants with MAF>0.01 derived using --indep-pairwise 50 5 0.1. On the assumption that near relatives were not present in different recruitment regions, identity-by-descent was determined for all pairs of individuals within each region using --genome gz, from which first and second degree relatives were defined using PI-HAT thresholds of >0.375 and >0.1875, respectively (Tables S6 and S7; Figure S6) and, from the first-degree relatives, parent-child pairs were identified as those with Z0 <0.05 and Z1 >0.5, with the parent identified as the older of the pair. Each pair of siblings was checked for the number of recorded first-degree relatives in the dataset, and the family structures of mismatches were investigated, leading to the identification of one instance of 2 sets of putative three-quarter siblings.

PCA was conducted using FlashPCA v2.1^83^ after LD pruning and exclusion of regions of long-range LD which, if not excluded or otherwise accounted for, can interfere with PCA potentially leading to erroneous conclusions about population structure, or to erroneous genetic association signals. Initial PCA used an LD-pruned set of SNPs excluding previously-identified regions of long-range LD,^91^ but visualisation of variant weights revealed that multiple PCs were nevertheless affected by disproportionate contributions from particular regions of the genome, likely reflecting further regions of long-range LD present in the Chinese population (Figure S16). Therefore, following an approach similar to that previously used for UK Biobank,^95^ a systematic iterative search was conducted to identify and remove regions of long range LD that influenced PCA in this way, using as a starting point an LD-pruned set of 180,570 autosomal variants with MAF>0.01, call rate>0.99, HWE p > 10^−4^, derived using --indep-pairwise 50 5 0.2, in 76,719 unrelated CKB participants. Leading PCs from PCA were tested for the presence of long range LD regions, pairs of identified regions closer than 1Mbp were merged into single extended regions, variants within those regions were excluded, and the PCA was repeated. This process was continued until no long range LD regions were identified in any of the leading 11 PCs informative for CKB population structure, nor in the 12^th^ (not informative) PC. Long range LD regions were identified using a hidden Markov model: presence within/outside a long range LD region was the hidden state; transition between states was in proportion to EAS recombination rates (downloaded from SNiPA^79^); and emission was the posterior probability of being in a long range LD region given the square of the *Z* score for the variant loadings for that PC. Variants were identified as within a long range LD region if they had a posterior marginal probability >0.5. A total of 223 regions were identified (Table S11) and variants within these regions were excluded so that 171,236 variants and 76,719 unrelated CKB participants were included in the final PCA; PCs for the remaining individuals were derived from the corresponding variant weights.

To identify PCs informative for population structure of the full CKB dataset, models were constructed predicting individuals’ recruitment region in which the top PCs were progressively added to the model, using multinom() from R package ‘nnet’,^96^ and Bayes Information Criterion (BIC) was derived using the R BIC() function, informative PCs being those that reduced BIC when added to the model (Figure S8). These were confirmed by ANOVA tests for non-random association of PCs with region of recruitment; above-trend eigenvalues on a scree plot; and visual examination of plots of the top PCs with colour-coding of region of recruitment (Figure S9). Similarly, PCs informative for local population structure were identified on the basis of BIC for linear models predicting latitude and longitude for the assessment center at which individuals were recruited (Figure S11) or Han status (Figure S12). Maps used in PCA plots were drawn with R package ‘ggmap’^84^ using map tiles by Stamen Design (maps.stamen.com) under CC BY 3.0, using data by OpenStreetMap under Open Data Commons Open Database License.

#### Analysis subsets

Subsets of the full genotyped dataset were derived for different analysis approaches (Table S9). For region-stratified analyses, samples with non-local ancestry were excluded; these were identified as outliers for one or more of the informative PCs for that region, on the basis of a robust Mahalanobis distance (from the R mahalanobis() function) of >3 SDs. For analyses requiring unrelated individuals, these were defined using 122,675 LD-pruned variants with MAF>0.01, as above, but applying PLINK --king-cutoff 0.05 which generally gives a larger set of unrelated individuals than --rel-cutoff.

Construction of a subset of genotyped individuals that was largely representative of the overall CKB cohort was based on the fact that the majority of genotyped samples were not selected individually but as complete boxes of DNA samples. These boxes of DNA were prioritised for genotyping solely according to the number of samples they contained that were from participants recruited at study clinics subsequently used for the second resurvey; these clinics had themselves been selected to be population-representative. The procedures for sample collection and DNA extraction meant that each box of DNA included a mixture of samples from at least two randomly-selected boxes of buffy coat samples. Therefore, samples in boxes of DNA were either from individuals invited to the second resurvey and therefore largely representative of the overall CKB cohort, or were random collections of samples from other recruitment locations.

An initial attempt to construct a cohort-representative subset used samples from those boxes with at least 70% of samples genotyped (irrespective of QC), but this was found to be *depleted* for certain ascertained disease cases; this was due to the early prioritisation of a proportion of ICH, SAH, and fatal IHD cases, which led to the transfer of these samples to different storage locations prior to DNA extraction. Therefore, the CKB-representative subset was instead based on the boxes in which blood samples were originally stored immediately after collection and processing at time of recruitment, and used samples originating from boxes of buffy coat with ≥40% of samples selected for genotyping. This gave a set of 77,176 participants which were representative of the overall CKB cohort, in which over-representation of the ascertained diseases was eliminated.

#### Genome-wide association

GWASs were performed for each 3-character ICD-10 chapter with at least 100 genotyped cases, with non-case members of the population representative set of 77,176 individuals as controls (Tables S1 and S9); for diseases expected to be specific to males or females, analyses were restricted to the corresponding sex. Analyses used SAIGE^25^ version 0.42.1 with array version, sex, age, age,^2^ recruitment region and the first 11 national PCs as covariates, and relatedness defined by the LD-pruned set of 122,675 autosomal variants, and were restricted to variants with MAF>0.01, with additional filtering of variants with an effective MAC<20, according to the formula MAC_eff_ = 2∗MAF∗(imputation info)∗N_eff_, where N_eff_ = 4/(1/N_cases_ + 1/N_controls_). Loci at genome-wide significant variants (P < 5 × 10^−8^) were defined by LD-clumping with --clump-p1 5e-8 --clump-kb 5000 --clump-r2 0.05 --clump-p2 0.05 options. Locus novelty was assessed by checking for previously-reported genome-wide significant associations or pathogenic mutations within locus boundaries, according to GWAS catalog,^76^ OpenGWAS,^77^ and ClinVar.^78^ Regional association plots were generated using LocusZoom v1.4^85^ using 10,000 randomly-selected unrelated CKB participants for the LD reference and recombination rates derived from the 1000 Genomes Project Phase 3 EAS populations^20^ using SniPA.^79^

## Data Availability

• Data from baseline, first and second resurveys, and disease follow-up are available under the CKB Open Access Data Policy to bona fide researchers. Full details of the CKB Data Sharing Policy are available at www.ckbiobank.org. • Sharing of genotyping data is currently constrained by the Administrative Regulations on Human Genetic Resources of the People’s Republic of China. Access to these and certain other data is available through collaboration with CKB researchers. • GWAS summary statistics are available at https://pheweb.ckbiobank.org and have been deposited at GWAS Catalog, and are publicly available as of the date of publication. Accession numbers are listed in the key resources table. • The paper does not report original code.

## Supplemental information

Document S1. Figures S1–S16 and Data S1 and S2

Table S1. Baseline characteristics of the overall CKB population and of resurvey participants and the population-representative genotyped subset, related to Figure 1

Table S2. CKB Axiom_1 array module content, related to Figure 2

Table S3. CKB Axiom_2 array module content, related to Figure 2

Table S4. Genotyping and imputation quality control summary, related to STAR Methods

Table S5. Imputation performance of individual and combined array versions, related to STAR Methods

Table S6. Numbers of genotyped CKB participants having relatives elsewhere in the genotyped dataset, related to STAR Methods

Table S7. Patterns of relatedness within genotyped CKB participants, related to STAR Methods

Table S8. Numbers of cases in the full CKB cohort and the genotyped subset for all 3-character ICD-10 codes with at least 100 cases in genotyped participants, related to Table 2

Table S9. Numbers of participants in analysis datasets after applying exclusions for relatedness and/or non-local ancestry, within the full genotyped dataset and in the cohort-representative subset, related to STAR Methods For diseases occurring exclusively or predominantly in men/women only, case counts are restricted to the corresponding sex.

Table S10. Genome-wide significant associations from GWASs of 3-character ICD-10 coded diseases, related to Figure 5

Table S11. Regions of long-range LD identified from PCA of unrelated CKB participants, related to STAR Methods

Document S2. Article plus supplemental information

## References

[1] Chen Z., Lee L., Chen J., Collins R., Wu F., Guo Y., Linksted P., Peto R. (2005). Cohort profile: the Kadoorie Study of Chronic Disease in China (KSCDC). Int. J. Epidemiol..

[2] Chen Z., Chen J., Collins R., Guo Y., Peto R., Wu F., Li L., China Kadoorie Biobank CKB collaborative group (2011). China Kadoorie Biobank of 0.5 million people: survey methods, baseline characteristics and long-term follow-up. Int. J. Epidemiol..

[3] Hindorff L.A., Bonham V.L., Brody L.C., Ginoza M.E.C., Hutter C.M., Manolio T.A., Green E.D. (2018). Prioritizing diversity in human genomics research. Nat. Rev. Genet..

[4] Martin A.R., Kanai M., Kamatani Y., Okada Y., Neale B.M., Daly M.J. (2019). Clinical use of current polygenic risk scores may exacerbate health disparities. Nat. Genet..

[5] Millwood I.Y., Bennett D.A., Walters R.G., Clarke R., Waterworth D., Johnson T., Chen Y., Yang L., Guo Y., Bian Z. (2016). A phenome-wide association study of a lipoprotein-associated phospholipase A2 loss-of-function variant in 90 000 Chinese adults. Int. J. Epidemiol..

[6] Millwood I.Y., Bennett D.A., Holmes M.V., Boxall R., Guo Y., Bian Z., Yang L., Sansome S., Chen Y., Du H. (2018). Association of CETP Gene Variants With Risk for Vascular and Nonvascular Diseases Among Chinese Adults. JAMA Cardiol..

[7] Sliz E., Kettunen J., Holmes M.V., Williams C.O., Boachie C., Wang Q., Männikkö M., Sebert S., Walters R., Lin K. (2018). Metabolomic Consequences of Genetic Inhibition of PCSK9 Compared With Statin Treatment. Circulation.

[8] Bovijn J., Krebs K., Chen C.Y., Boxall R., Censin J.C., Ferreira T., Pulit S.L., Glastonbury C.A., Laber S., Millwood I.Y. (2020). Evaluating the cardiovascular safety of sclerostin inhibition using evidence from meta-analysis of clinical trials and human genetics. Sci. Transl. Med..

[9] Shrine N., Guyatt A.L., Erzurumluoglu A.M., Jackson V.E., Hobbs B.D., Melbourne C.A., Batini C., Fawcett K.A., Song K., Sakornsakolpat P. (2019). New genetic signals for lung function highlight pathways and chronic obstructive pulmonary disease associations across multiple ancestries. Nat. Genet..

[10] Bakker M.K., van der Spek R.A.A., van Rheenen W., Morel S., Bourcier R., Hostettler I.C., Alg V.S., van Eijk K.R., Koido M., Akiyama M. (2020). Genome-wide association study of intracranial aneurysms identifies 17 risk loci and genetic overlap with clinical risk factors. Nat. Genet..

[11] Laisk T., Soares A.L.G., Ferreira T., Painter J.N., Censin J.C., Laber S., Bacelis J., Chen C.Y., Lepamets M., Lin K. (2020). The genetic architecture of sporadic and multiple consecutive miscarriage. Nat. Commun..

[12] Ruth K.S., Day F.R., Hussain J., Martínez-Marchal A., Aiken C.E., Azad A., Thompson D.J., Knoblochova L., Abe H., Tarry-Adkins J.L. (2021). Genetic insights into biological mechanisms governing human ovarian ageing. Nature.

[13] Yengo L., Vedantam S., Marouli E., Sidorenko J., Bartell E., Sakaue S., Graff M., Eliasen A.U., Jiang Y., Raghavan S. (2022). A saturated map of common genetic variants associated with human height. Nature.

[14] Zhou W., Kanai M., Wu K.-H.H., Rasheed H., Tsuo K., Hirbo J.B., Wang Y., Bhattacharya A., Zhao H., Namba S. (2022). Global Biobank Meta-analysis Initiative: Powering genetic discovery across human disease. Cell Genom..

[15] UK Biobank (2014). UK Biobank Axiom Array Content Summary. https://assets.thermofisher.com/TFS-Assets/LSG/brochures/uk_axiom_biobank_contentsummary_brochure.pdf.

[16] Bycroft C., Freeman C., Petkova D., Band G., Elliott L.T., Sharp K., Motyer A., Vukcevic D., Delaneau O., O’Connell J. (2018). The UK Biobank resource with deep phenotyping and genomic data. Nature.

[17] ThermoFisher Scientific (2019). Axiom Precision Medicine Diversity Research Array. https://assets.thermofisher.com/TFS-Assets/GSD/Reference-Materials/axiom-microarray-pmda-datasheet.pdf.

[18] Affymetrix (2017). Axiom Genotyping Solution Data Analysis Guide. https://assets.thermofisher.com/TFS-Assets/LSG/manuals/axiom_genotyping_solution_analysis_guide.pdf.

[19] Millwood I.Y., Walters R.G., Chen Z. (2020). Population Biobank Studies: A Practical Guide.

[20] Auton A., Brooks L.D., Durbin R.M., Garrison E.P., Kang H.M., Korbel J.O., Marchini J.L., McCarthy S., McVean G.A., Abecasis G.R., 1000 Genomes Project Consortium (2015). A global reference for human genetic variation. Nature.

[21] Rentzsch P., Witten D., Cooper G.M., Shendure J., Kircher M. (2019). CADD: predicting the deleteriousness of variants throughout the human genome. Nucleic Acids Res..

[22] Rentzsch P., Schubach M., Shendure J., Kircher M. (2021). CADD-Splice—improving genome-wide variant effect prediction using deep learning-derived splice scores. Genome Med..

[23] van der Velde K.J., de Boer E.N., van Diemen C.C., Sikkema-Raddatz B., Abbott K.M., Knopperts A., Franke L., Sijmons R.H., de Koning T.J., Wijmenga C. (2017). GAVIN: Gene-Aware Variant INterpretation for medical sequencing. Genome Biol..

[24] Yang L., Chen Z., Chen Z. (2020). Population Biobank Studies: A Practical Guide.

[25] Zhou W., Nielsen J.B., Fritsche L.G., Dey R., Gabrielsen M.E., Wolford B.N., LeFaive J., VandeHaar P., Gagliano S.A., Gifford A. (2018). Efficiently controlling for case-control imbalance and sample relatedness in large-scale genetic association studies. Nat. Genet..

[26] Wu P., Gifford A., Meng X., Li X., Campbell H., Varley T., Zhao J., Carroll R., Bastarache L., Denny J.C. (2019). Mapping ICD-10 and ICD-10-CM Codes to Phecodes: Workflow Development and Initial Evaluation. JMIR Med. Inform..

[27] Clark D.W., Okada Y., Moore K.H.S., Mason D., Pirastu N., Gandin I., Mattsson H., Barnes C.L.K., Lin K., Zhao J.H. (2019). Associations of autozygosity with a broad range of human phenotypes. Nat. Commun..

[28] Howe L.J., Nivard M.G., Morris T.T., Hansen A.F., Rasheed H., Cho Y., Chittoor G., Ahlskog R., Lind P.A., Palviainen T. (2022). Within-sibship genome-wide association analyses decrease bias in estimates of direct genetic effects. Nat. Genet..

[29] Loh P.-R., Tucker G., Bulik-Sullivan B.K., Vilhjálmsson B.J., Finucane H.K., Salem R.M., Chasman D.I., Ridker P.M., Neale B.M., Berger B. (2015). Efficient Bayesian mixed-model analysis increases association power in large cohorts. Nat. Genet..

[30] Shrine N., Izquierdo A.G., Chen J., Packer R., Hall R.J., Guyatt A.L., Batini C., Thompson R.J., Pavuluri C., Malik V. (2023). Multi-ancestry genome-wide association analyses improve resolution of genes and pathways influencing lung function and chronic obstructive pulmonary disease risk. Nat. Genet..

[31] Fairhurst-Hunter Z., Lin K., Millwood I.Y., Pozarickij A., Chen T.-T., Torres J.M., Lun J.-a., Kartsonaki C., Gan W., Mahajan A. (2022). Trans-ancestry meta-analysis improves performance of genetic scores for multiple adiposity-related traits in East Asian populations. medRxiv.

[32] Pozarickij A., Gan W., Lin K., Clarke R., Fairhurst-Hunter Z., Koido M., Kanai M., Okada Y., Kamatani Y., Guo Y. (2023). Causal relevance of different blood pressure traits on risk of cardiovascular diseases: GWAS and Mendelian randomisation in 100,000 Chinese adults. medRxiv.

[33] Tsuo K., Zhou W., Wang Y., Kanai M., Namba S., Gupta R., Majara L., Nkambule L.L., Morisaki T., Okada Y. (2022). Multi-ancestry meta-analysis of asthma identifies novel associations and highlights the value of increased power and diversity. Cell Genom..

[34] Wu K.-H.H., Douville N.J., Konerman M.C., Mathis M.R., Hummel S.L., Wolford B.N., Surakka I., Graham S.E., Joo H., Hirbo J. (2021). Polygenic risk score from a multi-ancestry GWAS uncovers susceptibility of heart failure. medRxiv.

[35] Partanen J.J., Häppölä P., Zhou W., Lehisto A.A., Ainola M., Sutinen E., Allen R.J., Stockwell A.D., Leavy O.C., Oldham J.M. (2022). Leveraging global multi-ancestry meta-analysis in the study of idiopathic pulmonary fibrosis genetics. Cell Genom..

[36] Mbatchou J., Barnard L., Backman J., Marcketta A., Kosmicki J.A., Ziyatdinov A., Benner C., O’Dushlaine C., Barber M., Boutkov B. (2021). Computationally efficient whole-genome regression for quantitative and binary traits. Nat. Genet..

[37] Sakaue S., Kanai M., Tanigawa Y., Karjalainen J., Kurki M., Koshiba S., Narita A., Konuma T., Yamamoto K., Akiyama M. (2021). A cross-population atlas of genetic associations for 220 human phenotypes. Nat. Genet..

[38] Hung M.C., Chen Y.Y. (2022). Association between retinitis pigmentosa and an increased risk of primary angle closure glaucoma: A population-based cohort study. PLoS One.

[39] Han X., Qassim A., An J., Marshall H., Zhou T., Ong J.S., Hassall M.M., Hysi P.G., Foster P.J., Khaw P.T. (2019). Genome-wide association analysis of 95 549 individuals identifies novel loci and genes influencing optic disc morphology. Hum. Mol. Genet..

[40] Osman W., Low S.-K., Takahashi A., Kubo M., Nakamura Y. (2012). A genome-wide association study in the Japanese population confirms 9p21 and 14q23 as susceptibility loci for primary open angle glaucoma. Hum. Mol. Genet..

[41] Zgheib H., Wakil C., Shayya S., Mailhac A., Al-Taki M., El Sayed M., Tamim H. (2019). Utility of liver function tests in acute cholecystitis. Ann. Hepatobiliary. Pancreat. Surg..

[42] Maguire L.H., Handelman S.K., Du X., Chen Y., Pers T.H., Speliotes E.K. (2018). Genome-wide association analyses identify 39 new susceptibility loci for diverticular disease. Nat. Genet..

[43] Gagliano Taliun S.A., VandeHaar P., Boughton A.P., Welch R.P., Taliun D., Schmidt E.M., Zhou W., Nielsen J.B., Willer C.J., Lee S. (2020). Exploring and visualizing large-scale genetic associations by using PheWeb. Nat. Genet..

[44] Millwood I.Y., Bennett D.A., Walters R.G., Clarke R., Waterworth D., Johnson T., Chen Y., Yang L., Guo Y., Bian Z. (2016). Lipoprotein-Associated Phospholipase A2 Loss-of-Function Variant and Risk of Vascular Diseases in 90,000 Chinese Adults. J. Am. Coll. Cardiol..

[45] Millwood I.Y., Walters R.G., Mei X.W., Guo Y., Yang L., Bian Z., Bennett D.A., Chen Y., Dong C., Hu R. (2019). Conventional and genetic evidence on alcohol and vascular disease aetiology: a prospective study of 500 000 men and women in China. Lancet.

[46] Lu L., Bennett D.A., Millwood I.Y., Parish S., McCarthy M.I., Mahajan A., Lin X., Bragg F., Guo Y., Holmes M.V. (2018). Association of vitamin D with risk of type 2 diabetes: A Mendelian randomisation study in European and Chinese adults. PLoS Med..

[47] Huang T., Afzal S., Yu C., Guo Y., Bian Z., Yang L., Millwood I.Y., Walters R.G., Chen Y., Chen N. (2019). Vitamin D and cause-specific vascular disease and mortality: a Mendelian randomisation study involving 99,012 Chinese and 106,911 European adults. BMC Med..

[48] Gan W., Bragg F., Walters R.G., Millwood I.Y., Lin K., Chen Y., Guo Y., Vaucher J., Bian Z., Bennett D. (2019). Genetic Predisposition to Type 2 Diabetes and Risk of Subclinical Atherosclerosis and Cardiovascular Diseases Among 160,000 Chinese Adults. Diabetes.

[49] Sun L., Clarke R., Bennett D., Guo Y., Walters R.G., Hill M., Parish S., Millwood I.Y., Bian Z., Chen Y. (2019). Causal associations of blood lipids with risk of ischemic stroke and intracerebral hemorrhage in Chinese adults. Nat. Med..

[50] Falcone G.J., Kirsch E., Acosta J.N., Noche R.B., Leasure A., Marini S., Chung J., Selim M., Meschia J.F., Brown D.L. (2020). Genetically Elevated LDL Associates with Lower Risk of Intracerebral Hemorrhage. Ann. Neurol..

[51] Gan W., Clarke R.J., Mahajan A., Kulohoma B., Kitajima H., Robertson N.R., Rayner N.W., Walters R.G., Holmes M.V., Chen Z., McCarthy M.I. (2017). Bone mineral density and risk of type 2 diabetes and coronary heart disease: A Mendelian randomization study. Wellcome Open Res..

[52] Pang Y., Lv J., Kartsonaki C., Guo Y., Yu C., Chen Y., Yang L., Bian Z., Millwood I.Y., Walters R.G. (2021). Causal effects of gallstone disease on risk of gastrointestinal cancer in Chinese. Br. J. Cancer.

[53] Linden A.B., Clarke R., Hammami I., Hopewell J.C., Guo Y., Whiteley W.N., Lin K., Turnbull I., Chen Y., Yu C. (2022). Genetic associations of adult height with risk of cardioembolic and other subtypes of ischemic stroke: A mendelian randomization study in multiple ancestries. PLoS Med..

[54] Clarke R., Wright N., Walters R., Gan W., Guo Y., Millwood I.Y., Yang L., Chen Y., Lewington S., Lv J. (2023). Genetically Predicted Differences in Systolic Blood Pressure and Risk of Cardiovascular and Noncardiovascular Diseases: A Mendelian Randomization Study in Chinese Adults. Hypertension.

[55] Wang W., Wang J., Lv J., Yu C., Shao C., Tang Y., Guo Y., Bian Z., Du H., Yang L. (2021). Association of heart rate and diabetes among 0.5 million adults in the China Kadoorie biobank: Results from observational and Mendelian randomization analyses. Nutr. Metab. Cardiovasc. Dis..

[56] Huang T., Wang W., Wang J., Lv J., Yu C., Guo Y., Pei P., Huang N., Yang L., Millwood I.Y. (2022). Conventional and bi-directional genetic evidence on resting heart rate and cardiometabolic traits. J. Clin. Endocrinol. Metab..

[57] Takeuchi F., Akiyama M., Matoba N., Katsuya T., Nakatochi M., Tabara Y., Narita A., Saw W.-Y., Moon S., Spracklen C.N. (2018). Interethnic analyses of blood pressure loci in populations of East Asian and European descent. Nat. Commun..

[58] Jaworek T., Xu H., Gaynor B.J., Cole J.W., Rannikmae K., Stanne T.M., Tomppo L., Abedi V., Amouyel P., Armstrong N.D. (2022). Contribution of Common Genetic Variants to Risk of Early-Onset Ischemic Stroke. Neurology.

[59] Wain L.V., Shrine N., Artigas M.S., Erzurumluoglu A.M., Noyvert B., Bossini-Castillo L., Obeidat M., Henry A.P., Portelli M.A., Hall R.J. (2017). Genome-wide association analyses for lung function and chronic obstructive pulmonary disease identify new loci and potential druggable targets. Nat. Genet..

[60] Dai J., Lv J., Zhu M., Wang Y., Qin N., Ma H., He Y.Q., Zhang R., Tan W., Fan J. (2019). Identification of risk loci and a polygenic risk score for lung cancer: a large-scale prospective cohort study in Chinese populations. Lancet Respir. Med..

[61] Lu T., Forgetta V., Keller-Baruch J., Nethander M., Bennett D., Forest M., Bhatnagar S., Walters R.G., Lin K., Chen Z. (2021). Improved prediction of fracture risk leveraging a genome-wide polygenic risk score. Genome Med..

[62] Ho W.K., Tai M.C., Dennis J., Shu X., Li J., Ho P.J., Millwood I.Y., Lin K., Jee Y.H., Lee S.H. (2022). Polygenic risk scores for prediction of breast cancer risk in Asian populations. Genet. Med..

[63] Zhu Z., Li J., Si J., Ma B., Shi H., Lv J., Cao W., Guo Y., Millwood I.Y., Walters R.G. (2021). A large-scale genome-wide association analysis of lung function in the Chinese population identifies novel loci and highlights shared genetic aetiology with obesity. Eur. Respir. J..

[64] Graham S.E., Clarke S.L., Wu K.-H.H., Kanoni S., Zajac G.J.M., Ramdas S., Surakka I., Ntalla I., Vedantam S., Winkler T.W. (2021). The power of genetic diversity in genome-wide association studies of lipids. Nature.

[65] Li J., Glover J.D., Zhang H., Peng M., Tan J., Mallick C.B., Hou D., Yang Y., Wu S., Liu Y. (2022). Limb development genes underlie variation in human fingerprint patterns. Cell.

[66] Mahajan A., Spracklen C.N., Zhang W., Ng M.C.Y., Petty L.E., Kitajima H., Yu G.Z., Rüeger S., Speidel L., Kim Y.J. (2022). Multi-ancestry genetic study of type 2 diabetes highlights the power of diverse populations for discovery and translation. Nat. Genet..

[67] Giannakopoulou O., Lin K., Meng X., Su M.H., Kuo P.H., Peterson R.E., Awasthi S., Moscati A., Coleman J.R.I., Bass N. (2021). The Genetic Architecture of Depression in Individuals of East Asian Ancestry: A Genome-Wide Association Study. JAMA Psychiatr..

[68] Spracklen C.N., Horikoshi M., Kim Y.J., Lin K., Bragg F., Moon S., Suzuki K., Tam C.H.T., Tabara Y., Kwak S.H. (2020). Identification of type 2 diabetes loci in 433,540 East Asian individuals. Nature.

[69] Liu S., Huang S., Chen F., Zhao L., Yuan Y., Francis S.S., Fang L., Li Z., Lin L., Liu R. (2018). Genomic Analyses from Non-invasive Prenatal Testing Reveal Genetic Associations, Patterns of Viral Infections, and Chinese Population History. Cell.

[70] Kuchenbaecker K., Telkar N., Reiker T., Walters R.G., Lin K., Eriksson A., Gurdasani D., Gilly A., Southam L., Tsafantakis E. (2019). The transferability of lipid loci across African, Asian and European cohorts. Nat. Commun..

[71] Turley P., Martin A.R., Goldman G., Li H., Kanai M., Walters R.K., Jala J.B., Lin K., Millwood I.Y., Carey C.E. (2021). Multi-Ancestry Meta-Analysis yields novel genetic discoveries and ancestry-specific associations. bioRxiv.

[72] Si J., Yang S., Sun D., Yu C., Guo Y., Lin Y., Millwood I.Y., Walters R.G., Yang L., Chen Y. (2021). Epigenome-wide analysis of DNA methylation and coronary heart disease: a nested case-control study. Elife.

[73] Taliun D., Harris D.N., Kessler M.D., Carlson J., Szpiech Z.A., Torres R., Taliun S.A.G., Corvelo A., Gogarten S.M., Kang H.M. (2021). Sequencing of 53,831 diverse genomes from the NHLBI TOPMed Program. Nature.

[74] Zhu X.-W., Liu K.-Q., Wang P.-Y., Liu J.-Q., Chen J.-Y., Xu X.-J., Xu J.-J., Qiu M.-C., Sun Y., Liu C. (2021). Cohort profile: the Westlake BioBank for Chinese (WBBC) pilot project. BMJ Open.

[75] Yu C., Lan X., Tao Y., Guo Y., Sun D., Qian P., Zhou Y., Walters R., Li L., Millwood I. (2022). A High-resolution Haplotype-resolved Reference Panel Constructed from the China Kadoorie Biobank Study. medRxiv.

[76] Sollis E., Mosaku A., Abid A., Buniello A., Cerezo M., Gil L., Groza T., Güneş O., Hall P., Hayhurst J. (2023). The NHGRI-EBI GWAS Catalog: knowledgebase and deposition resource. Nucleic Acids Res..

[77] Elsworth B., Lyon M., Alexander T., Liu Y., Matthews P., Hallett J., Bates P., Palmer T., Haberland V., Smith G.D. (2020). The MRC IEU OpenGWAS data infrastructure. bioRxiv.

[78] Landrum M.J., Lee J.M., Benson M., Brown G.R., Chao C., Chitipiralla S., Gu B., Hart J., Hoffman D., Jang W. (2018). ClinVar: improving access to variant interpretations and supporting evidence. Nucleic Acids Res..

[79] Arnold M., Raffler J., Pfeufer A., Suhre K., Kastenmüller G. (2015). SNiPA: an interactive, genetic variant-centered annotation browser. Bioinformatics.

[80] Chang C.C., Chow C.C., Tellier L.C., Vattikuti S., Purcell S.M., Lee J.J. (2015). Second-generation PLINK: rising to the challenge of larger and richer datasets. GigaScience.

[81] O'Connell J., Sharp K., Shrine N., Wain L., Hall I., Tobin M., Zagury J.-F., Delaneau O., Marchini J. (2016). Haplotype estimation for biobank-scale data sets. Nat. Genet..

[82] Phan L., Jin Y., Zhang H., Qiang W., Shekhtman E., Shao D., Revoe D., Villamarin R., Ivanchenko E., Kimura M. (2020). ALFA: Allele Frequency Aggregator. http://www.ncbi.nlm.nih.gov/snp/docs/gsr/alfa/.

[83] Abraham G., Qiu Y., Inouye M. (2017). FlashPCA2: principal component analysis of Biobank-scale genotype datasets. Bioinformatics.

[84] Kahle D., Wickham H. (2013). ggmap: Spatial Visualization with ggplot2. R J..

[85] Pruim R.J., Welch R.P., Sanna S., Teslovich T.M., Chines P.S., Gliedt T.P., Boehnke M., Abecasis G.R., Willer C.J. (2010). LocusZoom: regional visualization of genome-wide association scan results. Bioinformatics.

[86] Lancaster G., Gilbert S., Yang X., Chen Z. (2020). Population Biobank Studies: A Practical Guide.

[87] Sansome G., Hacker A., Chen Z. (2020). Population Biobank Studies: A Practical Guide.

[88] Illumina (2010). GoldenGate Genotyping Assay Guide. https://support.illumina.com/content/dam/illumina-support/documents/documentation/chemistry_documentation/arraykits/goldengate/GoldenGate_Genotyping_Assay_Guide_15004065_B.pdf.

[89] Affymetrix (2010). Axiom Genotyping Assay. https://www.affymetrix.com/support/downloads/manuals/axiom_assay_user_manual.pdf.

[90] Lyons P.A., Peters J.E., Alberici F., Liley J., Coulson R.M.R., Astle W., Baldini C., Bonatti F., Cid M.C., Elding H. (2019). Genome-wide association study of eosinophilic granulomatosis with polyangiitis reveals genomic loci stratified by ANCA status. Nat. Commun..

[91] Price A.L., Weale M.E., Patterson N., Myers S.R., Need A.C., Shianna K.V., Ge D., Rotter J.I., Torres E., Taylor K.D. (2008). Long-range LD can confound genome scans in admixed populations. Am. J. Hum. Genet..

[92] Affymetrix (2013). Axiom CNV Summary Tool. http://www.affymetrix.com/support/downloads/manuals/axiom_cnv_summary_tool_usermanual.pdf.

[93] Delaneau O., Marchini J., Lunter G., Marchini J.L., Myers S., Gupta-Hinch A., Iqbal Z., Mathieson I., 1000 Genomes Project Consortium, 1000 Genomes Project Consortium (2014). Integrating sequence and array data to create an improved 1000 Genomes Project haplotype reference panel. Nat. Commun..

[94] Howie B.N., Donnelly P., Marchini J. (2009). A Flexible and Accurate Genotype Imputation Method for the Next Generation of Genome-Wide Association Studies. PLoS Genet..

[95] Privé F., Luu K., Blum M.G.B., McGrath J.J., Vilhjálmsson B.J. (2020). Efficient toolkit implementing best practices for principal component analysis of population genetic data. Bioinformatics.

[96] Venables B., Ripley B.D. (2002).

