# Supplementary material for: Genotyping and population characteristics of the China Kadoorie Biobank
Source: Cell Genom. 2023 Jul 20;3(8):100361. doi: 10.1016/j.xgen.2023.100361 (PMC10435379; doi:10.1016/j.xgen.2023.100361)
Supplement: Document S1. Figures S1–S16 and Data S1 and S2 [file mmc1.pdf]

Cell Genomics, Volume *3*

Supplemental information

Genotyping and population characteristics of the China Kadoorie Biobank

Robin G. Walters, Iona Y. Millwood, Kuang Lin, Dan Schmidt Valle, Pandora McDonnell, Alex Hacker, Daniel Avery, Ahmed Edris, Hannah Fry, Na Cai, Warren W. Kretzschmar, M. Azim Ansari, Paul A. Lyons, Rory Collins, Peter Donnelly, Michael Hill, Richard Peto, Hongbing Shen, Xin Jin, Chao Nie, Xun Xu, Yu Guo, Canqing Yu, Jun Lv, Robert J. Clarke, Liming Li, Zhengming Chen, and China Kadoorie Biobank Collaborative Group

Genotyping and population characteristics of the China Kadoorie Biobank

Robin G Walters, Iona Y Millwood, Kuang Lin, Dan Schmidt Valle, Pandora McDonnell, Alex Hacker, Daniel Avery, Ahmed Edris, Hannah Fry, Na Cai, Warren W Kretzschmar, M Azim Ansari, Paul A Lyons, Rory Collins, Peter Donnelly, Michael R Hill, Richard Peto, Hongbing Shen, Xun Xu, Yu Guo, Canqing Yu, Jun Lv, Robert J Clarke, Liming Li, Zhengming Chen, for the China Kadoorie Biobank Collaborative Group

Supplementary Information

Members of the China Kadoorie Biobank Collaborative Group 2

Supplementary Figures 3

Supplementary Data S1: China Kadoorie Biobank Array Design 21

Supplementary Data S2: China Kadoorie Biobank Array Revision 26

Members of the China Kadoorie Biobank Collaborative Group

International Steering Committee: Junshi Chen, Zhengming Chen (PI), Robert Clarke, Rory Collins, Yu Guo, Liming Li (PI), Chen Wang, Jun Lv, Richard Peto, Robin Walters.

International Co-ordinating Centre, Oxford: Daniel Avery, Derrick Bennett, Ruth Boxall, Sushila Burgess, Ka Hung Chan, Yiping Chen, Zhengming Chen, Johnathan Clarke; Robert Clarke, Huaidong Du, Ahmed Edris, Hannah Fry, Simon Gilbert, Mike Hill, Pek Kei Im, Andri Iona, Maria Kakkoura, Christiana Kartsonaki, Hubert Lam, Kuang Lin, Mohsen Mazidi, Iona Millwood, Sam Morris, Qunhua Nie, Alfred Pozarickij, Paul Ryder, Saredo Said, Dan Schmidt, Paul Sherliker, Becky Stevens, Iain Turnbull, Robin Walters, Baihan Wang, Lin Wang, Neil Wright, Ling Yang, Xiaoming Yang, Pang Yao.

National Co-ordinating Centre, Beijing: Xiao Han, Can Hou, Qingmei Xia, Chao Liu, Jun Lv, Pei Pei, Canqing Yu.

Regional Co-ordinating Centres:

Gansu: Gansu Provincial CDC – Caixia Dong, Pengfei Ge, Xiaolan Ren. Maiji CDC – Zhongxiao Li, Enke Mao, Tao Wang, Hui Zhang, Xi Zhang. **Haikou:** Hainan Provincial CDC – Jinyan Chen, Ximin Hu, Xiaohuan Wang. Meilan CDC – Zhendong Guo, Huimei Li, Yilei Li, Min Weng, Shukuan Wu. **Harbin:** Heilongjiang Provincial CDC – Shichun Yan, Mingyuan Zou, Xue Zhou. Nangang CDC – Ziyan Guo, Quan Kang, Yanjie Li, Bo Yu, Qinai Xu. **Henan:** Henan Provincial CDC – Liang Chang, Lei Fan, Shixian Feng, Ding Zhang, Gang Zhou. Huixian CDC – Yulian Gao, Tianyou He, Pan He, Chen Hu, Huarong Sun, Xukui Zhang. **Hunan:** Hunan Provincial CDC – Biyun Chen, Zhongxi Fu, Yuelong Huang, Huilin Liu, Qiaohua Xu, Li Yin. Liuyang CDC – Huajun Long, Xin Xu, Hao Zhang, Libo Zhang. **Liuzhou:** Guangxi Provincial CDC – Naying Chen, Duo Liu, Zhenzhu Tang. Liuzhou CDC – Ningyu Chen, Qilian Jiang, Jian Lan, Mingqiang Li, Yun Liu, Fanwen Meng, Jinhuai Meng, Rong Pan, Yulu Qin, Ping Wang, Sisi Wang, Liuping Wei, Liyuan Zhou. **Qingdao:** Qingdao CDC – Liang Cheng, Ranran Du, Ruqin Gao, Feifei Li, Shanpeng Li, Yongmei Liu, Feng Ning, Zengchang Pang, Xiaohui Sun, Xiaocao Tian, Shaojie Wang, Yaoming Zhai, Hua Zhang, Licang CDC – Wei Hou, Silu Lv, Junzheng Wang. **Sichuan:** Sichuan Provincial CDC – Xiaofang Chen, Xianping Wu, Ningmei Zhang, Weiwei Zhou. Pengzhou CDC – Xiaofang Chen, Jianguo Li, Jiaqiu Liu, Guojin Luo, Qiang Sun, Xunfu Zhong. **Suzhou:** Jiangsu Provincial CDC – Jian Su, Ran Tao, Ming Wu, Jie Yang, Jinyi Zhou, Yonglin Zhou. Suzhou CDC – Yihe Hu, Yujie Hua, Jianrong Jin Fang Liu, Jingchao Liu, Yan Lu, Liangcai Ma, Aiyu Tang, Jun Zhang. **Zhejiang**: Zhejiang Provincial CDC – Weiwei Gong, Ruying Hu, Hao Wang, Meng Wang, Min Yu. Tongxiang CDC – Lingli Chen, Qijun Gu, Dongxia Pan, Chunmei Wang, Kaixu Xie, Xiaoyi Zhang.

Supplementary Figures

Figure S1. CKB Axiom® array design overview. The figure summarises the data sources used for array design and the filtering, QC, and variants selection procedures applied. Related to **Figure 2**.

Figure S2. Design of the CKB Axiom® genotyping array. The figure illustrates the different categories of content on the initial CKB array. Numbers indicate the approximate counts of variants in each category. Some variants fall into more than one category. Related to **Figure 2**.

Figure S3. CKB Axiom® array design revision overview. The figure summarises the procedures used to update the array design. Related to **Figure 2**.

Figure S4. Allele frequency of genotyped variants in CKB regions. Allele frequency of QCed variants on array v2 in each CKB region, compared with the corresponding allele in the East Asian subset of the 1000 genomes Phase 3 reference. Related to **Figure 3**.

Figure S5. Imputation quality for each CKB array version. The distribution is shown for imputation INFO score for variants in 4 MAF ranges, for the results of imputation using genotyping data from each CKB array version separately. Related to **STAR Methods**.

Figure S6. Patterns of relatedness in CKB regions. The histograms show for each CKB region the distributions of the relatedness between all possible pairs of genotyped individuals. Related to **STAR Methods**.

Figure S7. Quality control for heterozygosity and homozygosity. Overall heterozygosity and total runs of homozygosity were determined for each genotyping dataset. Blue symbols denote samples with low heterozygosity that is accounted for by extended runs of homozygosity. Red symbols, indicated by an arrow, denote samples whose heterozygosity is not accounted for by runs of homozygosity and which were excluded from the analysis dataset. Related to **STAR Methods**.

Figure S8. Identification of informative principal components. Models predicting participant recruitment region were constructed by progressively adding PCs from PCA of the full CKB cohort, and Bayes Information Criterion was determined. Related to **STAR Methods**.

Figure S9. Principal component analysis of CKB. The results of PCA of the full CKB genotyped dataset are shown for pairwise plots of all PCs that were informative for CKB recruitment region. Data points are colour coded according to the region from which that participant was recruited. Related to **Figure 4**.

Figure S10. Population structure in CKB regions as informed by whole cohort PCA. Local maps are shown for each recruitment region, showing the geolocation of the individual recruitment clinics, colour coded according to latitude and longitude; the size of the symbol is proportional to the number of genotyped individuals from that clinic. Corresponding PCA plots show the first two principal components from PCA of the full CKB cohort, colour coded according to their recruitment clinic. Top 2 rows ¬– urban regions; bottom 2 rows – rural regions. Related to **Figure 4**.

Figure S11. Identification of informative principal components for CKB regions. Models predicting the latitude (blue) and longitude (red) of participants' recruitment clinic were constructed by progressively adding PCs, and Bayes Information Criterion was determined. Broken lines – PCA of the entire CKB cohort; solid lines – PCA of each region separately. Related to **STAR Methods**.

Figure S12. Population structure in Liuzhou region. Participants recruited in Liuzhou who attended the second resurvey are plotted according to PCA from the entire CKB cohort (top) or Liuzhou only (bottom). Blue – self-reported Han ancestry; orange – mixed ancestry; red – non-Han ancestry. Plots (right) show the Bayes Information Criterion for models predicting Han status using increasing numbers of PCs. Related to **Figure 4**.

Figure S13. Population diversity in CKB. Population differences as measured by Fst were derived, and trees were constructed to illustrate the relationships between them the populations shown. (A) Phylogenetic tree derived using the full unrelated CKB dataset, except for Liuzhou (RC46) for which only second resurvey participants were included. (b) Neighbour-joining tree constructed using 100 unrelated individuals from each population. RC12 – Qingdao; RC16 – Harbin; RC26 – Haikou; RC36 – Suzhou; RC46 – Liuzhou; RC52 – Sichuan; RC58 – Gansu; RC68 – Henan; RC78 – Zhejiang; RC88 – Hunan; CHB, CHS, JPT, CDX, KHV – East Asian 1000 Genomes populations. Related to **Figure 4**.

Figure S14. PCA projection onto 1000 Genomes. PCA was conducted for the 1000 Genomes Phase 3 populations, and CKB participants were projected onto the resulting PCs. Top – 1000 Genomes populations; bottom – with CKB participants (black) included. Related to **STAR Methods**.

Figure S15. Identification of sex mismatches and chromosome XY aneuploidies. (A) plot showing relationship between chromosome X homozygosity and chromosome XY probe ratio. Sex-mismatched samples are visible within the main clusters of females (red) and males (blue). Open symbols denote samples identified as potential aneuploidies. (B) Plots across chromosome X of the BAF parameter which reflects the proportion of signal on the genotyping array coming from the two possible alleles at each site. 3 classes of aneuploidy are illustrated, the red marks highlighting systematic deviations from the expected 3 possible genotypes. Related to **STAR Methods**.

Figure S16. Distortion of PCA by regions of long range LD. Plots show individual variant loadings (Z-squared), for each of the first 12 PCs from PCA of the full CKB cohort, that result if regions of long range LD are not fully excluded. Related to **STAR Methods**.

Figure S1

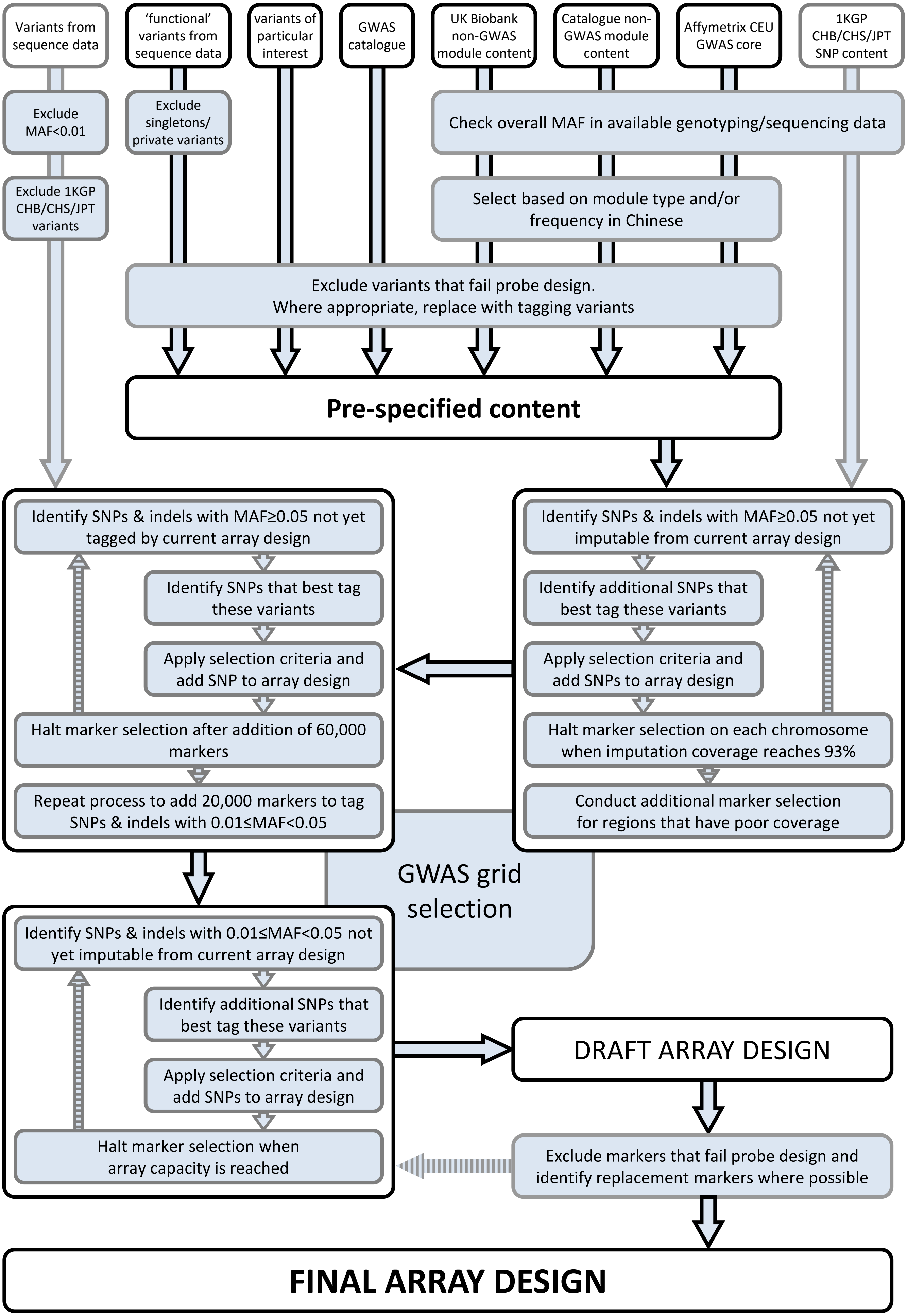
Variants from sequence data
'functional' variants from sequence data
variants of particular interest
GWAS catalogue
UK Biobank non-GWAS module content
Catalogue non-GWAS module content
Affymetrix CEU GWAS core
1KGP CHB/CHS/JPT SNP content
Exclude MAF<0.01
Exclude singletons/ private variants
Check overall MAF in available genotyping/sequencing data
Exclude 1KGP CHB/CHS/JPT variants
Select based on module type and/or frequency in Chinese
Exclude variants that fail probe design.
Where appropriate, replace with tagging variants
Pre-specified content
Identify SNPs & indels with MAF≥0.05 not yet tagged by current array design
Identify SNPs that best tag these variants
Apply selection criteria and add SNP to array design
Halt marker selection after addition of 60,000 markers
Repeat process to add 20,000 markers to tag SNPs & indels with 0.01≤MAF<0.05
Identify SNPs & indels with MAF≥0.05 not yet imputable from current array design
Identify additional SNPs that best tag these variants
Apply selection criteria and add SNPs to array design
Halt marker selection on each chromosome when imputation coverage reaches 93%
Conduct additional marker selection for regions that have poor coverage
GWAS grid selection
Identify SNPs & indels with 0.01≤MAF<0.05 not yet imputable from current array design
Identify additional SNPs that best tag these variants
Apply selection criteria and add SNPs to array design
Halt marker selection when array capacity is reached
DRAFT ARRAY DESIGN
Exclude markers that fail probe design and identify replacement markers where possible
FINAL ARRAY DESIGN

Figure S2

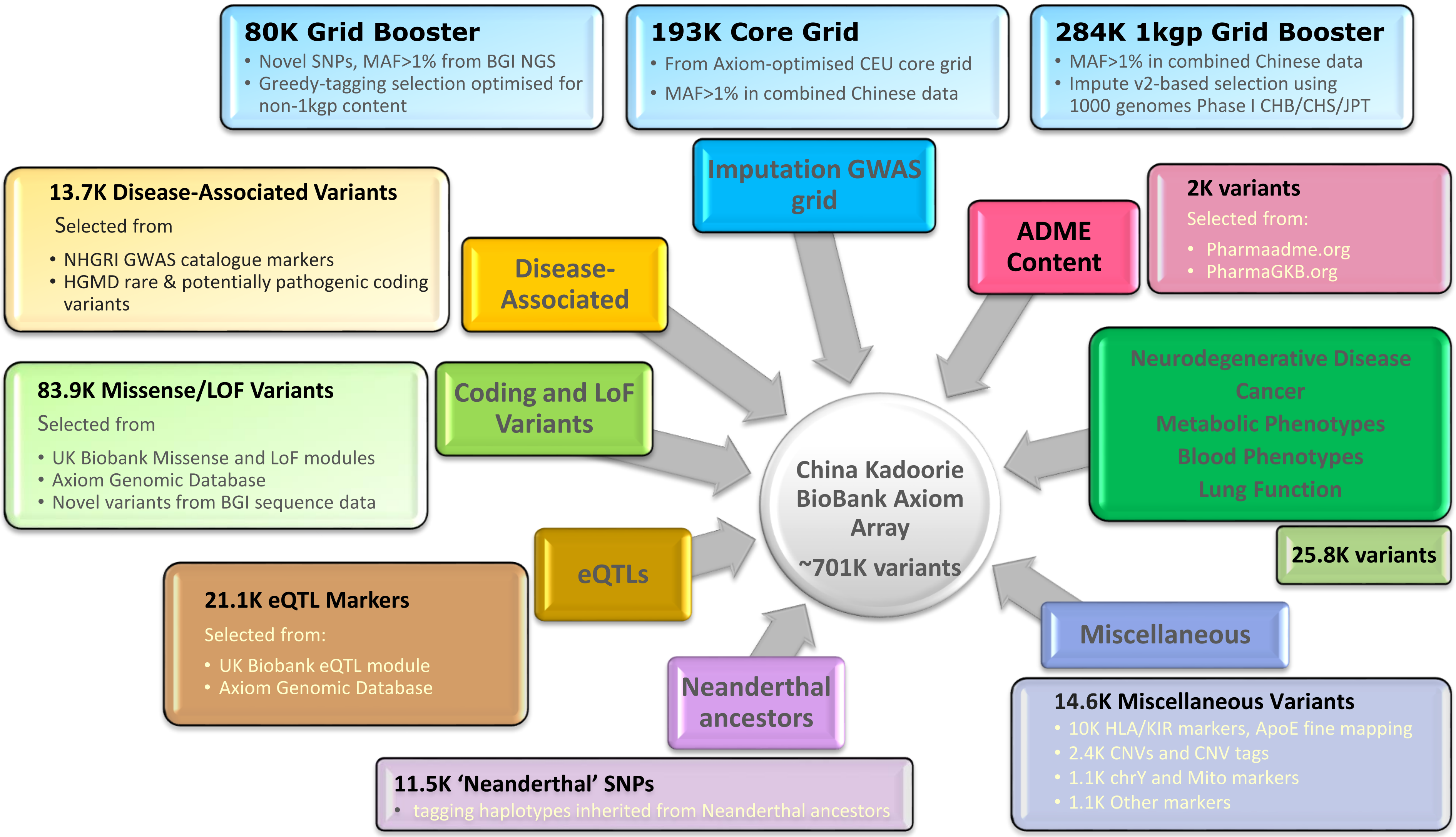
80K Grid Booster
Novel SNPs, MAF>1% from BGI NGS
Greedy-tagging selection optimised for non-1kgp content
193K Core Grid
From Axiom-optimised CEU core grid
MAF>1% in combined Chinese data
284K 1kgp Grid Booster
MAF>1% in combined Chinese data
Impute v2-based selection using 1000 genomes Phase I CHB/CHS/JPT
13.7K Disease-Associated Variants
Selected from
NHGRI GWAS catalogue markers
HGMD rare & potentially pathogenic coding variants
Disease-Associated
Imputation GWAS grid
ADME Content
2K variants
Selected from:
Pharmaadme.org
PharmaGKB.org
83.9K Missense/LOF Variants
Selected from
UK Biobank Missense and LoF modules
Axiom Genomic Database
Novel variants from BGI sequence data
Coding and LoF Variants
China Kadoorie BioBank Axiom Array
~701K variants
Neurodegenerative Disease
Cancer
Metabolic Phenotypes
Blood Phenotypes
Lung Function
25.8K variants
eQTLs
21.1K eQTL Markers
Selected from:
UK Biobank eQTL module
Axiom Genomic Database
Neanderthal ancestors
Miscellaneous
14.6K Miscellaneous Variants
10K HLA/KIR markers, ApoE fine mapping
2.4K CNVs and CNV tags
1.1K chrY and Mito markers
1.1K Other markers
11.5K 'Neanderthal' SNPs
tagging haplotypes inherited from Neanderthal ancestors

Figure S3

Review results from first 100 plates

Poly High Resolution	Review, default=retain
No Minor Homozygote	Review, default=retain
Mono High Resolution	Review and check frequency in CONVERGE
Hemizygous	Review
Off Target Variant	Review, default=exclude
Call Rate Below Threshold	Review, default=exclude
Other	Review, default=exclude
Viral probes	Review, default=retain

Identify unneeded 2nd probesets
Check call rate for all retained SNPs

Remove from design

- Failed and low-quality assays
- Non-preferred probesets
- Monomorphic variants (except for selected variants e.g. those confirmed at acceptable frequencies in CONVERGE)
- Viral probes?

Add to design

- Modified assays for retained SNPs
- Tag SNPs for important failed assays
- Novel content from CONVERGE
- New GWAS hits
- Additional content from collaborators

Augment GWAS grid

- Restore gaps caused by SNP removal
- Supplement low freq. grid

Figure S4

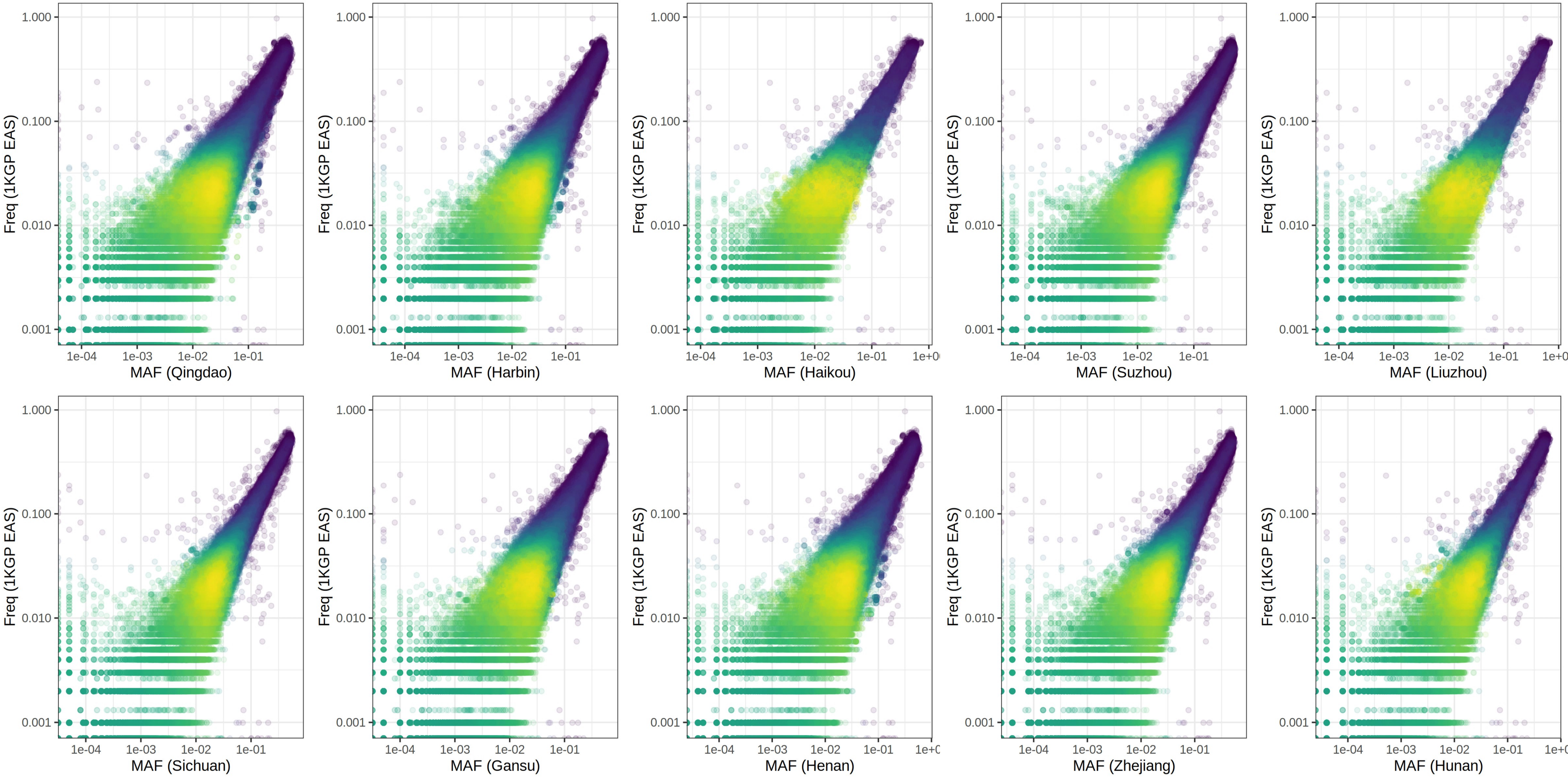
Freq (1KGP EAS)
1.000
0.100
0.010
0.001
1e-04
1e-03
1e-02
1e-01
1e+0
MAF (Qingdao)
MAF (Harbin)
MAF (Haikou)
MAF (Suzhou)
MAF (Liuzhou)
MAF (Sichuan)
MAF (Gansu)
MAF (Henan)
MAF (Zhejiang)
MAF (Hunan)

Figure S5

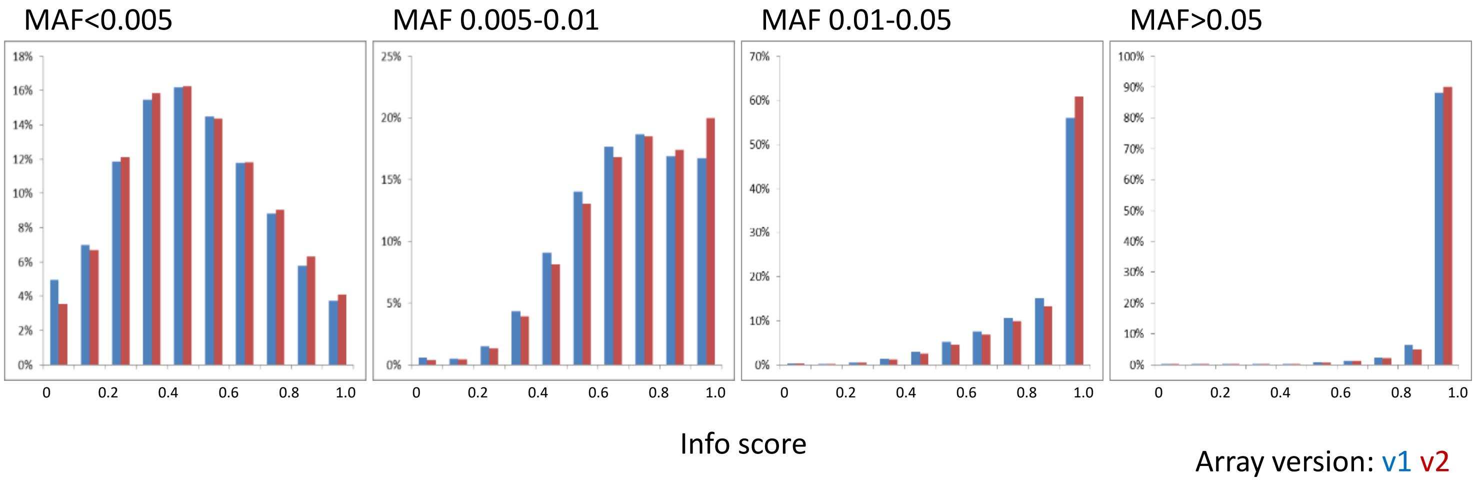

MAF<0.005
MAF 0.005-0.01
MAF 0.01-0.05
MAF>0.05
0
0.2
0.4
0.6
0.8
1.0
Info score
Array version: v1 v2

Figure S6

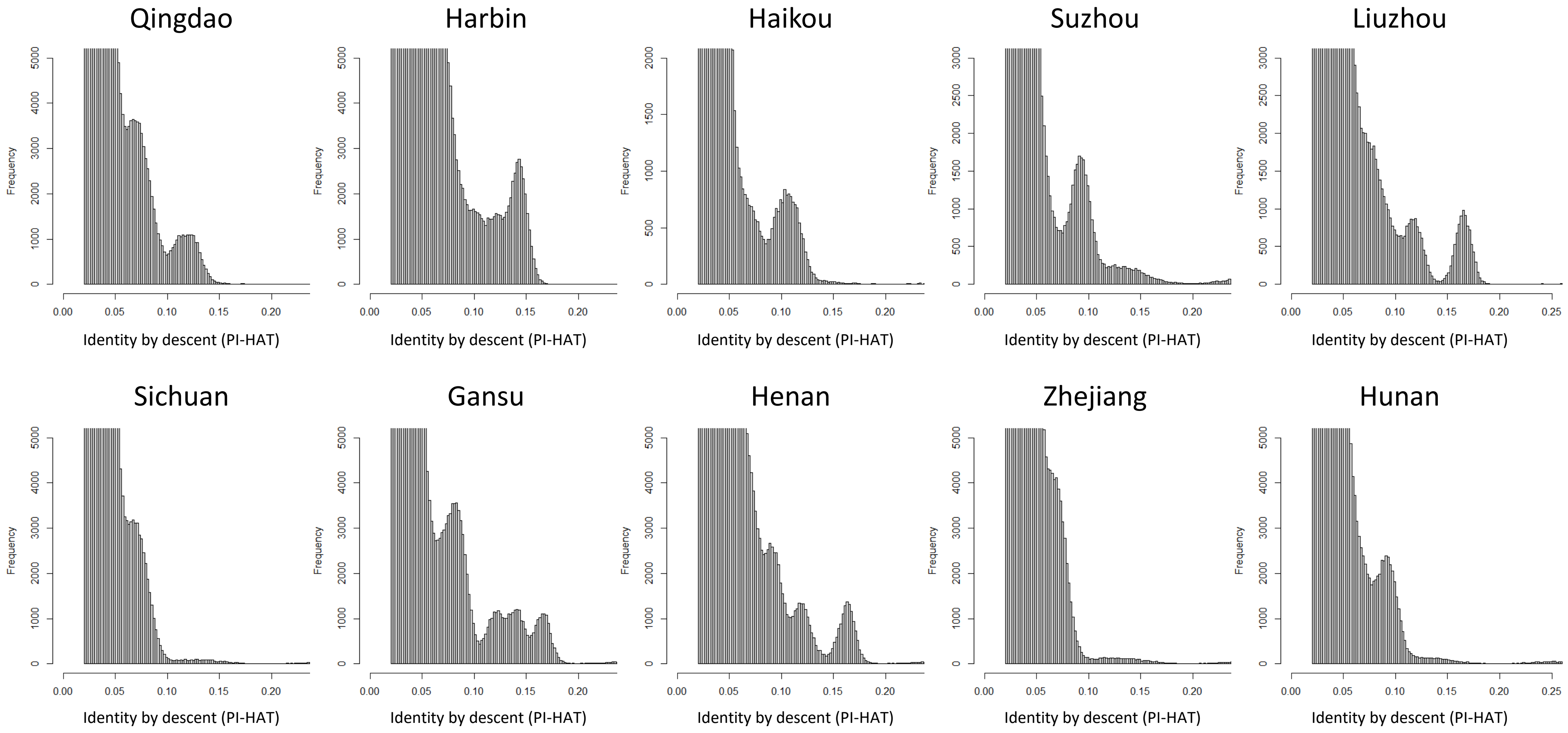
Qingdao
Harbin
Haikou
Suzhou
Liuzhou
Sichuan
Gansu
Henan
Zhejiang
Hunan
Frequency
Identity by descent (PI-HAT)

Figure S7

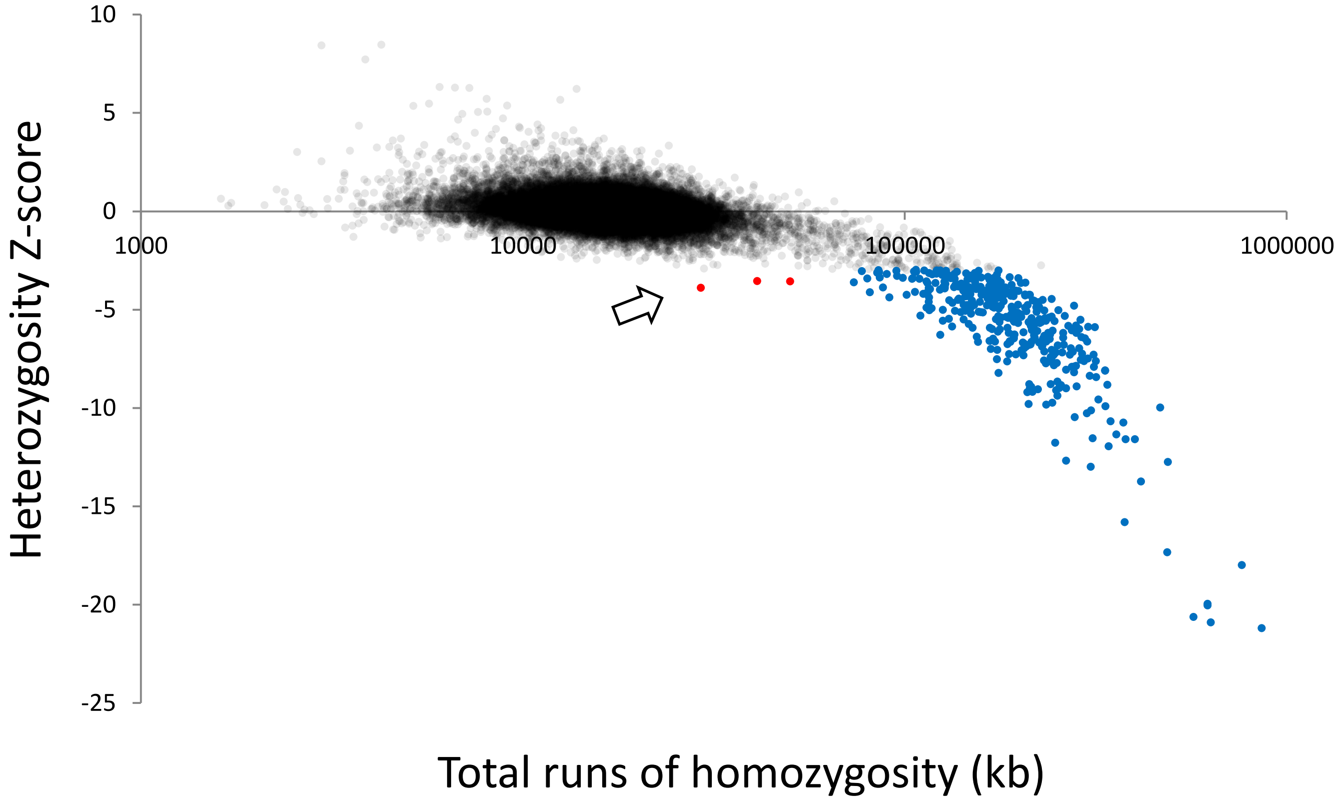

10
5
0
-5
-10
-15
-20
-25
1000
10000
100000
1000000
Heterozygosity Z-score
Total runs of homozygosity (kb)

Figure S8

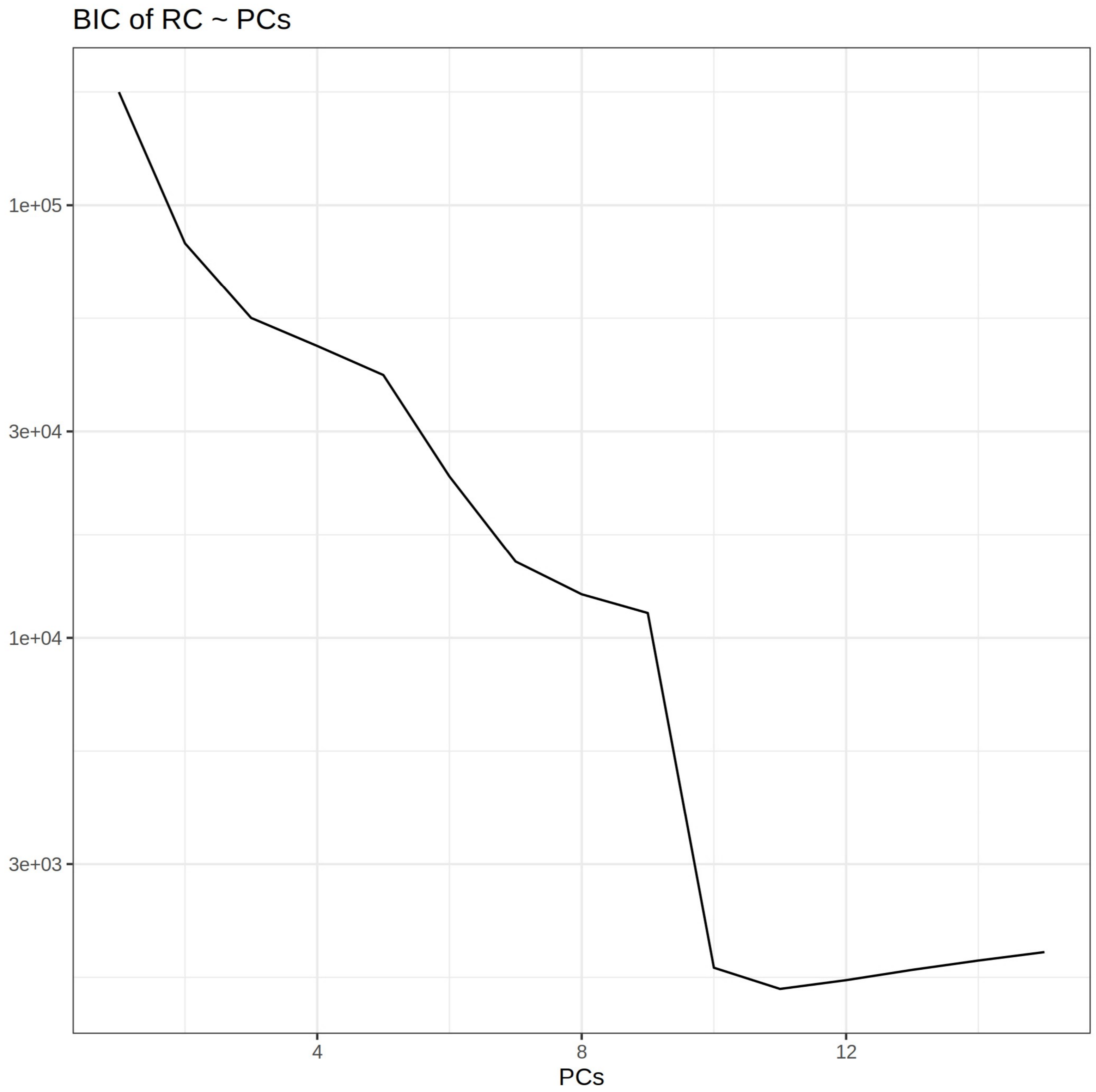

Figure S9

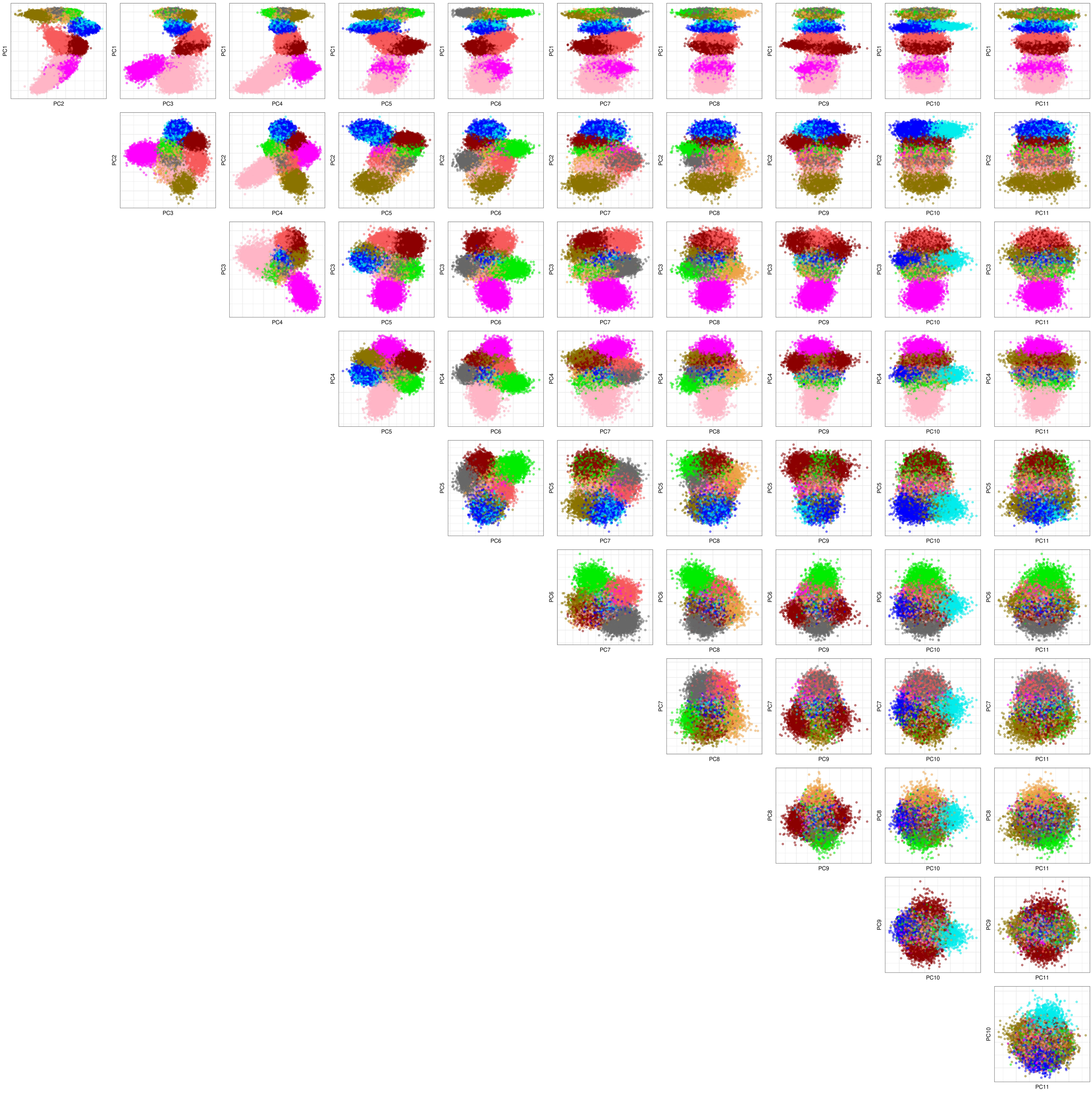

Figure S10

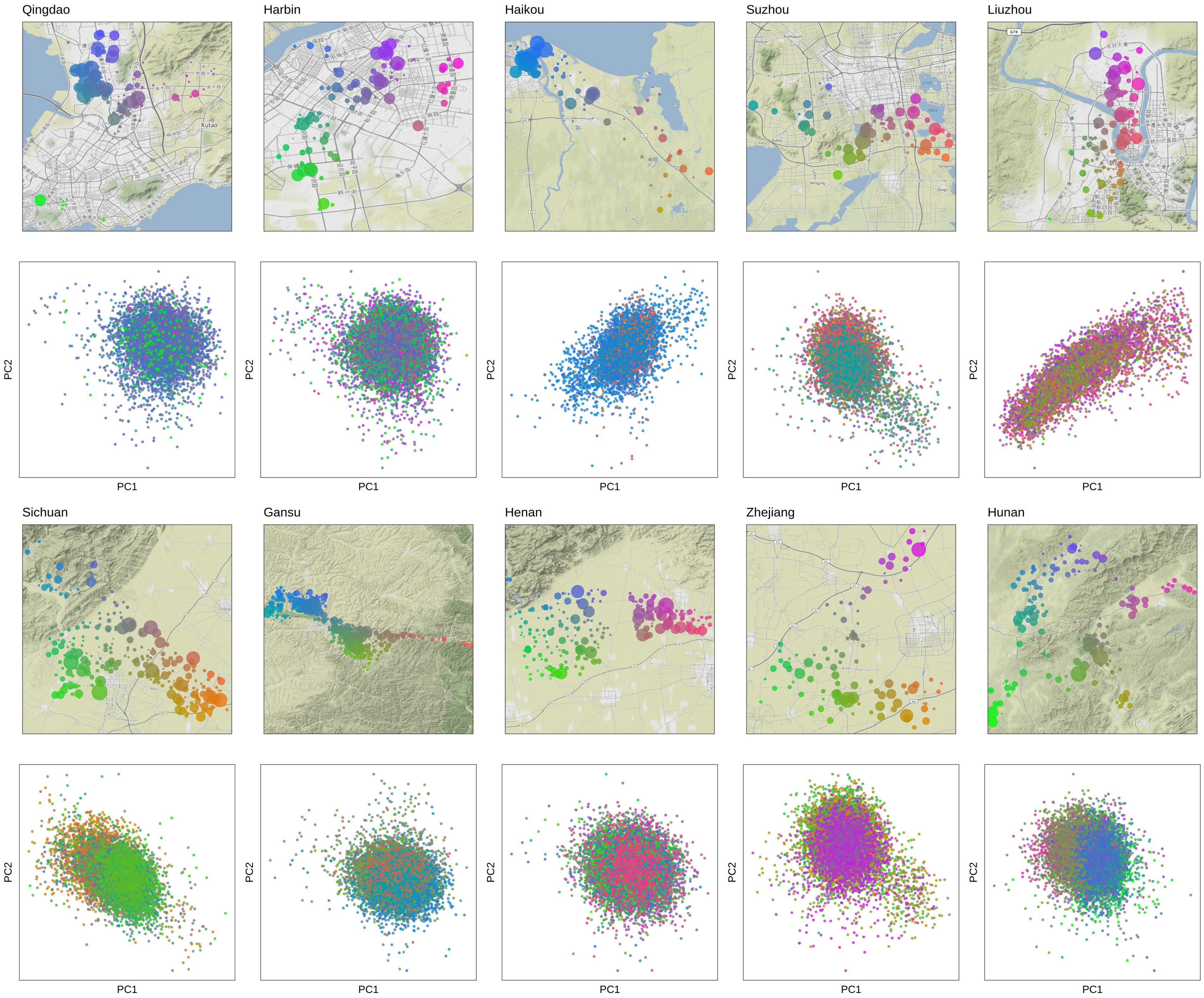

Qingdao
Harbin
Haikou
Suzhou
Liuzhou
PC2
PC1
Sichuan
Gansu
Henan
Zhejiang
Hunan

Figure S11

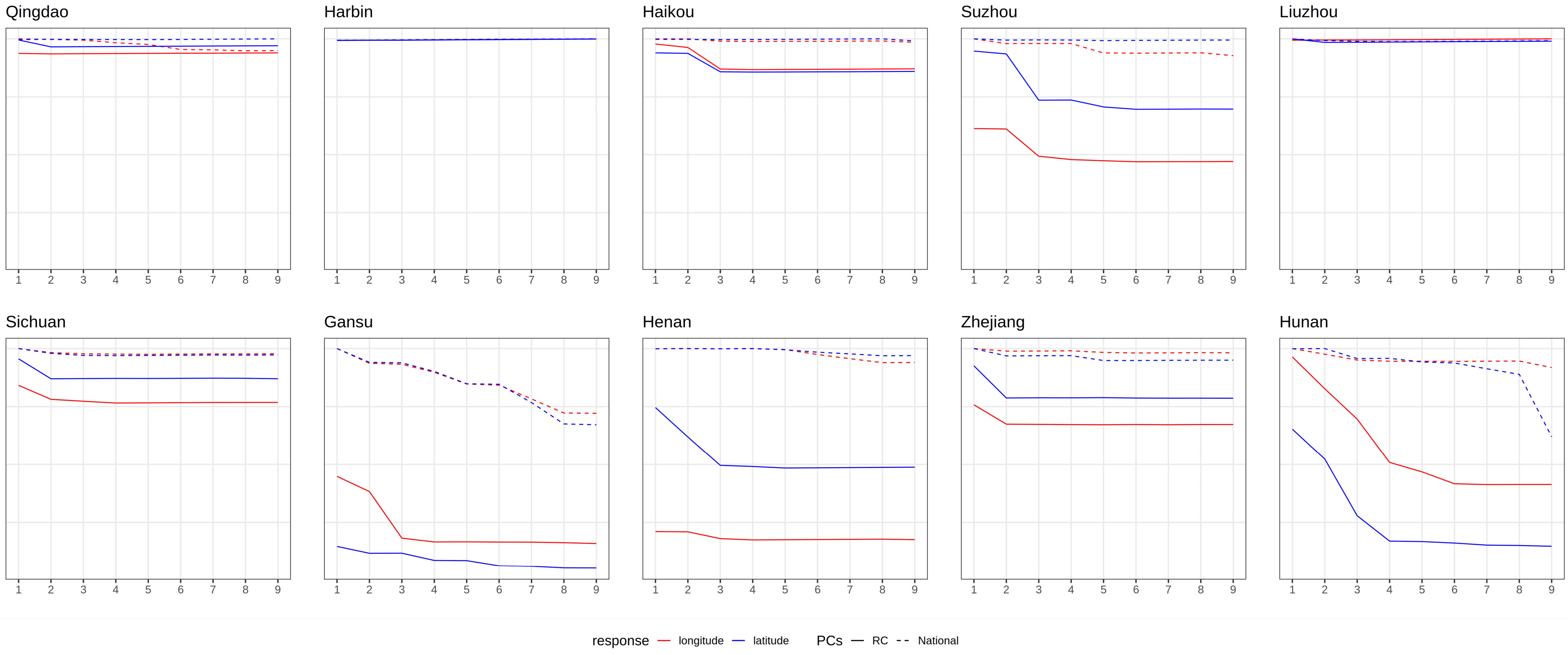

Qingdao
Harbin
Haikou
Suzhou
Liuzhou
Sichuan
Gansu
Henan
Zhejiang
Hunan
1 2 3 4 5 6 7 8 9
response longitude latitude
PCs RC National

Figure S12

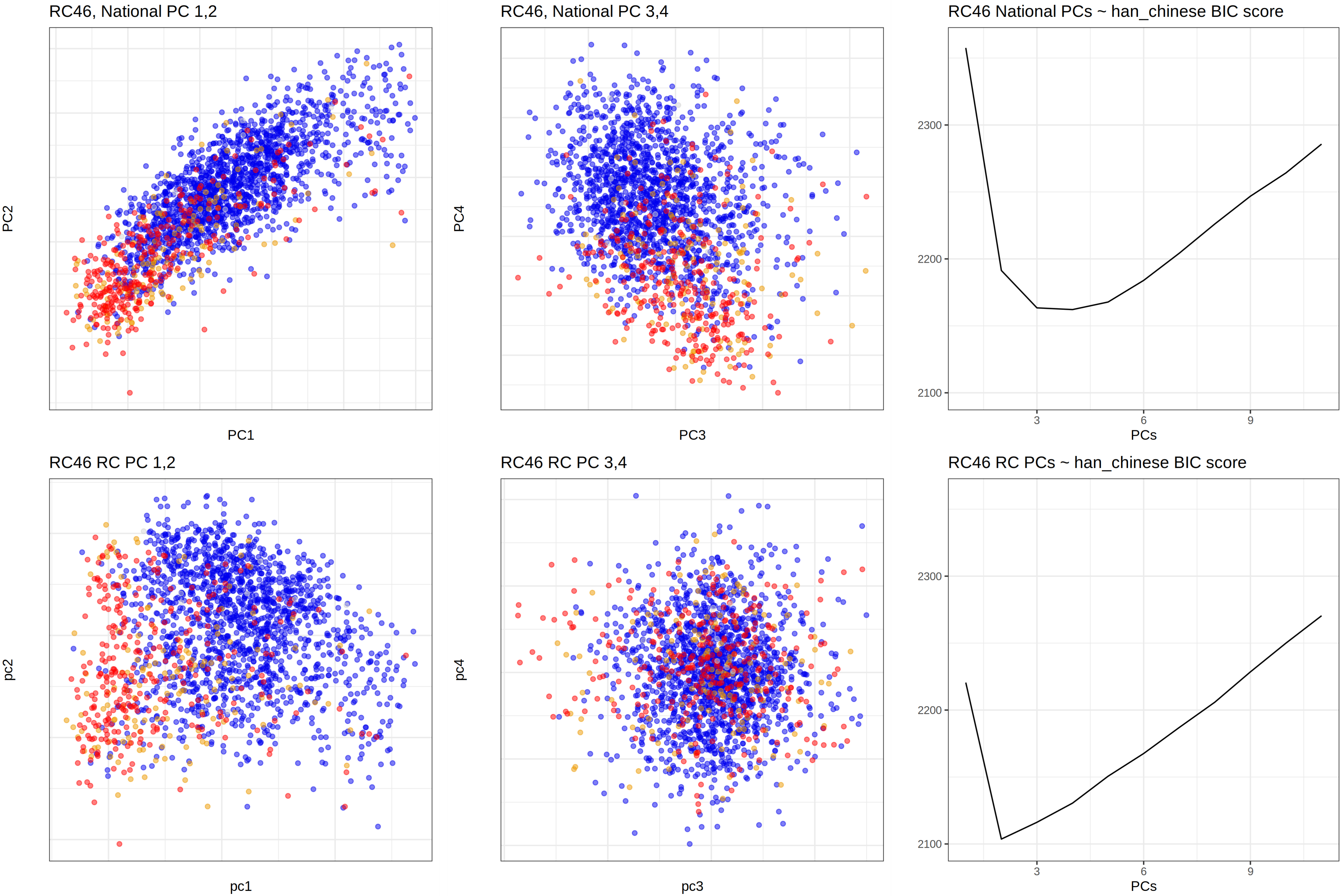

RC46, National PC 1,2
PC2
PC1
RC46, National PC 3,4
PC4
PC3
RC46 National PCs ~ han_chinese BIC score
2300
2200
2100
3
6
9
PCs
RC46 RC PC 1,2
pc2
pc1
RC46 RC PC 3,4
pc4
pc3
RC46 RC PCs ~ han_chinese BIC score
2300
2200
2100
3
6
9
PCs

Figure S13

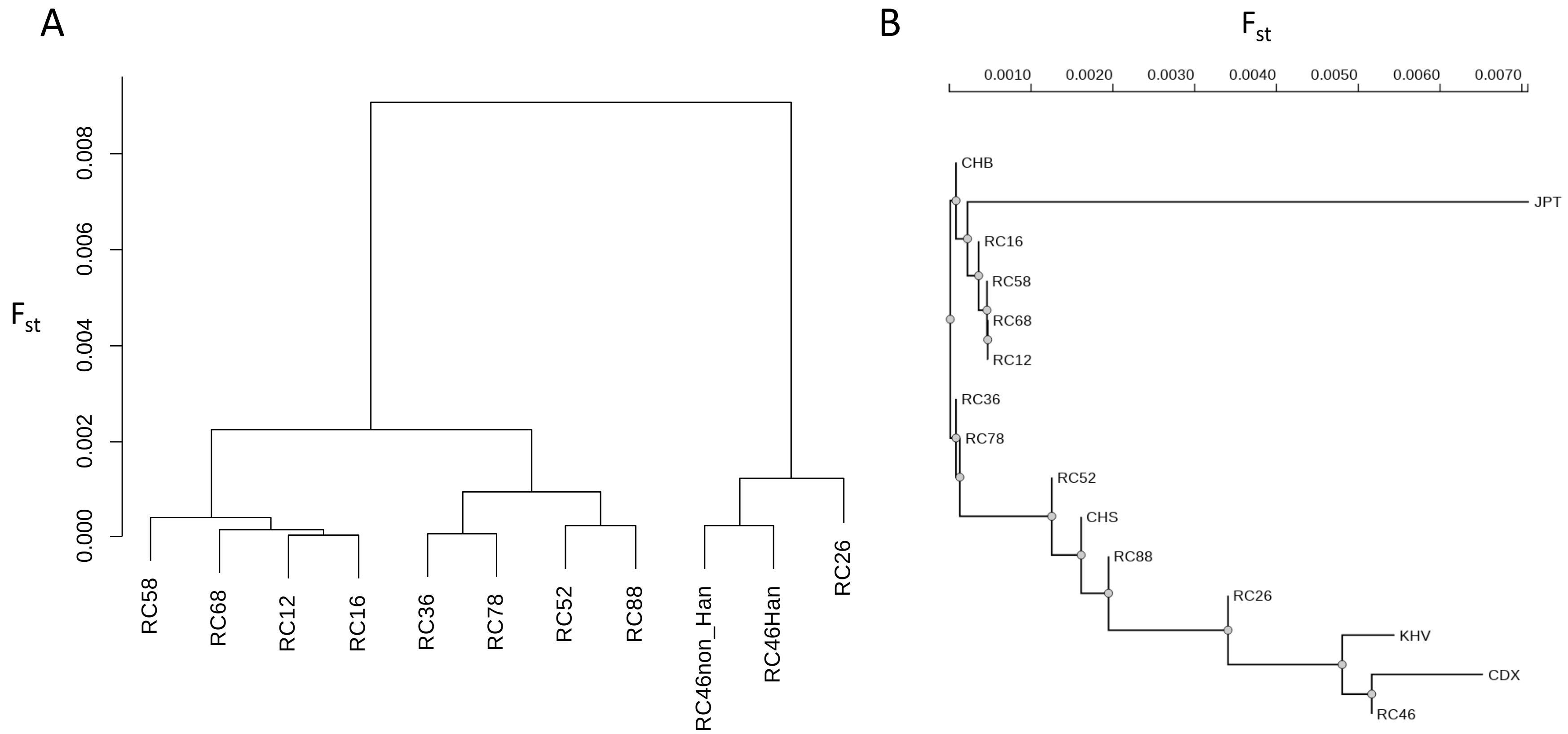
A
Fst
0.000
0.002
0.004
0.006
0.008
RC58
RC68
RC12
RC16
RC36
RC78
RC52
RC88
RC46non_Han
RC46Han
RC26
B
Fst
0.0010
0.0020
0.0030
0.0040
0.0050
0.0060
0.0070
CHB
JPT
RC16
RC58
RC68
RC12
RC36
RC78
RC52
CHS
RC88
RC26
KHV
CDX
RC46

Figure S14

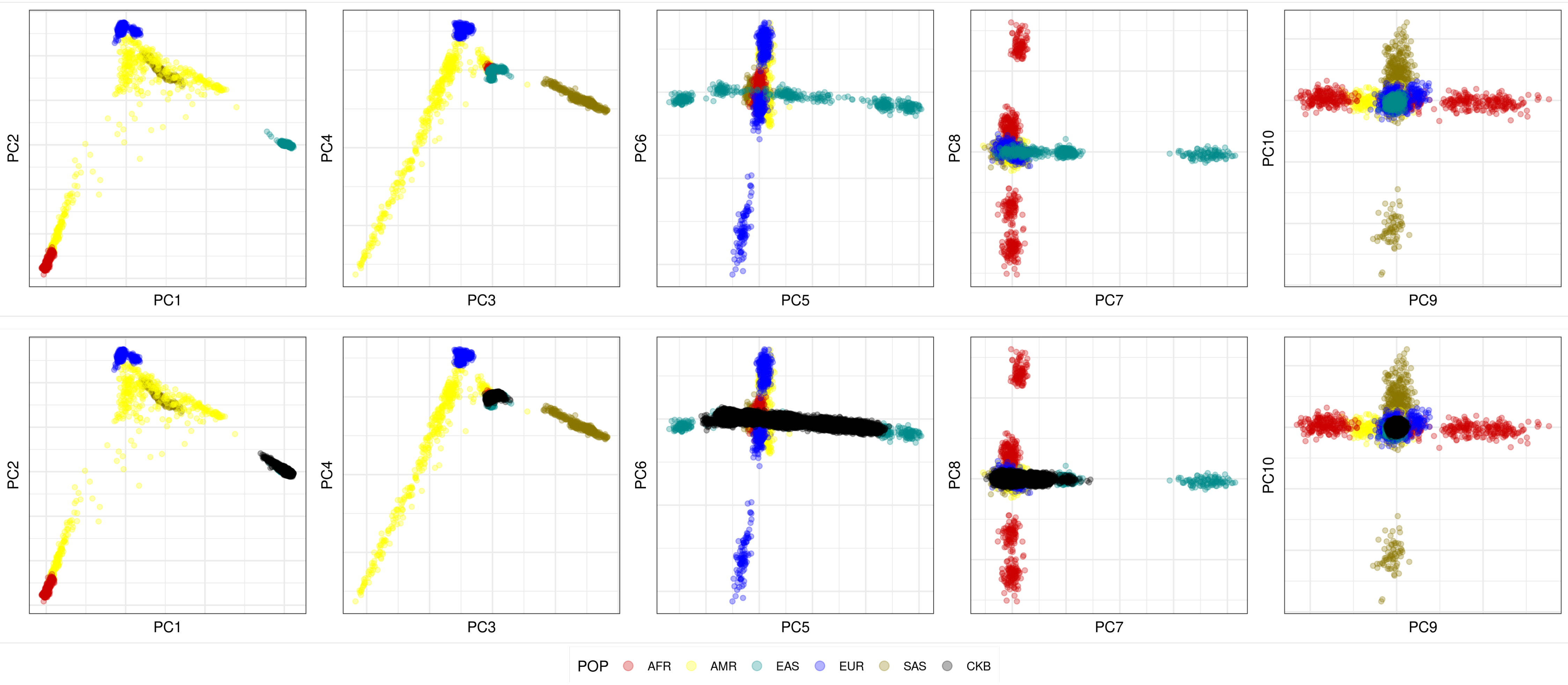

PC2
PC1
PC4
PC3
PC6
PC5
PC8
PC7
PC10
PC9
PC2
PC1
PC4
PC3
PC6
PC5
PC8
PC7
PC10
PC9
POP
AFR
AMR
EAS
EUR
SAS
CKB

Figure S15

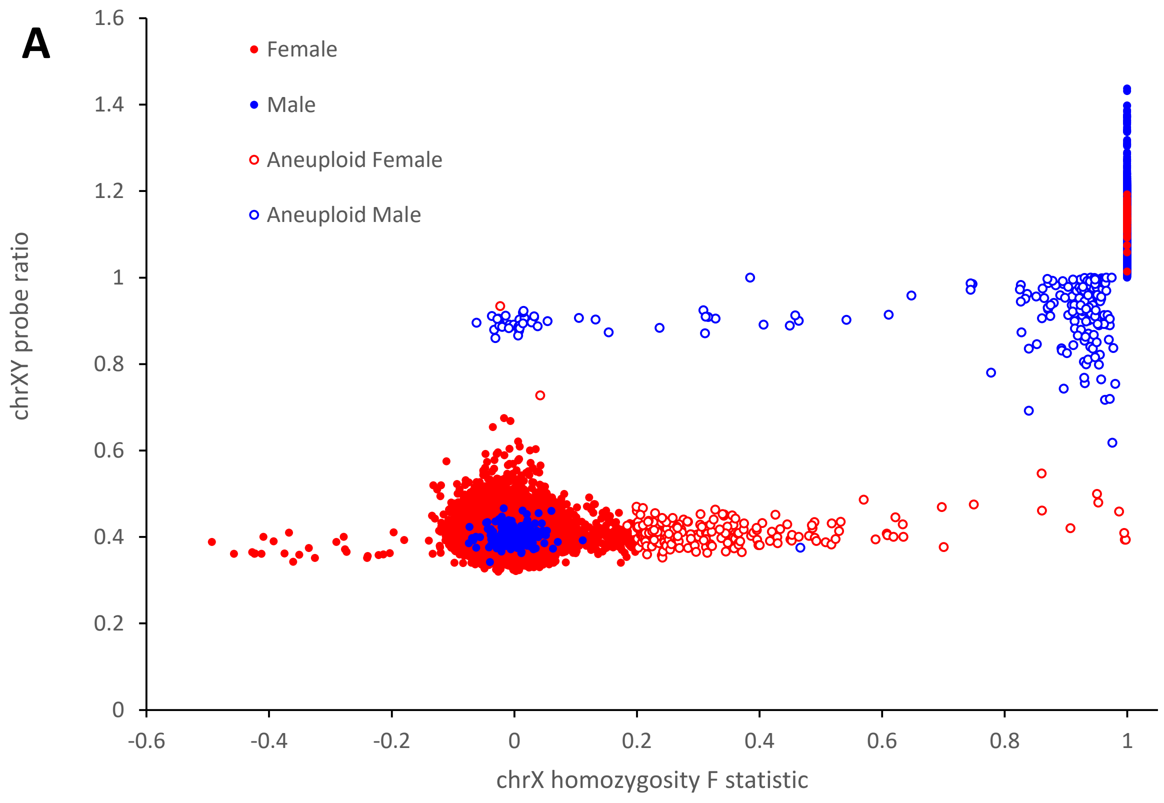

A
1.6
1.4
1.2
1
0.8
0.6
0.4
0.2
0
Female
Male
Aneuploid Female
Aneuploid Male
chrXY probe ratio
-0.6
-0.4
-0.2
0
0.2
0.4
0.6
0.8
1
chrX homozygosity F statistic

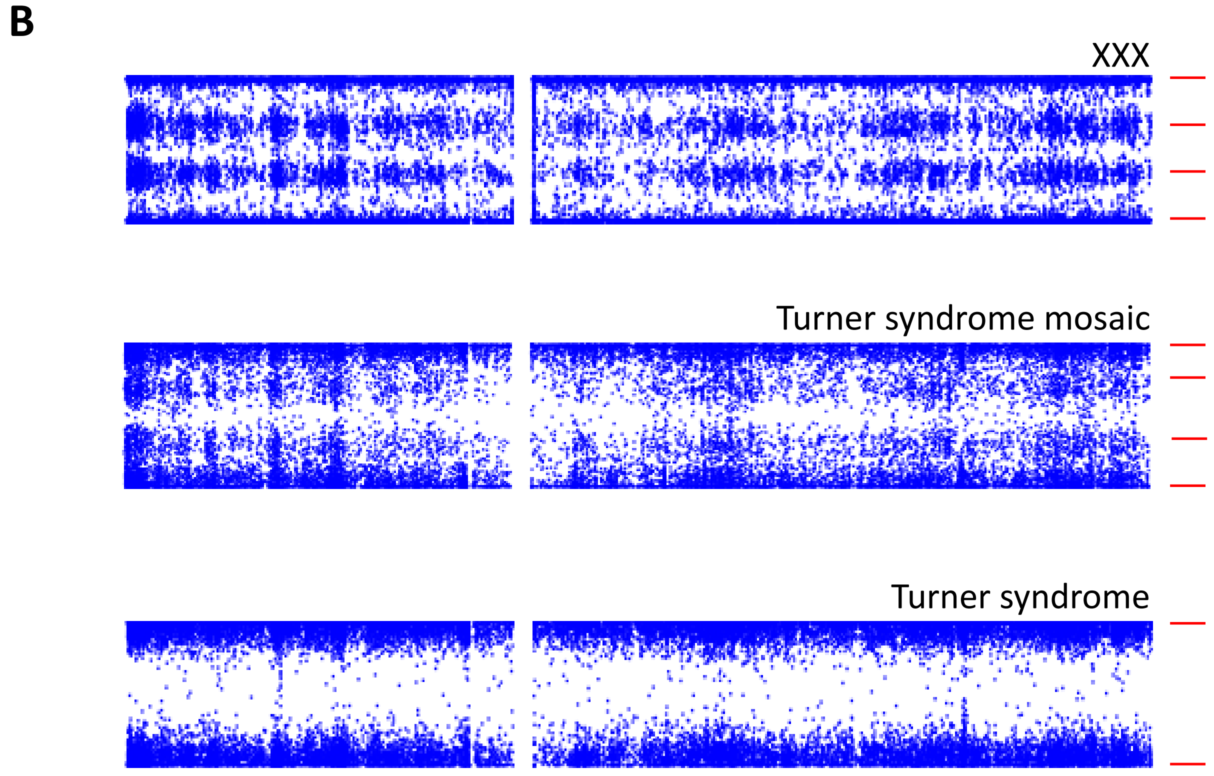

B
XXX
Turner syndrome mosaic
Turner syndrome

Figure S16

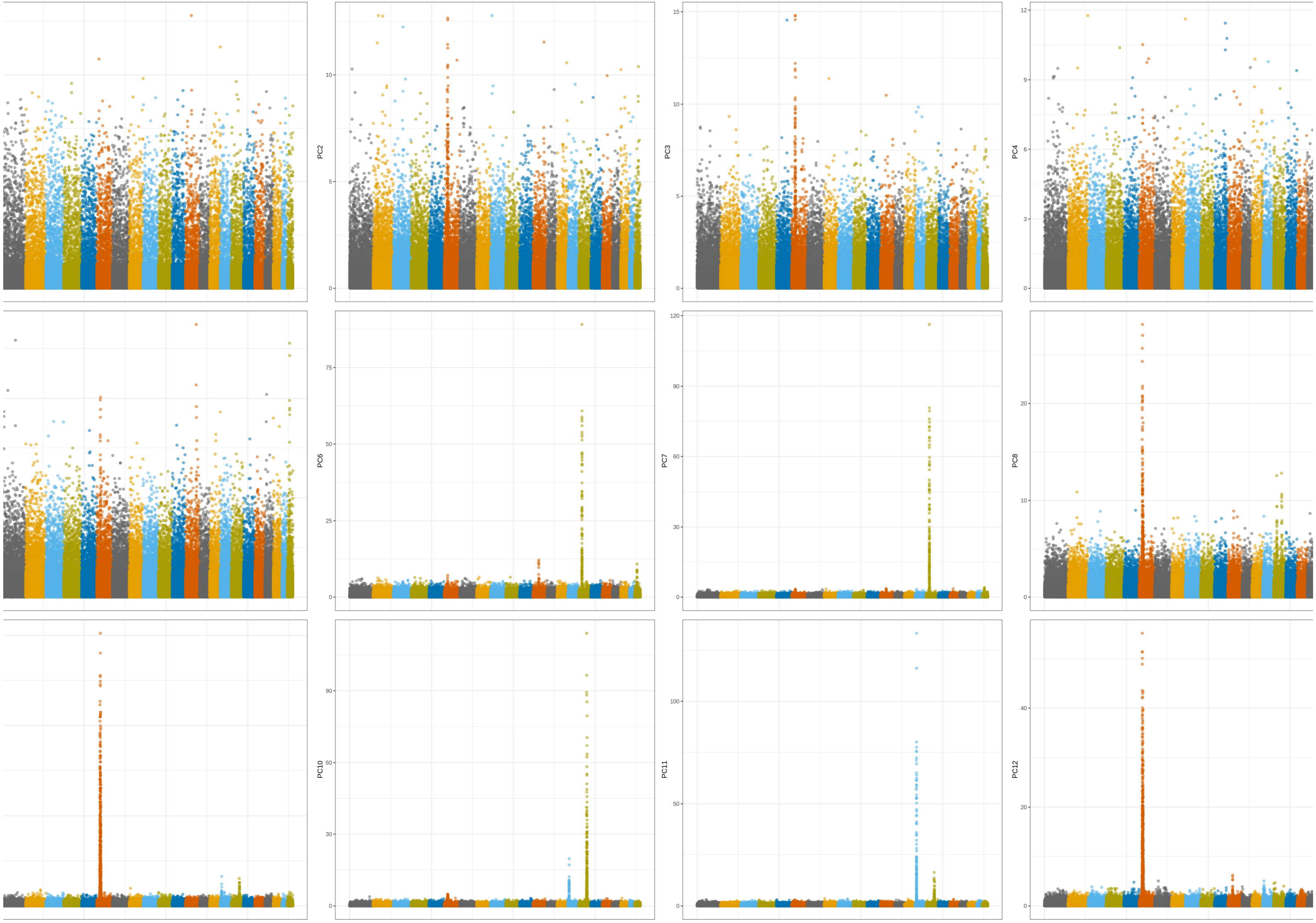

Supplementary Data S1. China Kadoorie Biobank Array Design. Related to **Figure 2.**

The overall scheme for design of the CKB array is shown in **Figure S1**. Array content was selected from 8 distinct (but overlapping) classes:

1. Variants specified for various purposes by the CKB study group and collaborators;
2. Known GWAS hits present in the GWAS Catalog plus additional loci provided by colleagues and collaborators prior to publication;
3. Putative 'functional' variants identified in BGI sequencing data;
4. Content from (non-GWAS) modules defined for the UK Biobank array design;
5. Content from (non-GWAS) modules from Affymetrix catalogue arrays;
6. The optimised CEU core GWAS module, as used in the UK Biobank array;
7. ASN (JPT/CHB/CHS) SNPs/indels identified by HapMap/1000 genomes;
8. SNPs/indels with MAF>0.01 in unpublished sequence data (BGI).
9. Viral sequences for detection of Hepatitis B virus infection/subtypes;

The data sources used to select content included:

a. 1000 genomes Phase I data from 197 CHB/CHS subjects
b. 1000 genomes pilot data from 91 CHB subjects (for SNPs absent from the Phase I data)
c. BGI high coverage WGS data for 156 of the CHS/CHB 1000genomes subjects
d. BGI WGS data from 1,746 exomes from subjects mainly from southern China
e. Allele frequency data from genotyping of 1,802 subjects using the Taiwan Biobank array
f. Low coverage sequencing data from ~9,000 Chinese from the CONVERGE consortium

Together, these were used (A) to define pre-specified content on the array; and (B) to build a Chinese-optimised GWAS grid.

For use in SNP selection (and for determining MAF-defined SNP target lists), allele frequencies from (f) were used where available. Otherwise, data from (a/b), (d), (e) were combined, except that the 1000 genomes data (a) were replaced by BGI WGS data (c) if the latter gave a non-zero number of minor alleles – these high-coverage data were expected to be more accurate than the 1000 genomes low-coverage data.

The principles underlying the array design included:

- Maximising overlap with the UK Biobank array
- Taking account of Chinese-specific content
- Ensuring detection of specific important variants
- Maximising space-efficiency of the selected variants

Thus, the UK Biobank design was taken as a starting point, with the default being to include UKB content unless there was a good reason not to do so.

A. Pre-Specified Content

Pre-specified content was determined as follows. The marker counts given are for those that survived probe design QC.

1. *Affymetrix CEU GWAS core*

The optimised CEU GWAS core (UK Biobank module 'GWAS Grid') contained 246,055 SNPs and indels. SNPs/indels were removed from this list if they had MAF<0.01 or were not present in the 1000 genomes Phase I data (used by Affymetrix for imputation aware SNP selection) and, therefore, were not able to contribute to building of the GWAS grid, leaving 191,056 SNPs. Some markers were subsequently added back due to being in other modules (see below) or during GWAS grid selection, so that in total 193,326 markers from this module were included on the array.

2. *Other UK Biobank non-GWAS module content*

<u>Markers found in Chinese samples:</u>

All SNPs on the UK Biobank array that had been specified for some reason other than GWAS were checked for their presence in Chinese populations. All variants identified in at least one individual in the datasets above were provisionally included. This included all variants in several UKB modules.

<u>Markers not found in Chinese samples:</u>

The remaining variants, for which no instance of the minor allele was identified in Chinese samples, were treated as follows:

<u>'HLA/KIR', 'KIR', 'chrMT', 'chrY', 'ApoE', 'CNV Coverage', 'CNV tag', 'Fingerprint', 'ADME', 'Blood', 'BP/HT', 'Neanderthal', 'Alzheimers', 'eQTL', 'Lung Function':</u> A relatively small proportion of these modules were not found in the available Chinese data. It was decided to include all such variants on the array, irrespective of detection in Chinese.

<u>'Cancer', 'HGMD', 'cardiac', 'neuro':</u> 6 well-evidenced cancer-related SNPs common in CEU but not already included were included. The remaining markers not already selected and present only in these modules were excluded.

<u>'missense' or 'LOF' modules</u>. These were mainly low-frequency variants selected on the basis of detection in the UK population. Such variants not present in Chinese were excluded.

Altogether, a further 100,411 variants were added to the array design, giving a total of 293,737.

3. *Affymetrix modules*

Markers from the Affymetrix catalogue modules eQTL, Exome319 and LOF, that were not included on the UK Biobank design but were detected in one or more of our Chinese datasets, were added to the array. The additional markers totalled 33,546, giving 327,283 in total.

4. *Known GWAS hits*

The NHGRI catalogue was downloaded on 6 December 2013. 11,745 unique lead SNPs were identified. These were merged with the UK Biobank 'GWAS compatibility' module, which included some tag SNPs (i.e. some loci had both the original hit and a tag SNP) and unpublished GWAS loci, giving a total of 12,735 markers. All of these were included on the array (QC permitting), irrespective of their prior detection in Chinese populations. As a result, a further 5,626 SNPs were added to the array, giving 332,909 in total.

5. CKB Collaborative Group-selected SNPs

683 SNPs and indels were specified by the CKB group and/or collaborators – including all SNPs previously successfully genotyped on the Illumina Golden Gate platform in 100,000 subjects – many of which were already included on the array. As a result, a further 252 SNPs were added to the array, giving 333,161 in total.

6. Functional and chrY/MT SNPs from BGI data

BGI provided data for 10,662 coding SNPs novel to Chinese (many completely novel) with putative functional effects (missense and nonsense), identified in datasets (c) and (d), and also some novel chrY/MT variants. To avoid private variants or calls due to sequencing errors, those identified in 1-2 individuals were excluded. Novel chrY/MT variants from Taiwan Biobank data were also included. In total, 5,288 additional variants were included on the final array design, giving 338,449 in total.

7. Detection of Hepatitis B virus infection and type

The available aligned HBV sequence data in late 2013 were downloaded from https://hbvdb.ibcp.fr/HBVdb/HBVdbIndex and used to calculate the entropy – i.e. the amount of variation – for each site along the genome. A sliding window of 71bp was then used to calculate the average entropy along the sequences, to identify the most conserved regions suitable for probe design. Six regions of approximately 100bp were identified, one of which contained a series of sequence variants that were expected to be sufficient to distinguish between HBV Genotypes B and C (the most common in China). For each candidate probe region, sequence variation at each site was tallied. Sites with more than one variant with a frequency ≥0.01 were recorded as 'SNPs' which would be catered for during probe design. The frequencies of all other variants (i.e. with frequency <0.01) were summed and recorded as 'residual variation' at each site.

Probe design was carried out based on sites that could be treated as 2-allele SNPs for the purposes of array data analysis. 35-mers in each direction from such 'SNPs' were recorded, treating multiallele 'SNPs' within those probes as degenerate positions requiring the design of multiple probe sequences. For each candidate 35-mer, residual variation across its full length was summed, to give a parameter "risk of probe failure" – viruses with a lot of inter-individual residual variation in these regions would potentially remain undetected due to poor probe hybridisation. Different alternative probes for each candidate probe region were compared in terms of their degeneracy and risk of failure, and the location of each probe set was selected so as to minimise these parameters.

For the majority of probe regions, whose primary purpose was to provide 'yes/no' detection of HBV, non-overlapping probe sets were selected. However, for the region diagnostic for Genotypes B and C, multiple overlapping probesets were designed so as to ensure that all diagnostic variant sites were interrogated. 15 different probesets were designed, with degeneracy at sites with variants with frequency ≥0.01, giving a total of 123 unique sequences. These were confirmed as having no appreciable homology to the human genome. Each was present in 8 copies on the array, to improve the ability to reliably detect low copy-numbers of HBV DNA.

B. GWAS Grid Selection

Chinese-specific MAFs were estimated for each marker in latest available 1000 genomes content (Phase I) as noted above, using the available data sources. These were allocated to bins corresponding to 0.05≤MAF≤0.50 and 0.01≤MAF<0.05, representing the sets of target markers to be tagged by the GWAS grid. The candidate SNPs available for selection were the full set of CHB/CHS/JPT 1000 genomes content for which Axiom assays could be designed (i.e. taking account of potential nearby interfering variants and/or sequences with appreciable similarity to other regions of the genome).

1. Selection of SNPs to tag 1000 genomes content (Affymetrix)

Using the pre-selected markers as the starting point, the Affymetrix imputation-aware SNP selection procedure was applied, initially targeting the SNPs with MAF≥0.05. Where there was a choice of SNPs to add to the array design, SNPs were prioritised that met one or more of the following criteria:

- Axiom-validated
- Not A/T or G/C allele pairs (i.e. requiring less array 'real estate')
- Present on UK Biobank array

The imputation coverage (proportion of target markers imputable at $r^2 \geq 0.8$) for each chromosome was determined at the end of each phase, and sufficient SNPs were selected and added to the array design such as to achieve 93% coverage for each chromosome. This procedure resulted in the addition of 237,246 additional variants to the final array design.

Despite this high overall coverage, some chromosomal regions remained for which coverage was poor. These were visually identified by randomly sampling 80,000 target markers for each chromosome and plotting their imputation r^2 values against chromosomal coordinate. Coverage in these regions was improved by selecting a further 12,107 SNPs, giving a total of 587,802 markers.

2. Selection of SNPs to tag novel Chinese content (BGI)

The analysis of WGS data available to BGI that provided putative functional variants (see pre-specified content part 6) also identified numerous common and low-frequency SNPs and indels not reported in 1000 genomes CHB/CHS/JPT populations. Coverage of those not already tagged (by 1000 genomes markers that could already be imputed using the currently-selected markers) was achieved using a greedy-tagging procedure with SNP prioritisation on the basis of the same criteria as Affymetrix SNP selection, combined with a score calculated using the sequencing quality scores of tagged and tagging SNPs and the extent to which untagged variants would tag other nearby untagged variants (using pairwise LD calculated from BGI's data).

This was carried out first for tagging of SNPs with MAF≥0.05, with selection of ~60,000 SNPs, and then for tagging of SNPs with 0.01≤MAF<0.05, with selection of ~20,000 SNPs. The final number of additional SNPs selected was 80,370, giving a total of 668,172 markers.

3. Selection of SNPs to increase coverage of low frequency 1000 genomes content (Affymetrix)

To improve imputation coverage of low-frequency variants, the remaining capacity of the array was used for a further 32,529 SNPs, selected using the Affymetrix imputation aware procedure. This gave a total of 700,701 markers.

4. Final QC and array design confirmation

During final array design, a number of markers were identified for which it was not possible to design array probes or for which pairs of probes were sufficiently similar in sequence that it was necessary to exclude one of them. Where this occurred, alternative SNPs were identified where possible (e.g. tag SNPs for known GWAS hits). Otherwise, additional SNPs for low frequency coverage were added. The marker numbers given above reflect the final figures after array design.

C. Array Characteristics

Out of 700,701 markers on the array, 354,399 are also present on the UK Biobank array.

Based on data collected during the array design process, predicted coverage of 1000 genomes CHB/CHS content was as follows:

method	MAF	% r^2 ≥0.8	Mean r^2
imputation	≥0.05	93.0	0.936
imputation	≥0.01	87.1	0.901
imputation	0.01-0.05	68.6	0.788
pairwise	≥0.05	68.9	
pairwise	≥0.01	67.5	
pairwise	0.01-0.05	62.9	

Supplementary Data S2. China Kadoorie Biobank Array Revision. Related to **Figure 2.**

The overall strategy for revision of the CKB array design (summarised in **Figure S3**) was as follows:

- The overall performance of probesets on version 1 of the CKB array was assessed
- Probesets were identified for removal from the design on the basis of:
  - Redundancy (where a variant was interrogated by 2 probesets)
  - Assay failure or low quality
  - Low allele frequency (monomorphic in the first 100 plates of data and absent from other datasets), unless retained for other reasons
- Potential new content was identified including
  - Alternative assays for excluded probesets
  - Tag SNPs for excluded probesets
  - Novel content with putative functional effects
  - New GWAS hits
  - Additional content from collaborators
  - Improvements in/restoration of GWAS grid coverage

Included in this strategy was the use of the full sequencing dataset from the CONVERGE consortium[1], for both content identification and assessment of GWAS grid coverage.

A. Array Version 1 Performance

Two batches of 50 plates underwent standard QC; after exclusion of 5 plates that failed initial QC, a total of 8,995 datasets passed QC, including 98 duplicates. Genotyping of all probesets was carried out, and metrics were derived, using 0.98 as the call rate threshold.

B. Identification of Probesets to be Removed

Using the stated metrics output during genotype calling, SNPs/probesets were flagged for retention, exclusion, or review as follows:

- Retained: All HBV probesets [130 probesets];
- Excluded: Redundant probesets that were not the "preferred" probeset in either batch [57,223 probesets];
- Marked for Review (1): Probesets classed as "PolyHighResolution" or "NoMinorHom" in both batches and included in all "recommended" and "preferred" lists of probesets [585,939 probesets];
- Excluded: Probesets classed as any of "CallRateBelowThreshold", "OffTargetVariant" or "Other" in both batches [39,869 probesets];
- Marked for Review (2): Probesets classed as "MonoHighResolution" in one batch and either "PolyHighResolution" or "NoMinorHom" in the other, and included in all "recommended" and "preferred" lists of probesets [13,251 probesets];
- Marked for Review (3): Probesets classed as "MonoHighResolution" in both batches, and included in all "recommended" and "preferred" lists of probesets [30,628 probesets];
- Marked for Review (4): All hemizygous probesets from MT and chrY [1,162 probesets];

- Excluded: Probesets with a call rate <0.98 in at least one batch [8,570 probesets];
- Excluded: Remaining probesets that were not one of a pair of probesets but were not recommended in both batches [7,989 probesets];
- Excluded: Remaining probesets that in at least one batch were identified as “preferred” probesets but were nevertheless not recommended [5,964 probesets];
- Excluded: Remaining probesets that called one or both batches as “OffTargetVariant”, “CallRateBelowThreshold” or “Other” [8,956 probesets];
- Excluded: Of each remaining pair of probesets, the probeset with the lower overall call rate [7,556 probesets], or if tied the lowest FLD [237 probesets], or if FLD comparison not possible the lowest HomRO [622 probesets];
- Marked for Review (5): Remaining probesets, recommended in one batch but not in the other, which were classed as “MonoHighResolution” in both batches [8,771 probesets];
- Marked for Review (6): Remaining probesets, recommended in one batch but not in the other, classed as “MonoHighResolution” in one batch and either “PolyHighResolution” or “NoMinorHom” in the other [2,246 probesets];
- Marked for Review (7): Remaining probesets, recommended in one batch but not in the other, classed as either “PolyHighResolution” or “NoMinorHom” in both batches [2,824 probesets].

Further review was carried out as follows:

1. Cluster statistics were checked for the reported FLD values. Probesets for which one or both batches had FLD<4.90 were excluded [21,528 probesets];
2. Cluster statistics were checked for the reported FLD and HomRO values. Probesets for which at least one batch had HomRO<0.4 were excluded (all of these were called as MonoHighResolution” in one batch and “PolyHighResolution” in the other) [91 probesets]. Probesets for which the non-monomorphic batch had FLD<4.90 were excluded [294 probesets];
3. These were reviewed together with probesets from (5).
   The frequencies of these SNPs in the CONVERGE dataset were checked. An appreciable number were found to have MAFs in CONVERGE that were sufficiently high that failure to identify any minor alleles was highly unlikely. Probesets corresponding to SNPs with a CONVERGE MAF>0.00158 (corresponding to $P<10^{-6}$; $P<10^{-3}$ even for probesets with 5 minor alleles in the first 2 batches of genotypes) were excluded [1456 probesets].
   Probesets for SNPs that were not found (at whatever frequency) in CONVERGE were excluded unless they were originally included on the array in one of the modules HLA/KIR (193), ApoE (493), Fingerprint (0), Neanderthal (887), LOF (2060), Ax-LOF (1535), GWAS hits (247), novel nonsense (39) [31,907 probesets].
4. Hemizygous SNPs were reanalysed with an updated version of SNPolisher, and treated as follows: (a) exclude duplicate probesets that were not preferred in either batch [65 MT probesets]; (b) exclude probesets for SNPs monomorphic in both batches, Taiwanese data and (for chrY) 1000 genomes CHB/CHS [660 chrY probesets, 62 MT probesets]; (c) examine cluster plots to select between remaining pairs of duplicate probesets, for similar-quality clustering selecting the probeset with higher call rate (or excluding them both) [17 MT probesets].
5. These were reviewed together with probesets from (3), see above.
6. Cluster statistics were checked for the reported FLD and HomRO values. Probesets for which at least one batch had HomRO<0.4 were excluded (all of these were called as

MonoHighResolution" in one batch and "PolyHighResolution" in the other) [16 probesets]. Probesets for which the non-monomorphic batch had FLD<4.90 were excluded [75 probesets];

7. Cluster statistics were checked for the reported FLD values. Probesets for which one or both batches had FLD<4.90 were excluded [282 probesets];

Additional probesets were excluded as follows:

Autosomal SNPs whose minor allele frequency gave an expected minor homozygote count of at least 5 (from review classes 1 and 7) were tested for Hardy-Weinberg disequilibrium. With Holm-Bonferroni multiple testing correction (5% family-wise error rate), probesets with $P<1.13\times10^{-7}$ were excluded [2,734 probesets].

To further check SNPs with low MAF, the total minor allele count was extracted for those polymorphic SNPs still under consideration that were not "PolyHighResolution" in either batch (review classes 2 and 6). There was no obvious excess of SNPs with low minor allele count (1-3 minor alleles). Inspection of selected cluster plots did not indicate any problems.

C. Restoration of selected "monomorphic" SNPs

The list of exclusions was checked for Fingerprint, CKB group, and GWAS hits and these were reviewed (2,410 in total).

7 Fingerprint SNPs restored

55 SNPs specified by the CKB group or collaborators were restored

For GWAS hits:

- Marginal call rate or QC failures were restored [776 variants];
- Variants with lower call rate were restored, but were not used in constructing the GWAS grid [159 variants];
- Monomorphic or near monomorphic probesets that failed initial QC were excluded.

After all exclusions and restorations were complete, 586,528 probesets were retained.

D. Selection of New Content

Novel content was defined as follows (some variants were included for more than one reason):

1. Novel functional content

Coding variants (nonsynonymous, stop gain, etc.) were identified from CONVERGE. These were filtered to remove previously considered variants; this was achieved by excluding SNPs for which no 1000 genomes project frequency information was available. The remaining 72,332 variants were analysed by multiple functional prediction algorithms using TABLE_ANNOVAR. The results from these algorithms were combined to give an average score for whether a variant was deleterious – (sum of deleterious predictions)/(total number of predictions). Predictions were classed as deleterious as follows: SIFT – D=1; Polyphen_HDIV – D=1, P=0.5; LRT – D=1; Mutation_taster – A=1, D=1; Mutation_assessor – H=1, M=0.5; FATHMM – D=1; RadialSVM – D=1; LR_score – D=1.

Variants were selected for inclusion on the array if they had a score ≥0.5 derived from at least 3 algorithms, and had a variant-calling info score ≥0.1. As a result 9,619 variants were identified for addition to the array.

2. *New GWAS hits*

The NHGRI catalogue was downloaded on 28/4/15. There were 7,523 new entries since the list used for the original array design, of which 6,157 were for variants not previously included in the catalogue. Of these, 2,790 were associations at genome-wide significance ($P \leq 5x10^{-8}$), for 1,386 unique variants. 307 of these were already included on the array design, 2 were HLA haplotypes, and 49 were GxG interactions (for which a much higher P-value threshold would be appropriate).

As a result, 1,028 SNPs were identified for addition to the array.

3. *Additional content from collaborators*

Various external and internal collaborators supplied lists of variants, which were checked against the current array content. 292 additional variants were included.

Preliminary analysis of the HBV probes indicated they were successfully identifying HBV infection (strong association with HBV antigen test conducted at baseline). These results informed design of a further 24 HBV probes for inclusion on the array.

4. *Alternative assays/tags for important SNPs*

Key variants, specified by the CKB group and collaborators [15 variants] or which were GWAS hits [431 variants], that failed QC were marked as requiring alternative assays. Where possible, an assay from the opposite strand was designed, otherwise 'tag' SNPs (r^2>0.9) were selected from the Affymetrix library of validated assays.

E. Building of GWAS Grid

The existing GWAS grid was patched and extended using similar procedures to those used during the original array design, with some modifications, as follows:

- Since completion of the original array design, low coverage sequence data from ~9,000 subjects from across China had become available from the CONVERGE Consortium[1]. These were used to update the allele frequency bins used to define the variant target list.
- The variant prioritisation criteria were updated to remove from consideration any variant already excluded from the array design
- Imputation aware variant selection was initially conducted simultaneously for all variants with MAF>0.01, and was halted once coverage of variants with MAF>0.05 reached 94.5% (an improvement on the previous 93%).
- Further greedy tagging of variants not covered by the 1000 genomes reference used both BGI and CONVERGE sequence data.
- Further imputation aware selection was conducted specifically targeting low-frequency variants (0.01≤MAF<0.05) and regions with poor coverage

In total a further 205,176 variants were added to the GWAS grid.

F. Array Characteristics

Out of 803,030 markers on the array, 340,562 are also present on the UK Biobank array.

Predicted coverage of 1000 Genomes CHB/CHS content is as follows:

**method**	**MAF**	**% r^2 ≥0.8**	**Mean r^2**
imputation	≥0.05	93.3	0.942
imputation	≥0.01	85.3	0.900
imputation	0.01-0.05	63.1	0.766
pairwise	≥0.05	73.9	
pairwise	≥0.01	67.5	
pairwise	0.01-0.05	62.3	

Note that there were some changes in the target sets, particularly for the low frequency bin, so these results are not directly comparable to those for the original array design.

1. CONVERGE Consortium (2015). Sparse whole-genome sequencing identifies two loci for major depressive disorder. Nature *523*, 588-591. 10.1038/nature14659.
