# Supplementary material for: Genotyping and population characteristics of the China Kadoorie Biobank
Source: Cell Genom. 2023 Jul 20;3(8):100361. doi: 10.1016/j.xgen.2023.100361 (PMC10435379; doi:10.1016/j.xgen.2023.100361)
Supplement: Document S2. Article plus supplemental information [file mmc13.pdf]

Cell Genomics

Resource

Genotyping and population characteristics of the China Kadoorie Biobank

Graphical abstract

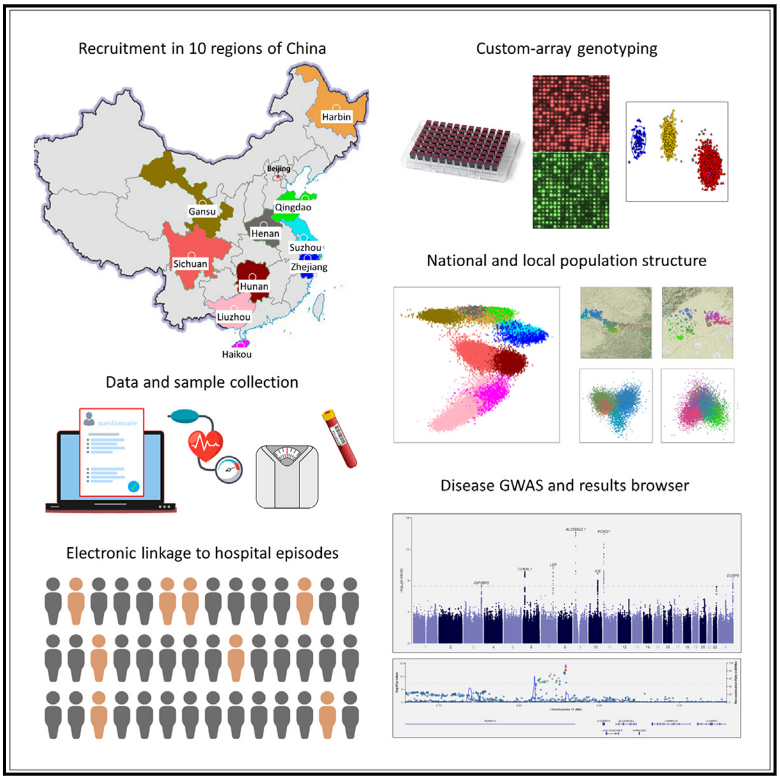

Authors

Robin G. Walters, Iona Y. Millwood, Kuang Lin, ..., Liming Li, Zhengming Chen, China Kadoorie Biobank Collaborative Group

Correspondence

robin.walters@ndph.ox.ac.uk (R.G.W.),
lmleeph@vip.163.com (L.L.),
zhengming.chen@ndph.ox.ac.uk (Z.C.)

In brief

Walters et al. present genetic analyses of >100,000 participants of the China Kadoorie Biobank (CKB), a population-based, prospective cohort of >512,000 from 10 diverse regions of China. They describe how the CKB has contributed to understanding of the genetic basis for many diseases and risk factors and report GWASs of 124 diverse disease outcomes.

Highlights

- The China Kadoorie Biobank is a very large, population-based, prospective cohort
- Comprehensive data and biosamples collected at baseline and during periodic resurveys
- Electronic linkage to death/disease registries and national health insurance system
- Genotyping of >100,000 participants enables GWASs of many diseases and risk factors

Walters et al., 2023, Cell Genomics *3*, 100361
August 9, 2023 © 2023 The Authors.
https://doi.org/10.1016/j.xgen.2023.100361

CellPress

Cell Genomics

CellPress
OPEN ACCESS

Resource

Genotyping and population characteristics of the China Kadoorie Biobank

Robin G. Walters,[1,2,16,17,18,*] Iona Y. Millwood,[1,2,17] Kuang Lin,[1,17] Dan Schmidt Valle,[1] Pandora McDonnell,[1] Alex Hacker,[1] Daniel Avery,[1] Ahmed Edris,[1] Hannah Fry,[1] Na Cai,[3,13] Warren W. Kretzschmar,[3,14] M. Azim Ansari,[4,5] Paul A. Lyons,[6,7] Rory Collins,[1] Peter Donnelly,[3,15] Michael Hill,[1,2] Richard Peto,[1] Hongbing Shen,[8] Xin Jin,[9] Chao Nie,[9] Xun Xu,[9] Yu Guo,[10] Canqing Yu,[11,12] Jun Lv,[11,12] Robert J. Clarke,[1] Liming Li,[11,12,16,*] Zhengming Chen,[1,2,16,*] and China Kadoorie Biobank Collaborative Group

[1]Nuffield Department of Population Health, University of Oxford, Oxford OX3 7LF, UK
[2]MRC Population Health Research Unit, University of Oxford, Oxford OX3 7LF, UK
[3]Wellcome Centre for Human Genetics, University of Oxford, Oxford OX3 7BN, UK
[4]Nuffield Department of Medicine, Oxford University, Oxford OX1 3SY, UK
[5]NIHR Oxford Biomedical Research Centre, Oxford University Hospitals NHS Foundation Trust, Oxford OX3 9DU, UK
[6]Cambridge Institute for Therapeutic Immunology and Infectious Disease, University of Cambridge, Cambridge CB2 0AW, UK
[7]Department of Medicine, University of Cambridge, Cambridge CB2 0QQ, UK
[8]Department of Epidemiology, Collaborative Innovation Center for Cancer Medicine, Nanjing Medical University, Nanjing 211116, China
[9]BGI-Shenzhen, Shenzhen 518083, China
[10]Fuwai Hospital, Chinese Academy of Medical Sciences, Beijing 100037, China
[11]Department of Epidemiology and Biostatistics, School of Public Health, Peking University, Beijing 100191, China
[12]Center for Public Health and Epidemic Preparedness and Response, Peking University, Beijing 100191, China
[13]Present address: Helmholtz Pioneer Campus, Helmholtz Zentrum München, 85764 Neuherberg, Germany
[14]Present address: Vanadis Diagnostics, 191 38 Sollentuna, Sweden
[15]Present address: Genomics plc, Oxford OX1 1JD, UK
[16]Senior author
[17]These authors contributed equally
[18]Lead contact
*Correspondence: robin.walters@ndph.ox.ac.uk (R.G.W.), lmleeph@vip.163.com (L.L.), zhengming.chen@ndph.ox.ac.uk (Z.C.)
https://doi.org/10.1016/j.xgen.2023.100361

SUMMARY

The China Kadoorie Biobank (CKB) is a population-based prospective cohort of >512,000 adults recruited from 2004 to 2008 from 10 geographically diverse regions across China. Detailed data from questionnaires and physical measurements were collected at baseline, with additional measurements at three resurveys involving ~5% of surviving participants. Analyses of genome-wide genotyping, for >100,000 participants using custom-designed Axiom arrays, reveal extensive relatedness, recent consanguinity, and signatures reflecting large-scale population movements from recent Chinese history. Systematic genome-wide association studies of incident disease, captured through electronic linkage to death and disease registries and to the national health insurance system, replicate established disease loci and identify 14 novel disease associations. Together with studies of candidate drug targets and disease risk factors and contributions to international genetics consortia, these demonstrate the breadth, depth, and quality of the CKB data. Ongoing high-throughput omics assays of collected biosamples and planned whole-genome sequencing will further enhance the scientific value of this biobank.

INTRODUCTION

Major non-communicable chronic diseases, such as heart attack, stroke, cancer, and chronic obstructive pulmonary disease (COPD), account for much of the adult disease burden in China and globally. Several such diseases display large unexplained variations in incidence between different regions in China, indicating that important genetic and non-genetic causes remain to be discovered. The China Kadoorie Biobank (CKB) was initiated in 2002, with the goal of investigating the causal relevance of established and novel disease risk factors in the adult Chinese population.[1] From 2004 to 2008, CKB recruited >512,000 adults aged 30–79 years from 10 geographically diverse (five urban, five rural) regions across China, making it one of the largest blood-based prospective biobanks in the world.[2]

Many aspects of the CKB study design support a wide range of hypothesis-driven and hypothesis-free research into many different diseases: population-based recruitment; prospective sample collection; a relatively medication-naive population;

This is an open access article under the CC BY license (http://creativecommons.org/licenses/by/4.0/).

rich and diverse exposure and lifestyle data; and comprehensive capture of incident disease events through electronic linkage to death and disease registries and to health insurance records. The CKB also contributes to the growing demand for ancestrally diverse biobanks, which not only expand opportunities for novel discoveries of potential value to all human populations but also address potential inequalities in healthcare that may arise from the historical focus of research on individuals of European ancestry, findings from which are not necessarily transferable to other populations.[3,4] In common with many other large biobanks, the value of the CKB has been greatly enhanced by large-scale genotyping of study participants. Such genotype information enables investigation of the contribution of genetic variation to phenotype and disease risk, Mendelian randomization (MR) assessment of the causal contribution of risk factors and behaviors to disease, and phenome-wide analyses of the impact of variation at specific loci.

We describe the design and performance of a custom Affymetrix Axiom array optimized for individuals of Chinese Han ancestry, which provides both genome-wide coverage to enable high-quality imputation of both common and low-frequency variation, and direct genotyping of ~68,000 putative loss-of-function, missense, and expression quantitative trait loci (eQTL) variants of potential use for MR or phenome-wide association studies. On the basis of genotyping of >100,000 CKB participants, we demonstrate extensive population diversity across China, identify substantial relatedness within the CKB study population, and observe principal component (PC) signatures consistent with population movements from recent Chinese history. Through linkage to deep phenotyping and >1.2 million recorded disease outcomes in the CKB, this genotyping has already facilitated a wide range of studies, from investigation of ancestry-specific loss of function variants to inform drug target identification, validation, and repurposing[5–8]; to participation in international trans-ancestry genome-wide association study (GWAS) consortia including the Global Biobank Meta-Analysis Initiative (GBMI).[9–14] We report the results of GWASs of 224 disease outcomes, which can be accessed through a CKB PheWeb browser.

STUDY POPULATION AND DATA COLLECTION

Recruitment of CKB participants was community-based, taking place at a large number of local assessment centers within each of 10 diverse regions of China (five rural counties and five urban districts), respectively referred to by the name of the province or city in which recruitment took place. At baseline assessment participants completed an extensive interviewer-administered questionnaire on factors including demographics and socioeconomic status, diet and lifestyle (e.g., smoking, alcohol), physical activity, reproductive history (for women), and medical history and current medication. In addition, all participants underwent physical examination including measurements of anthropometrics, blood pressure, spirometry, exhaled carbon monoxide, and body composition (using bioimpedance). Furthermore, there were onsite blood tests of (non-fasting) glucose and hepatitis B virus (HBV) surface antigen, and blood samples were processed within a few hours to separate plasma and buffy coat for long-term storage.[2]

Subsequent to initial recruitment, three periodic resurveys have been undertaken of approximately 5% of surviving participants, selected on the basis of representative random samples of assessment centers, to provide repeat measurements for correction of regression dilution bias, gather additional questionnaire information, conduct additional physical measurements and blood tests, and collect repeat blood samples and other additional biosamples for long-term storage (Figure 1). The first resurvey of 19,802 participants, conducted immediately after completion of study recruitment in 2008, was largely a repeat of the baseline survey.[2] This was extended in the second resurvey (25,091 participants), conducted in 2013 and 2014, with additional questionnaire data, additional physical measurements, on-site assays of blood lipids, and collection, testing, and storage of urine samples. Additional enhancements in the third resurvey (25,087 participants) in 2020 and 2021 included additional measurements of abdominal ultrasound and retinal imaging, and collection of saliva and stool samples. Baseline characteristics of resurvey participants were similar to those of the overall CKB cohort, with only minor differences attributable to survivor bias (Table S1). More than 22,000 individuals attended at least two resurveys; these multiple measurements at different time points will enable future longitudinal analyses of trajectories of risk factors for major diseases.

In addition to data collected at baseline and the resurveys, an increasing range of data are being generated from assays of stored biosamples. As part of a nested case-control study of stroke and ischemic heart disease (IHD), plasma samples from up to 18,728 participants (all with genotyping) were assayed for 17 clinical biochemistry measurements, with ^{1}H nuclear magnetic resonance (NMR) metabolomics for 4,657; further ^{1}H-NMR metabolomics measurements were conducted for other nested case-control studies of pancreatic cancer and diabetes (2,500 samples to date). Olink proteomics (3,072 proteins) and SomaLogic proteomics (up to 7,000 proteins) have recently been assayed for a further nested case-subcohort study of myocardial infarction (MI; 3,977 participants, all with genotyping), with additional larger scale measurements planned in the near future. Further assays include multiplex serology of antibodies to antigens from 19 pathogens (4,500 samples to date, with measurement in 40,000 cancer cases and controls under way) and ^{1}H-NMR metabolomics of urine samples from 25,251 resurvey participants.

ARRAY DESIGN

CKB genotyping used custom-designed arrays on the Affymetrix (now Thermo Fisher Scientific) Axiom platform, with content selection based on similar overall principles to those used for the UK Biobank array design,[15,16] but adapted to optimize performance for individuals of East Asian ancestry. This addressed three high-level criteria: (1) maximization of genome-wide coverage of common and low-frequency variation in individuals across the whole of China, (2) detection of important variants and of rare loss-of-function and other protein-coding variants that

CellPress
OPEN ACCESS

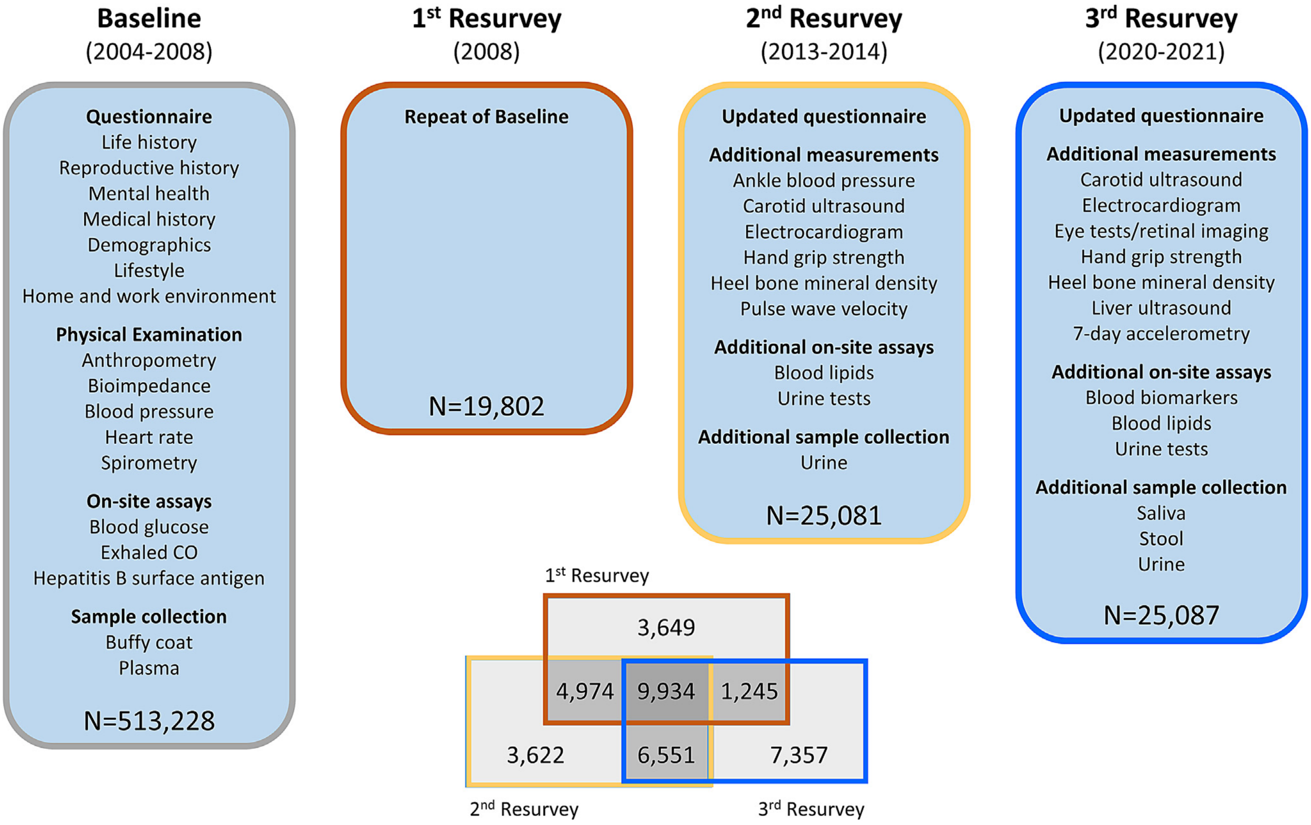

Figure 1. China Kadoorie Biobank (CKB) survey details

Baseline questionnaire content, physical measurements, on-site assays, and biosample collection were repeated at three subsequent resurveys of approximately 5% of surviving participants. Second and third resurveys used updated questionnaires, included additional physical measurements and on-site assays, and collected additional biosamples, as shown. Participants attending more than one resurvey are as indicated in the Venn diagram.
See also Table S1.

are present in Chinese populations, and (3) consistent performance of photolithographic manufacturing processes across array designs and batches of arrays manufactured over an extended time period.[17]

Using the UK Biobank probe list as the starting point for the CKB array design, this was then modified and extended, informed by allele frequency and sequence data for more than 12,000 East Asians that were available to us in 2013 (Data S1; Figure S1). In brief, we (1) removed variants identified as absent or at low frequency in East Asians; (2) added specific variants confirmed as being present in East Asians, including loss-of-function, missense, and eQTL variants; (3) constructed an East Asian-specific genome-wide grid that maximized imputation of both common (>5%) and low-frequency (1%–5%) variants; and (4) included multiple copies of a series of degenerate probes for detection and classification of circulating HBV viral DNA. The resulting array design comprised 781,937 probe sets assaying 700,701 variants, of which 354,399 were also present in the UK Biobank array (Figure S2; Table S2). The design included duplicate probe sets, one for each strand, for 81,236 variants that did not have a validated assay on the Axiom platform.

The initial array design was revised on the basis of genotyping data from the first 100 plates (8,995 samples after quality control [QC]) (Data S2; Figure S3). Failed, poor-quality, or otherwise uninformative monomorphic probe sets were removed, along with the poorer-performing probes of each pair of duplicate probe sets. Further specific content of interest (including further HBV probes and tags for variants that failed QC) were added to the design. Finally, variants were added to improve or restore genome-wide imputation coverage. Figure 2 summarizes the content of the final updated array, comprising 804,496 probe sets assaying 803,030 variants, of which 340,562 are present in the UK Biobank array (Table S3).

GENOTYPING AND QC

Genotyping and QC of a total of 105,408 CKB DNA samples are summarized in Table 1. The initial CKB array design was used to genotype 33,408 samples that had been selected for nested case-control studies of cardiovascular disease and COPD. On the basis of disease follow-up to January 1, 2014 (see below), this initial genotyping included all incident cases of intracerebral hemorrhage (ICH 5,020), subarachnoid hemorrhage (SAH; 455), and fatal IHD (753); randomly selected incident cases of ischemic stroke (IS; 5,662), MI (1,008), and COPD (5,376); participants with no cardiovascular events (n = 10,038) at time of

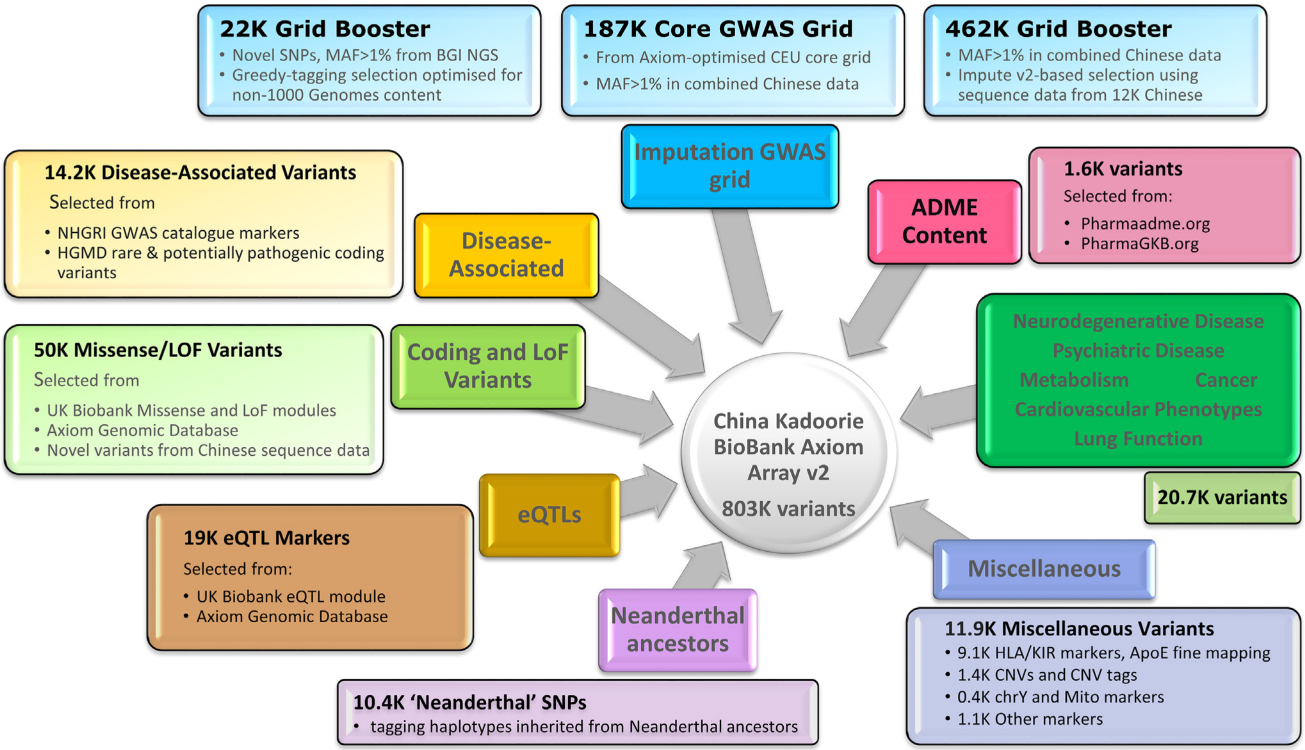

Figure 2. Design of the CKB Axiom genotyping array
The figure illustrates the different categories of content on the revised CKB array. Numbers indicate the approximate counts of variants in each category. Some variants fall into more than one category.
See also Figures S1–S3, Tables S2 and S3, and Data S1 and S2.

selection, matched to ICH cases for sex, age, and region; and 4,766 randomly selected individuals who had attended the second resurvey.

The updated array design was then used to genotype a further 72,000 samples, including all available additional cases of ICH (602), SAH (46), MI (1,028), and fatal IHD (163) that had not previously been genotyped. The remaining genotyped samples came from boxes of DNA samples that were either randomly selected or selected as containing samples derived from the assessment centers used for the second resurvey, in either case being largely representative of the overall CKB cohort. Duplicate samples were present on each pair of consecutive plates to support sample and plate tracking and for other QC purposes.

Genotyping and QC followed the Affymetrix best practice workflow[18] with additional checks for probe sets that displayed substantial between-plate or between-batch differences in allele frequency or call rate. After QC and removal of duplicate probe sets, 76.1% and 85.5% of probe sets remained for the initial and revised arrays, respectively (Table S4); 3.4% of samples failed QC (summarized in Table 1), which were mostly initial QC failures, likely reflecting low quality or low concentration DNA samples; only 0.16% of samples were excluded because of sex mismatch, reflecting the CKB's stringent sample tracking procedures.[19] All pairs of duplicate samples showed high concordance of non-missing genotypes, overall concordance being 99.88% and 99.87% for the first and second array designs respectively (with 0.67% and 0.64% calls missing in one or both of a pair). Where probe sets on the revised CKB array were present in the UK Biobank array, 192 samples genotyped on both arrays yielded concordance of 99.80% (0.46% missing).

The allele frequency distribution for the two datasets reflected the design characteristics of the arrays (Figure 3A). There were many more monomorphic or very low frequency (minor allele frequency [MAF] < 0.0001) variants on the original array design (5.7%) than on the revised array (1.6%) on which many such variants had been removed. Conversely, revision of the array design included selection of additional variants to improve imputation of variants with MAF of 0.01–0.05, and correspondingly more variants passed QC in this MAF range. The allele frequencies of variants that passed QC showed strong agreement with the East Asian populations in the 1000 Genomes reference dataset[20] (Figure 3B), even at lower MAF where estimates in the 1000 Genomes reference were affected by small sample size, and this agreement was consistent across all recruitment regions, although with somewhat greater variation at lower MAF (Figure S4).

These allele frequency data provide some insight into the potential utility of variants included on the CKB array for purposes of investigating the impact of protein loss of function. Variants on the revised array that passed QC were categorized according to their potential functional significance as predicted by Combined

Table 1. Genotyping sample selection and quality control

	Array version 1	Array version 2	Total
Total samples genotyped[a]	33,408	72,000	105,408
Sample ascertainment			
ICH	5,020	602	5,622
SAH	455	46	501
IS	5,662	–	5,662
MI	1,008	1,028	2,036
Fatal IHD	753	163	936
COPD hospitalization	5,358	–	5,358
ICH-matched controls	10,038	–	10,038
Random selection	4,766[b]	69,378[c]	74,144
Intentional duplicates	347	766	1,113
Unintentional duplicates	1	36	37
Total unique samples	33,060	71,198	104,277
QC exclusions			
Failed initial QC	524	2,184	2,708
Call rate <95%	2	0	2
Excess heterozygosity[d]	89	253	342
Excess homozygosity[e]	3	0	3
Sex mismatch	47	121	168
Other linkage error	2	33	35
XY aneuploidy[f]	91	173	264
Ancestry outlier	3	1	4
Consent missing/withdrawn	–	31	31
Samples in current data release	32,300	68,406	100,706

Number of samples genotyped on each genotyping array, showing reasons for selection for genotyping and for quality control exclusion.
[a]Excluding repeats of plate failures.
[b]Random selection of samples from participants attending the second resurvey.
[c]Selected as complete boxes of DNA samples, prioritizing boxes with large numbers of samples from participants eligible for the second resurvey.
[d]More than 3 SDs greater than mean heterozygosity for participants recruited in the same region.
[e]More than 3 SDs less than mean heterozygosity for participants recruited in the same region and with total runs of homozygosity <2 SDs greater than the mean.
[f]Identified as XXY, XY with non-negligible chrX heterozygosity, XXX, X0, or mosaic X0. Some samples failed QC on the basis of >1 criterion.

Annotation Dependent Depletion (CADD version 1.6)[21,22]; this identified 7 classes of variant annotation representing 23,867 variants that had both substantially lower mean MAFs and higher mean Phred values than the other classes, indicating strong enrichment for deleterious variants (Figure 3C). Overall, 37,697 variants had Phred values > 15, corresponding to the top 3% most damaging variants genome-wide, with a high likelihood of pathogenicity.[23] Of these variants, more than half (20,355 [54%]) had MAFs >0.01 in the CKB (28,057 [74%] for CKB MAF > 0.001), of which 5,489 (27%) are virtually absent from European populations (Figure 3D). These will provide opportunities not available in European cohorts for genetic investigations of the importance of the affected genes for disease and disease risk, such as those already conducted for *PLA2G7* and *CETP*.[5,6]

Initially, imputation was performed separately for each array dataset, but the revised array provided only a modest improvement in imputation quality, despite the substantially larger number of informative variants passing QC (Table S4). Therefore, to minimize batch and array effects, we derived a single imputation dataset, using only those variants passing QC in all batches on both array versions (although variants excluded for imputation remain available for analysis in the final dataset). We achieved high-confidence imputation for the large majority of common and low-frequency variants present in the EAS populations of the 1000 Genomes Phase 3 reference (Tables S4 and S5; Figure S5): mean info scores were 0.950 for variants with MAFs > 0.05, 0.849 for MAFs of 0.01–0.05, and 0.695 for MAFs of 0.005–0.01. Imputation was typically poorer for rare variants with MAF <0.005, which are excluded from many analyses.

RELATEDNESS

The community-based recruitment of CKB participants resulted in family groups attending together, so that many individuals had close relatives also recruited into the study (Tables S6 and S7). Among genotyped participants, 31.9% had an also genotyped second-degree or closer relative (23.6% having at least one first-degree relative), with more relatedness in rural than in urban regions (39.0% vs. 22.8% with first-/second-degree relatives), with the exception of participants in Suzhou (54.7%); Suzhou

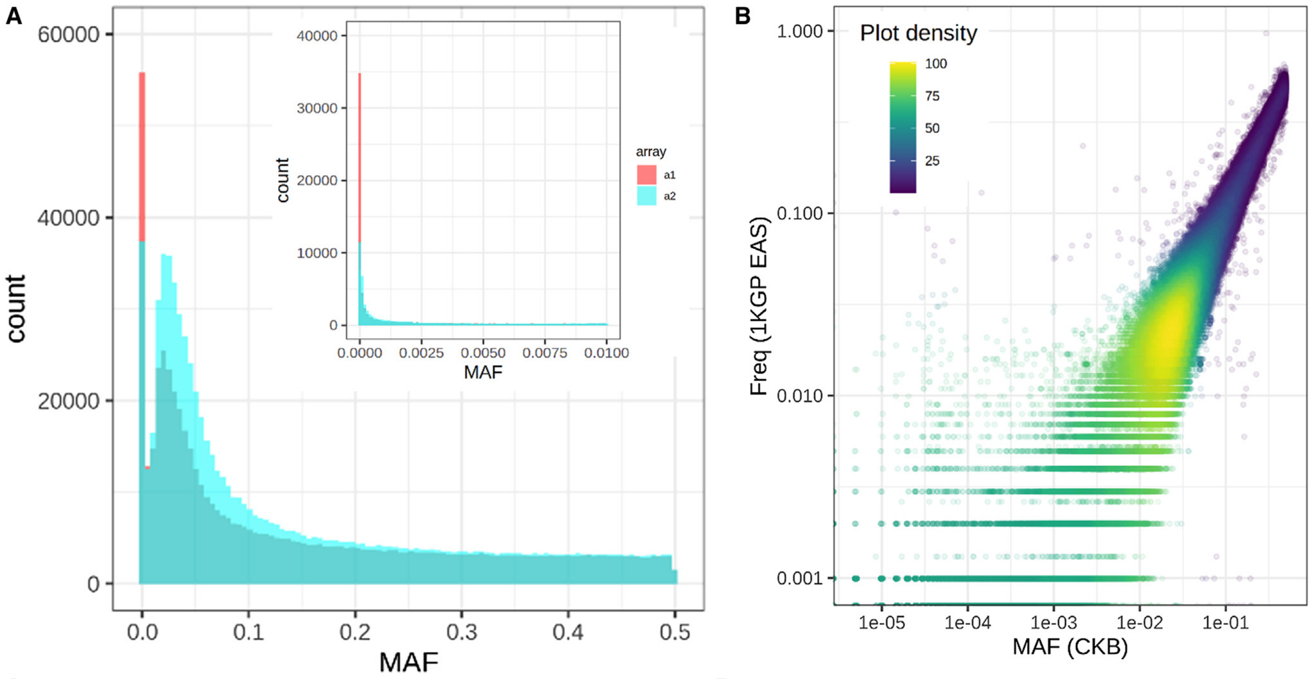

C

Consequence	N	mean MAF	PHRED>15	mean PHRED
STOP_GAINED	656	0.014	656	36.59
CANONICAL_SPLICE	311	0.041	270	26.96
FRAME_SHIFT	86	0.075	73	21.33
NON_SYNONYMOUS	21239	0.075	13075	16.29
INFRAME	46	0.123	20	12.61
SPLICE_SITE	1502	0.083	317	8.60
STOP_LOST	27	0.085	3	7.10
5PRIME_UTR	307	0.137	27	6.10
SYNONYMOUS	4525	0.139	15	4.38
INTRONIC	256116	0.142	5448	3.17
3PRIME_UTR	4681	0.142	193	4.45
INTERGENIC	184939	0.143	3224	2.94
REGULATORY	170861	0.144	10763	4.46
DOWNSTREAM	36549	0.145	1793	3.97
UPSTREAM	34239	0.146	1583	4.00
NONCODING_CHANGE	5224	0.150	237	4.30
**ALL**	**721308**	**0.141**	**37697**	**3.96**

D

PHRED	CKB MAF				
	<0.001	0.001-0.01	0.01-0.05	>0.05	ALL
**ALL**					
>30	1493	434	123	123	2173
15-30	8147	7268	7625	12484	35524
<15	11956	17579	227041	427036	683612
**EUR MAF<0.0001**					
>30	761	363	59	8	1191
15-30	4886	5486	2692	337	13401
<15	2277	7137	56004	12553	77971
**EUR MAF<0.001**					
>30	1163	410	92	24	1689
15-30	6488	6329	4173	1200	18190
<15	3927	8642	97295	37809	147673

Figure 3. Allele frequency and functional annotation of genotyped variants
(A) Allele frequency distribution in unrelated CKB participants of variants passing QC on the two versions of the CKB genotyping array.
(B) Comparison of CKB allele frequency of quality-controlled variants on array version 2 with the corresponding allele in the East Asian subset of the 1000 Genomes Phase 3 reference.
(C) Frequency and characteristics of different classes of quality-controlled variants on array version 2, according to Combined Annotation Dependent Depletion (CADD version 1.6).[21,22]
(D) Allele frequency distribution in CKB and European populations of quality-controlled variants on array version 2, for variants with different levels of predicted functional impact according to CADD.
See also Figure S4.

recruitment took place in a previously rural district that has only recently become urbanized. Suzhou also had a particularly high proportion of individuals with multiple close relatives (1,561 [20%] with $\geq$3 genotyped relatives). Further analysis of relatedness in the CKB also identified 32 pairs of twins, 13,875 individuals with at least 1 sibling (comprising 6,325 family groupings of up to 7 siblings) and 6,571 parent-child relationships, including 1,189 trios.

Several regions displayed patterns of relatedness suggestive of historical consanguinity (Figure S6). Histograms of pairwise

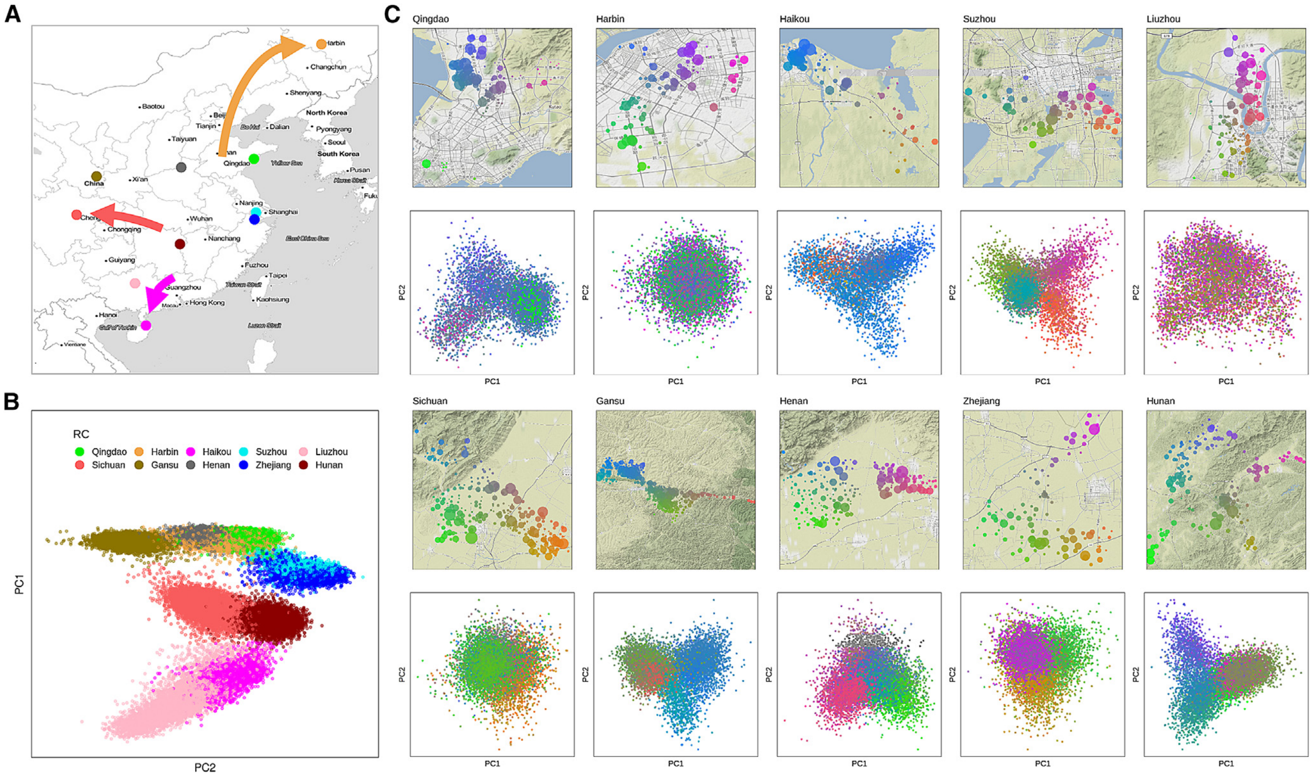

Figure 4. National and local population structure in the CKB

(A) Map of China and adjacent countries showing the locations of the ten CKB regional centers (RCs). Arrows denote known major population movements in recent history that can account for mismatches in the correlation between PCA and geography.

(B) Plot of the two leading principal components from PCA of CKB genotypes, with each participant color-coded according to the RC where they were recruited.

(C) Local maps are shown for each recruitment region, showing the geolocation of individual assessment centers color-coded according to latitude and longitude; the size of the symbol is proportional to the number of genotyped individuals from that center. Corresponding PCA plots show the first two principal components from PCA of individuals from that region, color-coded according to their recruitment center. Top 2 rows, urban regions; bottom 2 rows, rural regions.

See also Figures S9, S10, S12, and S13.

identity by descent (IBD) displayed not only the expected peaks corresponding to integer numbers of meioses separating relatives (at IBDs of 0.5, 0.25, 0.125, etc.), but also other peaks centered on values corresponding to relationships that arise as a result of consanguineous unions between individuals with recent common ancestors, for instance triple second cousins (expected IBD of 0.09375). Consistent with this, 1,050 participants had heterozygosity >3 SDs below the mean values across all genotyped samples, all but 3 of whom had correspondingly extensive runs of homozygosity (Figure S7).

CKB POPULATION STRUCTURE

We performed PC analysis (PCA) of 76,719 unrelated CKB participants and identified that the first 11 PCs were informative for CKB population structure, according to the Bayesian information criterion (BIC) for models predicting individuals' recruitment region (Figure S8). Consistent with findings from many previous studies, individuals formed discrete clusters, whose locations on a plot of the first 2 PCs closely resembled the pattern of longitude and latitude for the regions in which they were recruited (Figures 4A, 4B, and S9). For three regions, however, the positions of their PCA clusters were clearly offset compared with their geographic location. In each case, the apparent discrepancy corresponds to a known major population movement from recent Chinese history: large-scale migration in the 16th and 17th centuries AD from Guangdong to Hainan island; repopulation of the Chengdu Basin in Sichuan in the late 17th and early 18th centuries, a large proportion of migrants coming from Huguang (now Hubei/Hunan); and settlement of largely unpopulated Manchuria in the late 19th and early 20th centuries, with the majority of settlers originating from Shandong province. Thus, the lead PCs reflect the known historical geographic origins of the population in a region, rather than its current physical location.

Although the leading PCs tightly clustered most individuals for each recruitment region, a small proportion (5.7% overall) lay outside the main cluster (>3 SDs from the region mean for PCs 1–11) and thus appeared to have non-local ancestry. Of these, for those with data available from the second resurvey, a high proportion (47.8%) reported that they or at least one parent were born in a different province of China; by comparison, only 12.5% of the remainder reported origins from a province other than that in which they were recruited. The large majority of

participants with non-local ancestry was recruited in Liuzhou, among whom a substantial fraction of individuals (25.4%) lay outside the main PCA cluster, mostly reflecting outlier values for PC 1 (corresponding to the major north-south axis); 87.2% of these individuals reported that either they or a parent was born outside Guangxi province.

Local population structure within each region was not readily apparent from the above pan-China PCA but was clearly observed for individuals within each region-specific cluster (excluding those identified as having non-local ancestry). Between 2 and 9 PCs were informative for the latitude and longitude of the assessment center at which an individual was recruited (Figures 4C, S10, and S11). Rural regions (plus previously rural Suzhou) typically displayed substantial structure, reflecting established communities with little population movement; by contrast, there was only limited population structure for most urban regions, with the exception of Liuzhou, for which an appreciable proportion of second resurvey participants reported having non-Han ancestry (17.6% compared with 1.0% across the other 9 regions). Although uninformative for geographical location within Liuzhou, the first 4 PCs from whole-cohort PCA or the first 2 local PCs were informative for Han status (Figure S12); however, there was no clear discrimination between Han and non-Han that might suggest a need to analyze these individuals separately.

The geographical and/or historical relationships between the different CKB populations are reflected in F_{st} analyses of the genetic distance between regions (Figure S13): the four northern regions cluster together, and also with the northern Han 1000 Genomes population (CHB); the four regions situated on or near the Yangtze River cluster together, in two pairs, and also with the southern Han 1000 Genomes population (CHS); and the southern two regions cluster together, with no appreciable distinction in Liuzhou between Han and non-Han identity. The positions of the 1000 Genomes Project East Asian populations in this analysis indicate that the 10 regions in the CKB population are components of a continuum running from north to south with no clear separation from neighboring countries (KHV, from Vietnam) or ethnic populations (Dai Chinese, from near to the borders with Laos and Myanmar).

DISEASE OUTCOMES

In common with the other biobanks contributing to the GBMI,[14] one of the chief strengths of the CKB is the ability to follow up study participants for a wide range of fatal and non-fatal disease outcomes.[2] In the CKB, disease follow-up is obtained by electronic linkage using participants' unique national identity numbers to registries for death (with cause of death recorded) and for 4 major diseases (stroke, IHD, cancers, diabetes) and to the national health insurance system, which records all inpatient hospital events. These procedures are complemented by active follow-up through annual checks of local residential records and, if necessary, in-person visits by local staff to check key data including vital status and to identify hospitalized episodes in a small proportion of the CKB (currently approximately 2%) who have not joined the health insurance scheme.[24] Data from these multiple sources, including parsing of free-text Chinese language disease descriptions and matching to a clinician-curated disease description library, are integrated and standardized into International Classification of Diseases, 10th Revision (ICD-10), coded incident disease events. By January 1, 2019, >1.2 million incident events and 49,428 deaths (including causes of death) had been recorded for the whole of the CKB, covering >5,000 separate disease types (defined according to three-character ICD-10 code), with only 5,302 (1.0%) of participants being lost to follow-up.

Table 2 shows the number of individuals with selected incident events as recorded through disease follow-up, in all CKB participants and in the genotyped subset. Additional prevalent cases are available through medical questionnaire data, and on-site measurements at baseline (e.g., type 2 diabetes and COPD, through blood glucose assays and spirometry). Reflecting the strategy for selecting the genotyped samples, there was enrichment for cardiovascular diseases, including IS (ICD-10 code I63), intracerebral hemorrhage (ICD-10 code I61), and MI (I21), for COPD (ICD-10 codes J41–J44 and J47), and for all-cause mortality. By contrast, other diseases unrelated to the ascertained case types were present in proportion with the number of genotyped samples.

The wide range of disease outcomes recorded during follow-up is illustrated by the 224 different 3-character ICD-10 codes that have at least 100 incident events recorded in genotyped participants (Table S8). Although limited, this number of cases is sufficient to permit analysis using software packages such as SAIGE,[25] and subsequent contributions to multi-cohort meta-analyses. Work is ongoing to convert the ICD-10 data into Phecodes,[26] to aid harmonization of disease outcomes between CKB and other biobanks and to facilitate phenome-wide association analyses.

ANALYTICAL APPROACH AND GWAS

The historical consanguinity and extensive relatedness in the CKB have been exploited in analysis of the impact of inbreeding on reproductive success[27] and for within-sibship GWASs to derive estimates of direct genetic effects unaffected by genetic nurture.[28] However, for the majority of studies, the population structure of the CKB cohort together with the strategy for selection of samples for genotyping require thoughtful analytical approaches. The substantial relatedness within the CKB, as in many other population-based cohorts, means that exclusion of individuals to avoid inclusion of pairs of close relatives (typically kinship > 0.05, corresponding to third-degree relatives, e.g., first cousins) would result in a substantial reduction in sample size, with some recruitment regions being disproportionately affected (Tables S6, S7, and S9). Therefore, we typically use well-established software packages such as BOLT-LMM[29] and SAIGE[25] that implement linear mixed models to account for both relatedness and population stratification, thereby permitting inclusion of related individuals.

It is unclear, however, that current software packages fully account for all aspects of population structure in the CKB, with its recruitment in 10 discrete regions each with their own distinct genetic characteristics, varying environments, cultures, demographics, and incidence rates of major disease outcomes. For some diseases, this may be not only because they vary in

Table 2. Death and disease events in the CKB

Disease	Definition, ICD-10 codes	All participants	Genotyped
Tuberculosis	A15–A19, J65, K23.0, K67.3, M01.1, M49.0, M90.0, N33.0, N74.0, N74.1	3,053	768
Lung cancer	C33–C34	6,574	1,552
Liver cancer	C22	3,256	665
Stomach cancer	C16	3,771	756
Diabetes	E10–E14	32,748	7,310
MI	I21	7,984	3,386
ICH	I61	11,638	6,663
IS	I63	50,675	14,302
Heart failure	I50	5,160	1,467
Pneumonia	J12–J18	30,339	7,160
Asthma	J45–J46	2,846	880
COPD	J41–J44	20,391	7,971
Liver cirrhosis	K70, K74	2,785	552
Chronic kidney disease	N02–N03, N07, N11, N18	2,879	678
Self-harm	T39.0, T39.1, T39.3, T40, T42, T43, T51, T52, T60, X60–X84, Y87.0, Z91.5	654	114
All hospitalized events	A00–Z99	286,167	60,558
All deaths	A00–Z99, underlying cause	49,428	16,101

Numbers of CKB participants among the whole cohort and the genotyped subset who underwent selected death and disease events during follow-up prior to January 1, 2018. See also Table S8.

prevalence but also because of varying access to healthcare (e.g., in rural compared with urban regions), so that patterns of severity in reported cases may also vary between regions. Therefore, although it is frequently appropriate to conduct analyses across the full genotyped dataset with adjustment for recruitment center (as a 10-level categorical covariate), wherever possible we supplement this with region-stratified analyses (excluding the 5.7% of individuals with non-local ancestry) and meta-analysis, to ensure that association signals are not due to unresolved population stratification or subject to other biases (e.g., arising from heterogeneity between regions).

A second consideration is that the selection for genotyping of nested case-control samples has resulted in substantial over-representation of participants with hospitalization for cardiovascular disease or COPD; although the additional cases were selected on the basis of incident events (i.e., after recruitment), the baseline characteristics of these individuals nevertheless differ from the overall population (e.g., cardiovascular disease events are positively associated with blood pressure, adiposity, blood lipids, smoking, and alcohol consumption). Their inclusion potentially introduces biases or confounding into analyses using the complete genotyping dataset, and we have therefore developed approaches that seek to minimize or eliminate these biases. Where traits are available only in non-random subsets of individuals (e.g., clinical biochemistry measures), we either exclude ascertained cases entirely, include case ascertainment as a covariate, or conduct analyses stratified by ascertainment; note that this is not required for measurements taken at the second resurvey, which was representative of surviving CKB participants. For quantitative traits available for all participants, such as blood pressure or reproductive traits, we perform all adjustments for covariates and data transformations in the full CKB cohort, prior to genetic analyses, so that these adjustments are not distorted by the non-random nature of those genotyped; this is typically performed as a single regression including region as covariates, but we also check the impact of instead performing such adjustments in each region separately. This is the approach used both for contributions to large multi-ancestry meta-analyses[13,30] and for several CKB-specific GWASs.[31,32]

For the analysis of dichotomous variables, to overcome the potential biases due to case enrichment, we have constructed a subset of 77,176 individuals representative of the full CKB cohort in which over-representation of ascertained disease cases was eliminated (STAR Methods; Tables S1 and S9). Analyses of disease outcomes and other binary phenotypes, including contributions to the first round of GBMI studies,[14,33–35] have typically used this population-representative subset supplemented with additional cases from the remainder of the dataset. For these analyses we use SAIGE software,[25] which is designed to account for imbalances in numbers of cases and controls. This approach is combined with region stratification and meta-analysis for diseases with large numbers of cases, so that separate analyses in each region are possible and do not lead to exclusion from analysis of large numbers of variants due to low minor allele count (MAC; see STAR Methods). It should be noted that use of the population subset does not result in a noticeable loss of power, despite the exclusion of ~25% of samples, as there is invariably a large excess of controls even for more common diseases. It also provides a reduced dataset, unaffected by disease ascertainment biases, which can be used for sensitivity analyses of studies of other traits that use the full genotyped dataset.

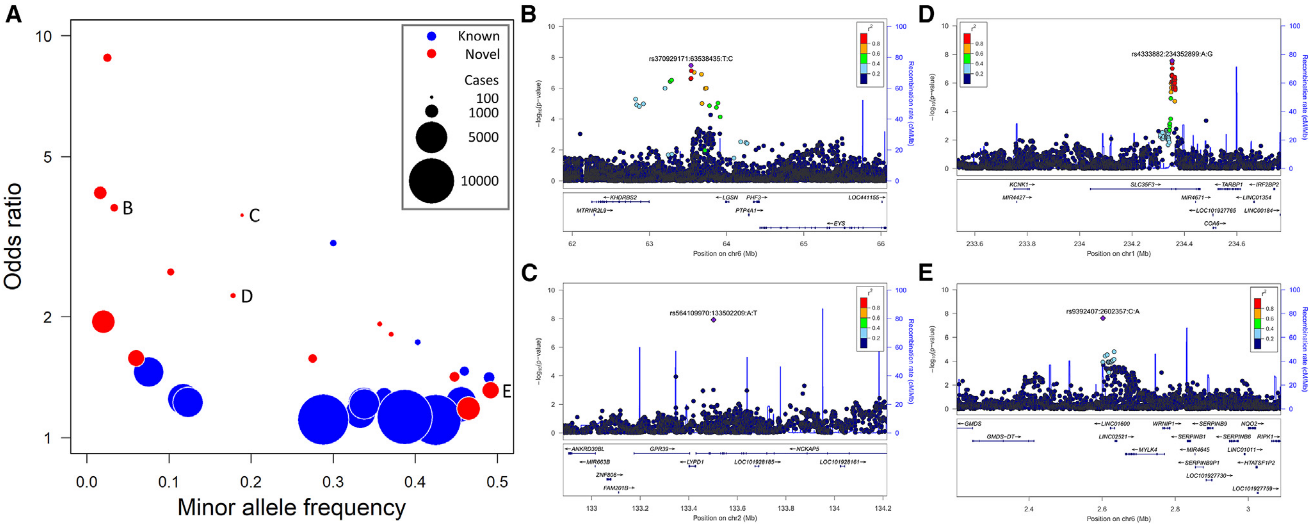

Figure 5. Genome-wide significant associations from GWASs of ICD-10-coded disease events

(A) Summary of the minor allele frequency and the effect size for the risk allele, for all associations with ICD-10-coded outcomes reaching genome-wide significance (5×10^{-8}). Symbols are colored according to whether the association has previously been reported, and are sized in proportion to the number of cases in the corresponding GWAS.

(B–E) Labels denote newly identified associations with (B) H40 (glaucoma), (C) H43 (disorders of vitreous body), (D) K60 (fissure and fistula of anal and rectal regions), and (E) K81 (cholecystitis), illustrated in the corresponding regional association plots, for which there are previously reported associations with related phenotypes or diseases at the same locus. Further plots for these and all other ICD-10 GWASs are available on the CKB PheWeb browser at pheweb.ckbiobank.org.

See also Table S10.

Using this approach, we have conducted GWASs of the ICD-10 codes with at least 100 genotyped cases, yielding 35 associations at genome-wide significance (5×10^{-8}) (Figure 5A; Table S10). The majority of these replicated known association signals for a range of diseases (type 2 diabetes, hypertension, atrial fibrillation, cerebral infarction, gout, liver cirrhosis, liver cancer, lung cancer), but we also identified 14 potentially novel disease-associated loci. Although none of the latter associations survived a strict Bonferroni adjustment to take account of the multiple GWASs, and some were based on a small number of cases, several were nevertheless at loci associated with closely related diseases or phenotypes, suggesting that these reflect robust associations: an association with ICD-10 H40 (glaucoma) near *EYS*, at which there is also an association with retinitis pigmentosa,[36,37] a known risk factor for primary angle-closure glaucoma[38] (Figure 5B); an association with H43 (disorders of vitreous body) at *NCKAP5*, close to reported associations with optic disc size[39] and primary open-angle glaucoma[40] (Figure 5C); an association with K81 (cholecystitis) at *MYLK4*, at which there is an association with serum alkaline phosphatase,[37] an established marker of bile duct stones and acute cholecystitis[41] (Figure 5D); and a variant in *SLC35F3* (rs4333882) associated with K60 (fissure and fistula of anal and rectal regions) and also with one of the causes of fistulas, diverticular disease[42] (Figure 5E). We have made the results and associated plots of all these GWASs available through a PheWeb browser.[43]

RESEARCH CONTRIBUTIONS

In combination with genotyping and imputation, and the wide range of phenotypes and disease outcomes available for CKB participants, the above analytical approaches have been applied in diverse studies. Initial studies, using directly genotyped variants, were MR-based investigations that emphasized the value of ancestry diversity for genetic analyses. In early examples of "drug target MR," we found no association of East Asian-specific variants in *PLA2G7* and *CETP* with major cardiovascular disease outcomes or other major diseases, in each case complementing the results of clinical trials that found no major benefit of drug treatments targeting their respective protein products.[5,6,44] We also exploited the high frequency in East Asians of variants influencing alcohol metabolism, and thereby drinking behavior, to investigate the causal relationship between alcohol consumption and deleterious effects on health: we showed a clear link between alcohol and risk for stroke and, for the first time, we robustly refuted previous reports from observational studies of apparent protective effects of moderate drinking.[45] Other early genetic studies in the CKB investigated the causal relevance of other disease risk factors, providing evidence that vitamin D deficiency increases risk for diabetes and cardiovascular disease,[46,47] that diabetes is itself causally associated with increased risk for cardiovascular disease,[48] and that, although lowering of low-density lipoprotein (LDL) cholesterol decreases risk for IS, it also increases risk for hemorrhagic stroke.[49,50]

Since becoming available, the genome-wide imputed data have enabled a wider range of studies, including MR investigation of further drug targets[7,8] and of diverse disease risk factors such as bone mineral density,[51] gallstone disease,[52] height,[53] blood pressure,[32,54] and resting heart rate.[55,56] We have contributed to replication of novel association signals from large GWASs of blood pressure,[57] menopause,[12] and early-onset

stroke,[58] and have evaluated the performance of polygenic scores in predicting disease risk for lung function,[9,59] lung cancer,[60] fracture,[61] and breast cancer.[62] A comprehensive set of GWASs for major diseases and disease risk factors are in progress, including multiple adiposity and blood pressure traits.[31,32] We also recently published the first large GWAS of lung function in an East Asian population,[63] identifying 48 independent associations of which 18 were novel, once again emphasizing the value of expanding ancestry diversity in genetic studies.

In addition to these analyses conducted primarily within the CKB, we have contributed to many genome-wide association studies in collaboration with major consortia, including trans-ancestry studies of intracranial aneurysm,[10] recurrent miscarriage,[11] blood lipids,[64] fingerprint patterns,[65] diabetes,[66] and height.[13] In particular, CKB has made important contributions to the growing number of studies focused specifically on populations of East Asian ancestry, including the largest single contribution to a GWAS of depression in East Asians[67]; and a major contribution to a GWAS of type 2 diabetes, the largest East Asian GWAS to date.[68] Summary statistics from CKB GWASs have also contributed to the development of methods for genetic association analyses using very low coverage whole-genome sequencing from non-invasive prenatal testing[69]; trans-ancestry colocalization to assess whether two populations share causal variants[70]; and improved genetic discovery in multi-ancestry meta-analyses.[71]

LIMITATIONS OF THE STUDY

Although also one of its strengths, the population-based nature of CKB recruitment leads to certain limitations. First, voluntary participation in the study, although mitigated by very low loss to follow-up, might lead to selection bias, with those recruited potentially being healthier and with fewer health conditions that would reduce likelihood of study participation. In addition, because of recruitment in specific, not necessarily representative locations, care may sometimes be required in extrapolating results from CKB to the Chinese population overall.

Second, although there is near-complete linkage to any episode of hospitalization subsequent to recruitment, data on medical (and family) history is restricted to the limited details recorded during the baseline questionnaire, with no outpatient or primary care data recorded (not covered by the health insurance system). This, together with the middle- to old-age profile of the cohort, means that some categories of disease (e.g., relating to female reproduction) are under-reported or not captured. Although addressed in part by updates to the resurvey questionnaires, this only applies to 5% of participants. Nevertheless, the prospective nature of the cohort and comprehensive linkage ensures that analyses in the CKB can provide reliable assessment of the contribution of genetic and non-genetic factors to major diseases in China.

FUTURE PROSPECTS

Together, these many analyses have established the high-quality linkage between genotyping, data collected at baseline recruitment and resurveys, and disease follow-up. With its breadth of phenotypes and disease outcomes, prospective study design, and growing range of diverse omics assays, CKB will continue to make significant contributions to genetic discovery and elucidation of disease etiology and causality. Ongoing work will further enhance the available genetic resources, including DNA methylation arrays for 982 samples[72]; imputation using the Trans-Omics for Precision Medicine (TopMED)[73] and Westlake Biobank for Chinese (WBBC)[74] reference panels; and whole-genome sequencing of 10,000 participants with incident IS.[75] Whole-genome sequencing of the entire 512,000 cohort is planned in the near future through private-public partnership. Together with other notable biobanks across the world, CKB is addressing the recognized need for ancestrally diverse biobanks, and will continue to make strong contributions to the East Asian and trans-ancestry genetic analyses that are beginning to correct the strong Euro-centric bias of the genetic literature.[4]

STAR★METHODS

Detailed methods are provided in the online version of this paper and include the following:

- KEY RESOURCES TABLE
- RESOURCE AVAILABILITY
  - Lead contact
  - Materials availability
  - Data and code availability
- EXPERIMENTAL MODEL AND SUBJECT DETAILS
  - Study permissions
  - CKB study data
- METHOD DETAILS
  - DNA extraction and SNP genotyping
  - Genome-wide genotyping
  - Variant QC
  - Sample QC
  - Imputation
  - Genetic analyses
  - Analysis subsets
  - Genome-wide association

SUPPLEMENTAL INFORMATION

Supplemental information can be found online at https://doi.org/10.1016/j.xgen.2023.100361.

ACKNOWLEDGMENTS

The most important acknowledgment is to the participants in the study and the members of the survey teams in each of the 10 regional centers and to the project development and management teams based at Beijing, Oxford, and the 10 regional centers. China's National Health Insurance provides electronic linkage to all hospital treatments. We thank Jeanette Schmidt, Teresa Webster, Yontao Lu, and colleagues at Affymetrix (Santa Clara, CA) for genotyping array design; Rory Bowden for helpful discussions during design of HBV probes included on the genotyping array; and Hongcheng Zhou, Haoxiang Lin, Jieqin Liang, and their colleagues at BGI (Shenzhen, China) for DNA extraction, genotyping array design, and genotyping. P.A.L. would like to acknowledge financial support from Versus Arthritis (20593) and the British Heart Foundation (PG/13/64/30435) and to thank Cambridge Genomic Services (Cambridge, UK) for genotyping support, and we acknowledge the European Vasculitis Genetics

Consortium for access to data. The CKB baseline survey and the first resurvey were supported by the Kadoorie Charitable Foundation in Hong Kong. Long-term follow-up was supported by the Wellcome Trust (212946/Z/18/Z, 202922/Z/16/Z, 104085/Z/14/Z, 088158/Z/09/Z), the National Key Research and Development Program of China (2016YFC0900500, 2016YFC0900501, 2016YFC0900504, 2016YFC1303904), and the National Natural Science Foundation of China (81941018, 82192900, 91843302, 91846303). DNA extraction and genotyping was funded by GlaxoSmithKline and the UK Medical Research Council (MC-PC-13049, MC-PC-14135). The project is supported by core funding from the UK Medical Research Council (MC_UU_00017/1, MC_UU_12026/2, MC_U137686851), Cancer Research UK (C16077/A29186; C500/A16896), and the British Heart Foundation (CH/1996001/9454) to the Clinical Trial Service Unit and Epidemiological Studies Unit and to the MRC Population Health Research Unit at Oxford University. Computation used the Oxford Biomedical Research Computing (BMRC) facility, a joint development between the Wellcome Centre for Human Genetics and the Big Data Institute supported by Health Data Research UK and the NIHR Oxford Biomedical Research Centre; the views expressed are those of the authors and not necessarily those of the NHS, the NIHR, or the Department of Health. We are grateful to Ida Surakka and Cristen Willer for helpful comments during drafting of the manuscript, and to Paul Sherliker for assistance with compiling figures in which the key information is accessible to those who are colorblind. This research was funded in whole or in part by the Wellcome Trust (212946/Z/18/Z, 202922/Z/16/Z, 104085/Z/14/Z, 088158/Z/09/Z). For the purpose of open access, the authors have applied a CC-BY public copyright license to any author-accepted manuscript version arising from this submission.

AUTHOR CONTRIBUTIONS

Data collection and analysis, R.G.W., I.Y.M., K.L., D.S.V., P.M., A.H., D.A., A.E., H.F., N.A., W.W.K., M.A.A., P.A.L., X.J., C.N., Y.G., and C.Y.; funding, R.G.W., I.Y.M., R.C., P.D., M.H., R.P., H.S., X.X., R.J.C., L.L., and Z.C.; study oversight, R.G.W., R.C., R.P., J.L., R.J.C., L.L., and Z.C.; manuscript draft and revision, R.G.W., I.Y.M., K.L., R.J.C., and Z.C. All authors reviewed the submitted manuscript.

DECLARATION OF INTERESTS

The authors declare no competing interests.

Received: May 4, 2022
Revised: February 9, 2023
Accepted: June 24, 2023
Published: July 20, 2023

REFERENCES

1. Chen, Z., Lee, L., Chen, J., Collins, R., Wu, F., Guo, Y., Linksted, P., and Peto, R. (2005). Cohort profile: the Kadoorie Study of Chronic Disease in China (KSCDC). Int. J. Epidemiol. *34*, 1243–1249. https://doi.org/10.1093/ije/dyi174.

2. Chen, Z., Chen, J., Collins, R., Guo, Y., Peto, R., Wu, F., and Li, L.; China Kadoorie Biobank CKB collaborative group (2011). China Kadoorie Biobank of 0.5 million people: survey methods, baseline characteristics and long-term follow-up. Int. J. Epidemiol. *40*, 1652–1666. https://doi.org/10.1093/ije/dyr120.

3. Hindorff, L.A., Bonham, V.L., Brody, L.C., Ginoza, M.E.C., Hutter, C.M., Manolio, T.A., and Green, E.D. (2018). Prioritizing diversity in human genomics research. Nat. Rev. Genet. *19*, 175–185. https://doi.org/10.1038/nrg.2017.89.

4. Martin, A.R., Kanai, M., Kamatani, Y., Okada, Y., Neale, B.M., and Daly, M.J. (2019). Clinical use of current polygenic risk scores may exacerbate health disparities. Nat. Genet. *51*, 584–591. https://doi.org/10.1038/s41588-019-0379-x.

5. Millwood, I.Y., Bennett, D.A., Walters, R.G., Clarke, R., Waterworth, D., Johnson, T., Chen, Y., Yang, L., Guo, Y., Bian, Z., et al. (2016). A phenome-wide association study of a lipoprotein-associated phospholipase A2 loss-of-function variant in 90 000 Chinese adults. Int. J. Epidemiol. *45*, 1588–1599. https://doi.org/10.1093/ije/dyw087.

6. Millwood, I.Y., Bennett, D.A., Holmes, M.V., Boxall, R., Guo, Y., Bian, Z., Yang, L., Sansome, S., Chen, Y., Du, H., et al. (2018). Association of CETP Gene Variants With Risk for Vascular and Nonvascular Diseases Among Chinese Adults. JAMA Cardiol. *3*, 34–43. https://doi.org/10.1001/jamacardio.2017.4177.

7. Sliz, E., Kettunen, J., Holmes, M.V., Williams, C.O., Boachie, C., Wang, Q., Männikkö, M., Sebert, S., Walters, R., Lin, K., et al. (2018). Metabolomic Consequences of Genetic Inhibition of PCSK9 Compared With Statin Treatment. Circulation *138*, 2499–2512. https://doi.org/10.1161/circulationaha.118.034942.

8. Bovijn, J., Krebs, K., Chen, C.Y., Boxall, R., Censin, J.C., Ferreira, T., Pulit, S.L., Glastonbury, C.A., Laber, S., Millwood, I.Y., et al. (2020). Evaluating the cardiovascular safety of sclerostin inhibition using evidence from meta-analysis of clinical trials and human genetics. Sci. Transl. Med. *12*, eaay6570. https://doi.org/10.1126/scitranslmed.aay6570.

9. Shrine, N., Guyatt, A.L., Erzurumluoglu, A.M., Jackson, V.E., Hobbs, B.D., Melbourne, C.A., Batini, C., Fawcett, K.A., Song, K., Sakornsakolpat, P., et al. (2019). New genetic signals for lung function highlight pathways and chronic obstructive pulmonary disease associations across multiple ancestries. Nat. Genet. *51*, 481–493. https://doi.org/10.1038/s41588-018-0321-7.

10. Bakker, M.K., van der Spek, R.A.A., van Rheenen, W., Morel, S., Bourcier, R., Hostettler, I.C., Alg, V.S., van Eijk, K.R., Koido, M., Akiyama, M., et al. (2020). Genome-wide association study of intracranial aneurysms identifies 17 risk loci and genetic overlap with clinical risk factors. Nat. Genet. *52*, 1303–1313. https://doi.org/10.1038/s41588-020-00725-7.

11. Laisk, T., Soares, A.L.G., Ferreira, T., Painter, J.N., Censin, J.C., Laber, S., Bacelis, J., Chen, C.Y., Lepamets, M., Lin, K., et al. (2020). The genetic architecture of sporadic and multiple consecutive miscarriage. Nat. Commun. *11*, 5980. https://doi.org/10.1038/s41467-020-19742-5.

12. Ruth, K.S., Day, F.R., Hussain, J., Martínez-Marchal, A., Aiken, C.E., Azad, A., Thompson, D.J., Knoblochova, L., Abe, H., Tarry-Adkins, J.L., et al. (2021). Genetic insights into biological mechanisms governing human ovarian ageing. Nature *596*, 393–397. https://doi.org/10.1038/s41586-021-03779-7.

13. Yengo, L., Vedantam, S., Marouli, E., Sidorenko, J., Bartell, E., Sakaue, S., Graff, M., Eliasen, A.U., Jiang, Y., Raghavan, S., et al. (2022). A saturated map of common genetic variants associated with human height. Nature *610*, 704–712. https://doi.org/10.1038/s41586-022-05275-y.

14. Zhou, W., Kanai, M., Wu, K.-H.H., Rasheed, H., Tsuo, K., Hirbo, J.B., Wang, Y., Bhattacharya, A., Zhao, H., Namba, S., et al. (2022). Global Biobank Meta-analysis Initiative: Powering genetic discovery across human disease. Cell Genom. *2*, 100192. https://doi.org/10.1016/j.xgen.2022.100192.

15. UK Biobank (2014). UK Biobank Axiom Array Content Summary. https://assets.thermofisher.com/TFS-Assets/LSG/brochures/uk_axiom_biobank_contentsummary_brochure.pdf.

16. Bycroft, C., Freeman, C., Petkova, D., Band, G., Elliott, L.T., Sharp, K., Motyer, A., Vukcevic, D., Delaneau, O., O'Connell, J., et al. (2018). The UK Biobank resource with deep phenotyping and genomic data. Nature *562*, 203–209. https://doi.org/10.1038/s41586-018-0579-z.

17. ThermoFisher Scientific (2019). Axiom Precision Medicine Diversity Research Array. https://assets.thermofisher.com/TFS-Assets/GSD/Reference-Materials/axiom-microarray-pmda-datasheet.pdf.

18. Affymetrix (2017). Axiom Genotyping Solution Data Analysis Guide. https://assets.thermofisher.com/TFS-Assets/LSG/manuals/axiom_genotyping_solution_analysis_guide.pdf.

19. Millwood, I.Y., and Walters, R.G. (2020). Collection, Processing, and Management of Biological Samples in Biobank Studies. In Population Biobank

Studies: A Practical Guide, Z. Chen, ed. (Springer), pp. 77–97. https://doi.org/10.1007/978-981-15-7666-9_4.

20. 1000 Genomes Project Consortium; Auton, A., Brooks, L.D., Durbin, R.M., Garrison, E.P., Kang, H.M., Korbel, J.O., Marchini, J.L., McCarthy, S., McVean, G.A., and Abecasis, G.R. (2015). A global reference for human genetic variation. Nature *526*, 68–74. https://doi.org/10.1038/nature15393.

21. Rentzsch, P., Witten, D., Cooper, G.M., Shendure, J., and Kircher, M. (2019). CADD: predicting the deleteriousness of variants throughout the human genome. Nucleic Acids Res. *47*, D886-d894. https://doi.org/10.1093/nar/gky1016.

22. Rentzsch, P., Schubach, M., Shendure, J., and Kircher, M. (2021). CADD-Splice—improving genome-wide variant effect prediction using deep learning-derived splice scores. Genome Med. *13*, 31. https://doi.org/10.1186/s13073-021-00835-9.

23. van der Velde, K.J., de Boer, E.N., van Diemen, C.C., Sikkema-Raddatz, B., Abbott, K.M., Knopperts, A., Franke, L., Sijmons, R.H., de Koning, T.J., Wijmenga, C., et al. (2017). GAVIN: Gene-Aware Variant INterpretation for medical sequencing. Genome Biol. *18*, 6. https://doi.org/10.1186/s13059-016-1141-7.

24. Yang, L., and Chen, Z. (2020). Monitoring Long-Term Health Outcomes of Biobank Participants by Record Linkages. In Population Biobank Studies: A Practical Guide, Z. Chen, ed. (Springer), pp. 99–121. https://doi.org/10.1007/978-981-15-7666-9_5.

25. Zhou, W., Nielsen, J.B., Fritsche, L.G., Dey, R., Gabrielsen, M.E., Wolford, B.N., LeFaive, J., VandeHaar, P., Gagliano, S.A., Gifford, A., et al. (2018). Efficiently controlling for case-control imbalance and sample relatedness in large-scale genetic association studies. Nat. Genet. *50*, 1335–1341. https://doi.org/10.1038/s41588-018-0184-y.

26. Wu, P., Gifford, A., Meng, X., Li, X., Campbell, H., Varley, T., Zhao, J., Carroll, R., Bastarache, L., Denny, J.C., et al. (2019). Mapping ICD-10 and ICD-10-CM Codes to Phecodes: Workflow Development and Initial Evaluation. JMIR Med. Inform. *7*, e14325. https://doi.org/10.2196/14325.

27. Clark, D.W., Okada, Y., Moore, K.H.S., Mason, D., Pirastu, N., Gandin, I., Mattsson, H., Barnes, C.L.K., Lin, K., Zhao, J.H., et al. (2019). Associations of autozygosity with a broad range of human phenotypes. Nat. Commun. *10*, 4957. https://doi.org/10.1038/s41467-019-12283-6.

28. Howe, L.J., Nivard, M.G., Morris, T.T., Hansen, A.F., Rasheed, H., Cho, Y., Chittoor, G., Ahlskog, R., Lind, P.A., Palviainen, T., et al. (2022). Within-sibship genome-wide association analyses decrease bias in estimates of direct genetic effects. Nat. Genet. *54*, 581–592. https://doi.org/10.1038/s41588-022-01062-7.

29. Loh, P.-R., Tucker, G., Bulik-Sullivan, B.K., Vilhjálmsson, B.J., Finucane, H.K., Salem, R.M., Chasman, D.I., Ridker, P.M., Neale, B.M., Berger, B., et al. (2015). Efficient Bayesian mixed-model analysis increases association power in large cohorts. Nat. Genet. *47*, 284–290. https://doi.org/10.1038/ng.3190.

30. Shrine, N., Izquierdo, A.G., Chen, J., Packer, R., Hall, R.J., Guyatt, A.L., Batini, C., Thompson, R.J., Pavuluri, C., Malik, V., et al. (2023). Multi-ancestry genome-wide association analyses improve resolution of genes and pathways influencing lung function and chronic obstructive pulmonary disease risk. Nat. Genet. *55*, 410–422. https://doi.org/10.1038/s41588-023-01314-0.

31. Fairhurst-Hunter, Z., Lin, K., Millwood, I.Y., Pozarickij, A., Chen, T.-T., Torres, J.M., Lun, J.-a., Kartsonaki, C., Gan, W., Mahajan, A., et al. (2022). Trans-ancestry meta-analysis improves performance of genetic scores for multiple adiposity-related traits in East Asian populations. Preprint at medRxiv. https://doi.org/10.1101/2022.07.05.22277254.

32. Pozarickij, A., Gan, W., Lin, K., Clarke, R., Fairhurst-Hunter, Z., Koido, M., Kanai, M., Okada, Y., Kamatani, Y., Guo, Y., et al. (2023). Causal relevance of different blood pressure traits on risk of cardiovascular diseases: GWAS and Mendelian randomisation in 100,000 Chinese adults. Preprint at medRxiv. https://doi.org/10.1101/2023.01.20.23284709.

33. Tsuo, K., Zhou, W., Wang, Y., Kanai, M., Namba, S., Gupta, R., Majara, L., Nkambule, L.L., Morisaki, T., Okada, Y., et al. (2022). Multi-ancestry meta-analysis of asthma identifies novel associations and highlights the value of increased power and diversity. Cell Genom. *2*, 100212. https://doi.org/10.1016/j.xgen.2022.100212.

34. Wu, K.-H.H., Douville, N.J., Konerman, M.C., Mathis, M.R., Hummel, S.L., Wolford, B.N., Surakka, I., Graham, S.E., Joo, H., Hirbo, J., et al. (2021). Polygenic risk score from a multi-ancestry GWAS uncovers susceptibility of heart failure. Preprint at medRxiv. https://doi.org/10.1101/2021.12.06.21267389.

35. Partanen, J.J., Häppölä, P., Zhou, W., Lehisto, A.A., Ainola, M., Sutinen, E., Allen, R.J., Stockwell, A.D., Leavy, O.C., Oldham, J.M., et al. (2022). Leveraging global multi-ancestry meta-analysis in the study of idiopathic pulmonary fibrosis genetics. Cell Genom. *2*, 100181. https://doi.org/10.1016/j.xgen.2022.100181.

36. Mbatchou, J., Barnard, L., Backman, J., Marcketta, A., Kosmicki, J.A., Ziyatdinov, A., Benner, C., O'Dushlaine, C., Barber, M., Boutkov, B., et al. (2021). Computationally efficient whole-genome regression for quantitative and binary traits. Nat. Genet. *53*, 1097–1103. https://doi.org/10.1038/s41588-021-00870-7.

37. Sakaue, S., Kanai, M., Tanigawa, Y., Karjalainen, J., Kurki, M., Koshiba, S., Narita, A., Konuma, T., Yamamoto, K., Akiyama, M., et al. (2021). A cross-population atlas of genetic associations for 220 human phenotypes. Nat. Genet. *53*, 1415–1424. https://doi.org/10.1038/s41588-021-00931-x.

38. Hung, M.C., and Chen, Y.Y. (2022). Association between retinitis pigmentosa and an increased risk of primary angle closure glaucoma: A population-based cohort study. PLoS One *17*, e0274066. https://doi.org/10.1371/journal.pone.0274066.

39. Han, X., Qassim, A., An, J., Marshall, H., Zhou, T., Ong, J.S., Hassall, M.M., Hysi, P.G., Foster, P.J., Khaw, P.T., et al. (2019). Genome-wide association analysis of 95 549 individuals identifies novel loci and genes influencing optic disc morphology. Hum. Mol. Genet. *28*, 3680–3690. https://doi.org/10.1093/hmg/ddz193.

40. Osman, W., Low, S.-K., Takahashi, A., Kubo, M., and Nakamura, Y. (2012). A genome-wide association study in the Japanese population confirms 9p21 and 14q23 as susceptibility loci for primary open angle glaucoma. Hum. Mol. Genet. *21*, 2836–2842. https://doi.org/10.1093/hmg/dds103.

41. Zgheib, H., Wakil, C., Shayya, S., Mailhac, A., Al-Taki, M., El Sayed, M., and Tamim, H. (2019). Utility of liver function tests in acute cholecystitis. Ann. Hepatobiliary. Pancreat. Surg. *23*, 219–227. https://doi.org/10.14701/ahbps.2019.23.3.219.

42. Maguire, L.H., Handelman, S.K., Du, X., Chen, Y., Pers, T.H., and Speliotes, E.K. (2018). Genome-wide association analyses identify 39 new susceptibility loci for diverticular disease. Nat. Genet. *50*, 1359–1365. https://doi.org/10.1038/s41588-018-0203-z.

43. Gagliano Taliun, S.A., VandeHaar, P., Boughton, A.P., Welch, R.P., Taliun, D., Schmidt, E.M., Zhou, W., Nielsen, J.B., Willer, C.J., Lee, S., et al. (2020). Exploring and visualizing large-scale genetic associations by using PheWeb. Nat. Genet. *52*, 550–552. https://doi.org/10.1038/s41588-020-0622-5.

44. Millwood, I.Y., Bennett, D.A., Walters, R.G., Clarke, R., Waterworth, D., Johnson, T., Chen, Y., Yang, L., Guo, Y., Bian, Z., et al. (2016). Lipoprotein-Associated Phospholipase A2 Loss-of-Function Variant and Risk of Vascular Diseases in 90,000 Chinese Adults. J. Am. Coll. Cardiol. *67*, 230–231. https://doi.org/10.1016/j.jacc.2015.10.056.

45. Millwood, I.Y., Walters, R.G., Mei, X.W., Guo, Y., Yang, L., Bian, Z., Bennett, D.A., Chen, Y., Dong, C., Hu, R., et al. (2019). Conventional and genetic evidence on alcohol and vascular disease aetiology: a prospective study of 500 000 men and women in China. Lancet *393*, 1831–1842. https://doi.org/10.1016/s0140-6736(18)31772-0.

46. Lu, L., Bennett, D.A., Millwood, I.Y., Parish, S., McCarthy, M.I., Mahajan, A., Lin, X., Bragg, F., Guo, Y., Holmes, M.V., et al. (2018). Association of vitamin D with risk of type 2 diabetes: A Mendelian randomisation study

in European and Chinese adults. PLoS Med. *15*, e1002566. https://doi.org/10.1371/journal.pmed.1002566.

47. Huang, T., Afzal, S., Yu, C., Guo, Y., Bian, Z., Yang, L., Millwood, I.Y., Walters, R.G., Chen, Y., Chen, N., et al. (2019). Vitamin D and cause-specific vascular disease and mortality: a Mendelian randomisation study involving 99,012 Chinese and 106,911 European adults. BMC Med. *17*, 160. https://doi.org/10.1186/s12916-019-1401-y.

48. Gan, W., Bragg, F., Walters, R.G., Millwood, I.Y., Lin, K., Chen, Y., Guo, Y., Vaucher, J., Bian, Z., Bennett, D., et al. (2019). Genetic Predisposition to Type 2 Diabetes and Risk of Subclinical Atherosclerosis and Cardiovascular Diseases Among 160,000 Chinese Adults. Diabetes *68*, 2155–2164. https://doi.org/10.2337/db19-0224.

49. Sun, L., Clarke, R., Bennett, D., Guo, Y., Walters, R.G., Hill, M., Parish, S., Millwood, I.Y., Bian, Z., Chen, Y., et al. (2019). Causal associations of blood lipids with risk of ischemic stroke and intracerebral hemorrhage in Chinese adults. Nat. Med. *25*, 569–574. https://doi.org/10.1038/s41591-019-0366-x.

50. Falcone, G.J., Kirsch, E., Acosta, J.N., Noche, R.B., Leasure, A., Marini, S., Chung, J., Selim, M., Meschia, J.F., Brown, D.L., et al. (2020). Genetically Elevated LDL Associates with Lower Risk of Intracerebral Hemorrhage. Ann. Neurol. *88*, 56–66. https://doi.org/10.1002/ana.25740.

51. Gan, W., Clarke, R.J., Mahajan, A., Kulohoma, B., Kitajima, H., Robertson, N.R., Rayner, N.W., Walters, R.G., Holmes, M.V., Chen, Z., and McCarthy, M.I. (2017). Bone mineral density and risk of type 2 diabetes and coronary heart disease: A Mendelian randomization study. Wellcome Open Res. *2*, 68. https://doi.org/10.12688/wellcomeopenres.12288.1.

52. Pang, Y., Lv, J., Kartsonaki, C., Guo, Y., Yu, C., Chen, Y., Yang, L., Bian, Z., Millwood, I.Y., Walters, R.G., et al. (2021). Causal effects of gallstone disease on risk of gastrointestinal cancer in Chinese. Br. J. Cancer *124*, 1864–1872. https://doi.org/10.1038/s41416-021-01325-w.

53. Linden, A.B., Clarke, R., Hammami, I., Hopewell, J.C., Guo, Y., Whiteley, W.N., Lin, K., Turnbull, I., Chen, Y., Yu, C., et al. (2022). Genetic associations of adult height with risk of cardioembolic and other subtypes of ischemic stroke: A mendelian randomization study in multiple ancestries. PLoS Med. *19*, e1003967. https://doi.org/10.1371/journal.pmed.1003967.

54. Clarke, R., Wright, N., Walters, R., Gan, W., Guo, Y., Millwood, I.Y., Yang, L., Chen, Y., Lewington, S., Lv, J., et al. (2023). Genetically Predicted Differences in Systolic Blood Pressure and Risk of Cardiovascular and Noncardiovascular Diseases: A Mendelian Randomization Study in Chinese Adults. Hypertension *80*, 566–576. https://doi.org/10.1161/HYPERTENSIONAHA.122.20120.

55. Wang, W., Wang, J., Lv, J., Yu, C., Shao, C., Tang, Y., Guo, Y., Bian, Z., Du, H., Yang, L., et al. (2021). Association of heart rate and diabetes among 0.5 million adults in the China Kadoorie biobank: Results from observational and Mendelian randomization analyses. Nutr. Metab. Cardiovasc. Dis. *31*, 2328–2337. https://doi.org/10.1016/j.numecd.2021.04.015.

56. Huang, T., Wang, W., Wang, J., Lv, J., Yu, C., Guo, Y., Pei, P., Huang, N., Yang, L., Millwood, I.Y., et al. (2022). Conventional and bi-directional genetic evidence on resting heart rate and cardiometabolic traits. J. Clin. Endocrinol. Metab. *107*, e1518–e1527. https://doi.org/10.1210/clinem/dgab847.

57. Takeuchi, F., Akiyama, M., Matoba, N., Katsuya, T., Nakatochi, M., Tabara, Y., Narita, A., Saw, W.-Y., Moon, S., Spracklen, C.N., et al. (2018). Interethnic analyses of blood pressure loci in populations of East Asian and European descent. Nat. Commun. *9*, 5052. https://doi.org/10.1038/s41467-018-07345-0.

58. Jaworek, T., Xu, H., Gaynor, B.J., Cole, J.W., Rannikmae, K., Stanne, T.M., Tomppo, L., Abedi, V., Amouyel, P., Armstrong, N.D., et al. (2022). Contribution of Common Genetic Variants to Risk of Early-Onset Ischemic Stroke. Neurology *99*, e1738–e1754. https://doi.org/10.1212/WNL.0000000000201006.

59. Wain, L.V., Shrine, N., Artigas, M.S., Erzurumluoglu, A.M., Noyvert, B., Bossini-Castillo, L., Obeidat, M., Henry, A.P., Portelli, M.A., Hall, R.J., et al. (2017). Genome-wide association analyses for lung function and chronic obstructive pulmonary disease identify new loci and potential druggable targets. Nat. Genet. *49*, 416–425. https://doi.org/10.1038/ng.3787.

60. Dai, J., Lv, J., Zhu, M., Wang, Y., Qin, N., Ma, H., He, Y.Q., Zhang, R., Tan, W., Fan, J., et al. (2019). Identification of risk loci and a polygenic risk score for lung cancer: a large-scale prospective cohort study in Chinese populations. Lancet Respir. Med. *7*, 881–891. https://doi.org/10.1016/s2213-2600(19)30144-4.

61. Lu, T., Forgetta, V., Keller-Baruch, J., Nethander, M., Bennett, D., Forest, M., Bhatnagar, S., Walters, R.G., Lin, K., Chen, Z., et al. (2021). Improved prediction of fracture risk leveraging a genome-wide polygenic risk score. Genome Med. *13*, 16. https://doi.org/10.1186/s13073-021-00838-6.

62. Ho, W.K., Tai, M.C., Dennis, J., Shu, X., Li, J., Ho, P.J., Millwood, I.Y., Lin, K., Jee, Y.H., Lee, S.H., et al. (2022). Polygenic risk scores for prediction of breast cancer risk in Asian populations. Genet. Med. *24*, 586–600. https://doi.org/10.1016/j.gim.2021.11.008.

63. Zhu, Z., Li, J., Si, J., Ma, B., Shi, H., Lv, J., Cao, W., Guo, Y., Millwood, I.Y., Walters, R.G., et al. (2021). A large-scale genome-wide association analysis of lung function in the Chinese population identifies novel loci and highlights shared genetic aetiology with obesity. Eur. Respir. J. *58*, 2100199. https://doi.org/10.1183/13993003.00199-2021.

64. Graham, S.E., Clarke, S.L., Wu, K.-H.H., Kanoni, S., Zajac, G.J.M., Ramdas, S., Surakka, I., Ntalla, I., Vedantam, S., Winkler, T.W., et al. (2021). The power of genetic diversity in genome-wide association studies of lipids. Nature *600*, 675–679. https://doi.org/10.1038/s41586-021-04064-3.

65. Li, J., Glover, J.D., Zhang, H., Peng, M., Tan, J., Mallick, C.B., Hou, D., Yang, Y., Wu, S., Liu, Y., et al. (2022). Limb development genes underlie variation in human fingerprint patterns. Cell *185*, 95–112.e18. https://doi.org/10.1016/j.cell.2021.12.008.

66. Mahajan, A., Spracklen, C.N., Zhang, W., Ng, M.C.Y., Petty, L.E., Kitajima, H., Yu, G.Z., Rüeger, S., Speidel, L., Kim, Y.J., et al. (2022). Multi-ancestry genetic study of type 2 diabetes highlights the power of diverse populations for discovery and translation. Nat. Genet. *54*, 560–572. https://doi.org/10.1038/s41588-022-01058-3.

67. Giannakopoulou, O., Lin, K., Meng, X., Su, M.H., Kuo, P.H., Peterson, R.E., Awasthi, S., Moscati, A., Coleman, J.R.I., Bass, N., et al. (2021). The Genetic Architecture of Depression in Individuals of East Asian Ancestry: A Genome-Wide Association Study. JAMA Psychiatr. *78*, 1258–1269. https://doi.org/10.1001/jamapsychiatry.2021.2099.

68. Spracklen, C.N., Horikoshi, M., Kim, Y.J., Lin, K., Bragg, F., Moon, S., Suzuki, K., Tam, C.H.T., Tabara, Y., Kwak, S.H., et al. (2020). Identification of type 2 diabetes loci in 433,540 East Asian individuals. Nature *582*, 240–245. https://doi.org/10.1038/s41586-020-2263-3.

69. Liu, S., Huang, S., Chen, F., Zhao, L., Yuan, Y., Francis, S.S., Fang, L., Li, Z., Lin, L., Liu, R., et al. (2018). Genomic Analyses from Non-invasive Prenatal Testing Reveal Genetic Associations, Patterns of Viral Infections, and Chinese Population History. Cell *175*, 347–359.e14. https://doi.org/10.1016/j.cell.2018.08.016.

70. Kuchenbaecker, K., Telkar, N., Reiker, T., Walters, R.G., Lin, K., Eriksson, A., Gurdasani, D., Gilly, A., Southam, L., Tsafantakis, E., et al. (2019). The transferability of lipid loci across African, Asian and European cohorts. Nat. Commun. *10*, 4330. https://doi.org/10.1038/s41467-019-12026-7.

71. Turley, P., Martin, A.R., Goldman, G., Li, H., Kanai, M., Walters, R.K., Jala, J.B., Lin, K., Millwood, I.Y., Carey, C.E., et al. (2021). Multi-Ancestry Meta-Analysis yields novel genetic discoveries and ancestry-specific associations. bioRxiv. https://doi.org/10.1101/2021.04.23.441003.

72. Si, J., Yang, S., Sun, D., Yu, C., Guo, Y., Lin, Y., Millwood, I.Y., Walters, R.G., Yang, L., Chen, Y., et al. (2021). Epigenome-wide analysis of DNA methylation and coronary heart disease: a nested case-control study. Elife *10*, e68671. https://doi.org/10.7554/eLife.68671.

73. Taliun, D., Harris, D.N., Kessler, M.D., Carlson, J., Szpiech, Z.A., Torres, R., Taliun, S.A.G., Corvelo, A., Gogarten, S.M., Kang, H.M., et al. (2021). Sequencing of 53,831 diverse genomes from the NHLBI TOPMed Program. Nature *590*, 290–299. https://doi.org/10.1038/s41586-021-03205-y.

74. Zhu, X.-W., Liu, K.-Q., Wang, P.-Y., Liu, J.-Q., Chen, J.-Y., Xu, X.-J., Xu, J.-J., Qiu, M.-C., Sun, Y., Liu, C., et al. (2021). Cohort profile: the Westlake BioBank for Chinese (WBBC) pilot project. BMJ Open *11*, e045564. https://doi.org/10.1136/bmjopen-2020-045564.

75. Yu, C., Lan, X., Tao, Y., Guo, Y., Sun, D., Qian, P., Zhou, Y., Walters, R., Li, L., Millwood, I., et al. (2022). A High-resolution Haplotype-resolved Reference Panel Constructed from the China Kadoorie Biobank Study. medRxiv. https://doi.org/10.1101/2022.12.14.22283491.

76. Sollis, E., Mosaku, A., Abid, A., Buniello, A., Cerezo, M., Gil, L., Groza, T., Güneş, O., Hall, P., Hayhurst, J., et al. (2023). The NHGRI-EBI GWAS Catalog: knowledgebase and deposition resource. Nucleic Acids Res. *51*, D977-d985. https://doi.org/10.1093/nar/gkac1010.

77. Elsworth, B., Lyon, M., Alexander, T., Liu, Y., Matthews, P., Hallett, J., Bates, P., Palmer, T., Haberland, V., Smith, G.D., et al. (2020). The MRC IEU OpenGWAS data infrastructure. Preprint at bioRxiv. https://doi.org/10.1101/2020.08.10.244293.

78. Landrum, M.J., Lee, J.M., Benson, M., Brown, G.R., Chao, C., Chitipiralla, S., Gu, B., Hart, J., Hoffman, D., Jang, W., et al. (2018). ClinVar: improving access to variant interpretations and supporting evidence. Nucleic Acids Res. *46*, D1062-d1067. https://doi.org/10.1093/nar/gkx1153.

79. Arnold, M., Raffler, J., Pfeufer, A., Suhre, K., and Kastenmüller, G. (2015). SNiPA: an interactive, genetic variant-centered annotation browser. Bioinformatics *31*, 1334–1336. https://doi.org/10.1093/bioinformatics/btu779.

80. Chang, C.C., Chow, C.C., Tellier, L.C., Vattikuti, S., Purcell, S.M., and Lee, J.J. (2015). Second-generation PLINK: rising to the challenge of larger and richer datasets. GigaScience *4*, 7. https://doi.org/10.1186/s13742-015-0047-8.

81. O'Connell, J., Sharp, K., Shrine, N., Wain, L., Hall, I., Tobin, M., Zagury, J.-F., Delaneau, O., and Marchini, J. (2016). Haplotype estimation for biobank-scale data sets. Nat. Genet. *48*, 817–820. https://doi.org/10.1038/ng.3583.

82. Phan, L., Jin, Y., Zhang, H., Qiang, W., Shekhtman, E., Shao, D., Revoe, D., Villamarin, R., Ivanchenko, E., Kimura, M., et al. (2020). ALFA: Allele Frequency Aggregator. www.ncbi.nlm.nih.gov/snp/docs/gsr/alfa/.

83. Abraham, G., Qiu, Y., and Inouye, M. (2017). FlashPCA2: principal component analysis of Biobank-scale genotype datasets. Bioinformatics *33*, 2776–2778. https://doi.org/10.1093/bioinformatics/btx299.

84. Kahle, D., and Wickham, H. (2013). ggmap: Spatial Visualization with ggplot2. R J. 5, 144–161.

85. Pruim, R.J., Welch, R.P., Sanna, S., Teslovich, T.M., Chines, P.S., Gliedt, T.P., Boehnke, M., Abecasis, G.R., and Willer, C.J. (2010). LocusZoom: regional visualization of genome-wide association scan results. Bioinformatics *26*, 2336–2337. https://doi.org/10.1093/bioinformatics/btq419.

86. Lancaster, G., Gilbert, S., and Yang, X. (2020). Development and Application of IT Systems in Biobank Studies. In Population Biobank Studies: A Practical Guide, Z. Chen, ed. (Springer), pp. 145–169. https://doi.org/10.1007/978-981-15-7666-9_7.

87. Sansome, G., and Hacker, A. (2020). Management and Curation of Multi-Dimensional Data in Biobank Studies. In Population Biobank Studies: A Practical Guide, Z. Chen, ed. (Springer), pp. 171–202. https://doi.org/10.1007/978-981-15-7666-9_8.

88. Illumina (2010). GoldenGate Genotyping Assay Guide. https://support.illumina.com/content/dam/illumina-support/documents/documentation/chemistry_documentation/arraykits/goldengate/GoldenGate_Genotyping_Assay_Guide_15004065_B.pdf.

89. Affymetrix (2010). Axiom Genotyping Assay. https://www.affymetrix.com/support/downloads/manuals/axiom_assay_user_manual.pdf.

90. Lyons, P.A., Peters, J.E., Alberici, F., Liley, J., Coulson, R.M.R., Astle, W., Baldini, C., Bonatti, F., Cid, M.C., Elding, H., et al. (2019). Genome-wide association study of eosinophilic granulomatosis with polyangiitis reveals genomic loci stratified by ANCA status. Nat. Commun. *10*, 5120. https://doi.org/10.1038/s41467-019-12515-9.

91. Price, A.L., Weale, M.E., Patterson, N., Myers, S.R., Need, A.C., Shianna, K.V., Ge, D., Rotter, J.I., Torres, E., Taylor, K.D., et al. (2008). Long-range LD can confound genome scans in admixed populations. Am. J. Hum. Genet. *83*, 132–135. https://doi.org/10.1016/j.ajhg.2008.06.005.

92. Affymetrix (2013). Axiom CNV Summary Tool. http://www.affymetrix.com/support/downloads/manuals/axiom_cnv_summary_tool_usermanual.pdf.

93. Delaneau, O., Marchini, J., 1000 Genomes Project Consortium; 1000 Genomes Project Consortium; Lunter, G., Marchini, J.L., Myers, S., Gupta-Hinch, A., Iqbal, Z., Mathieson, I., et al. (2014). Integrating sequence and array data to create an improved 1000 Genomes Project haplotype reference panel. Nat. Commun. *5*, 3934. https://doi.org/10.1038/ncomms4934.

94. Howie, B.N., Donnelly, P., and Marchini, J. (2009). A Flexible and Accurate Genotype Imputation Method for the Next Generation of Genome-Wide Association Studies. PLoS Genet. *5*, e1000529. https://doi.org/10.1371/journal.pgen.1000529.

95. Privé, F., Luu, K., Blum, M.G.B., McGrath, J.J., and Vilhjálmsson, B.J. (2020). Efficient toolkit implementing best practices for principal component analysis of population genetic data. Bioinformatics *36*, 4449–4457. https://doi.org/10.1093/bioinformatics/btaa520.

96. Venables, B., and Ripley, B.D. (2002). Modern Applied Statistics with S (Springer). https://doi.org/10.1007/978-0-387-21706-2.

STAR★METHODS

KEY RESOURCES TABLE

REAGENT or RESOURCE	SOURCE	IDENTIFIER
Critical commercial assays		
Golden Gate genotyping	Illumina	https://www.illumina.com/documents/products/technotes/technote_veracode_goldengate_genotyping.pdf
CKB_1/CKB_2 Axiom® arrays	ThermoFisher	Array manifests not publicly available, provided on application
Deposited data		
Association results	This paper	https://pheweb.ckbiobank.org
Association results	This paper	GWAS Catalog accessions GCST90246012-GCST90246229, http://ftp.ebi.ac.uk/pub/databases/gwas/summary_statistics/GCST90246001-GCST90247000/
1000 Genomes	1000 Genomes Project Consortium[20]	http://ftp.1000genomes.ebi.ac.uk/vol1/ftp/release/20130502/
GWAS Catalog	Sollis et al.[76]	https://www.ebi.ac.uk/gwas/
OpenGWAS	Elsworth et al.[77]	https://gwas.mrcieu.ac.uk/
ClinVar	Landrum et al.[78]	https://www.ncbi.nlm.nih.gov/clinvar/
Map data	OpenStreetMap	https://www.openstreetmap.org
Map tiles	Stamen	maps.stamen.com
EAS recombination rates	Arnold et al.[79]	http://snipa.helmholtz-muenchen.de/snipa/
Software and algorithms		
PLINK	Chang et al.[80]	https://www.cog-genomics.org/plink/
SHAPEIT3	O'Connell et al.[81]	https://jmarchini.org/software/
IMPUTE4	Bycroft et al.[16]	https://jmarchini.org/software/
CADD	Rentzsch et al.[22]	https://cadd.gs.washington.edu/
ALFA	Phan et al.[82]	https://www.ncbi.nlm.nih.gov/snp/docs/gsr/alfa/
FlashPCA	Abraham et al.[83]	https://github.com/gabraham/flashpca
Ggmap	Kahle et al.[84]	https://github.com/dkahle/ggmap
SAIGE	Zhou et al.[25]	https://github.com/weizhouUMICH/SAIGE
LocusZoom	Pruim et al.[85]	https://github.com/statgen/locuszoom-standalone

RESOURCE AVAILABILITY

Lead contact

Further information and requests for resources and reagents should be directed to and will be fulfilled by the lead contact, Robin Walters (robin.walters@ndph.ox.ac.uk).

Materials availability

There are restrictions on the availability of extracted DNA due to the Administrative Regulations on Human Genetic Resources of the People's Republic of China.

Data and code availability

- Data from baseline, first and second resurveys, and disease follow-up are available under the CKB Open Access Data Policy to bona fide researchers. Full details of the CKB Data Sharing Policy are available at www.ckbiobank.org.
- Sharing of genotyping data is currently constrained by the Administrative Regulations on Human Genetic Resources of the People's Republic of China. Access to these and certain other data is available through collaboration with CKB researchers.
- GWAS summary statistics are available at https://pheweb.ckbiobank.org and have been deposited at GWAS Catalog, and are publicly available as of the date of publication. Accession numbers are listed in the key resources table.
- The paper does not report original code.

EXPERIMENTAL MODEL AND SUBJECT DETAILS

Study permissions

All participants provided written informed consent at each survey visit, allowing access to their medical records and long-term storage of biosamples for future unspecified medical research purposes, without any feedback of results to the individuals concerned. Ethical approval was obtained from the Oxford Tropical Research Ethics Committee, the Ethical Review Committees of the Chinese Center for Disease Control and Prevention, Chinese Academy of Medical Sciences, and the Institutional Review Board (IRB) at Peking University. The Chinese Ministry of Health approved the study at the start in 2004 (including export of plasma samples to Oxford), and also approved electronic linkage to health insurance records in 2011. Raw genotyping data were exported from China to the Oxford CKB International Coordinating Center under Data Export Approvals 2014-13 and 2015-39 from the Office of Chinese Human Genetic Resource Administration.

CKB study data

Full details of the CKB study design and methods have been previously reported.[2] Briefly, 512,726 adults aged 30–79 years were enrolled during 2004–2008 from ten urban and rural areas across China. At local study assessment centers, trained health workers administered a laptop-based questionnaire; undertook physical measurements; and collected a blood sample for long-term storage and onsite blood tests. Three subsequent resurveys of ~5% randomly selected surviving participants were conducted using similar procedures in 2008, 2013–2014, and 2020–2021. With the exception of genomics data, all CKB survey data were collected and stored using bespoke IT systems and databases tailored to CKB requirements.[86,87] Disease follow-up data from death and disease registries and from health insurance records were processed and matched to study identifiers by local staff in each recruitment region, centrally processed and converted into ICD-10-coded events, and integrated into the main study database. The database is regularly processed into research-ready snapshots from which datasets are served to researchers. All results are based on CKB data release version 17.02, incorporating disease follow-up up to 1 January 2019.

METHOD DETAILS

DNA extraction and SNP genotyping

DNA extraction and genotyping was performed at BGI, Shenzhen, China, using KingFisher™ Blood DNA Kit and KingFisher™ Flex 24 Magnetic Particle Processors (Thermo Scientific), yielding 400μL DNA at 220 ng/L mean concentration. Buffy coat sample tubes were barcode scanned, and up to 800μL was manually pipetted into the extraction tube at the positions specified by a bespoke sample-tracking IT system. Extracted DNA was transferred using a Freedom EVO® (Tecan) fluid handling system, up to 200μL to each of two sets of 96-tube racks of 2D-barcoded cryovials (Fluidx, Azenta Life Sciences), and 12μL to a 96-well microtitre plate. DNA concentration and quality was recorded using a NanoDrop Microvolume Spectrophotometer (Thermo Scientific). Tubes were frozen and shipped on dry ice to the CKB sample storage facility in Beijing, for long-term storage at −70C. All sample movements were recorded by the sample-tracking IT system.

The first 95,680 DNA samples (from randomly-selected participants), extracted during 2012–2013, were genotyped using the multiplex Golden Gate® platform (Illumina), for panels of 384 variants which included 3 variants informative for sex within the chromosome XY pseudoautosomal regions. SNP genotyping was performed in 96-well microtitre plates, and used up to 5μL DNA from the microtitre plate produced during DNA extraction. Each genotype plate included positive and negative controls at fixed positions, and 2 pairs of duplicate samples at unique combinations of plate positions. Genotyping was performed for a total of 1,040 plates according to GoldenGate Genotyping Assay Manual Protocols,[88] with beadchip imaging using an iScan System (Illumina). The 384 SNP panel was revised after genotyping of the first 100 plates (9200 unique samples), and again after the second 100 plates.

Data processing, genotype calling, and quality control was conducted at CTSU, University of Oxford, UK. Genotyping calling was performed in 4 batches using GenomeStudio software, with initial QC based on automated clustering. All negative controls had an SNP call rate of 80% or less (mean = 34%). 15 plates were flagged for inspection due to an initial positive control call rate <95%, but no failures of genotyping were identified; the remaining positive controls had mean call rate of 98.6%. A further 12 plates were flagged for inspection due to 1 or both of 12 pairs of duplicates being among 709 samples excluded with call rate <90%, but again no failures of genotyping were identified.

Following this initial QC, and again after final sample QC, SNPs were reclustered, and within each batch SNPs with GenTrain score <0.7 were inspected manually, and manually reclustered or excluded as appropriate. Across the 3 SNP panels, 30 SNP assays failed genotyping within that panel (either due to gross genotyping failure or call rate <95%), and a further 42 SNP assays failed for a subset of the 4 'plexes'. Two SNPs displayed Hardy-Weinberg disequilibrium due to presumed assay interference by nearby SNVs or indels. 15 SNPs displayed potential batch effects, identifying genotype clustering errors that were adjusted manually.

Following SNP QC, an additional 1,518 unique samples (2,217 in total) were excluded on the basis of an SNP call rate <98%. One sample with excess heterozygosity (F-statistic >5 SDs above the mean) was excluded. For 2,063 remaining pairs of duplicate samples, genotyping concordance was between 98.66% and 100% (mean 99.98%), and only 118 samples (0.1%) were identified with mismatches of reported gender and inferred sex based on 3 sex-informative SNPs from chrXY pseudoautosomal regions, confirming

good DNA quality and robust linkage to originating study participants. A further 136 samples (0.1%) from blocks with multiple sex-mismatches or with other potential sample linkage errors were also excluded.

Genome-wide genotyping

For genotyping using the first version of the CKB array, samples were selected for genotyping as part of nested case-control or case-cohort study designs. Incident cardiovascular disease cases were selected according to available disease follow-up at time of sample selection (August 2014) from amongst those with extracted DNA and no self-reported prior cardiovascular disease history, as follows: (a) all cases of intracerebral haemorrhage (ICH – CD-10: I61, I69.1) where this was the first stroke event, including additional samples selected for prioritised DNA extraction and one case originally incorrectly recorded as an ischaemic stroke (IS); (b) all available cases of subarachnoid haemorrhage (SAH – ICD-10: I60, I69.0) where this was the first stroke event; (c) 5,662 cases of IS (ICD-10: I63, I69.3) occurring prior to 1 January 2014 at age $\leq$71 years where this was the first stroke event; (d) 1,008 incident cases of myocardial infarction (MI – ICD-10: I21-I23); and (e) all available cases of death with ischaemic heart disease as underlying cause (fatal IHD – ICD-10: I21-I25). Pairs of controls with no cardiovascular disease events or self-report were identified for each ICH case, matched to sex, recruitment region, and year of birth. For respiratory disease, 5,358 participants were selected with at least one event of hospitalisation with chronic obstructive pulmonary disease (COPD – ICD-10: J41-J44); as controls, 4,766 participants were randomly selected from amongst those who attended the second resurvey. For genotyping using the second version of the CKB array, selection was on the basis of complete boxes of DNA samples, prioritising those boxes that contained samples from participants originally recruited in clinics at which the second resurvey was conducted. These samples were supplemented with additional cases of ICH, SAH, MI, and fatal IHD that occurred subsequent to initial sample selection.

Genotyping was performed at BGI, Shenzhen, China. DNA samples selected for genome-wide genotyping were retrieved from storage at −70C, either as complete boxes of 96 samples or (for nested case-control samples) individually selected and transferred to new boxes, and were shipped on dry ice to BGI, Shenzhen. DNA concentration was checked using a NanoDrop Microvolume Spectrophotometer (Thermo Scientific), and a Microlab STAR liquid handling system (Hamilton) was used for transfer of sub-aliquots to new racks of 96 Fluidx cryovials and dilution with TE buffer to 80 ng/μL; the equivalent measured concentration of a subset of samples measured using Qubit DNA quantification (ThermoFisher) was 50 ng/μL. Diluted DNA was plated onto 96-well microtitre plates, with samples from a minimum of 3 boxes distributed across a single plate (a 1:1 mix of cases and controls for nested case-control samples). Samples with low DNA concentration were plated separately for genotyping with a modified first stage of the protocol, using a larger volume of DNA in place of TE buffer. Samples at position H12 were replaced with a duplicate sample from position D1 on the previous plate, thereby providing checks of genotyping quality and sample tracking. Genotyping was performed with manual target preparation according to Affymetrix protocols with automated plate processing and imaging using CKB_1 and CKB_2 Axiom® arrays and GeneTitan® Instruments.[89] Raw genotyping data were exported from China to the Oxford CKB International Coordinating Center under Data Export Approvals 2014-13 and 2015-39 from the Office of Chinese Human Genetic Resource Administration.

Genotyping quality control and calling (summarized in Table S4) was performed at CTSU, University of Oxford, UK, according to Affymetrix Best Practice workflow[18] using the Axiom Analysis Suite (Affymetrix) with default settings. Initial QC was performed on samples genotyped on batches of 50 plates. Genotyping was carried out for a preselected set of ~20K "high performance" SNPs, using the 'Sample QC' option, to give initial quality metrics. These were used to identify samples and plates to be excluded from subsequent steps, on the basis of sample DQC<0.82; sample QC call rate <97%; or plates with mean call rate for remaining samples <98.5%. Plates with sample pass rate <95% were flagged for inspection, and were excluded if there was evidence of a general failure of genotyping (e.g. large sections of the plate have failed), or if sample call rate was systematically low relative to sample DQC (rather than having a large number of failing samples due to e.g. a group of samples with poor-quality DNA). Some plate failures identified array manufacturing defects; genotyping of these plates was repeated using a new array.

Variant QC

Samples passing initial QC were processed and co-clustered, again in batches of 50 plates, to derive genotypes and further quality metrics. Within each batch, probesets were "failed" if they were classified as "OTV" (off target variation), "CallRateBelowThreshold" (using the default threshold 95%), or "Other", and genotypes for non-failed probesets; all further QC was performed using PLINK v1.9 and/or v2.0.[80] Within each batch, probesets were assessed for the presence of plate effects: logistic regressions were conducted to test each individual plate within a batch for significant deviations in genotype calling: each plate in turn was treated as "case" status with all other plates in the batch as controls, with recruitment center as covariate; probesets were failed according to criteria determined empirically through manual review of cluster plots to identify clustering failures – any plate effect with $p < 10^{-10}$, >3 instances of plate effect $p < 10^{-4}$, or any plate effect $p < 10^{-8}$ and clustering metrics FLD<8, HetSO<0.68, and HomRO<3.7; in addition, for probesets with any plate effect $P < 2 \times 10^{-5}$, cluster plots were manually reviewed, and appreciable clustering failures (e.g. poor cluster separation) were "failed".

Probesets passing this initial QC were combined into a single dataset, and a preliminary round of sample QC was performed (see below). A set of autosomal probesets pruned for linkage disequilibrium (LD; PLINK option `--indep-pairwise 50 5 0.1`) was then used to identify an unrelated subset of samples (PLINK `--rel-cutoff 0.025`). These were used to test for significant deviations in genotype calling between batches: logistic regressions were performed treating each batch in turn as "case" status with all other batches as controls, again with recruitment center as covariate; probesets were failed entirely, across *all* batches, again according

to criteria determined empirically through manual review of cluster plots to identify clustering failures: probesets with any batch effect with $p < 10^{-10}$, >2 or >7 with $p < 10^{-4}$, for array versions 1 and 2 respectively. Clustering was manually checked for remaining probesets with a batch effect with $p < 10^{-3}$ and were scored as either “Pass”, “Batch Fail” (fail in one batch only), or “Fail”. Probesets failing in >10% of batches (i.e. any batch for array version 1, >1 batch for version 2), or with call rates <98% (in passed batches) were excluded entirely from the dataset for that array version.

Probesets were then tested for deviation from Hardy-Weinberg equilibrium (HWE): tests were performed in each recruitment region separately (PLINK `--hardy midp`) using unrelated individuals (women only for chrX variants), and probesets with an HWE $p < 10^{-6}$ (10 degree of freedom sum-of-Chi-squared test) were excluded. In addition, variants with a minor allele frequency (MAF) > 0.2 different from that in the 3 Chinese populations from the 1000 Genome Project Phase 3 ref. [20] were excluded, and one pair of duplicate probesets assaying the same variant (that with the lower call rate) was removed.

Overall performance of the revised array was tested using 192 samples (152 Chinese, 40 European) from the European Vasculitis Genetics Consortium[90] genotyped using both CKB_2 and UK Biobank Axiom arrays. For 331,838 probesets passing QC on both arrays, concordance between the two arrays was assessed (PLINK `--merge-mode 7`): 99.5% of genotypes were non-missing for the data from both arrays, with a concordance of 99.80%. Combined Annotation Dependent Depletion (CADD v1.6)[21,22] was used to look up the predicted functional consequences of 721,308 variants passing QC on the CKB_2 array; the corresponding allele frequency in Europeans was according to the dbGAP Allele Frequency Aggregator (ALFA)[82] v2020-11-14, population SAMN10492695.

Sample QC

Primary sample QC was conducted for each array version separately, on the basis of criteria as summarized in Table 1. Based on genotyped variants passing QC as above, samples were excluded which had genotyping call rate <0.95, or high/low heterozygosity determined as follows: sample heterozygosity was assessed for autosomal variants with MAF>0.01 (PLINK `–het` followed by calculation of heterozygosity as 1–HOM/NMISS), mean and SD was determined for samples from each recruitment region (Note: there was a clear North-South gradient in heterozygosity, with a range of values > 1 SD), and samples with a region-specific *Z* score >+3 were excluded; total runs of homozygosity were determined for each sample (PLINK `--homozyg-kb 1000`), and 3 samples with a region-specific *Z* score <–3 *and* a *Z* score <2 for total runs of homozygosity were excluded (Figure S7).

Samples from individuals with appreciable non-Chinese ancestry were identified by projecting onto principal components derived from 2,504 individuals from 26 populations (5 ancestries) from 1000 Genomes Project Phase 3[20] using an LD-pruned set of 104,866 variants with MAF >0.01, passing QC for both CKB array versions, and excluding major regions of long-range LD[91] (PLINK `--pca --within --pca-clusters`) (Figure S14). A total of 4 individuals were excluded who had a value > 10 SDs from the CKB-wide mean for at least one of the first 10 PCs.

Initial checks of computed sex with that reported in participant data (PLINK `--check-sex`) identified multiple clusters of sex mismatches, indicating systematic linkage errors. All such clusters of mismatches were tracked back through all steps of sample handling, and the majority could be unambiguously traced to specific sample-handling errors (e.g. 180° rotation of boxes of DNA samples), such that correcting such sample-linkage errors removed all instances of sex mismatch in a cluster without leading to new ones. For clusters that remained uncorrected, all samples in the affected block of samples, irrespective of sex mismatch, were marked for exclusion from the dataset. Other individual sex-mismatched samples were also excluded.

For more detailed checks for sex mismatch, the chrY/chrX probe intensity ratio (parameter cn-probe-chrXY-ratio_gender_ratio output to file AxiomGT1.report.txt during genotyping) was plotted against the chromosome X heterozygosity F-statistics (from PLINK `--check-sex`), grouping genetically male and female samples into distinct clusters and clearly identifying sex mismatches (Figure S15A). In addition, groups of samples were observed representing potential chromosome XY aneuploidies, including Klinefelter Syndrome and non-Klinefelter XXY, and XO (Turner Syndrome) or XXX, and phenotypic males with appreciable chrX heterozygosity and lower than average chrY/chrX probe ratio. These latter individuals may include individuals with partial chrX translocations, but for most of them the heterozygous markers were distributed along the length of chrX. The samples corresponding to phenotypically male participants were clearly identifiable and were excluded without further investigation. To more robustly identify aneuploid female samples, probe intensity data was extracted using Affymetrix Axiom® CNV Tools software,[92] and 363 samples were identified whose mean probe intensity (LRR) on chrX was >3 SDs from the mean; for these, probe heterozygosity (BAF) was visualised across chromosome X, enabling identification of 6 Turner, 10 Turner mosaic, and 36 XXX individuals, either with no chrX heterozygosity (Turner) or with BAF values for heterozygous states consistently different from 0.5 (Figure S15B); other aneuploidies were also identified including a partial deletion of the p-arm of chrX and a complex rearrangement with both a partial q-deletion and partial p-duplication. All these individuals were marked for exclusion.

After merging the datasets for the two array versions into a single dataset, genetically identical samples with PI_HAT of ~1.0 were identified using an LD-pruned and thinned set of 10k autosomal SNPs with MAF>0.05 (PLINK `--thin-count 10000 –make-rel`). All expected duplicate pairs (including a small number of samples genotyped twice in error) were identified, confirming correct genotyping plate layout and order. All unexpected duplicate pairs were resolved as due either to repeat samples from the ~2,000 individuals known to have attended the baseline survey twice, or to pairs of individuals whose personal data at recruitment (e.g. recruitment location, date of birth) supported their assignment as putative monozygotic twins. For each pair of duplicate samples, the dataset with the lower call rate was excluded.

Imputation

Prior to imputation, additional QC excluded variants at multiallelic sites or with mismatched alleles compared with the 1000 Genomes Project Phase 3 refernece[20] (October 2014 release); where indicated, strand-flips were performed to match the reference. Imputation was conducted for each array version separately, in each case excluding variants that failed QC in any genotyping batch, and for a combined dataset limited to variants passing QC in all batches on both array versions. For imputation, samples were included which had been excluded from the main dataset on the basis of sex mismatch, linkage errors, or chromosome XY aneuploidy (autosomal imputation only). Phasing was performed for entire chromosomes using biobank-scale SHAPEIT3 r882[81] with default parameters, except for chromosome X (SHAPEIT2 v2.17[93] with the -X option, and with pseudoautosomal regions excluded). Imputation used the 1000 Genomes Project Phase 3 reference panel[20] filtered to exclude variants with MAF = 0 in the 5 East Asian populations, leaving 24,759,908 variants, and was conducted in 20 batches of samples split into 713 chunks (length ranges from 330Kbp to 5264Kbp, mean 3948Kbp) with buffer regions of 500Kbp, using IMPUTE4 v4.r265[16] for autosomes and IMPUTE2 v2.3.2[94] for chromosome X. Subsequent to imputation, checks for batch and array-version effects were conducted by testing for association using BOLT-LMM v2.3.1[29] with individual batches or array version as binary variables; 3867 variants displaying significant batch effects ($P < 5 \times 10^{-8}$) were excluded from the imputed dataset. After exclusion of variants with imputation info<0.3, imputed genotypes were available for 21,024,481 variants, of which 8,976,892 had MAF $\geq$ 0.01 (Tables S4 and S5; Figure S5).

Genetic analyses

Unless otherwise specified, genetic analyses were conducted using PLINK v1.9 and PLINK v2.0.[80] Sets of unrelated samples for variant QC and F_{st} analyses were derived using `--rel-cutoff`, but for PCA and exclusions for GWAS instead used `--king-cutoff 0.05`, in each case determined using LD-pruned sets of 122,675 autosomal variants with MAF>0.01 derived using `--indep-pairwise 50 5 0.1`. On the assumption that near relatives were not present in different recruitment regions, identity-by-descent was determined for all pairs of individuals within each region using `--genome gz`, from which first and second degree relatives were defined using PI-HAT thresholds of >0.375 and >0.1875, respectively (Tables S6 and S7; Figure S6) and, from the first-degree relatives, parent-child pairs were identified as those with Z0 <0.05 and Z1 >0.5, with the parent identified as the older of the pair. Each pair of siblings was checked for the number of recorded first-degree relatives in the dataset, and the family structures of mismatches were investigated, leading to the identification of one instance of 2 sets of putative three-quarter siblings.

PCA was conducted using FlashPCA v2.1[83] after LD pruning and exclusion of regions of long-range LD which, if not excluded or otherwise accounted for, can interfere with PCA potentially leading to erroneous conclusions about population structure, or to erroneous genetic association signals. Initial PCA used an LD-pruned set of SNPs excluding previously-identified regions of long-range LD,[91] but visualisation of variant weights revealed that multiple PCs were nevertheless affected by disproportionate contributions from particular regions of the genome, likely reflecting further regions of long-range LD present in the Chinese population (Figure S16). Therefore, following an approach similar to that previously used for UK Biobank,[95] a systematic iterative search was conducted to identify and remove regions of long range LD that influenced PCA in this way, using as a starting point an LD-pruned set of 180,570 autosomal variants with MAF>0.01, call rate>0.99, HWE $p > 10^{-4}$, derived using `--indep-pairwise 50 5 0.2`, in 76,719 unrelated CKB participants. Leading PCs from PCA were tested for the presence of long range LD regions, pairs of identified regions closer than 1Mbp were merged into single extended regions, variants within those regions were excluded, and the PCA was repeated. This process was continued until no long range LD regions were identified in any of the leading 11 PCs informative for CKB population structure, nor in the 12th (not informative) PC. Long range LD regions were identified using a hidden Markov model: presence within/outside a long range LD region was the hidden state; transition between states was in proportion to EAS recombination rates (downloaded from SNiPA[79]); and emission was the posterior probability of being in a long range LD region given the square of the Z score for the variant loadings for that PC. Variants were identified as within a long range LD region if they had a posterior marginal probability >0.5. A total of 223 regions were identified (Table S11) and variants within these regions were excluded so that 171,236 variants and 76,719 unrelated CKB participants were included in the final PCA; PCs for the remaining individuals were derived from the corresponding variant weights.

To identify PCs informative for population structure of the full CKB dataset, models were constructed predicting individuals' recruitment region in which the top PCs were progressively added to the model, using multinom() from R package 'nnet',[96] and Bayes Information Criterion (BIC) was derived using the R BIC() function, informative PCs being those that reduced BIC when added to the model (Figure S8). These were confirmed by ANOVA tests for non-random association of PCs with region of recruitment; above-trend eigenvalues on a scree plot; and visual examination of plots of the top PCs with colour-coding of region of recruitment (Figure S9). Similarly, PCs informative for local population structure were identified on the basis of BIC for linear models predicting latitude and longitude for the assessment center at which individuals were recruited (Figure S11) or Han status (Figure S12). Maps used in PCA plots were drawn with R package 'ggmap'[84] using map tiles by Stamen Design (maps.stamen.com) under CC BY 3.0, using data by OpenStreetMap under Open Data Commons Open Database License.

Analysis subsets

Subsets of the full genotyped dataset were derived for different analysis approaches (Table S9). For region-stratified analyses, samples with non-local ancestry were excluded; these were identified as outliers for one or more of the informative PCs for that region, on the basis of a robust Mahalanobis distance (from the R mahalanobis() function) of >3 SDs. For analyses requiring unrelated

individuals, these were defined using 122,675 LD-pruned variants with MAF>0.01, as above, but applying PLINK `--king-cutoff 0.05` which generally gives a larger set of unrelated individuals than `--rel-cutoff`.

Construction of a subset of genotyped individuals that was largely representative of the overall CKB cohort was based on the fact that the majority of genotyped samples were not selected individually but as complete boxes of DNA samples. These boxes of DNA were prioritised for genotyping solely according to the number of samples they contained that were from participants recruited at study clinics subsequently used for the second resurvey; these clinics had themselves been selected to be population-representative. The procedures for sample collection and DNA extraction meant that each box of DNA included a mixture of samples from at least two randomly-selected boxes of buffy coat samples. Therefore, samples in boxes of DNA were either from individuals invited to the second resurvey and therefore largely representative of the overall CKB cohort, or were random collections of samples from other recruitment locations.

An initial attempt to construct a cohort-representative subset used samples from those boxes with at least 70% of samples genotyped (irrespective of QC), but this was found to be *depleted* for certain ascertained disease cases; this was due to the early prioritisation of a proportion of ICH, SAH, and fatal IHD cases, which led to the transfer of these samples to different storage locations prior to DNA extraction. Therefore, the CKB-representative subset was instead based on the boxes in which blood samples were originally stored immediately after collection and processing at time of recruitment, and used samples originating from boxes of buffy coat with $\geq$40% of samples selected for genotyping. This gave a set of 77,176 participants which were representative of the overall CKB cohort, in which over-representation of the ascertained diseases was eliminated.

Genome-wide association

GWASs were performed for each 3-character ICD-10 chapter with at least 100 genotyped cases, with non-case members of the population representative set of 77,176 individuals as controls (Tables S1 and S9); for diseases expected to be specific to males or females, analyses were restricted to the corresponding sex. Analyses used SAIGE[25] version 0.42.1 with array version, sex, age, age,[2] recruitment region and the first 11 national PCs as covariates, and relatedness defined by the LD-pruned set of 122,675 autosomal variants, and were restricted to variants with MAF>0.01, with additional filtering of variants with an effective MAC<20, according to the formula MAC_{eff} = 2*MAF*(imputation info)*N_{eff}, where $N_{eff} = 4/(1/N_{cases} + 1/N_{controls})$. Loci at genome-wide significant variants ($P < 5 \times 10^{-8}$) were defined by LD-clumping with `--clump-p1 5e-8 --clump-kb 5000 --clump-r2 0.05 --clump-p2 0.05` options. Locus novelty was assessed by checking for previously-reported genome-wide significant associations or pathogenic mutations within locus boundaries, according to GWAS catalog,[76] OpenGWAS,[77] and ClinVar.[78] Regional association plots were generated using LocusZoom v1.4[85] using 10,000 randomly-selected unrelated CKB participants for the LD reference and recombination rates derived from the 1000 Genomes Project Phase 3 EAS populations[20] using SniPA.[79]

Cell Genomics, Volume *3*

Supplemental information

Genotyping and population characteristics

# of the China Kadoorie Biobank

Robin G. Walters, Iona Y. Millwood, Kuang Lin, Dan Schmidt Valle, Pandora McDonnell, Alex Hacker, Daniel Avery, Ahmed Edris, Hannah Fry, Na Cai, Warren W. Kretzschmar, M. Azim Ansari, Paul A. Lyons, Rory Collins, Peter Donnelly, Michael Hill, Richard Peto, Hongbing Shen, Xin Jin, Chao Nie, Xun Xu, Yu Guo, Canqing Yu, Jun Lv, Robert J. Clarke, Liming Li, Zhengming Chen, and China Kadoorie Biobank Collaborative Group

Genotyping and population characteristics of the China Kadoorie Biobank

Robin G Walters, Iona Y Millwood, Kuang Lin, Dan Schmidt Valle, Pandora McDonnell, Alex Hacker, Daniel Avery, Ahmed Edris, Hannah Fry, Na Cai, Warren W Kretzschmar, M Azim Ansari, Paul A Lyons, Rory Collins, Peter Donnelly, Michael R Hill, Richard Peto, Hongbing Shen, Xun Xu, Yu Guo, Canqing Yu, Jun Lv, Robert J Clarke, Liming Li, Zhengming Chen, for the China Kadoorie Biobank Collaborative Group

Supplementary Information

Members of the China Kadoorie Biobank Collaborative Group 2

Supplementary Figures 3

Supplementary Data S1: China Kadoorie Biobank Array Design 21

Supplementary Data S2: China Kadoorie Biobank Array Revision 26

Members of the China Kadoorie Biobank Collaborative Group

International Steering Committee: Junshi Chen, Zhengming Chen (PI), Robert Clarke, Rory Collins, Yu Guo, Liming Li (PI), Chen Wang, Jun Lv, Richard Peto, Robin Walters.

International Co-ordinating Centre, Oxford: Daniel Avery, Derrick Bennett, Ruth Boxall, Sushila Burgess, Ka Hung Chan, Yiping Chen, Zhengming Chen, Johnathan Clarke; Robert Clarke, Huaidong Du, Ahmed Edris, Hannah Fry, Simon Gilbert, Mike Hill, Pek Kei Im, Andri Iona, Maria Kakkoura, Christiana Kartsonaki, Hubert Lam, Kuang Lin, Mohsen Mazidi, Iona Millwood, Sam Morris, Qunhua Nie, Alfred Pozarickij, Paul Ryder, Saredo Said, Dan Schmidt, Paul Sherliker, Becky Stevens, Iain Turnbull, Robin Walters, Baihan Wang, Lin Wang, Neil Wright, Ling Yang, Xiaoming Yang, Pang Yao.

National Co-ordinating Centre, Beijing: Xiao Han, Can Hou, Qingmei Xia, Chao Liu, Jun Lv, Pei Pei, Canqing Yu.

Regional Co-ordinating Centres:

Gansu: Gansu Provincial CDC – Caixia Dong, Pengfei Ge, Xiaolan Ren. Maiji CDC – Zhongxiao Li, Enke Mao, Tao Wang, Hui Zhang, Xi Zhang. **Haikou:** Hainan Provincial CDC – Jinyan Chen, Ximin Hu, Xiaohuan Wang. Meilan CDC – Zhendong Guo, Huimei Li, Yilei Li, Min Weng, Shukuan Wu. **Harbin:** Heilongjiang Provincial CDC – Shichun Yan, Mingyuan Zou, Xue Zhou. Nangang CDC – Ziyan Guo, Quan Kang, Yanjie Li, Bo Yu, Qinai Xu. **Henan:** Henan Provincial CDC – Liang Chang, Lei Fan, Shixian Feng, Ding Zhang, Gang Zhou. Huixian CDC – Yulian Gao, Tianyou He, Pan He, Chen Hu, Huarong Sun, Xukui Zhang. **Hunan:** Hunan Provincial CDC – Biyun Chen, Zhongxi Fu, Yuelong Huang, Huilin Liu, Qiaohua Xu, Li Yin. Liuyang CDC – Huajun Long, Xin Xu, Hao Zhang, Libo Zhang. **Liuzhou:** Guangxi Provincial CDC – Naying Chen, Duo Liu, Zhenzhu Tang. Liuzhou CDC – Ningyu Chen, Qilian Jiang, Jian Lan, Mingqiang Li, Yun Liu, Fanwen Meng, Jinhuai Meng, Rong Pan, Yulu Qin, Ping Wang, Sisi Wang, Liuping Wei, Liyuan Zhou. **Qingdao:** Qingdao CDC – Liang Cheng, Ranran Du, Ruqin Gao, Feifei Li, Shanpeng Li, Yongmei Liu, Feng Ning, Zengchang Pang, Xiaohui Sun, Xiaocao Tian, Shaojie Wang, Yaoming Zhai, Hua Zhang, Licang CDC – Wei Hou, Silu Lv, Junzheng Wang. **Sichuan:** Sichuan Provincial CDC – Xiaofang Chen, Xianping Wu, Ningmei Zhang, Weiwei Zhou. Pengzhou CDC – Xiaofang Chen, Jianguo Li, Jiaqiu Liu, Guojin Luo, Qiang Sun, Xunfu Zhong. **Suzhou:** Jiangsu Provincial CDC – Jian Su, Ran Tao, Ming Wu, Jie Yang, Jinyi Zhou, Yonglin Zhou. Suzhou CDC – Yihe Hu, Yujie Hua, Jianrong Jin Fang Liu, Jingchao Liu, Yan Lu, Liangcai Ma, Aiyu Tang, Jun Zhang. **Zhejiang**: Zhejiang Provincial CDC – Weiwei Gong, Ruying Hu, Hao Wang, Meng Wang, Min Yu. Tongxiang CDC – Lingli Chen, Qijun Gu, Dongxia Pan, Chunmei Wang, Kaixu Xie, Xiaoyi Zhang.

Supplementary Figures

Figure S1. CKB Axiom® array design overview. The figure summarises the data sources used for array design and the filtering, QC, and variants selection procedures applied. Related to **Figure 2**.

Figure S2. Design of the CKB Axiom® genotyping array. The figure illustrates the different categories of content on the initial CKB array. Numbers indicate the approximate counts of variants in each category. Some variants fall into more than one category. Related to **Figure 2**.

Figure S3. CKB Axiom® array design revision overview. The figure summarises the procedures used to update the array design. Related to **Figure 2**.

Figure S4. Allele frequency of genotyped variants in CKB regions. Allele frequency of QCed variants on array v2 in each CKB region, compared with the corresponding allele in the East Asian subset of the 1000 genomes Phase 3 reference. Related to **Figure 3**.

Figure S5. Imputation quality for each CKB array version. The distribution is shown for imputation INFO score for variants in 4 MAF ranges, for the results of imputation using genotyping data from each CKB array version separately. Related to **STAR Methods**.

Figure S6. Patterns of relatedness in CKB regions. The histograms show for each CKB region the distributions of the relatedness between all possible pairs of genotyped individuals. Related to **STAR Methods**.

Figure S7. Quality control for heterozygosity and homozygosity. Overall heterozygosity and total runs of homozygosity were determined for each genotyping dataset. Blue symbols denote samples with low heterozygosity that is accounted for by extended runs of homozygosity. Red symbols, indicated by an arrow, denote samples whose heterozygosity is not accounted for by runs of homozygosity and which were excluded from the analysis dataset. Related to **STAR Methods**.

Figure S8. Identification of informative principal components. Models predicting participant recruitment region were constructed by progressively adding PCs from PCA of the full CKB cohort, and Bayes Information Criterion was determined. Related to **STAR Methods**.

Figure S9. Principal component analysis of CKB. The results of PCA of the full CKB genotyped dataset are shown for pairwise plots of all PCs that were informative for CKB recruitment region. Data points are colour coded according to the region from which that participant was recruited. Related to **Figure 4**.

Figure S10. Population structure in CKB regions as informed by whole cohort PCA. Local maps are shown for each recruitment region, showing the geolocation of the individual recruitment clinics, colour coded according to latitude and longitude; the size of the symbol is proportional to the number of genotyped individuals from that clinic. Corresponding PCA plots show the first two principal components from PCA of the full CKB cohort, colour coded according to their recruitment clinic. Top 2 rows ¬– urban regions; bottom 2 rows – rural regions. Related to **Figure 4**.

Figure S11. Identification of informative principal components for CKB regions. Models predicting the latitude (blue) and longitude (red) of participants' recruitment clinic were constructed by progressively adding PCs, and Bayes Information Criterion was determined. Broken lines – PCA of the entire CKB cohort; solid lines – PCA of each region separately. Related to **STAR Methods**.

Figure S12. Population structure in Liuzhou region. Participants recruited in Liuzhou who attended the second resurvey are plotted according to PCA from the entire CKB cohort (top) or Liuzhou only (bottom). Blue – self-reported Han ancestry; orange – mixed ancestry; red – non-Han ancestry. Plots (right) show the Bayes Information Criterion for models predicting Han status using increasing numbers of PCs. Related to **Figure 4**.

Figure S13. Population diversity in CKB. Population differences as measured by Fst were derived, and trees were constructed to illustrate the relationships between them the populations shown. (A) Phylogenetic tree derived using the full unrelated CKB dataset, except for Liuzhou (RC46) for which only second resurvey participants were included. (b) Neighbour-joining tree constructed using 100 unrelated individuals from each population. RC12 – Qingdao; RC16 – Harbin; RC26 – Haikou; RC36 – Suzhou; RC46 – Liuzhou; RC52 – Sichuan; RC58 – Gansu; RC68 – Henan; RC78 – Zhejiang; RC88 – Hunan; CHB, CHS, JPT, CDX, KHV – East Asian 1000 Genomes populations. Related to **Figure 4**.

Figure S14. PCA projection onto 1000 Genomes. PCA was conducted for the 1000 Genomes Phase 3 populations, and CKB participants were projected onto the resulting PCs. Top – 1000 Genomes populations; bottom – with CKB participants (black) included. Related to **STAR Methods**.

Figure S15. Identification of sex mismatches and chromosome XY aneuploidies. (A) plot showing relationship between chromosome X homozygosity and chromosome XY probe ratio. Sex-mismatched samples are visible within the main clusters of females (red) and males (blue). Open symbols denote samples identified as potential aneuploidies. (B) Plots across chromosome X of the BAF parameter which reflects the proportion of signal on the genotyping array coming from the two possible alleles at each site. 3 classes of aneuploidy are illustrated, the red marks highlighting systematic deviations from the expected 3 possible genotypes. Related to **STAR Methods**.

Figure S16. Distortion of PCA by regions of long range LD. Plots show individual variant loadings (Z-squared), for each of the first 12 PCs from PCA of the full CKB cohort, that result if regions of long range LD are not fully excluded. Related to **STAR Methods**.

Figure S1

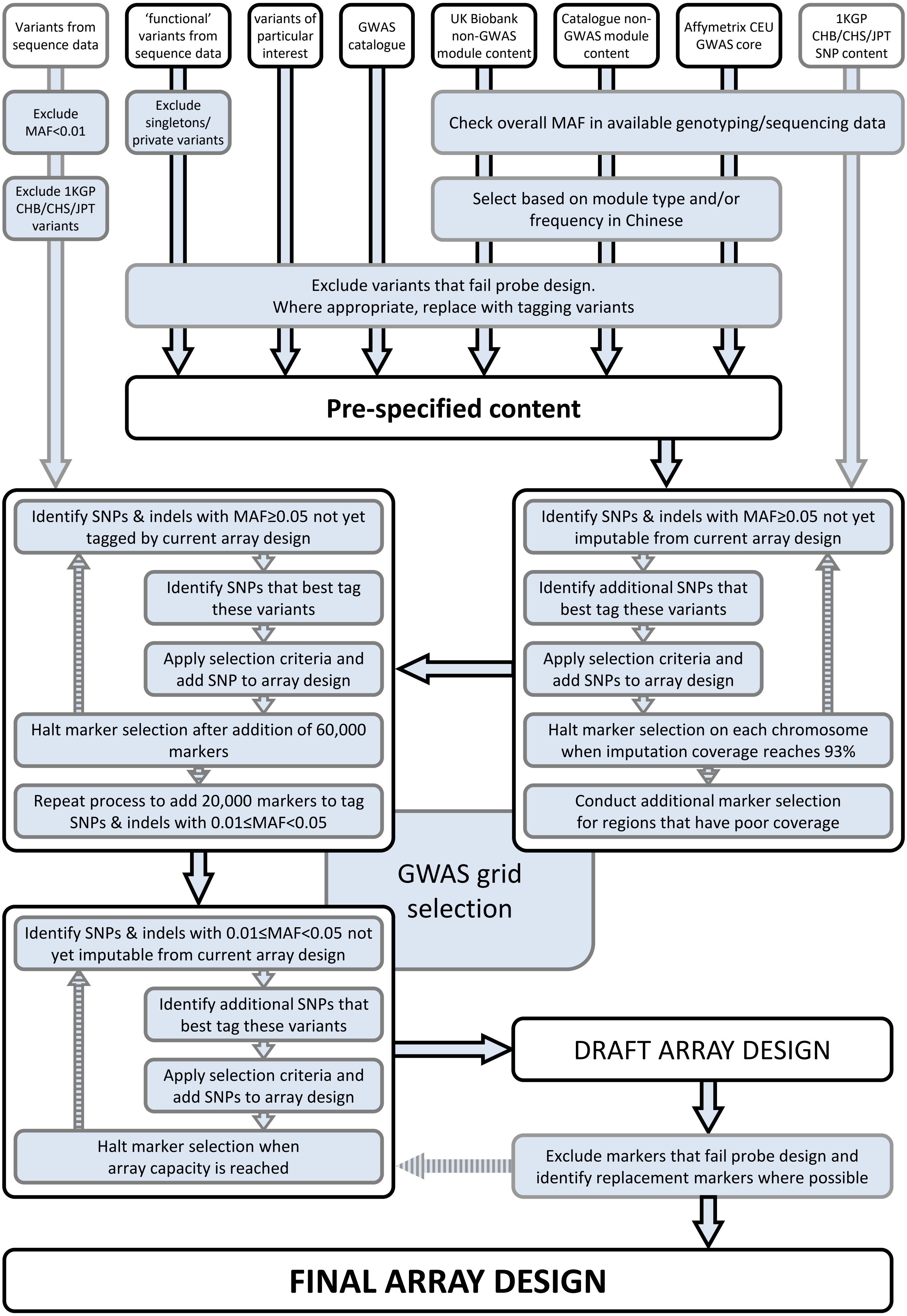
Variants from sequence data
'functional' variants from sequence data
variants of particular interest
GWAS catalogue
UK Biobank non-GWAS module content
Catalogue non-GWAS module content
Affymetrix CEU GWAS core
1KGP CHB/CHS/JPT SNP content
Exclude MAF<0.01
Exclude 1KGP CHB/CHS/JPT variants
Exclude singletons/ private variants
Check overall MAF in available genotyping/sequencing data
Select based on module type and/or frequency in Chinese
Exclude variants that fail probe design. Where appropriate, replace with tagging variants
Pre-specified content
Identify SNPs & indels with MAF≥0.05 not yet tagged by current array design
Identify SNPs that best tag these variants
Apply selection criteria and add SNP to array design
Halt marker selection after addition of 60,000 markers
Repeat process to add 20,000 markers to tag SNPs & indels with 0.01≤MAF<0.05
Identify SNPs & indels with MAF≥0.05 not yet imputable from current array design
Identify additional SNPs that best tag these variants
Apply selection criteria and add SNPs to array design
Halt marker selection on each chromosome when imputation coverage reaches 93%
Conduct additional marker selection for regions that have poor coverage
GWAS grid selection
Identify SNPs & indels with 0.01≤MAF<0.05 not yet imputable from current array design
Identify additional SNPs that best tag these variants
Apply selection criteria and add SNPs to array design
Halt marker selection when array capacity is reached
DRAFT ARRAY DESIGN
Exclude markers that fail probe design and identify replacement markers where possible
FINAL ARRAY DESIGN

Figure S2

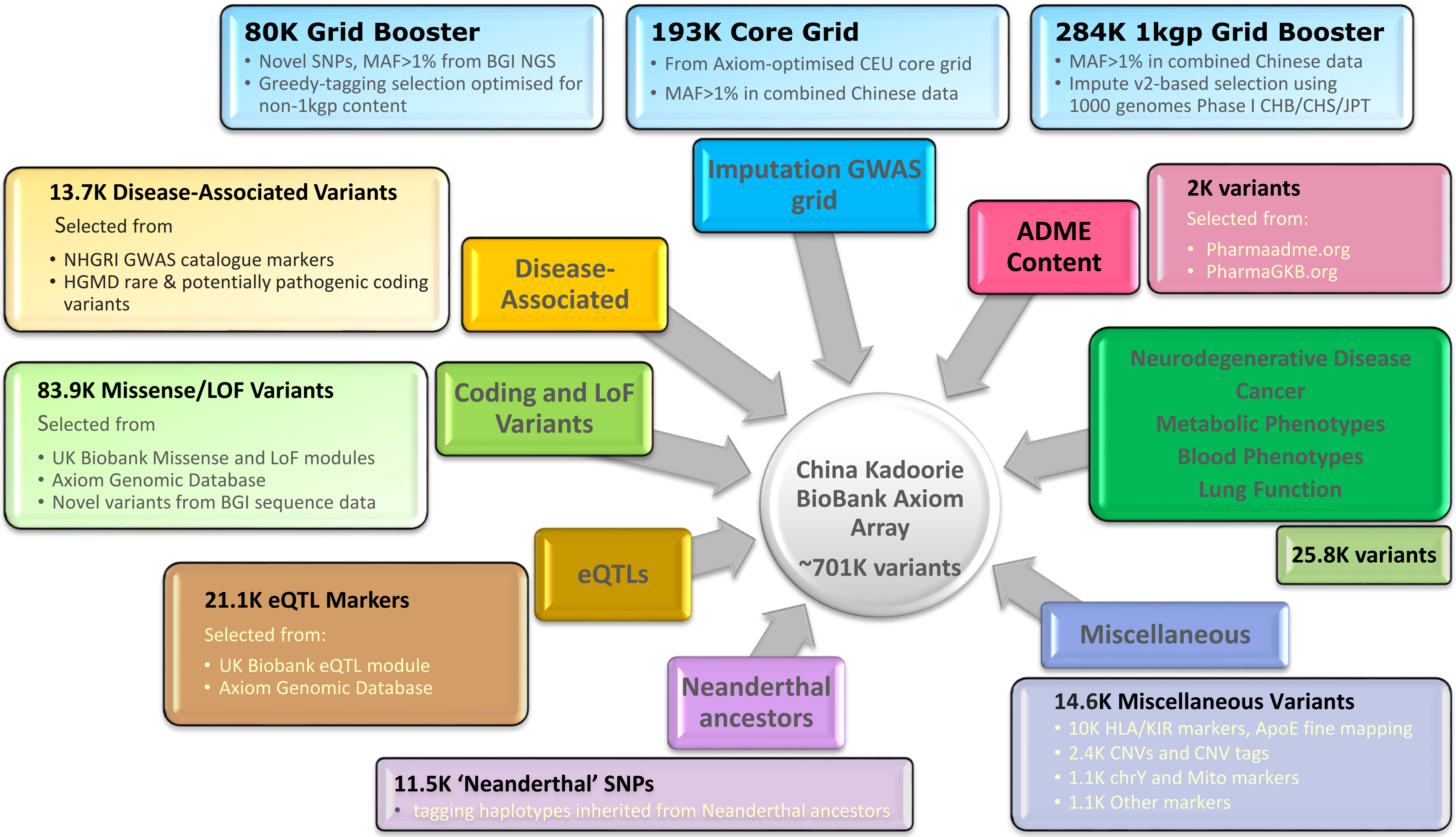
80K Grid Booster
• Novel SNPs, MAF>1% from BGI NGS
• Greedy-tagging selection optimised for non-1kgp content
193K Core Grid
• From Axiom-optimised CEU core grid
• MAF>1% in combined Chinese data
284K 1kgp Grid Booster
• MAF>1% in combined Chinese data
• Impute v2-based selection using 1000 genomes Phase I CHB/CHS/JPT
Imputation GWAS grid
13.7K Disease-Associated Variants
Selected from
• NHGRI GWAS catalogue markers
• HGMD rare & potentially pathogenic coding variants
Disease-Associated
ADME Content
2K variants
Selected from:
• Pharmaadme.org
• PharmaGKB.org
83.9K Missense/LOF Variants
Selected from
• UK Biobank Missense and LoF modules
• Axiom Genomic Database
• Novel variants from BGI sequence data
Coding and LoF Variants
Neurodegenerative Disease
Cancer
Metabolic Phenotypes
Blood Phenotypes
Lung Function
25.8K variants
China Kadoorie BioBank Axiom Array
~701K variants
eQTLs
21.1K eQTL Markers
Selected from:
• UK Biobank eQTL module
• Axiom Genomic Database
Neanderthal ancestors
Miscellaneous
14.6K Miscellaneous Variants
• 10K HLA/KIR markers, ApoE fine mapping
• 2.4K CNVs and CNV tags
• 1.1K chrY and Mito markers
• 1.1K Other markers
11.5K 'Neanderthal' SNPs
• tagging haplotypes inherited from Neanderthal ancestors

Figure S3

Review results from first 100 plates

Poly High Resolution	Review, default=retain
No Minor Homozygote	Review, default=retain
Mono High Resolution	Review and check frequency in CONVERGE
Hemizygous	Review
Off Target Variant	Review, default=exclude
Call Rate Below Threshold	Review, default=exclude
Other	Review, default=exclude
Viral probes	Review, default=retain

Identify unneeded 2nd probesets
Check call rate for all retained SNPs

Remove from design

- Failed and low-quality assays
- Non-preferred probesets
- Monomorphic variants (except for selected variants e.g. those confirmed at acceptable frequencies in CONVERGE)
- Viral probes?

Add to design

- Modified assays for retained SNPs
- Tag SNPs for important failed assays
- Novel content from CONVERGE
- New GWAS hits
- Additional content from collaborators

Augment GWAS grid

- Restore gaps caused by SNP removal
- Supplement low freq. grid

Figure S4

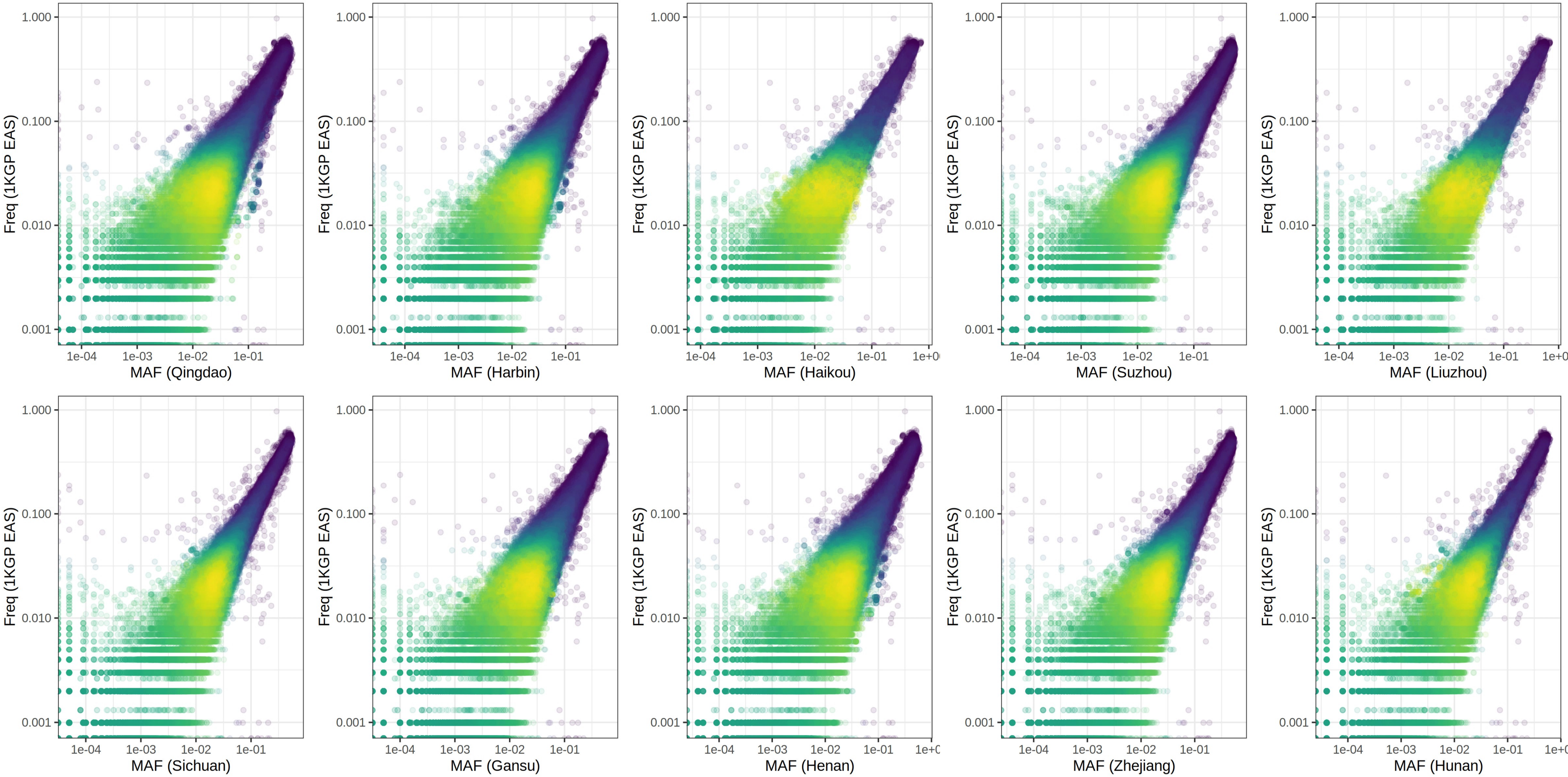

Freq (1KGP EAS)
1.000
0.100
0.010
0.001
1e-04
1e-03
1e-02
1e-01
1e+0
MAF (Qingdao)
MAF (Harbin)
MAF (Haikou)
MAF (Suzhou)
MAF (Liuzhou)
MAF (Sichuan)
MAF (Gansu)
MAF (Henan)
MAF (Zhejiang)
MAF (Hunan)

Figure S5

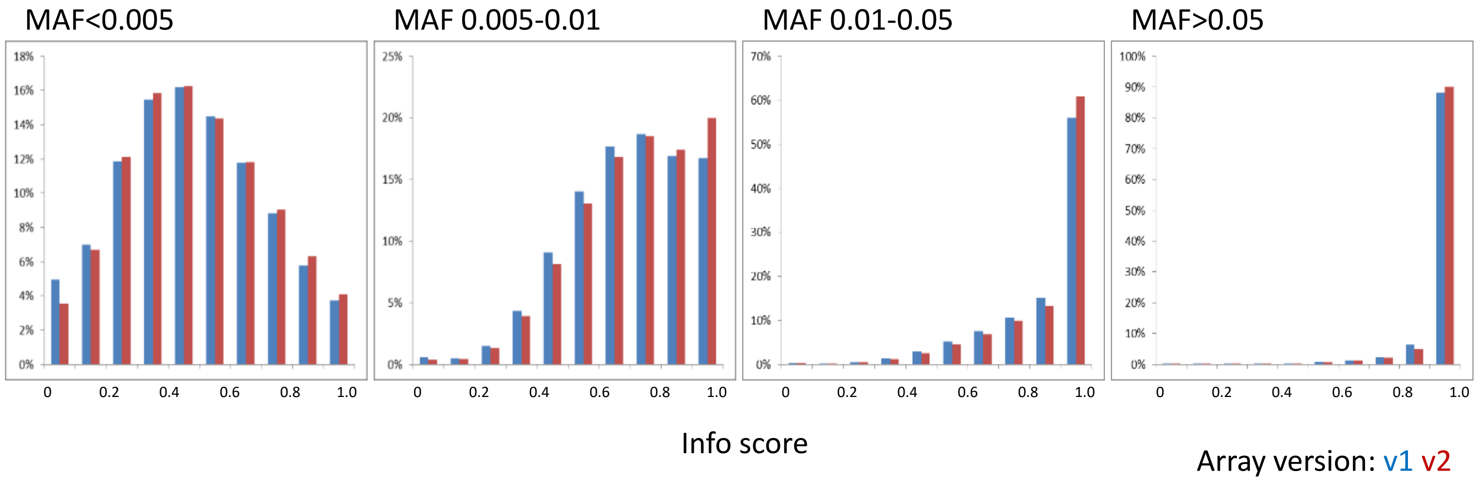
MAF<0.005
MAF 0.005-0.01
MAF 0.01-0.05
MAF>0.05
Info score
Array version: v1 v2

Figure S6

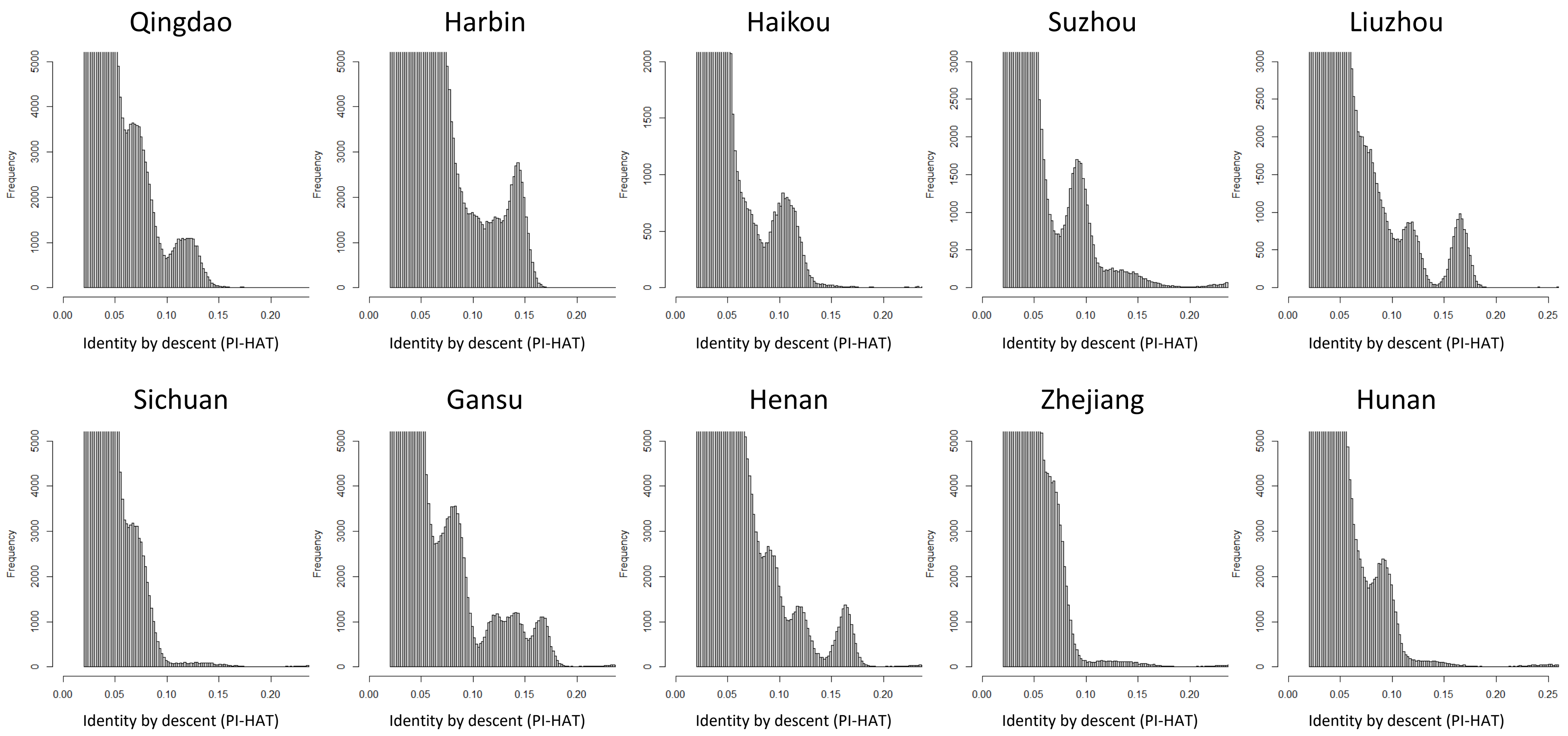
Qingdao
Harbin
Haikou
Suzhou
Liuzhou
Sichuan
Gansu
Henan
Zhejiang
Hunan
Frequency
Identity by descent (PI-HAT)
0.00
0.05
0.10
0.15
0.20
0.25
0
500
1000
1500
2000
2500
3000
4000
5000

Figure S7

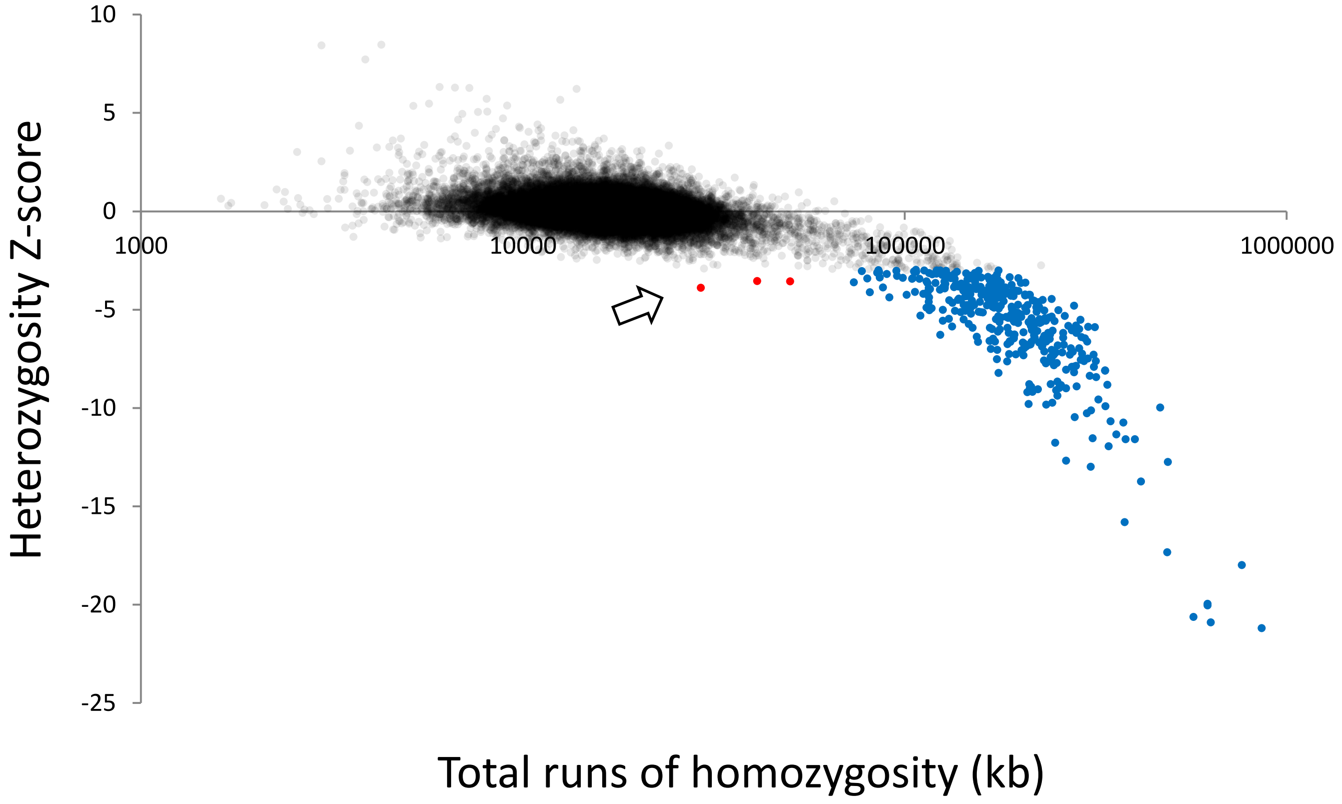

10
5
0
-5
-10
-15
-20
-25
1000
10000
100000
1000000
Heterozygosity Z-score
Total runs of homozygosity (kb)

Figure S8

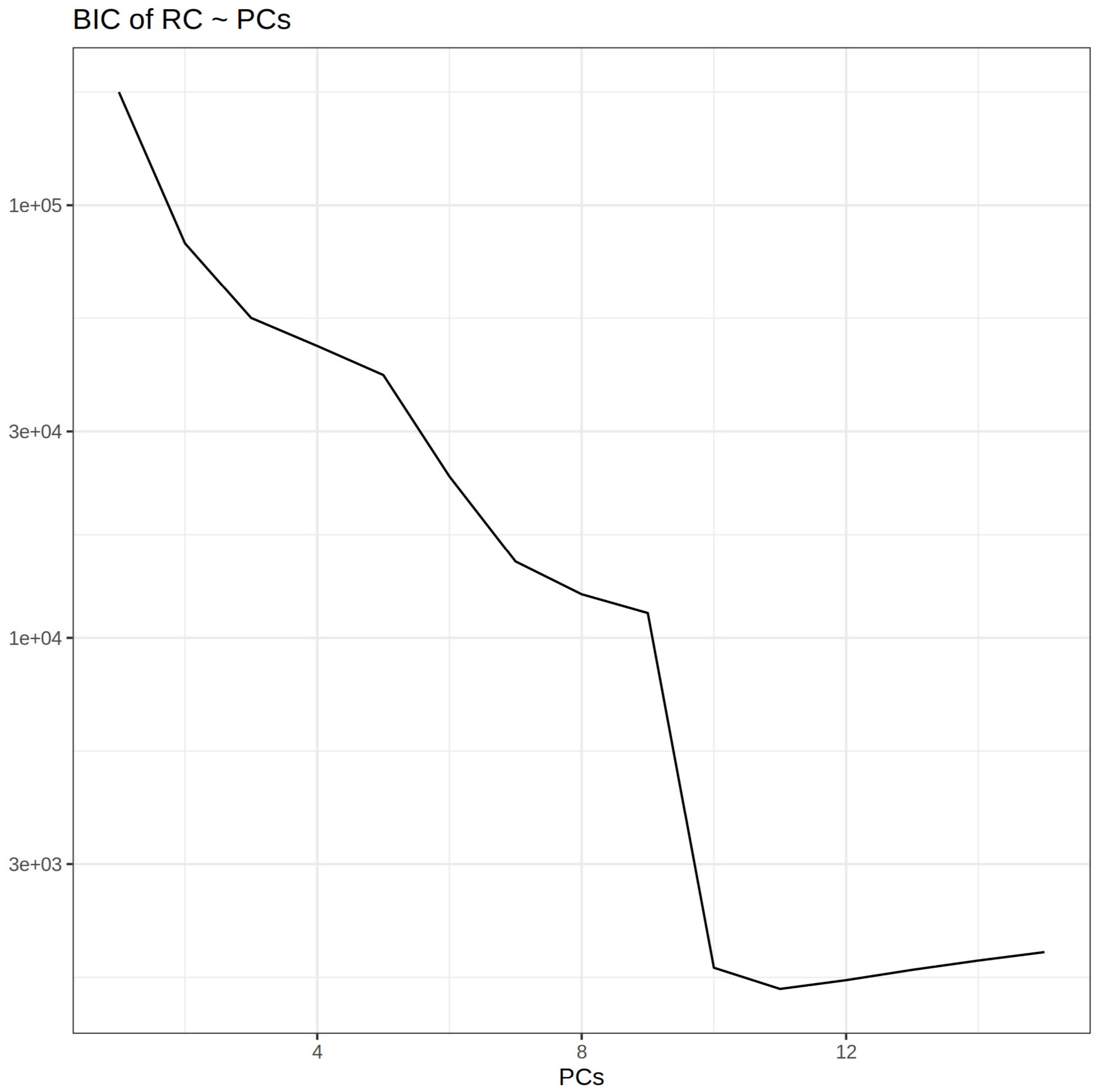

Figure S9

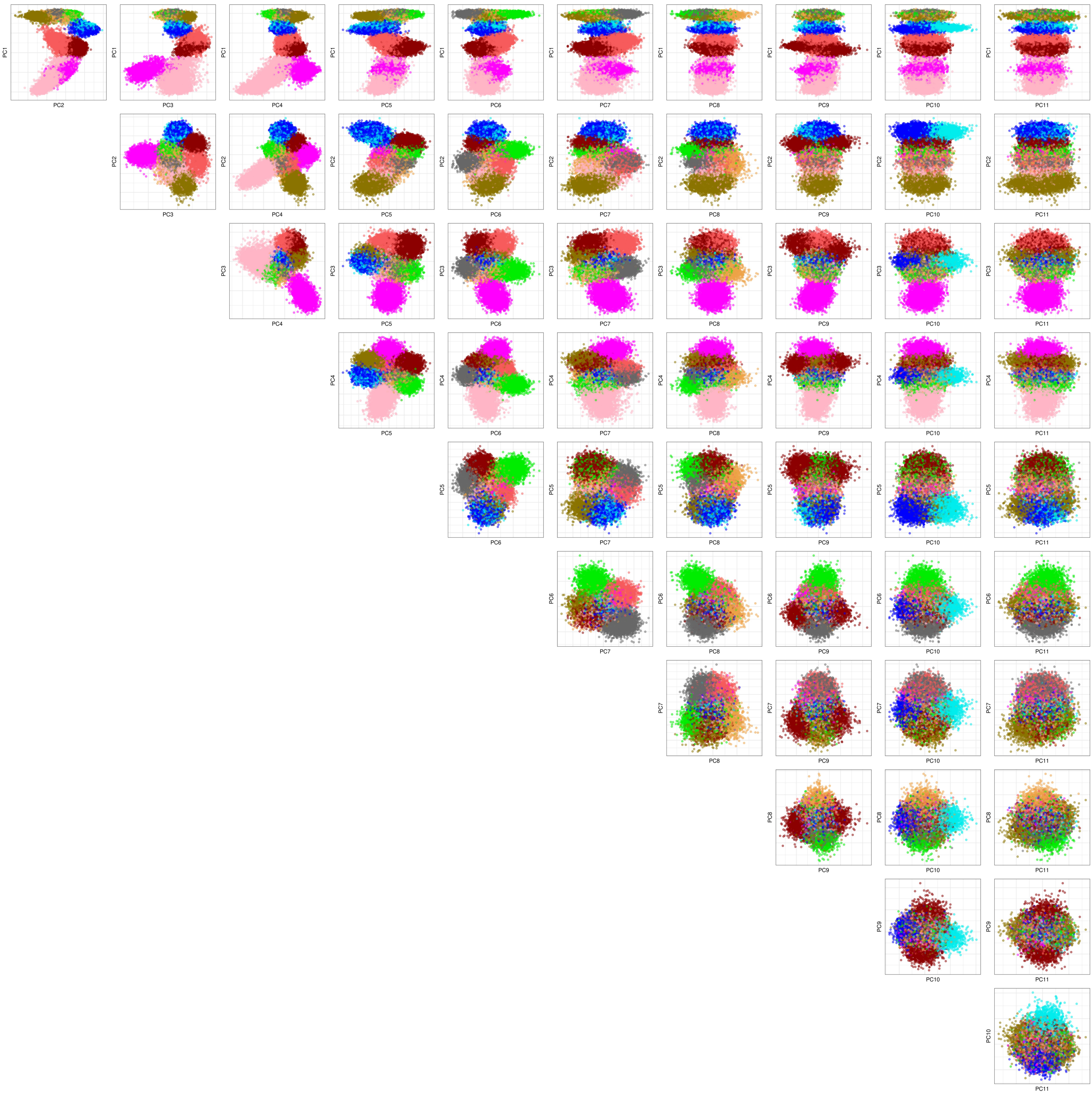

Figure S10

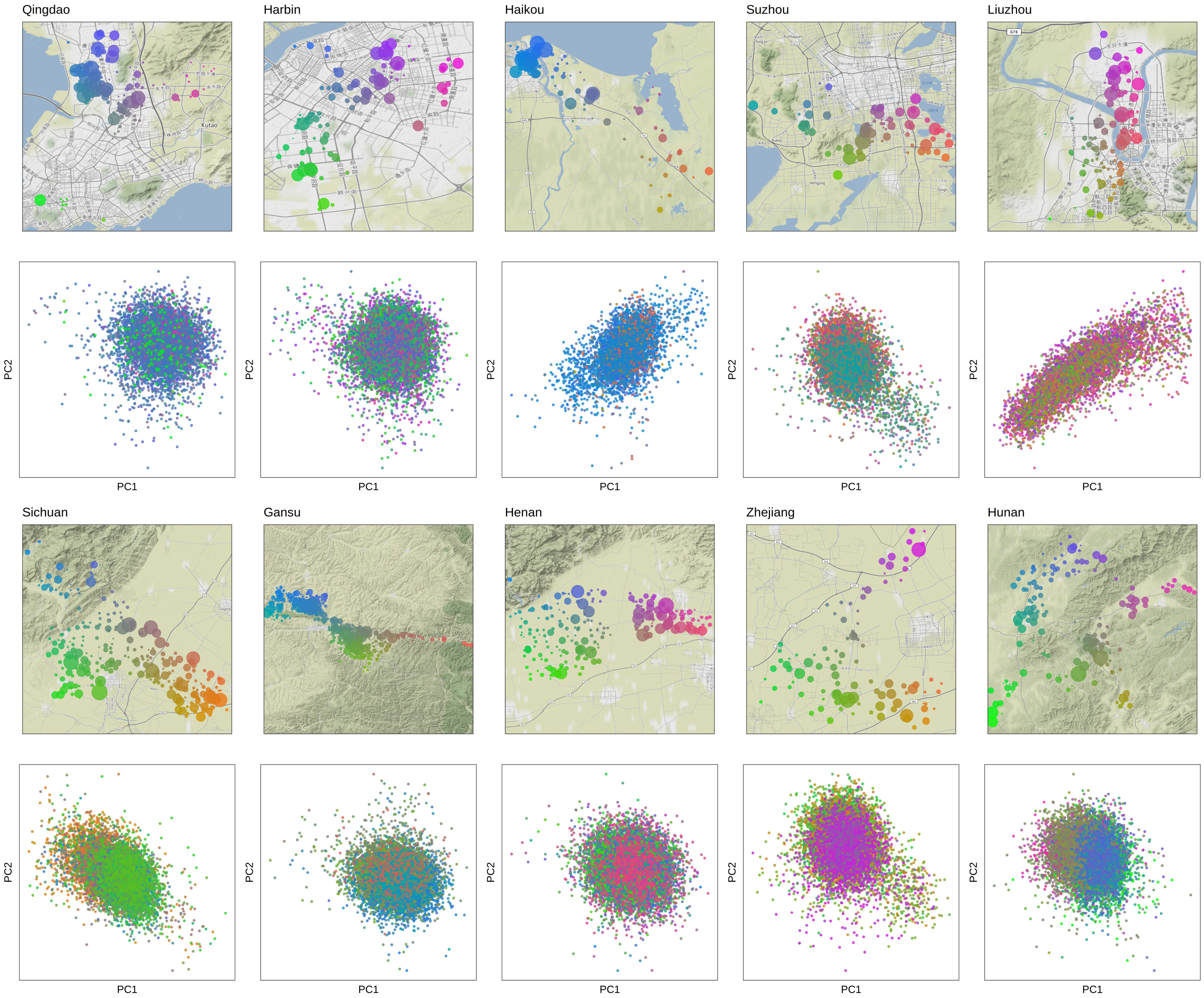
Qingdao
Harbin
Haikou
Suzhou
Liuzhou
Kutao
PC1
PC2
Sichuan
Gansu
Henan
Zhejiang
Hunan

Figure S11

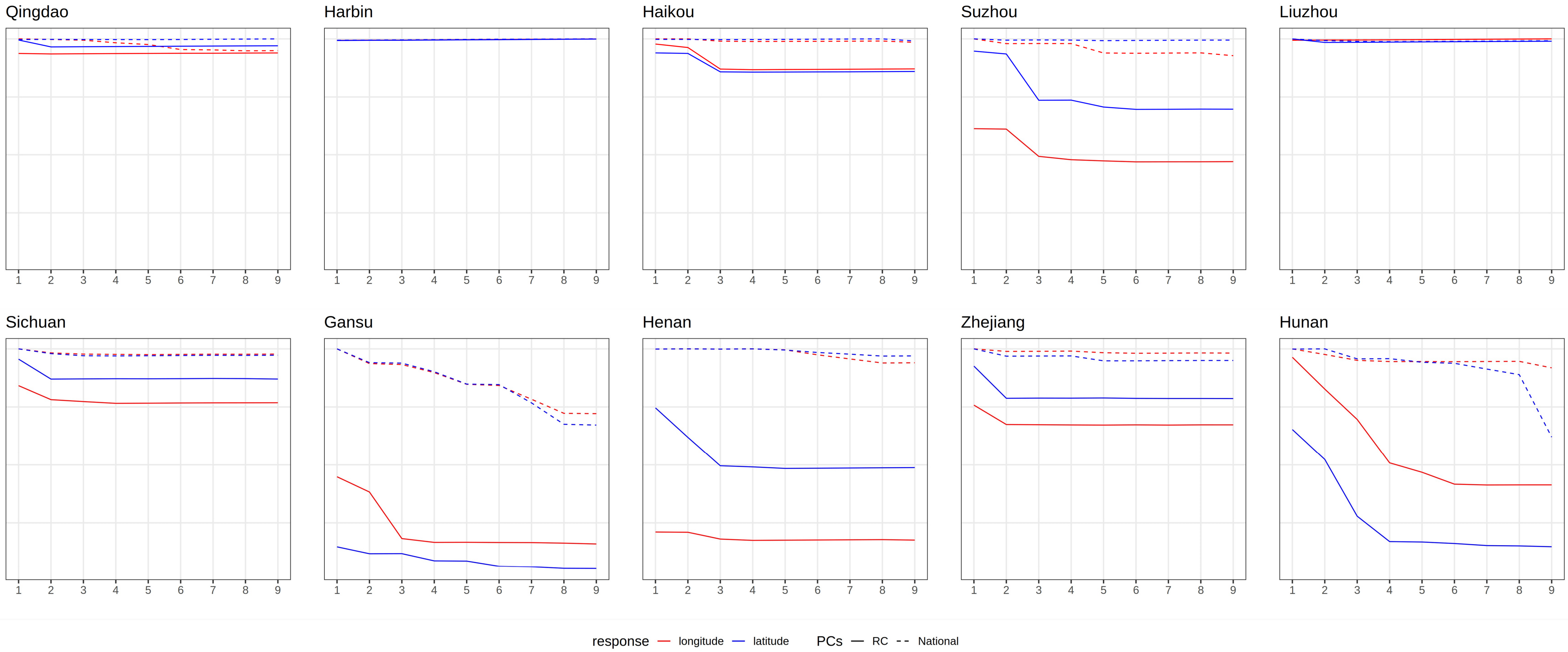

Qingdao
Harbin
Haikou
Suzhou
Liuzhou
Sichuan
Gansu
Henan
Zhejiang
Hunan
1 2 3 4 5 6 7 8 9
response longitude latitude
PCs RC National

Figure S12

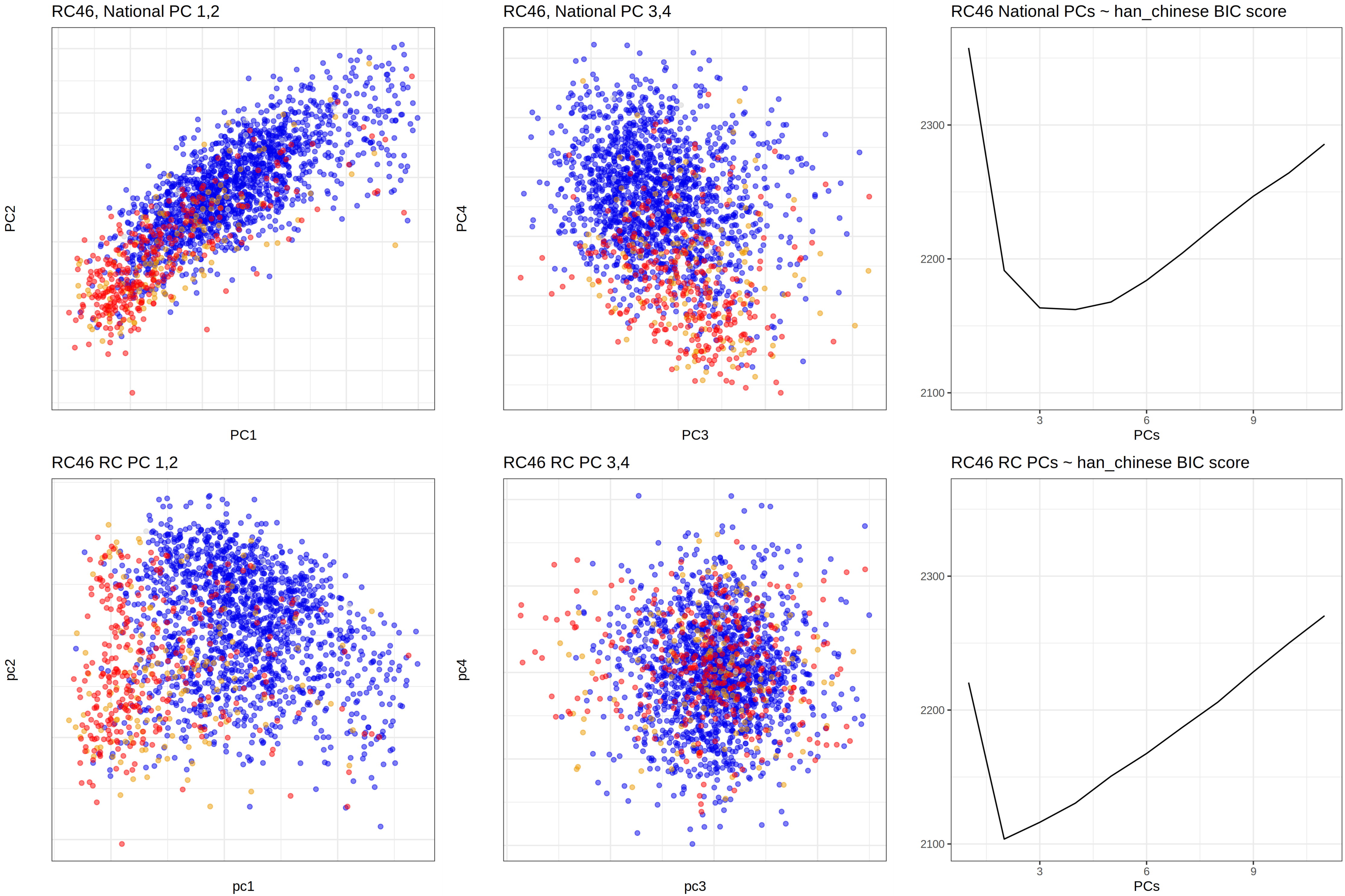
RC46, National PC 1,2
PC2
PC1
RC46, National PC 3,4
PC4
PC3
RC46 National PCs ~ han_chinese BIC score
2300
2200
2100
3
6
9
PCs
RC46 RC PC 1,2
pc2
pc1
RC46 RC PC 3,4
pc4
pc3
RC46 RC PCs ~ han_chinese BIC score
2300
2200
2100
3
6
9
PCs

Figure S13

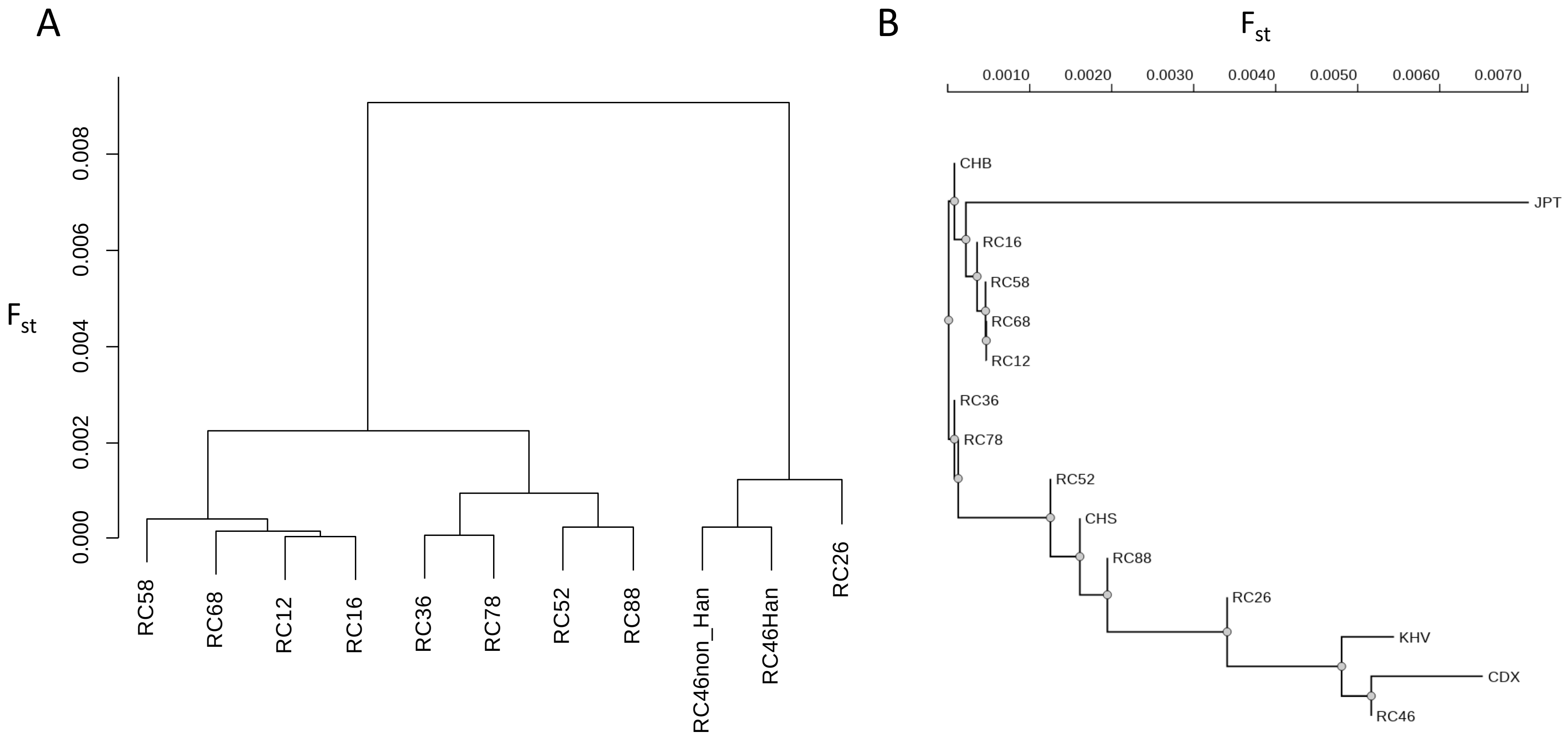
A
Fst
0.000
0.002
0.004
0.006
0.008
RC58
RC68
RC12
RC16
RC36
RC78
RC52
RC88
RC46non_Han
RC46Han
RC26
B
Fst
0.0010
0.0020
0.0030
0.0040
0.0050
0.0060
0.0070
CHB
JPT
RC16
RC58
RC68
RC12
RC36
RC78
RC52
CHS
RC88
RC26
KHV
CDX
RC46

Figure S14

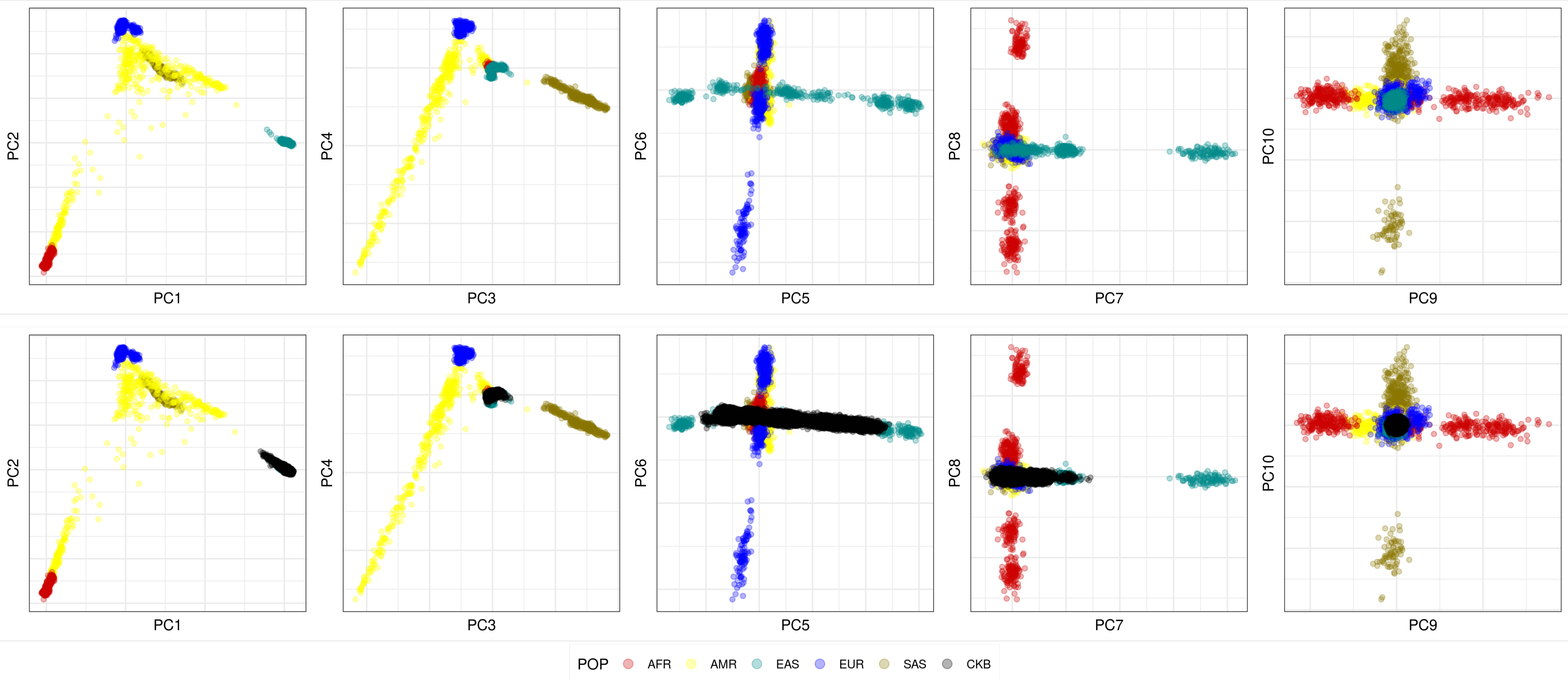
PC2
PC1
PC4
PC3
PC6
PC5
PC8
PC7
PC10
PC9
POP
AFR
AMR
EAS
EUR
SAS
CKB

Figure S15

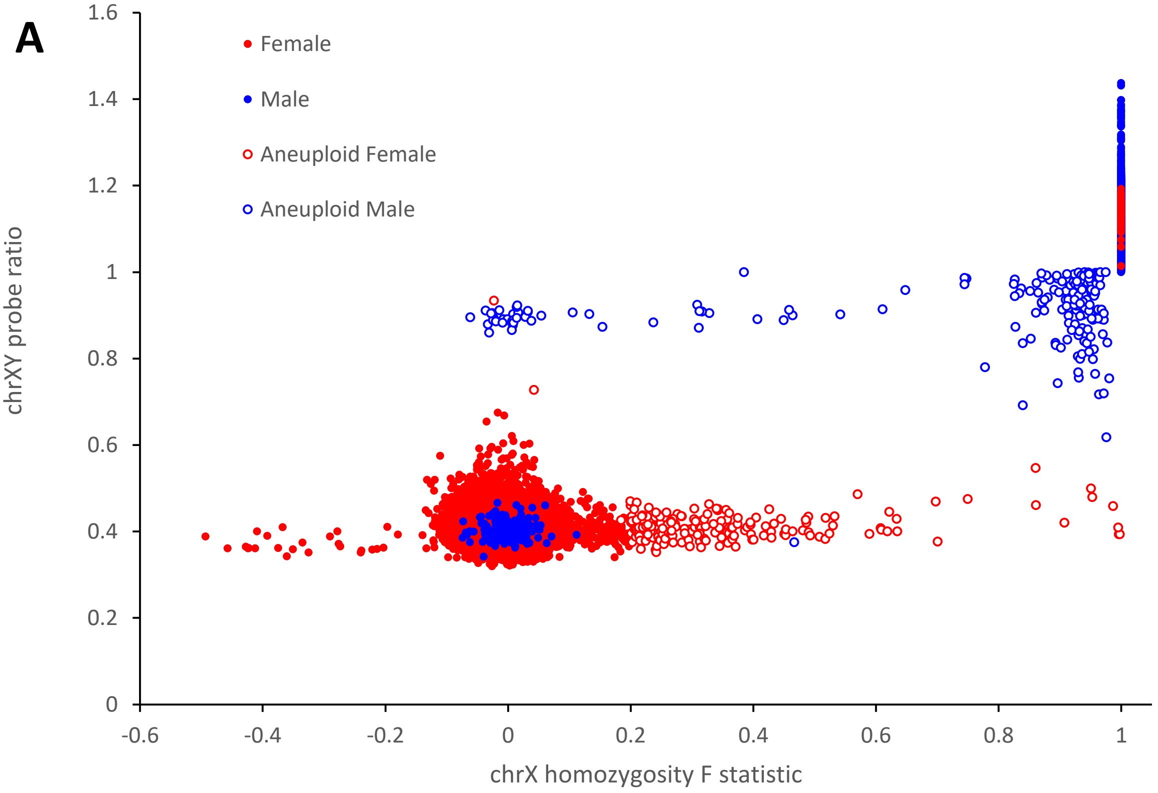
A
Female
Male
Aneuploid Female
Aneuploid Male
chrXY probe ratio
chrX homozygosity F statistic
1.6
1.4
1.2
1
0.8
0.6
0.4
0.2
0
-0.6
-0.4
-0.2
0
0.2
0.4
0.6
0.8
1

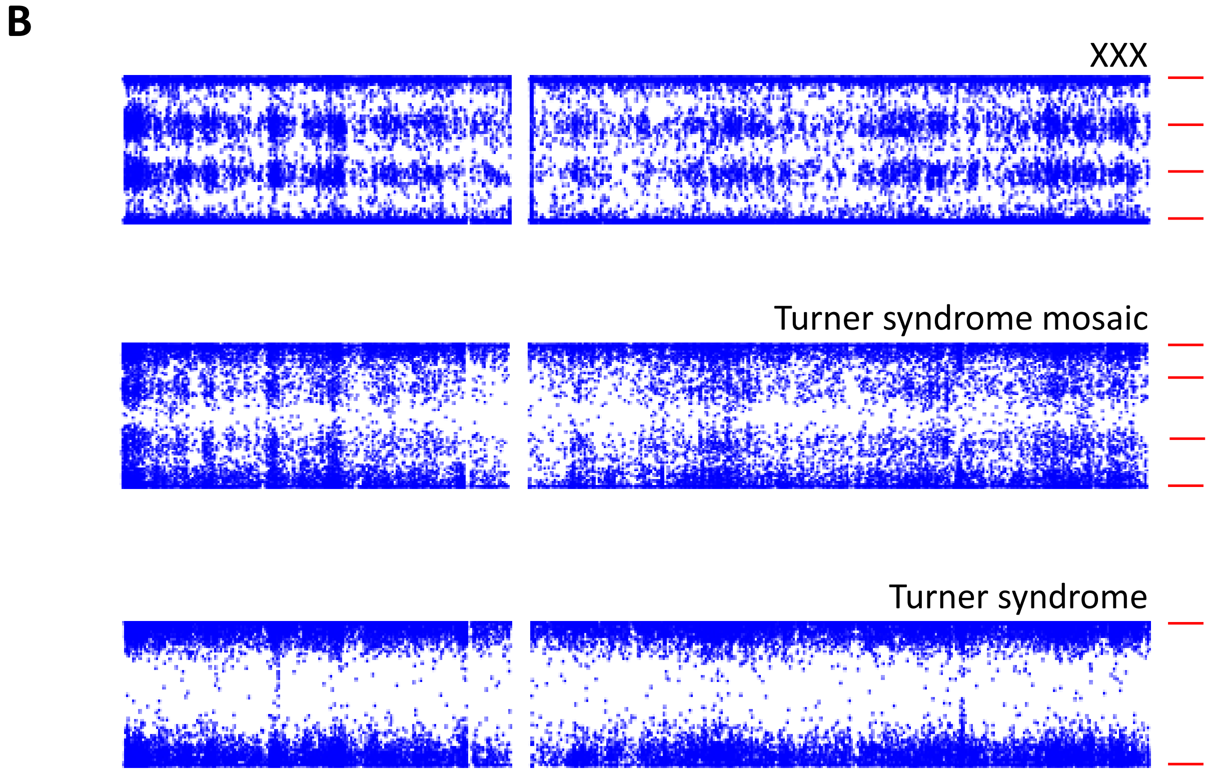
B
XXX
Turner syndrome mosaic
Turner syndrome

Figure S16

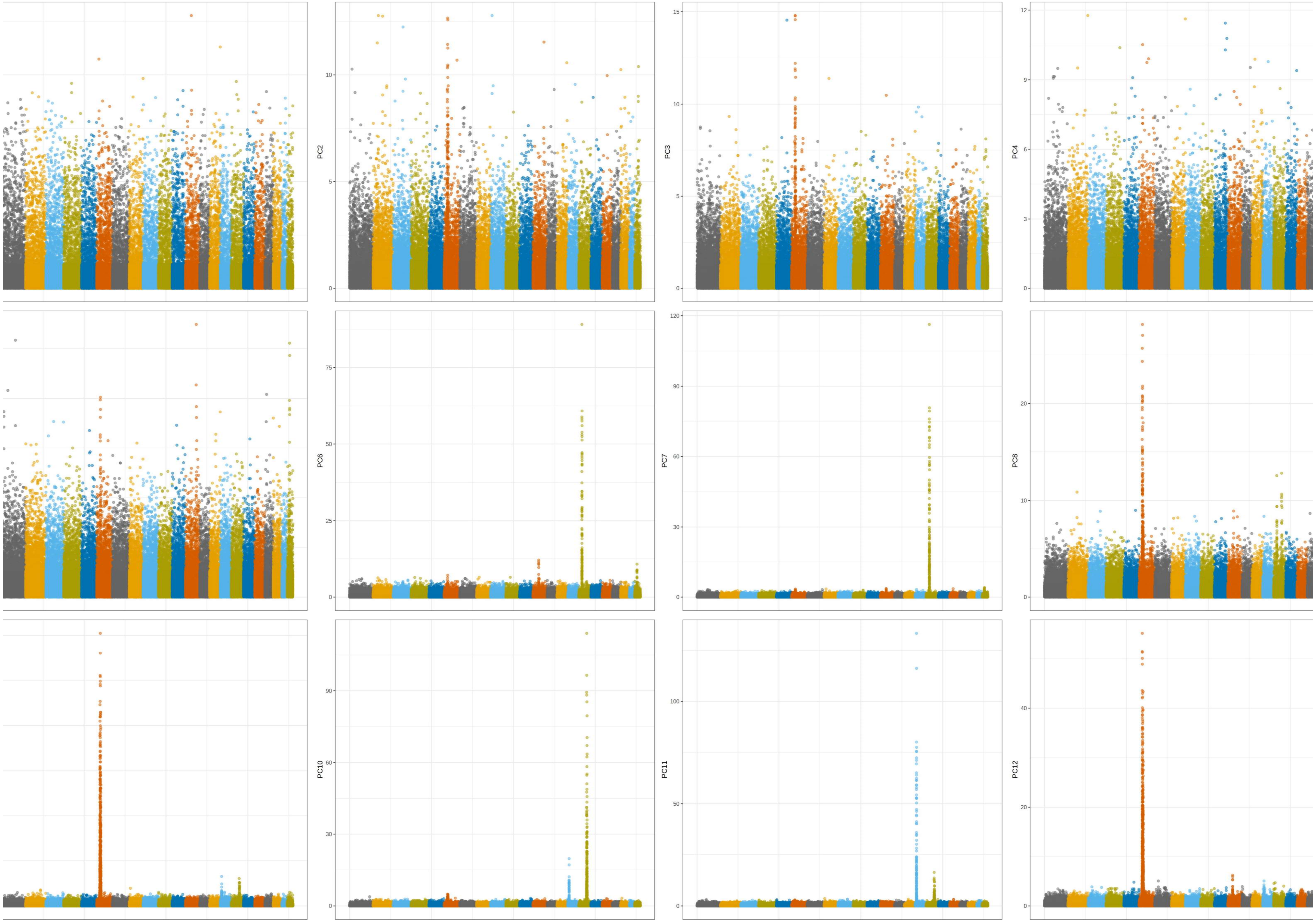

Supplementary Data S1. China Kadoorie Biobank Array Design. Related to **Figure 2.**

The overall scheme for design of the CKB array is shown in **Figure S1**. Array content was selected from 8 distinct (but overlapping) classes:

1. Variants specified for various purposes by the CKB study group and collaborators;
2. Known GWAS hits present in the GWAS Catalog plus additional loci provided by colleagues and collaborators prior to publication;
3. Putative 'functional' variants identified in BGI sequencing data;
4. Content from (non-GWAS) modules defined for the UK Biobank array design;
5. Content from (non-GWAS) modules from Affymetrix catalogue arrays;
6. The optimised CEU core GWAS module, as used in the UK Biobank array;
7. ASN (JPT/CHB/CHS) SNPs/indels identified by HapMap/1000 genomes;
8. SNPs/indels with MAF>0.01 in unpublished sequence data (BGI).
9. Viral sequences for detection of Hepatitis B virus infection/subtypes;

The data sources used to select content included:

a. 1000 genomes Phase I data from 197 CHB/CHS subjects
b. 1000 genomes pilot data from 91 CHB subjects (for SNPs absent from the Phase I data)
c. BGI high coverage WGS data for 156 of the CHS/CHB 1000genomes subjects
d. BGI WGS data from 1,746 exomes from subjects mainly from southern China
e. Allele frequency data from genotyping of 1,802 subjects using the Taiwan Biobank array
f. Low coverage sequencing data from ~9,000 Chinese from the CONVERGE consortium

Together, these were used (A) to define pre-specified content on the array; and (B) to build a Chinese-optimised GWAS grid.

For use in SNP selection (and for determining MAF-defined SNP target lists), allele frequencies from (f) were used where available. Otherwise, data from (a/b), (d), (e) were combined, except that the 1000 genomes data (a) were replaced by BGI WGS data (c) if the latter gave a non-zero number of minor alleles – these high-coverage data were expected to be more accurate than the 1000 genomes low-coverage data.

The principles underlying the array design included:

- Maximising overlap with the UK Biobank array
- Taking account of Chinese-specific content
- Ensuring detection of specific important variants
- Maximising space-efficiency of the selected variants

Thus, the UK Biobank design was taken as a starting point, with the default being to include UKB content unless there was a good reason not to do so.

A. Pre-Specified Content

Pre-specified content was determined as follows. The marker counts given are for those that survived probe design QC.

1. *Affymetrix CEU GWAS core*

The optimised CEU GWAS core (UK Biobank module 'GWAS Grid') contained 246,055 SNPs and indels. SNPs/indels were removed from this list if they had MAF<0.01 or were not present in the 1000 genomes Phase I data (used by Affymetrix for imputation aware SNP selection) and, therefore, were not able to contribute to building of the GWAS grid, leaving 191,056 SNPs. Some markers were subsequently added back due to being in other modules (see below) or during GWAS grid selection, so that in total 193,326 markers from this module were included on the array.

2. *Other UK Biobank non-GWAS module content*

Markers found in Chinese samples:

All SNPs on the UK Biobank array that had been specified for some reason other than GWAS were checked for their presence in Chinese populations. All variants identified in at least one individual in the datasets above were provisionally included. This included all variants in several UKB modules.

Markers not found in Chinese samples:

The remaining variants, for which no instance of the minor allele was identified in Chinese samples, were treated as follows:

'HLA/KIR', 'KIR', 'chrMT', 'chrY', 'ApoE', 'CNV Coverage', 'CNV tag', 'Fingerprint', 'ADME', 'Blood', 'BP/HT', 'Neanderthal', 'Alzheimers', 'eQTL', 'Lung Function': A relatively small proportion of these modules were not found in the available Chinese data. It was decided to include all such variants on the array, irrespective of detection in Chinese.

'Cancer', 'HGMD', 'cardiac', 'neuro': 6 well-evidenced cancer-related SNPs common in CEU but not already included were included. The remaining markers not already selected and present only in these modules were excluded.

'missense' or 'LOF' modules. These were mainly low-frequency variants selected on the basis of detection in the UK population. Such variants not present in Chinese were excluded.

Altogether, a further 100,411 variants were added to the array design, giving a total of 293,737.

3. *Affymetrix modules*

Markers from the Affymetrix catalogue modules eQTL, Exome319 and LOF, that were not included on the UK Biobank design but were detected in one or more of our Chinese datasets, were added to the array. The additional markers totalled 33,546, giving 327,283 in total.

4. *Known GWAS hits*

The NHGRI catalogue was downloaded on 6 December 2013. 11,745 unique lead SNPs were identified. These were merged with the UK Biobank 'GWAS compatibility' module, which included some tag SNPs (i.e. some loci had both the original hit and a tag SNP) and unpublished GWAS loci, giving a total of 12,735 markers. All of these were included on the array (QC permitting), irrespective of their prior detection in Chinese populations. As a result, a further 5,626 SNPs were added to the array, giving 332,909 in total.

5. CKB Collaborative Group-selected SNPs

683 SNPs and indels were specified by the CKB group and/or collaborators – including all SNPs previously successfully genotyped on the Illumina Golden Gate platform in 100,000 subjects – many of which were already included on the array. As a result, a further 252 SNPs were added to the array, giving 333,161 in total.

6. Functional and chrY/MT SNPs from BGI data

BGI provided data for 10,662 coding SNPs novel to Chinese (many completely novel) with putative functional effects (missense and nonsense), identified in datasets (c) and (d), and also some novel chrY/MT variants. To avoid private variants or calls due to sequencing errors, those identified in 1-2 individuals were excluded. Novel chrY/MT variants from Taiwan Biobank data were also included. In total, 5,288 additional variants were included on the final array design, giving 338,449 in total.

7. Detection of Hepatitis B virus infection and type

The available aligned HBV sequence data in late 2013 were downloaded from https://hbvdb.ibcp.fr/HBVdb/HBVdbIndex and used to calculate the entropy – i.e. the amount of variation – for each site along the genome. A sliding window of 71bp was then used to calculate the average entropy along the sequences, to identify the most conserved regions suitable for probe design. Six regions of approximately 100bp were identified, one of which contained a series of sequence variants that were expected to be sufficient to distinguish between HBV Genotypes B and C (the most common in China). For each candidate probe region, sequence variation at each site was tallied. Sites with more than one variant with a frequency ≥0.01 were recorded as 'SNPs' which would be catered for during probe design. The frequencies of all other variants (i.e. with frequency <0.01) were summed and recorded as 'residual variation' at each site.

Probe design was carried out based on sites that could be treated as 2-allele SNPs for the purposes of array data analysis. 35-mers in each direction from such 'SNPs' were recorded, treating multiallele 'SNPs' within those probes as degenerate positions requiring the design of multiple probe sequences. For each candidate 35-mer, residual variation across its full length was summed, to give a parameter "risk of probe failure" – viruses with a lot of inter-individual residual variation in these regions would potentially remain undetected due to poor probe hybridisation. Different alternative probes for each candidate probe region were compared in terms of their degeneracy and risk of failure, and the location of each probe set was selected so as to minimise these parameters.

For the majority of probe regions, whose primary purpose was to provide 'yes/no' detection of HBV, non-overlapping probe sets were selected. However, for the region diagnostic for Genotypes B and C, multiple overlapping probesets were designed so as to ensure that all diagnostic variant sites were interrogated. 15 different probesets were designed, with degeneracy at sites with variants with frequency ≥0.01, giving a total of 123 unique sequences. These were confirmed as having no appreciable homology to the human genome. Each was present in 8 copies on the array, to improve the ability to reliably detect low copy-numbers of HBV DNA.

B. GWAS Grid Selection

Chinese-specific MAFs were estimated for each marker in latest available 1000 genomes content (Phase I) as noted above, using the available data sources. These were allocated to bins corresponding to 0.05≤MAF≤0.50 and 0.01≤MAF<0.05, representing the sets of target markers to be tagged by the GWAS grid. The candidate SNPs available for selection were the full set of CHB/CHS/JPT 1000 genomes content for which Axiom assays could be designed (i.e. taking account of potential nearby interfering variants and/or sequences with appreciable similarity to other regions of the genome).

1. Selection of SNPs to tag 1000 genomes content (Affymetrix)

Using the pre-selected markers as the starting point, the Affymetrix imputation-aware SNP selection procedure was applied, initially targeting the SNPs with MAF≥0.05. Where there was a choice of SNPs to add to the array design, SNPs were prioritised that met one or more of the following criteria:

- Axiom-validated
- Not A/T or G/C allele pairs (i.e. requiring less array 'real estate')
- Present on UK Biobank array

The imputation coverage (proportion of target markers imputable at $r^2 \geq 0.8$) for each chromosome was determined at the end of each phase, and sufficient SNPs were selected and added to the array design such as to achieve 93% coverage for each chromosome. This procedure resulted in the addition of 237,246 additional variants to the final array design.

Despite this high overall coverage, some chromosomal regions remained for which coverage was poor. These were visually identified by randomly sampling 80,000 target markers for each chromosome and plotting their imputation r^2 values against chromosomal coordinate. Coverage in these regions was improved by selecting a further 12,107 SNPs, giving a total of 587,802 markers.

2. Selection of SNPs to tag novel Chinese content (BGI)

The analysis of WGS data available to BGI that provided putative functional variants (see pre-specified content part 6) also identified numerous common and low-frequency SNPs and indels not reported in 1000 genomes CHB/CHS/JPT populations. Coverage of those not already tagged (by 1000 genomes markers that could already be imputed using the currently-selected markers) was achieved using a greedy-tagging procedure with SNP prioritisation on the basis of the same criteria as Affymetrix SNP selection, combined with a score calculated using the sequencing quality scores of tagged and tagging SNPs and the extent to which untagged variants would tag other nearby untagged variants (using pairwise LD calculated from BGI's data).

This was carried out first for tagging of SNPs with MAF≥0.05, with selection of ~60,000 SNPs, and then for tagging of SNPs with 0.01≤MAF<0.05, with selection of ~20,000 SNPs. The final number of additional SNPs selected was 80,370, giving a total of 668,172 markers.

3. Selection of SNPs to increase coverage of low frequency 1000 genomes content (Affymetrix)

To improve imputation coverage of low-frequency variants, the remaining capacity of the array was used for a further 32,529 SNPs, selected using the Affymetrix imputation aware procedure. This gave a total of 700,701 markers.

4. Final QC and array design confirmation

During final array design, a number of markers were identified for which it was not possible to design array probes or for which pairs of probes were sufficiently similar in sequence that it was necessary to exclude one of them. Where this occurred, alternative SNPs were identified where possible (e.g. tag SNPs for known GWAS hits). Otherwise, additional SNPs for low frequency coverage were added. The marker numbers given above reflect the final figures after array design.

C. Array Characteristics

Out of 700,701 markers on the array, 354,399 are also present on the UK Biobank array.

Based on data collected during the array design process, predicted coverage of 1000 genomes CHB/CHS content was as follows:

method	MAF	% r^2 ≥0.8	Mean r^2
imputation	≥0.05	93.0	0.936
imputation	≥0.01	87.1	0.901
imputation	0.01-0.05	68.6	0.788
pairwise	≥0.05	68.9	
pairwise	≥0.01	67.5	
pairwise	0.01-0.05	62.9	

Supplementary Data S2. China Kadoorie Biobank Array Revision. Related to **Figure 2.**

The overall strategy for revision of the CKB array design (summarised in **Figure S3**) was as follows:

- The overall performance of probesets on version 1 of the CKB array was assessed
- Probesets were identified for removal from the design on the basis of:
  - Redundancy (where a variant was interrogated by 2 probesets)
  - Assay failure or low quality
  - Low allele frequency (monomorphic in the first 100 plates of data and absent from other datasets), unless retained for other reasons
- Potential new content was identified including
  - Alternative assays for excluded probesets
  - Tag SNPs for excluded probesets
  - Novel content with putative functional effects
  - New GWAS hits
  - Additional content from collaborators
  - Improvements in/restoration of GWAS grid coverage

Included in this strategy was the use of the full sequencing dataset from the CONVERGE consortium[1], for both content identification and assessment of GWAS grid coverage.

A. Array Version 1 Performance

Two batches of 50 plates underwent standard QC; after exclusion of 5 plates that failed initial QC, a total of 8,995 datasets passed QC, including 98 duplicates. Genotyping of all probesets was carried out, and metrics were derived, using 0.98 as the call rate threshold.

B. Identification of Probesets to be Removed

Using the stated metrics output during genotype calling, SNPs/probesets were flagged for retention, exclusion, or review as follows:

- Retained: All HBV probesets [130 probesets];
- Excluded: Redundant probesets that were not the "preferred" probeset in either batch [57,223 probesets];
- Marked for Review (1): Probesets classed as "PolyHighResolution" or "NoMinorHom" in both batches and included in all "recommended" and "preferred" lists of probesets [585,939 probesets];
- Excluded: Probesets classed as any of "CallRateBelowThreshold", "OffTargetVariant" or "Other" in both batches [39,869 probesets];
- Marked for Review (2): Probesets classed as "MonoHighResolution" in one batch and either "PolyHighResolution" or "NoMinorHom" in the other, and included in all "recommended" and "preferred" lists of probesets [13,251 probesets];
- Marked for Review (3): Probesets classed as "MonoHighResolution" in both batches, and included in all "recommended" and "preferred" lists of probesets [30,628 probesets];
- Marked for Review (4): All hemizygous probesets from MT and chrY [1,162 probesets];

- Excluded: Probesets with a call rate <0.98 in at least one batch [8,570 probesets];
- Excluded: Remaining probesets that were not one of a pair of probesets but were not recommended in both batches [7,989 probesets];
- Excluded: Remaining probesets that in at least one batch were identified as "preferred" probesets but were nevertheless not recommended [5,964 probesets];
- Excluded: Remaining probesets that called one or both batches as "OffTargetVariant", "CallRateBelowThreshold" or "Other" [8,956 probesets];
- Excluded: Of each remaining pair of probesets, the probeset with the lower overall call rate [7,556 probesets], or if tied the lowest FLD [237 probesets], or if FLD comparison not possible the lowest HomRO [622 probesets];
- Marked for Review (5): Remaining probesets, recommended in one batch but not in the other, which were classed as "MonoHighResolution" in both batches [8,771 probesets];
- Marked for Review (6): Remaining probesets, recommended in one batch but not in the other, classed as "MonoHighResolution" in one batch and either "PolyHighResolution" or "NoMinorHom" in the other [2,246 probesets];
- Marked for Review (7): Remaining probesets, recommended in one batch but not in the other, classed as either "PolyHighResolution" or "NoMinorHom" in both batches [2,824 probesets].

Further review was carried out as follows:

1. Cluster statistics were checked for the reported FLD values. Probesets for which one or both batches had FLD<4.90 were excluded [21,528 probesets];
2. Cluster statistics were checked for the reported FLD and HomRO values. Probesets for which at least one batch had HomRO<0.4 were excluded (all of these were called as MonoHighResolution" in one batch and "PolyHighResolution" in the other) [91 probesets]. Probesets for which the non-monomorphic batch had FLD<4.90 were excluded [294 probesets];
3. These were reviewed together with probesets from (5).
   The frequencies of these SNPs in the CONVERGE dataset were checked. An appreciable number were found to have MAFs in CONVERGE that were sufficiently high that failure to identify any minor alleles was highly unlikely. Probesets corresponding to SNPs with a CONVERGE MAF>0.00158 (corresponding to $P<10^{-6}$; $P<10^{-3}$ even for probesets with 5 minor alleles in the first 2 batches of genotypes) were excluded [1456 probesets].
   Probesets for SNPs that were not found (at whatever frequency) in CONVERGE were excluded unless they were originally included on the array in one of the modules HLA/KIR (193), ApoE (493), Fingerprint (0), Neanderthal (887), LOF (2060), Ax-LOF (1535), GWAS hits (247), novel nonsense (39) [31,907 probesets].
4. Hemizygous SNPs were reanalysed with an updated version of SNPolisher, and treated as follows: (a) exclude duplicate probesets that were not preferred in either batch [65 MT probesets]; (b) exclude probesets for SNPs monomorphic in both batches, Taiwanese data and (for chrY) 1000 genomes CHB/CHS [660 chrY probesets, 62 MT probesets]; (c) examine cluster plots to select between remaining pairs of duplicate probesets, for similar-quality clustering selecting the probeset with higher call rate (or excluding them both) [17 MT probesets].
5. These were reviewed together with probesets from (3), see above.
6. Cluster statistics were checked for the reported FLD and HomRO values. Probesets for which at least one batch had HomRO<0.4 were excluded (all of these were called as

MonoHighResolution" in one batch and "PolyHighResolution" in the other) [16 probesets]. Probesets for which the non-monomorphic batch had FLD<4.90 were excluded [75 probesets];

7. Cluster statistics were checked for the reported FLD values. Probesets for which one or both batches had FLD<4.90 were excluded [282 probesets];

Additional probesets were excluded as follows:

Autosomal SNPs whose minor allele frequency gave an expected minor homozygote count of at least 5 (from review classes 1 and 7) were tested for Hardy-Weinberg disequilibrium. With Holm-Bonferroni multiple testing correction (5% family-wise error rate), probesets with $P<1.13 \times 10^{-7}$ were excluded [2,734 probesets].

To further check SNPs with low MAF, the total minor allele count was extracted for those polymorphic SNPs still under consideration that were not "PolyHighResolution" in either batch (review classes 2 and 6). There was no obvious excess of SNPs with low minor allele count (1-3 minor alleles). Inspection of selected cluster plots did not indicate any problems.

C. Restoration of selected "monomorphic" SNPs

The list of exclusions was checked for Fingerprint, CKB group, and GWAS hits and these were reviewed (2,410 in total).

7 Fingerprint SNPs restored

55 SNPs specified by the CKB group or collaborators were restored

For GWAS hits:

- Marginal call rate or QC failures were restored [776 variants];
- Variants with lower call rate were restored, but were not used in constructing the GWAS grid [159 variants];
- Monomorphic or near monomorphic probesets that failed initial QC were excluded.

After all exclusions and restorations were complete, 586,528 probesets were retained.

D. Selection of New Content

Novel content was defined as follows (some variants were included for more than one reason):

1. Novel functional content

Coding variants (nonsynonymous, stop gain, etc.) were identified from CONVERGE. These were filtered to remove previously considered variants; this was achieved by excluding SNPs for which no 1000 genomes project frequency information was available. The remaining 72,332 variants were analysed by multiple functional prediction algorithms using TABLE_ANNOVAR. The results from these algorithms were combined to give an average score for whether a variant was deleterious – (sum of deleterious predictions)/(total number of predictions). Predictions were classed as deleterious as follows: SIFT – D=1; Polyphen_HDIV – D=1, P=0.5; LRT – D=1; Mutation_taster – A=1, D=1; Mutation_assessor – H=1, M=0.5; FATHMM – D=1; RadialSVM – D=1; LR_score – D=1.

Variants were selected for inclusion on the array if they had a score ≥0.5 derived from at least 3 algorithms, and had a variant-calling info score ≥0.1. As a result 9,619 variants were identified for addition to the array.

2. *New GWAS hits*

The NHGRI catalogue was downloaded on 28/4/15. There were 7,523 new entries since the list used for the original array design, of which 6,157 were for variants not previously included in the catalogue. Of these, 2,790 were associations at genome-wide significance ($P \leq 5x10^{-8}$), for 1,386 unique variants. 307 of these were already included on the array design, 2 were HLA haplotypes, and 49 were GxG interactions (for which a much higher P-value threshold would be appropriate).

As a result, 1,028 SNPs were identified for addition to the array.

3. *Additional content from collaborators*

Various external and internal collaborators supplied lists of variants, which were checked against the current array content. 292 additional variants were included.

Preliminary analysis of the HBV probes indicated they were successfully identifying HBV infection (strong association with HBV antigen test conducted at baseline). These results informed design of a further 24 HBV probes for inclusion on the array.

4. *Alternative assays/tags for important SNPs*

Key variants, specified by the CKB group and collaborators [15 variants] or which were GWAS hits [431 variants], that failed QC were marked as requiring alternative assays. Where possible, an assay from the opposite strand was designed, otherwise 'tag' SNPs (r^2>0.9) were selected from the Affymetrix library of validated assays.

E. Building of GWAS Grid

The existing GWAS grid was patched and extended using similar procedures to those used during the original array design, with some modifications, as follows:

- Since completion of the original array design, low coverage sequence data from ~9,000 subjects from across China had become available from the CONVERGE Consortium[1]. These were used to update the allele frequency bins used to define the variant target list.
- The variant prioritisation criteria were updated to remove from consideration any variant already excluded from the array design
- Imputation aware variant selection was initially conducted simultaneously for all variants with MAF>0.01, and was halted once coverage of variants with MAF>0.05 reached 94.5% (an improvement on the previous 93%).
- Further greedy tagging of variants not covered by the 1000 genomes reference used both BGI and CONVERGE sequence data.
- Further imputation aware selection was conducted specifically targeting low-frequency variants (0.01≤MAF<0.05) and regions with poor coverage

In total a further 205,176 variants were added to the GWAS grid.

F. Array Characteristics

Out of 803,030 markers on the array, 340,562 are also present on the UK Biobank array.

Predicted coverage of 1000 Genomes CHB/CHS content is as follows:

method	MAF	% r^2 ≥0.8	Mean r^2
imputation	≥0.05	93.3	0.942
imputation	≥0.01	85.3	0.900
imputation	0.01-0.05	63.1	0.766
pairwise	≥0.05	73.9	
pairwise	≥0.01	67.5	
pairwise	0.01-0.05	62.3	

Note that there were some changes in the target sets, particularly for the low frequency bin, so these results are not directly comparable to those for the original array design.

1. CONVERGE Consortium (2015). Sparse whole-genome sequencing identifies two loci for major depressive disorder. Nature *523*, 588-591. 10.1038/nature14659.
